# Systematic Understanding of Freely Optimized Excited-State Evolution in Ir(III) Photocatalysts

**DOI:** 10.1021/acsomega.6c04785

**Published:** 2026-08-29

**Authors:** Cristabella R. Fortna, Lisa A. Fredin

**Affiliations:** Department of Chemistry, 1687 Lehigh University, Bethlehem, Pennsylvania 18015, United States

## Abstract

Polypyridyl Ir­(III) complexes have dominated the field of transition metal photocatalysis due to their diverse tunability, photostability, and microsecond excited state lifetimes. Numerous studies have addressed structure–property and structure–activity relationships of Ir­(ppy)_3_ and [Ir­(ppy)_2_(bpy)]^+^ parent complexes, using density functional theory to explain experimental excited-state reactivity of the photoactive triplet states, but few works characterize the relationship of the competing charge-transfer and metal-centered triplet states and fewer still explore photoactive archetypes beyond the Ir­(ppy)_3_ and Ir­(ppy)_2_(bpy) classes. This work employs density functional theory to characterize and quantify the excited state surfaces of six Ir­(III) parent complexes and their isomers to gain a systematic understanding of how coordination at the metal center impacts photoactivity. The photophysical impact of (1) increasing the number of Ir**–**N contacts with bpy-type ligands, (2) altering through-metal C**–**Ir**–**N contact orientation and (3) increasing ligand rigidity and conjugation with fused aromatic rings is explored by plotting projected potential energy surfaces along quantitative energetic and structural axes. This investigation provides a theoretical understanding to support Ir­(III) fame and versatility as a photocatalyst, providing quantum mechanical characterization of design principles that augment excited state lifetimes.

## Graphical abstract

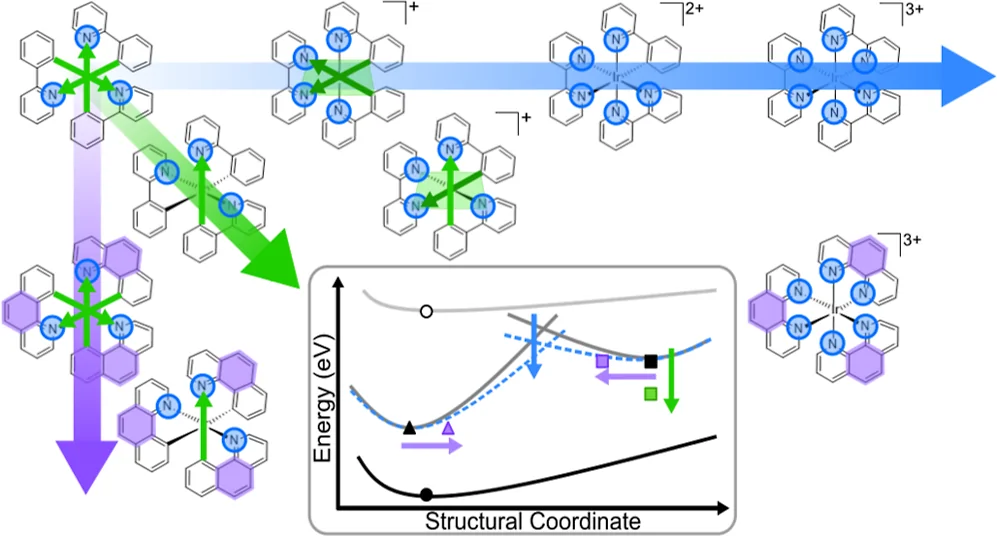

## 1 Introduction

Cyclometalated iridium III complexes have dominated the field of photochemistry for decades. With their robust excited state reactivity and facile tunability, Ir­(III) complexes are employed in organic light emitting devices,^1–3^ oxygen sensing,^4^ photodynamic and oncological therapies,^5–7^ water splitting,^8,9^ and photoredox catalysis.^4^ Many single-electron transfer (SET) reactions rely on irradiation of iridium­(III) complexes to provide a versatile way to initiate radical organic synthesis.^10–12^ While significant research efforts have focused on developing cheaper, i.e. Ru­(II),^13–16^ and more earth-abundant, i.e. Fe­(II)^17–21^ or Cu­(I),^22–25^ photosensitizers, Ir­(III) complexes are still widely used in synthesis for their versatility and reliability. Homo- and heteroleptic polypyridyl Ir­(III) complexes of the type [Ir­(C**–**N)_3–*n*_(N**–**N)_*n*_]^*n*+^, which include *fac*-trisphenylpyridine iridium (*fac*-Ir­(ppy)_3_), are widely used photocatalysts due to their robust reactivity, prolonged excited-state lifetimes, and discrete tunability from combinations of C**–**N/N**–**N type ligands and the steric and electronic modifications thereof.

Photoredox catalysts provide driving force for oxidation or reduction by creating either an excited electron that is high enough in energy to transfer to many acceptors or a hole upon excitation that can oxidize many donors. Tuning the properties of transition metal complexes to control the excited state reactivity remains a focus of many photophysical studies.^4,26–29^ Ligand design, due to its relative ease, is the dominant approach used to tune experimental reactivity.^4,26,29^ Some common modifications for polypyridyl [Ir­(C**–**N)_3–*n*_(N**–**N)_*n*_]^*n*+^ complexes include incorporating more nitrogens at the iridium center,^26–28^ adjusting the degree of conjugation and rigidity of the bidentate ligand,^26,27,30^ and other substituent effects.^4,26,27,29^ Additionally, the isomeric orientation of ligands about the iridium center alters excited state lifetimes and reduction potentials.^3,29,30^

In many popular photosensitizers, SET occurs from a high-energy triplet state as the unpaired electrons couple well with molecular donors and acceptors upon bimolecular collision in solution. There are four main types of excited states in photosensitizer complexes: metal-centered (MC), where most of the density lies on the metal center; ligand-centered (LC), where most of the density lies on the ligands; metal-to-ligand charge transfer (CT) (MLCT), where the density is redistributed from the metal and shared between both the metal and the ligands; and ligand-to-metal-charge-transfer (LMCT), where the density is redistributed from the ligands and shared between the metal and ligands. In fact, the lowest energy triplet (*T*_1_) surface is often a mixture of these domains because the geometries that stabilize the excited electron on the ligands vs the metal separate the two states structurally, even when they have very similar energies (Scheme 1). From the initial population of the triplet surface of some character, T_1*a*_, the complex can then explore other character domains of the T_1_ surface.

**Scheme 1:**
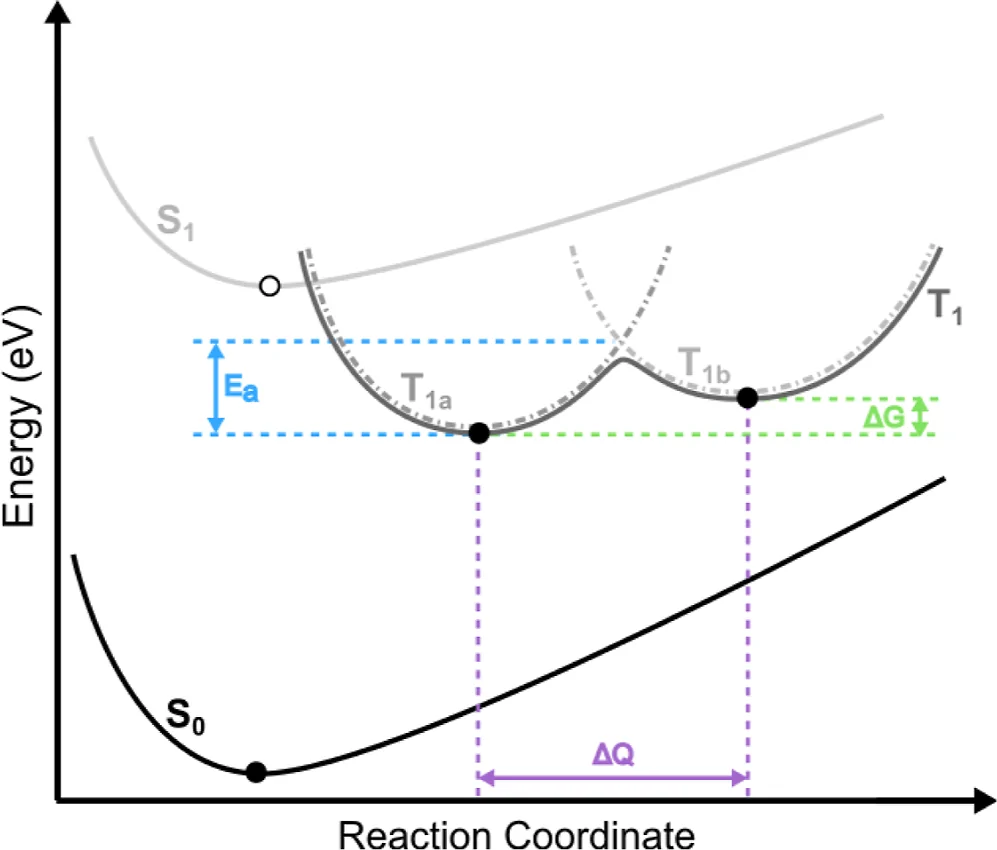
Generalized One-Dimensional Potential Energy Surface, Highlighting Parameters That Impact Excited State Lifetimes[Fn s1fn1]

a
*T*_*a*_ and *T*_*b*_ can be any combination of charge-transfer, ligand-centered, or metal-centered domains along the *T*_1_ manifold.

In these low-spin d^6^ complexes, the initially formed triplets are often MLCT in character, characterized by a spatial redistribution of electron density that leads to an excited electron on the ligands and the hole on the metal center. Populating these ^3^MLCT states requires large energies to physically redistribute the electron density so significantly. In addition, the excited electron on the ligands makes it more accessible to incoming substrates, thereby facilitating SET to or from a donor or acceptor molecule in solution through a bimolecular collision with ^3^MLCT. Effective SET requires long-lived, high-energy excited states to compete with the time scales of diffusion and bimolecular collisions in solution.

The increased density on the metal in ^3^MC states leads to highly distorted complexes as the ligands attempt to accommodate stronger repulsive forces. Additionally, for redox catalysis to occur, the electron would need to be transferred farther upon bimolecular collision with the substrate, traveling from the metal through the ligands to the substrate. Thus, ^3^MC states do not often result in the desired redox behavior of photocatalysts and instead function as deactivating excited states that compete with the desired ^3^MLCT. The presence of low-lying ^3^MC states has been shown to deactivate photocatalysts by both diminishing the population of catalytic ^3^MLCT states and leading to other chemistry like photoisomerization of the complex itself.^29–32^

Excited state lifetimes are largely governed by the activation energy (E_*a*_) between ^3^MLCT and ^3^MC minima on the *T*_1_ surface. The overall surface of the first excited triplet surface (solid dark gray curve in Scheme 1) is relatively flat. Assuming parabolic minima at both triplet subdomains (*T*_*a*_ and *T*_*b*_ in Scheme 1), the E_*a*_ is governed by the Gibbs free energy of each state (Δ*G*) and the geometric difference (Δ*Q*) between the two competing states (Scheme 1). Any design principles that increase the barrier from the ^3^MLCT to ^3^MC states would therefore increase the catalytic triplet lifetime, encouraging higher yields and minimizing off-cycle reactions. Thus, an understanding of which effects systematically alter the curvature of the T_1_ surface and the crossing of the ^3^MLCT and ^3^MC domains would provide quantum mechanical intuition for experimental reactivity and facilitate predictive photocatalyst behavior of new complexes.

While polypyridyl Ir­(III) complexes are widely used, we were surprised by the relative lack of quantum mechanical characterization of the full triplet potential energy surfaces for these complexes. Schematic surfaces with a quantitative energetic (vertical) axis and multiple optimized triplet minima can be found for some Ir­(ppy)_3_ and [Ir­(ppy)_2_(bpy)]^+^ complexes,^28,31–53^ providing generalized trends of excited state evolution. In addition, there are multiple studies that report the vertical TD-DFT triplet ^3^MLCT energy as the primary metric for the photoreactivity of the complexes,^37,54–60^ but lack quantum mechanical characterization or connection of optimized minima along a quantitative structural axis.

Here, we report the DFT ground state (GS) (singlet), ^3^MLCT, and ^3^MC geometries and potential energy curves (PECs) for six common [Ir­(III)­(C**–**N)_3–*n*_(N**–**N)_*n*_]^*n*+^ complexes, thereby quantifying changes of the excite state surfaces of under-reported [Ir­(C**–**N)­(N**–**N)_2_]^2+^ and [Ir­(N**–**N)_3_]^3+^ archetypes. As the reactivity of many transition metal complexes are sensitive to changes at the ligand, this theoretical study investigates incremental changes along three common modes of molecular design to present a systematic investigation of these effects along both quantitative geometric and energetic axes of the triplet excited state surface.

Combinatorial changes of (C**–**N)_3–*n*_ and (N**–**N)_*n*_ ligands like 2-phenylpyridine (ppy) or 2,2′-bipyridine (bpy) result in incremental variance of Ir**–**N contacts from three to six. These polypyridyl ligands have uneven electron-donating and -accepting character when coordinated to a metal, where the Ir**–**C contacts are more strongly donating than Ir**–**N contacts. Changing the number of Ir**–**N contacts therefore alters the donor and acceptor electronics of the complex, where more nitrogens on the complex favors photo-oxidant behavior.^26,61,62^ This difference in ligand–metal interaction may in turn affect the highest occupied molecular orbital (HOMO) and lowest unoccupied molecular orbital (LUMO) and excitations as the number of Ir**–**N contacts changes from three to six.

In a similar manner, the orientation of these coordinated carbon- and nitrogen-bearing ligands results in isomers with unique quantity and directionality of through-metal C**–**Ir**–**N axes. Electron-donating Ir**–**C contacts trans to electron-accepting Ir**–**N contacts result in favorable enhancement of metal back-donation from the carbon, through the iridium, onto the nitrogen. It is these same axes that result in isomeric naming of homoleptic tris-cyclometalated complexes as either *fac* or *mer*, describing the facial or meridional planes of similar atoms with respect to the metal center. The heteroleptic complex [Ir­(ppy)_2_(bpy)]^+^ has incomplete facial or meridional planes that still exhibit unique through-metal coordination axes and distinguish *f*- and *m*-isomers, which are *fac*- and *mer*-like, respectively. Complexes with more, or more favorable, through-metal coordination effects like that of *fac*-Ir­(ppy)_3_ may exhibit different excited state surfaces from isomers or related complexes with diminished through-metal relationships like *mer*-Ir­(ppy)_3_ or [Ir­(ppy)_2_(bpy)]^+^.

Without altering the coordinating atoms, ligands with fused aromatic rings like benzoquinoline (bzq) and phenanthroline (phen) increase the conjugation and structural rigidity of the ligand relative to that of the ppy or bpy parent, respectively. This is suggested to increase the visible-range absorbance of photoredox catalysts by expanding the π-network of the ligand.^63–71^ Additionally, as metal-centered states are highly distorted to accommodate the large electron density at the metal core, limiting the flexibility of the coordinated ligands may result in a less favorable ^3^MC.

Overall, this computational work investigates the effects of (1) increasing the number of Ir**–**N contacts with bpy-type ligands, (2) altering through-metal C**–**Ir**–**N orientation, and (3) increasing ligand rigidity and conjugation with fused aromatic rings on the curvature and crossing of excited state surfaces of six common archetypal polypyridyl iridium photocatalysts and their isomers (Scheme 2). The GS and triplet excited state surfaces of *fac*-/*mer*-Ir­(ppy)_3_ (*fac* **-ppy3** and *mer* **-ppy3**), *m*-/*f*-[Ir­(ppy)_2_(bpy)]^+^ (*m* **-ppy2** and *f* **-ppy2**), [Ir­(ppy)­(bpy)_2_]^2+^ (**bpy2**), [Ir­(bpy)_3_]^3+^ (**bpy3**), *fac*-/*mer*-Ir­(bzq)_3_ (*fac* **-bzq3** and *mer* **-bzq3**), and [Ir­(phen)_3_]^3+^ (**phen3**) are explored. This series of complexes achieves incremental systematic investigation of Ir**–**N, C**–**Ir**–**N, and rigidity effects for the most common archetypes of Ir­(III) photocatalysts, namely Ir­(ppy)_3_, [Ir­(ppy)_2_(bpy)]^+^, and [Ir­(bpy)_3_]^3+^, and expands upon under-reported complexes like [Ir­(ppy)­(bpy)_2_]^2+^. Relationships between the complexes’ structure and their photophysical function are mapped via the optimized minima on projected PECs like that of Scheme 1.

**Scheme 2:**
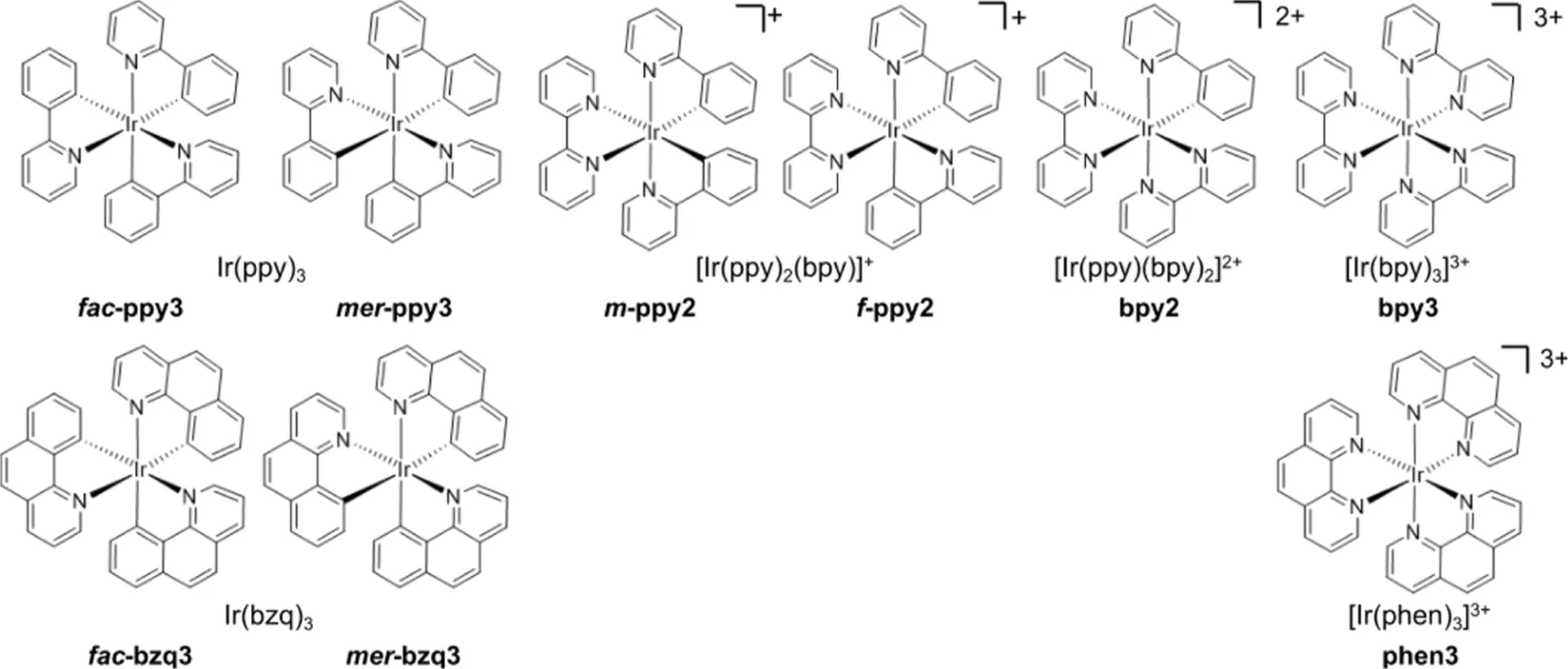
Structure and Naming of Complexes with Increasing Ir**–**N Contacts (Left-to-Right) and Increased Rigidity with Fused Rings (Bottom)^a^

a ppy = 2-phenylpyridine, bpy = 2,2’-bipyridine, bzq = benzoquinoline, and phen = phenanthroline.

## 2 Methods

GS singlet, charge-transfer (or ligand-centered), and metal-centered triplet optimizations were performed in Gaussian16 ^72^ using the PBE0 hybrid functional^73^ with Grimme’s D3 empirical dispersion.^74^ A mixed basis set consisting of the double-ζ LANL2DZ^75^ effective core potential basis on the iridium and a triple-ζ polarized diffuse 6–311G­(d,p)^76^ full-electron basis for the ligands was employed with no symmetry constraints and super fine integration grid. An integral equation formalism polarizable continuum model (PCM)^77^ was used to simulate solvent effects of acetonitrile. Optimized structures were confirmed as true minima with frequency calculations at the same level of theory. All reported energies are adiabatic and presented as relative to the respective GS of that molecule.

Mulliken spin population on Ir of optimized triplet structures was used to assign the character of the triplet, where charge-transfer populations were found to fall within 20–30%, ligand-centered <2%, and metal-centered >50% spin on Ir. Spin character was also assessed with Löwdin spin populations on the Ir using the Multiwfn program.^78,79^ On the triplet surface of each complex, multiple starting guesses were optimized separately and the lowest energy minima for each spin character are reported. The structures sampled in the search for metal-centered states, in particular, highlight broad low-energy regions across various structural features (Figures S2, S4, S6, S8, S10, S12, S14, S17, and S19). The reported ^3^MC structures are the lowest-energy freely optimized structures and are representative of the overall character of these wide and flat electronic wells.

Reduced and oxidized doublets were optimized at the same level of theory and confirmed as minima with frequency calculations. Ground- and excited-state reduction potentials (Table S11) were calculated as

$$E=\frac{-\Delta G}{nF}-E_{ref}$$

where –Δ*G* is the difference in electronic energies in J/mol, *F* is Faraday’s constant, and *n* is the number of electrons. As the dominant photoredox function of these complexes is the transfer of a single electron, *n* is taken to be unity. *E*_ref_ is the half-cell potential of the standard calomel electrode or 4.429 V in acetonitrile.^80^

Time-dependent DFT (TD-DFT)^81^ singlets of no less than 40 states provide comparable broadened spectra of ground-state absorption. The first 10 singlet ground-state excitations were characterized as natural transition orbitals (NTOs),^82^ as excitations beyond *S*_3_ are mixed-orbital transitions. Spin–orbit coupling (SOC) between the first 10 singlet and triplet excited states at the ground-state geometry was calculated with the PySOC program.^83^ The root-sum-square (RSS) of the three triplet substates are reported in the SI (Tables S13, S15, S17, S19, S21, S23 , S25, S27, and S29). The reorganization energies of crossing to the T_1*a*_ minimum from the S_1_ state at the S_0_ minimum are also reported in the Supporting Information (Table S30).

To inform the curvature and subdomain character of the triplet manifold, constrained optimizations were performed at the same level of theory between the two triplet minima. Intermediate geometries were built along the difference vector between the two triplet minima, constraining all Ir–ligand distances and the three bidentate ligand dihedral angles. Note: this difference vector is not along any one structural axis used for the projected PECs, but instead the points of the triplet surface are plotted independently for each projection. The last point of <50% spin on Ir and the first point of >50% spin on Ir are taken as the “switching neighbors” of the ^3^MLCT (or ^3^LC) and ^3^MC stepwise domains, respectively. Full constrained optimization scans of all complexes projected on multiple structural axes and twin-axis structure–structure plots are provided in the Supporting Information (Figures S50–S67).

Crossing points between spin-differentiated triplet minima were extrapolated by extending the parabolic wells through a hypothetical average geometric coordinate of the multiplicity-switching neighbors along the triplet manifold. Here, we have chosen constrained optimizations to scan the triplet manifold rather than optimized transition states between triplets of different spin character. First-order saddle points on the lowest-energy triplet surface are not necessarily a transition between states of MLCT and MC character, as a saddle point is defined energetically rather than by the spin density. There are many saddle points along these complicated and complex surfaces; a freely optimized transition state is not always representative of the chemical transition sought after.

PECs were compiled with optimized minima and single-point energies for complementary multiplicities at GS, triplet metal-to-ligand charge-transfer (^3^MLCT) or ligand-centered (^3^LC), and metal-centered (^3^MC) geometries and projected onto multiple quantitative structural axes including average Ir**–**C distance, average Ir**–**N distance, average of all Ir–ligand bonds (R) , ligand torsion, average ligand torsion (P) , and octahedricity ( O ). O is the deviation from true O_*h*_ symmetry, quantified as the average of the difference of each L–Ir–L angle from 90°. PECs along O were found to summarize the most structural changes with the greatest clarity of points, though PECs along all quantitative structural axes are provided in the SI (Figures S69–S78). Parabolic curves were drawn connecting optimized points of the same multiplicity and spin density when possible. The curvature of the triplet wells was heavily informed by the slope of the constrained optimization scans between the two minima, in some cases sacrificing parabolic behavior and intersection of individual points for a more representative slope of the full electronic well.

Minimum-energy crossing points (MECPs) between the ^3^MC and singlet GS surface (S_0_) were optimized in ORCA 6.1 ^84,85^ with the same functional, mixed basis set, and implicit solvent as those optimized in G16. These calculations employed Grimmes D3 dispersion with Becke–Johnson damping (D3BJ)^86–88^ and a conductor-like PCM (C-PCM)^89^ by default of the software. Expanded PECs that include the MECPs on the ^3^MC and S_0_ curves along the O coordinate, and deeper discussion of MECP energetics, are provided in the SI (Table S32, Figure S68, and pp.108–110). These MECPs provide another point to corroborate against other energetically quantitative works.^31–52,90–95^ Each crossing points is beyond the point of ligand dissociation (Figures S68–S78). Thus, deactivation of photoactivity is governed by the T_1_ surface, properties of the ^3^MLCT (or ^3^LC) and ^3^MC states, and the competition between these domains.

The constrained triplet scan of *fac* **-ppy3** showed a domain of mixed spin character that cannot be concretely described as ^3^MLCT or ^3^MC (Figures S50 and S51). Typical scans along the triplet manifold exhibit stepwise discontinuity of Mulliken spin at the switching neighbors. Triplet scans of *fac* **-ppy3** on any structural axis demonstrate a continuous, gradual change in spin character that is not indicative of a discrete ^3^MLCT and ^3^MC switch (Figure S50). Rather, extending the ^3^MLCT curve from the minimum along any projected structural axis (Figure S69) leads to a crossing barrier on the order of 2 eV. Along O , extending through the TDDFT-calculated T_1*a*_/S_0_ to the ^3^MC curve, the extrapolated crossing results in a barrier on the order of 2.8 eV for *fac* **-ppy3**. This extrapolated E_*a*_ supports the experimentally observed triplet lifetime of *fac* **-ppy3** of 1.9 μs^29^ and is representative of the experimentally observed photochemistry of this complex.^2,3,26,29,96^

A metal-centered minimum could not be optimized for *fac* **-bzq3**. Over 50 unique structure/calculation combinations were attempted (Figures S13 and S14). Of these, some single-point self consistent field (SCF) structures were found to be metal centered with a Mulliken spin population greater than 1.0 on Ir. However, any attempt to relax these metal-centered guesses by either constrained or free optimizations converged to the ^3^MLCT state. The lowest-energy SCF structure with over 50% spin on Ir was selected as a representative metal-centered point. A frequency calculation at the same level of theory shows four imaginary frequencies. This SCF structure follows electronic, structural, and chemical trends similar to freely optimized ^3^MC structures of other complexes, though the precise energy and structural coordinates are not fully relaxed. This overall search suggests that this reported point acts as a ^3^MC minimum, but a true ^3^MC global minimum remains elusive as much of the ^3^MC domain lies nested within the ^3^MLCT well (Figures S13, S14, S75, and S76).

## 3 Results and Discussion

Tris-bidentate polypyridyl Ir­(III) complexes are d^6^ low-spin pseudo-octahedral complexes. Deviation from true O_*h*_ octahedral symmetry can be calculated by octahedricity, O , the average of the difference of each L–Ir–L angle from 90°. True O_*h*_ complexes therefore have O = 0°, while more distorted structures have O > 0^°^. All of these complexes exhibit moderate geometric distortion ( O > 5^°^) even in the GS (Table S1 and Figures S1, S3, S5, S7, S9, S11, S15, S16 , and S18).

The frontier molecular orbitals of these Ir­(III) GSs demonstrate tremendous mixing of metal and ligand orbitals (Figures S22 –S30). For complexes with ppy and bzq ligands, the carbon-bearing moiety of the ligands holds more electron density than the nitrogen-bearing moiety (pyridine ring), demonstrating the σ-donation of the carbon. However, even the bpy and phen ligands also bear appreciable ligand density in GS HOMOs that are mostly metal *t*_2*g*_ density (Figure 1). While a N**–**Ir**–**N through-metal axis has no push–pull trans effect, the large amount of density on these ligands arises from the large π-acceptance of pyridyl moieties and the large Ir density that spreads into the conjugated ligands rather than the ligand adding density to and through the metal center. The GS LUMOs have extensive density on the ligands as expected (Figure 1). Thus, excitation into the LUMO populates the ligand-based π* orbitals. Therefore the lowest energy singlet HOMO-to-LUMO transitions are generally CT in character due to the large amount of density on the iridium that is lost upon excitation.

**Figure 1:**
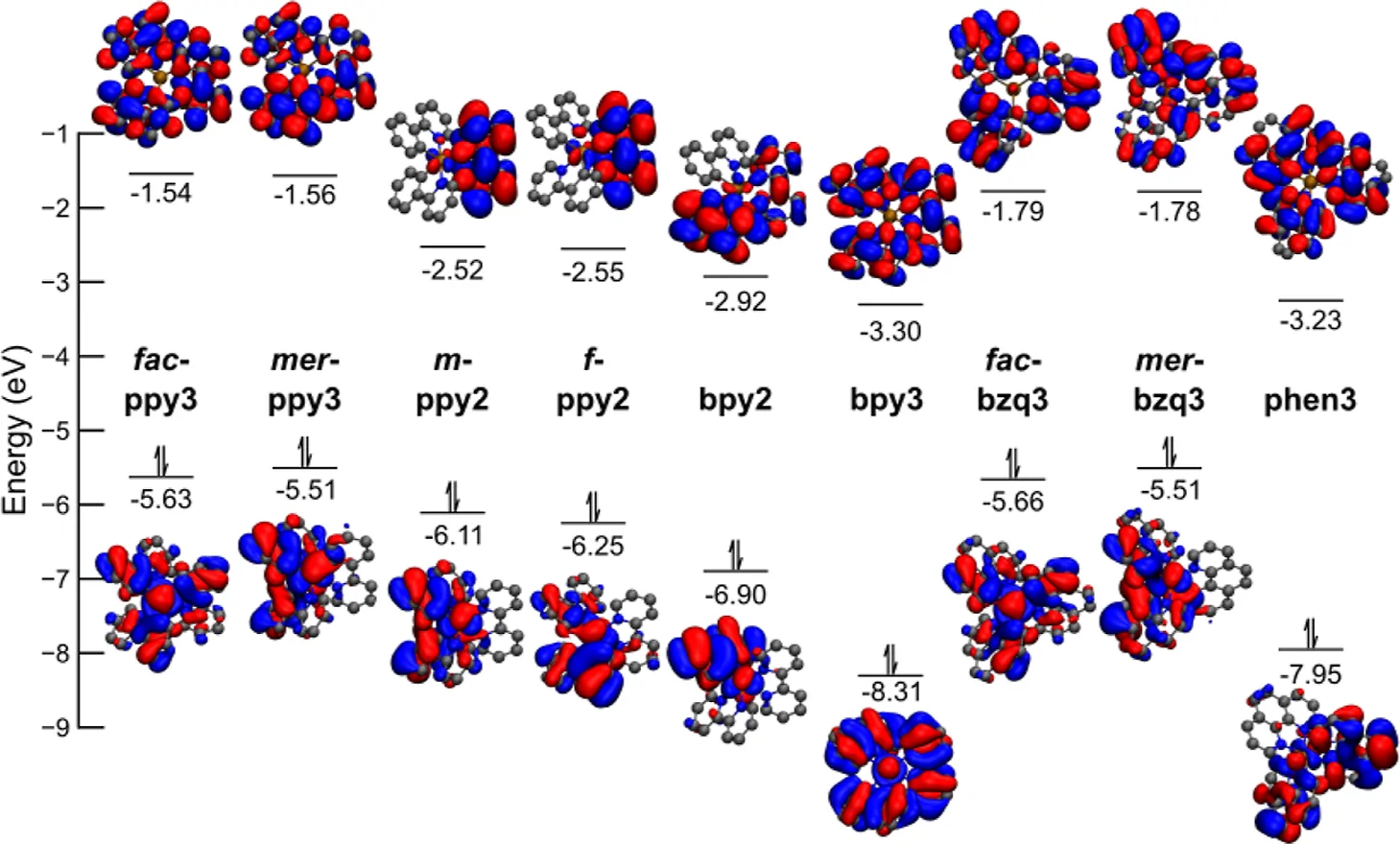
HOMO and LUMO of all GS complexes calculated at the PBE0-GD3/LANL2DZ­[Ir]+6–311G­(d,p)/PCM­(MeCN) level of theory (Isovalue ± 0.02). Hydrogen atoms omitted for visual clarity. Full molecular orbital diagrams of each complex are provided in Supporting Information (Figures S22 –S30).

CT excitations are traditionally characterized by large excitation energies and poor orbital overlap due to the large spatial difference between the molecular orbitals involved. However, the high orbital mixing of these MOs results in spatial overlap of HOMO and LUMO that is uncommon in traditional CT excitations. Thus, some of the lowest-energy TD-DFT transitions have fairly high oscillator strengths, and the predicted absorption of each complex reproduces experimental features (Figure S40 and Tables S12, S14, S16, S18, S20, S22, S24, S26, and S28). These excitations were also characterized as NTOs due to the mixed-orbital nature of higher order transitions (Figures S41–S49).

The lowest-lying triplet state for most complexes is ^3^MLCT in character as defined by the total Mulliken spin population on the Ir center (Table 1) yet there remains some electron density on the metal in the highest-energy singly occupied molecular orbital (SOMO) (Figure 2). For all nine complexes, the *T*_1*a*_ SOMO density is largely concentrated on one ligand and shows no significant trends with ligand, rigidity, or through-metal orientation. The spin density (Figures 2 and S31–S39) of the *T*_1*a*_ states of *fac* **-ppy3**, **bpy3**, and **phen3** are localized onto one ligand, while the other complexes exhibit dispersion of spin density across multiple, or all, ligands. For homoleptic **bpy3** and **phen3** complexes, this lowest-energy optimized triplet is ^3^LC in nature, with Ir spins less than 0.05 (<2%). Though the initial singlet excitation (Figures 2, S46, and S49) is classified as charge-transfer due to the tremendous contribution of Ir density that is transferred from the HOMO to the ligand π* orbitals, the spin density plots confirm a lack of comparative spin density at the freely optimized triplet state (Figures S36 and S39).

**Table 1:** Summary of Important Electronic and Structural Parameters of All Triplet Minima Calculated at the PBE0-GD3/LANL2DZ­[Ir]+6-311G­(d,p)­[C,H,N]/PCM­(MeCN) Level of Theory

complex	state	E^a^ (eV)	O (°)^b^	P (°)^c^	R (Å)^d^	spin on Ir^e^
*fac* **-ppy3**	^3^MLCT	2.60	5.59	0.83 ± 0.51	2.07 ± 0.03	0.40(0.39)
	^3^MC	3.15	13.73	14.55 ± 24.98	2.25 ± 0.33	1.08(1.12)
*mer* **-ppy3**	^3^MLCT	2.70	7.58	2.06 ± 1.32	2.08 ± 0.04	0.42(0.45)
	^3^MC	3.32	11.01	7.50 ± 9.82	2.26 ± 0.18	1.38(1.36)
*m* **-ppy2**	^3^MLCT	2.35	6.38	0.60 ± 0.23	2.06 ± 0.03	0.54(0.53)
	^3^MC	3.14	12.61	3.06 ± 1.87	2.20 ± 0.07	1.53(1.49)
*f* **-ppy2**	^3^MLCT	2.35	6.98	1.65 ± 1.39	2.06 ± 0.03	0.57(0.53)
	^3^MC	3.09	8.71	10.01 ± 7.25	2.18 ± 0.06	1.39(1.35)
**bpy2**	^3^MLCT	2.66	6.57	2.32 ± 1.47	2.05 ± 0.03	0.45(0.44)
	^3^MC	2.91	8.85	11.03 ± 9.25	2.16 ± 0.06	1.34(1.29)
**bpy3**	^3^LC	2.83	6.69	1.58 ± 0.16	2.05 ± 0.00	0.03(0.04)
	^3^MC	3.34	11.43	6.70 ± 6.57	2.18 ± 0.06	1.66(1.59)
*fac* **-bzq3**	^3^MLCT	2.51	5.99	0.52 ± 0.27	2.08 ± 0.03	0.57(0.56)
	**^3^MC** ^f^	3.99	7.11	0.39 ± 0.11	2.16 ± 0.07	1.11(1.06)
*mer* **-bzq3**	^3^MLCT	2.70	6.55	0.37 ± 0.40	2.08 ± 0.03	0.40(0.42)
	^3^MC	3.13	11.32	0.56 ± 0.76	2.21 ± 0.10	1.30(1.31)
**phen3**	^3^LC	2.80	6.11	0.92 ± 0.10	2.05 ± 0.00	0.00(0.00)
	^3^MC	3.39	8.63	2.37 ± 1.34	2.17 ± 0.06	1.54(1.47)

a Relative to the GS of the lowest-energy isomer of that complex.

b Average difference of L–Ir–L angles from 90°.

c Average ligand torsion across the bidentate dihedral.

d Average of all Ir–L distances.

e Mulliken population reported with the Löwdin population in parentheses.

f Lowest-energy SCF structure of ^3^MC character, see Methods.

**Figure 2:**
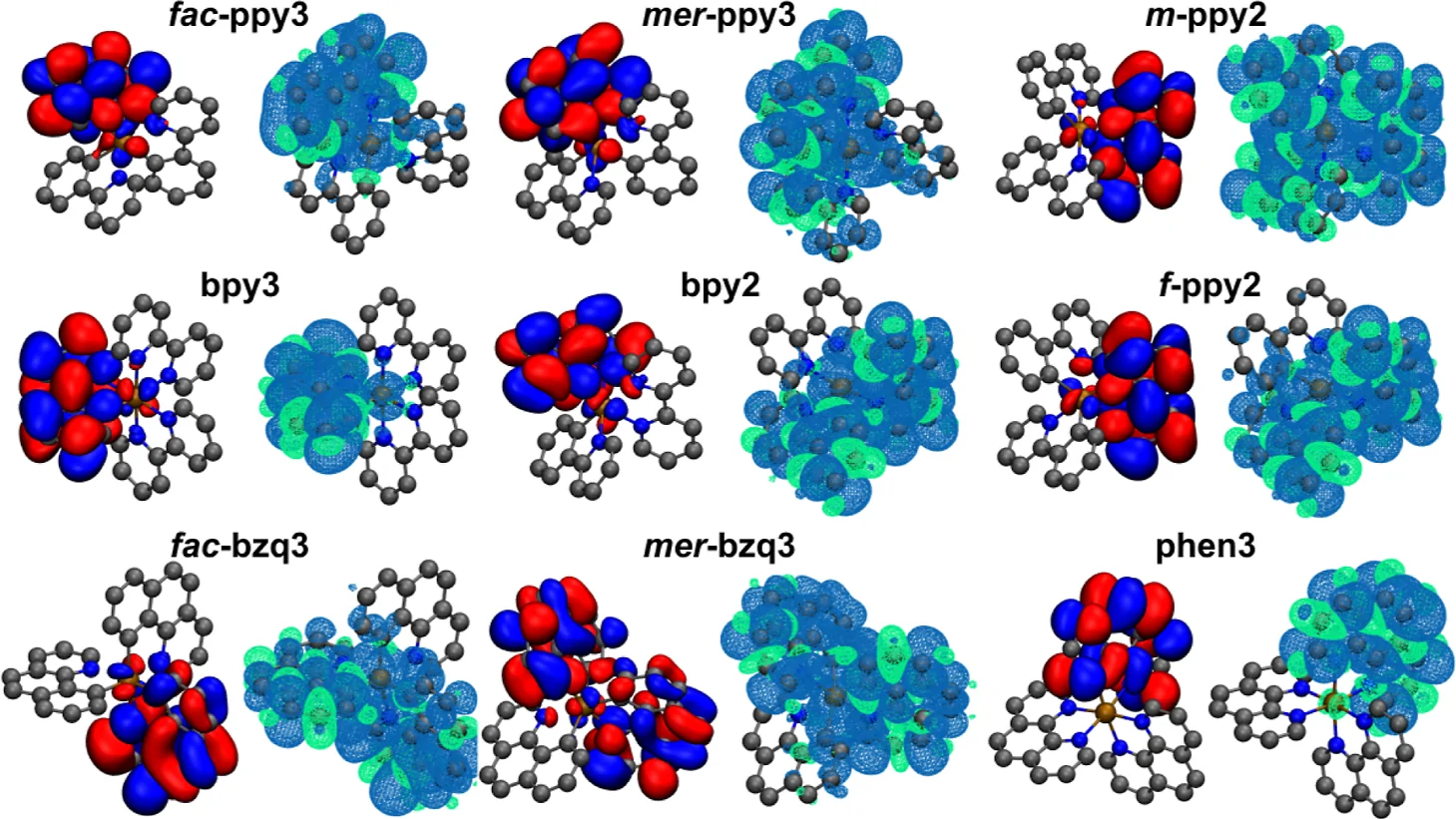
Highest-energy singly occupied molecular orbitals (left, Isolval ± 0.02) and spin density difference orbitals (right, Isoval ± 0.0004) for the lowest-energy, freely optimized T_1*a*_ state of each complex calculated at the PBE0-GD3/LANL2DZ­[Ir]+6–311G­(d,p)­[C,H,N]/PCM­(MeCN) level of theory. Hydrogen atoms are omitted for visual clarity.

Across all complexes, the *T*_1*a*_ optimized minima**—**be that ^3^MLCT or ^3^LC in character**—**range from 2.35 to 2.83 eV relative to the corresponding GS, with no significant energetic effect due to ligand, rigidity, or through-metal orientation (Table 1). Ground and excited state oxidation and reduction potentials (Table S11) show similar trends to the *T*_1*a*_ energies such that more Ir**–**N contacts favor photooxidant behavior as observed experimentally^61,62^

All ^3^MC points are ≈ 0.6 eV relative to their respective *T*_1*a*_ with minimal variation in density distribution across these complexes, bearing a through-metal nodal pattern in a line of metal-centered density (Figure 3). All freely optimized ^3^MCs, or *T*_1*b*_ minima, are 2.91–3.39 eV relative to their respective GSs (Table 1), except *fac* **-bzq3**, whose ^3^MC could not be freely optimized. In this case, the lowest-energy SCF structure with Mulliken spin > 50% was found to lie at 3.99 eV. Therefore, the ^3^MC minimum is thermodynamically inaccessible in all of these complexes and would not be initially populated upon irradiation. This triplet-to-triplet relationship is different from Ru­(II)^13^ and Fe­(II)^20^ photosensitizers, whose ^3^MC states are often lower-energy than ^3^MLCT and are more strongly influenced by subtle changes in the ligand structure.

**Figure 3:**
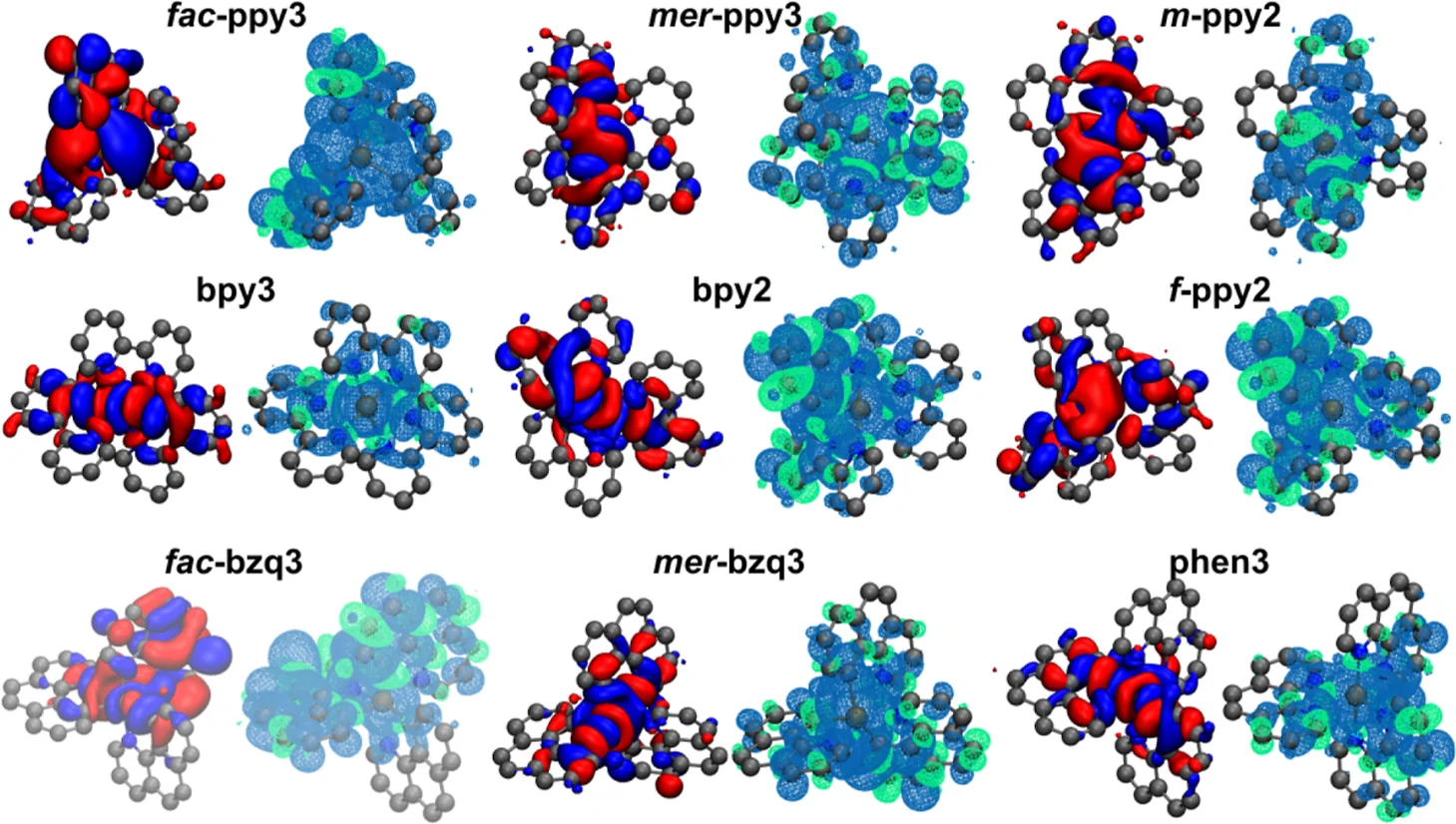
Highest-energy singly occupied molecular orbitals (left, Isolval ± 0.02) and spin density difference orbitals (right, Isoval ± 0.0004) for the ^3^MC state of each complex, with hydrogen atoms omitted for visual clarity. *fac* **-bzq3** could not be freely optimized (see Methods); the lowest-energy SCF structure is emphasized with muted opacity. Calculated at the PBE0-GD3/LANL2DZ­[Ir]+6–311G­(d,p)­[C,H,N]/PCM­(MeCN) level of theory.

Structurally, all ^3^MC complexes have increased geometric distortion as characterized by O (Table 1), which is common for transition metal photocatalysts. Adding a high energy electron onto the metal center results in larger d-orbital density. As a result, the ligands often distort in an attempt to maintain aromaticity or strong bonding to the metal. For all nine complexes, the torsion of a bidentate ligand, $p_{l}$ , is the dominant mode of structural distortion (Figure 4 and Tables S2 –S10). To accommodate the large spin density on the metal in ^3^MC states, the ligands twist, thereby breaking aromaticity across the polyaromatic dihedral angle, weakening the back-donation. The ^3^MC data presented here agrees well with the few reported Ir­(III)-^3^ MC structures, capturing the broken bidentate coordination and large dihedral twists.^28,31–33,38,53^

**Figure 4:**
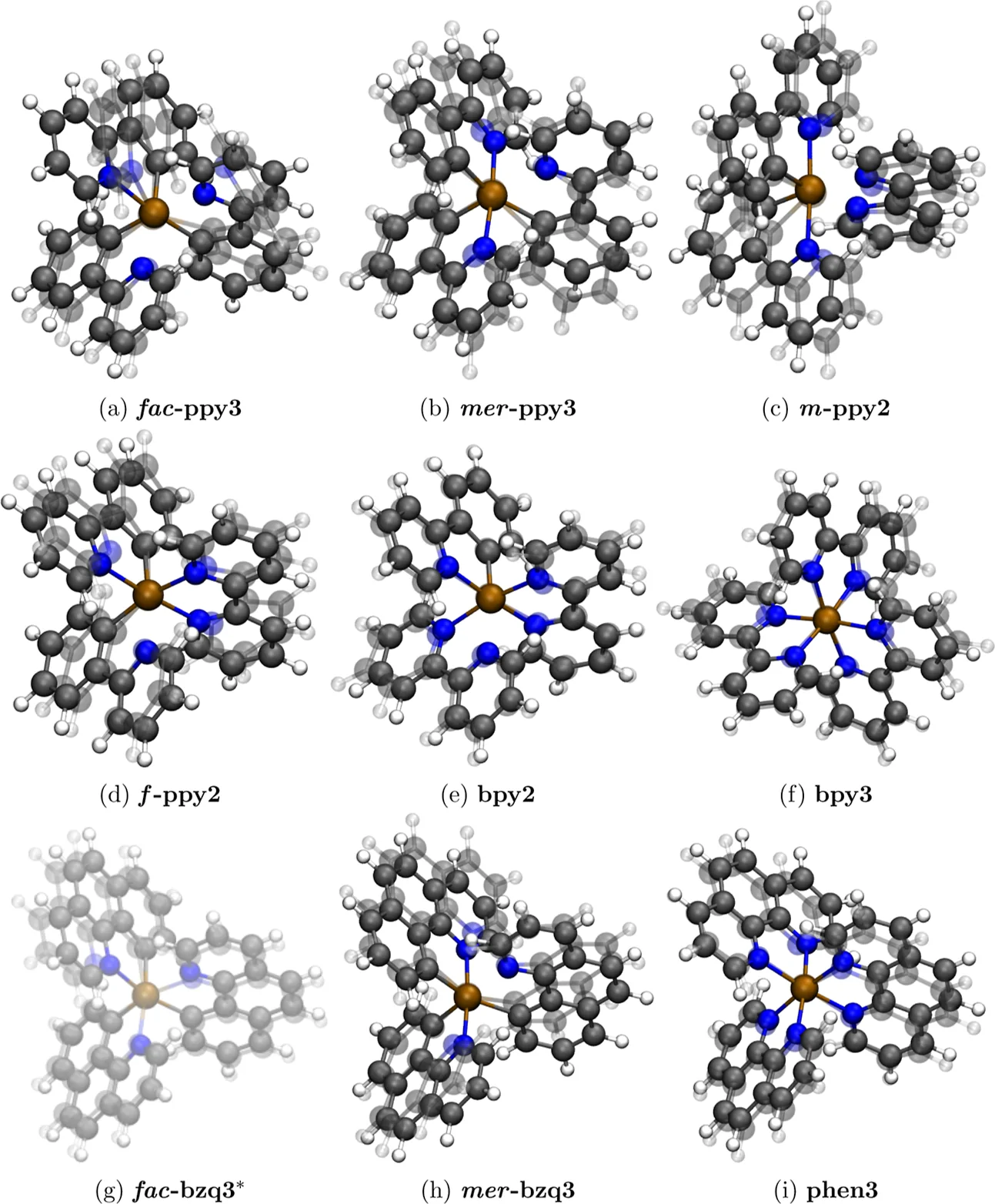
Overlay of GS and translucent ^3^MC structures calculated at the PBE0-GD3/LANL2DZ­[Ir]+6–311G­(d,p)­[C,H,N]/PCM­(MeCN) level of theory. *GS overlaid with the lowest-energy SCF structure of ^3^MC character and emphasized with muted opacity, see Methods.

Examining the average of all six Ir–L distances (R) , neither the average Ir**–**C, nor the average Ir**–**N distances alone reveal the amount of structural rearrangement in the ^3^MC states (Table 1). While the population of these ^3^MC states results in some inflation of $r_{l}$ , the average Ir–L distances of one bidentate ligand, this is a direct result of ligand torsion (Tables S2 –S10). For example, the ^3^MC state of *fac* **-ppy3** has a 43.39° twist of only one ppy ligand (Figure 4a, Table S2), resulting in an $r_{l}$ of 2.64 Å. Hidden within this average, however, the Ir**–**C bond remains at 2.06 Å, while the Ir**–**N distance stretches to 3.21 Å from the twisting. Therefore, the inflation of $r_{l}$ from GS to ^3^MC is a result of twisting of the ppy- and bpy-type ligands that asymmetrically distorts r_Ir–C_ and r_Ir**–**N_ distances.

Mapping the optimized GS, ^3^MLCT or ^3^LC, and ^3^MC states along a structural coordinate results in a doubly quantitative potential energy surface projected onto a chemically intuitive one-dimensional axis. These PECs are a useful tool to understand excited state chemistry and photoevolution pathways,^13,20,97–101^ as long as the projection axis is chosen well. For these complexes, O captures the distortion of ^3^MC states and summarizes changes in planarity and bond distance simultaneously. This brings the extreme ligand torsion of 43.39° (Table S2) and 0.31° (Table S8) onto a similar scale. PECs projected on other quantitative structural coordinates (r_Ir**–**C_, r_Ir**–**N_, R , $p_{l}$ , and P ) are provided in Figures S69–S78. Note that all projections obscure some character of the full multidimensional potential energy surface by the very nature of their projection. Combining singlet (circles) and triplet (^3^MLCT triangles, ^3^LC stars, and ^3^MC squares) freely optimized minima (filled markers) with complementary energies (open markers) obtained by SCF and TD-DFT allows mapping of the excited state evolution. The curvature and expected crossings between the spin-differentiated *T*_1*a*_ and ^3^MC minima were informed by constrained optimizations along the triplet manifold (Figures S50–S67). This combination of computational methods to describe excited states is common among other photophysical literature studies that report energetically quantitative electronic wells.^31–52,90–95^

The Ir­(III) complexes’ PECs (Figure S69–S78) highlight the various ligand, rigidity, and through-metal donation effects on the excited state surface. The energetic relationship of these curves agrees well with other energetically quantitative wells of [Ir­(C**–**N)_3_] and [Ir­(C**–**N)_2_(N**–**N)]^1+^ complexes,^28,31–34^ showcasing the high-energy ^3^MC across all Ir­(III) complexes that lies at ≈ 0.6 eV relative to the lower-energy *T*_1*a*_ minimum. Previously reported ^3^MC states of *fac* **-ppy3** describe similar ligand torsion that elongates the Ir**–**N distance to 3.37^28^–3.39^31^ Å, which lies 0.46^31^–0.47^28^ eV relative the ^3^MLCT minimum and agrees with data calculated here (3.21 Å at 0.55 eV relative to the ^3^MLCT). Additionally, other reported *m* **-ppy2** ^3^MCs of the same character furnish elongated Ir**–**N distances of 2.51 ^28,34^ –2.61^33^ Å that push the bpy ligand outward to 2.23 ^28,33,34^ Å and lie 0.62 ^33,34^ –0.90^28^ eV above the ^3^MLCT. These data agree with our findings of an ^3^MC that stretches the Ir**–**N to 2.53 Å, pushes the bpy moiety out to 2.24 Å, and sits 0.79 eV above the ^3^MLCT minimum. Further, the barriers between triplet minima calculated as the difference between spin neighbors along constrained optimization scans of **ppy3** and **ppy2** complexes (0.77–1.20 eV; Table S31 and Figures S50–S67) agree well with optimized transition states between spin-differentiated triplet minima reported by other groups, ranging from 0.33 to 0.96 eV,^28,31–34^ where the **ppy3** complexes have the lower barriers as found here. MECPs between the ^3^MC and S_0_ lie beyond the point of ligand dissociation, placing them beyond a reasonable range of quantitative structural axes for most of these complexes (Figures S68).

Though overall trends on distortion, electronic character, and excitation are minimal across the full set of nine complexes, (1) increasing Ir**–**N contacts by adding bpy ligands, (2) increasing ligand rigidity and conjugation with bzq and phen ligands, and (3) through-metal coordination effects reveal much more insightful control of excited state chemistry.

### 3.1 Increasing Ir**–**N Contacts by Increasing bpy Ligands

To investigate the effects of altering the ratio of Ir**–**C to Ir**–**N contacts, ppy ligands of **ppy3** were sequentially exchanged with bpys to reach **bpy3**. A systematic comparison of increasing Ir**–**N contacts in this Ir­(III) complexes is therefore achieved by highlighting the trends from ***fac*-** and *mer*-**ppy3** to ***m*-** and *f*-**ppy2** to **bpy2** to **bpy3**, which span three, four, five, and six Ir**–**N contacts, respectively.

In the GS structures, increasing Ir**–**N contacts by increasing bpy ligands results in greater O distortion (Table 2). This is a result of greater torsion at the bpy ligand backbone upon coordination, where more bpy ligands results in larger average ligand torsion, P . Across this series, however, the average of all Ir**–**L bonds, R , varies only slightly with each substitution of a ppy for bpy.

**Table 2:** Effects of Increasing Ir**–**N Contacts on GS Structural Parameters, Calculated at the PBE0-GD3/LANL2DZ­[Ir]+6-311G­(d,p)­[C,H,N]/PCM­(MeCN) Level of Theory

complex	Ir**–**N contacts	O (°)^a^	R (Å)^b^	P (°)^c^
*fac* **-ppy3**	3	5.70	2.07 ± 0.03	0.66 ± 0.06
*mer* **-ppy3**	3	5.84	2.07 ± 0.02	0.95 ± 0.54
*m* **-ppy2**	4	6.53	2.07 ± 0.02	1.28 ± 0.81
*f* **-ppy2**	4	6.31	2.07 ± 0.02	1.00 ± 0.47
**bpy2**	5	6.70	2.06 ± 0.02	1.25 ± 0.54
**bpy3**	6	6.89	2.05 ± 0.00	1.77 ± 0.11

a Average difference of L**–**Ir**–**L angles from 90°.

b Average Ir**–**L distances.

c Average ligand torsion through the bidentate dihedral.

Electronically, the symmetry of these complexes is impacted as a direct result of changing the number of bpy ligands chelating to the Ir center. Lower-symmetry complexes with more Ir**–**N contacts have lower symmetry HOMO densities (Figure 1). High-symmetry homoleptic complexes like *fac* **-ppy3** and **bpy3** have disperse GS HOMO density that spreads from the metal across all three ligands. Reducing the degree of rotational symmetry collapses the HOMO density onto the ppy ligand(s) in *m* **-ppy2** and **bpy2**. Similarly, for the GS LUMO, the homoleptic complexes *fac* **-ppy3** and **bpy3** have even density distribution on all three ligands. For ***m*-** and *f*-**ppy2**, **bpy2**, and **bpy3**, the LUMO density collapses onto however many bpy ligands are present; one, two, or three, respectively.

Exchanging the coordinating atom from carbon to nitrogen also results in an increasing overall complex charge with each bpy substitution. The contributions of changes in charge and heteroatoms cannot be cleanly separated, but frontier MOs are stabilized with more bpy ligands (Figure S21). The overall character of electron density is similar across the complexes (Figures 1 and S21 –S30), and thus, any changes in density distribution or excitation wavelength are more strongly correlated to structural effects and loss of *C*_*3*_ symmetry.

These changes in frontier orbital distributions affect both the wavelength and oscillator strength of the *S*_1_ absorption (Figure 5). The *m* **-ppy2** complex has the lowest-energy onset of absorbance at 457.10 nm, but with the lowest oscillator strength in this subset (0.0004). Both *fac* **-ppy3** and **bpy2** have similar wavelength (396.27 and 395.72 nm) and oscillator strength (0.016 and 0.014) of the first singlet excitation. The **bpy3** complex has the highest-energy onset of absorbance, with the first singlet excitation occurring at 299.45 nm (*f* = 0.242). Increasing the number of Ir**–**N contacts also favors the photooxidant behavior of the complexes in both the ground- and excited state potentials (Table S11), which agrees well with experimental trends.^26,26,61^

**Figure 5:**
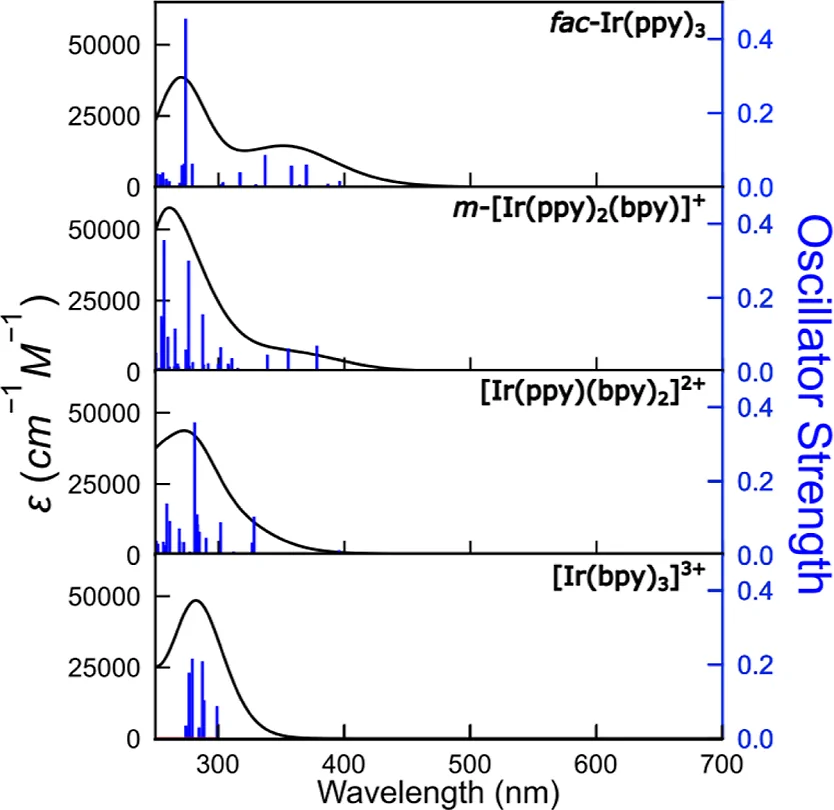
Effects of increasing Ir**–**N contacts on GS singlet TD-DFT transitions, calculated at the PBE0-GD3/LANL2DZ­[Ir]+6–311G­(d,p)­[C,H,N]/PCM­(MeCN) level of theory (blue stokes) with the corresponding Gaussian-broadened absorbance spectrum (black) for the preferred isomer complexes of three (top), four, five, and six (bottom) Ir**–**N contacts.

In the T_1*a*_ state, the SOMO density is localized on one ligand (Figure 2). For the bpy-containing complexes (*f* **-ppy2**, *m* **-ppy2**, **bpy2**), this density lies solely on a bpy ligand, rather than the more strongly σ-donating ppy ligand. Interestingly, the spin densities (Figures 2 and S31 –S39) show the opposite trends with the majority of the spin on ppy ligands. Following the GS trends, increasing the number of Ir**–**N contacts results in the greater O of T_1*a*_ structures, though the difference in distortion from GS to ^3^MLCT (or ^3^LC) is minimal. Though there is minimal variation in the energy of the initially excited triplet state, *m* **-ppy2** and *f* **-ppy2** have the lowest-lying ^3^MLCT at only 2.35 eV. Collectively, the similarity of GS LUMO and ^3^MLCT SOMO, the high-energy ^3^MLCTs that are nearly isoenergetic to the lowest singlet excited state, and the strong SOC between *S*_1_ and T_1_ states (Tables S17 and S18), as well as the small *S*_1_ to *T*_1*a*_ reorganization energy (Table S30), suggest facile and rapid intersystem crossing of both *m* **-ppy2** and *f* **-ppy2**. Their rapidly formed high-energy catalytic triplet state supports the popularity of **ppy2** derivatives as photosensitizers.^5,7,9,54,102^

The ^3^MC triplet is higher energy than the ^3^MLCT (or ^3^LC) and is not impacted by increasing the number of bpy ligands. Increasing the number of Ir**–**N contacts appears to have no effect on SOMO distribution of the ^3^MC state, as all complexes exhibit a through-complex nodal pattern (Figure 3). The ^3^MC spin densities (Figure 3 and S31 –S39) show some spread of the metal spin density into the ligands along the push–pull C**–**Ir**–**N axes. The degree of geometric distortion of the ^3^MC is correlated with the number of bpy ligands, as O decreases with increasing bpys until *C*_3_ symmetry is restored at **bpy3** (13.73°, 10.93°, 8.85°, and 11.43° for *fac* **-ppy3**, *m* **-ppy2**, **bpy2**, and **bpy3**, respectively), but is not strongly correlated with dihedral twisting (Table 3).

**Table 3:** Effects of Increasing Ir**–**N Contacts on Ligand Torsion and Ir**–**L Distances in ^3^MC Complexes, Calculated at the PBE0-GD3/LANL2DZ­[Ir]+6-311G­(d,p)­[C,H,N]/PCM­(MeCN) Level of Theory

complex	Ir**–**N contacts	$p_{l}$ (°)^a^	$r_{l}$ (Å)^b^	Ir–L_stay_ (Å)	Ir–L_twist_ (Å)
*fac* **-ppy3**	3	43.39	2.64	2.06	3.21
*mer* **-ppy3**	3	18.66	2.28	2.02	2.55
*m* **-ppy2**	4	4.23	2.12	2.02	2.24
*f* **-ppy2**	4	12.26	2.12	2.02	2.22
		15.86	2.34	2.28	2.41
**bpy2**	5	13.33	2.30	2.25	2.36
		18.91	2.17	2.07	2.27
**bpy3**	6	13.16	2.24	2.08	2.39

a Ligand torsion through a bidentate dihedral.

b Average Ir**–**L distance.

Increasing the number of bpy ligands on the complex decreases the ΔO between the triplet minima, which would be expected to increase E_*a*_ if each triplet state surface were perfect parabola (Scheme 1). Filling in points on *T*_1_ between the ^3^MLCT (or ^3^LC) and ^3^MC (Figures S50 –S67) provides a computational prediction of the curvature of the surfaces and an activation energy between ^3^MLCT (or ^3^LC) and ^3^MC where the spin of the complex switches ( $E_{a\left(T_{1}\right)}$ ). As $\Delta O_{T_{1}}$ decreases from **ppy3** to **ppy2** to **bpy2**, the $E_{a\left(T_{1}\right)}$ also decreases (Table 4), indicating that the ^3^MLCT and ^3^MC minima are not simple wells.

**Table 4:** Effects of Increasing Ir**–**N Contacts on Structural and Energetic Parameters along the Triplet Manifold That Correlate with Prolonged ^3^MLCT Lifetime, Calculated at the PBE0-GD3/LANL2DZ­[Ir]+6-311G­(d,p)­[C,H,N]/PCM­(MeCN) Level of Theory

complex	Ir–N contacts	$\Delta O_{T_{1}}$ (°)^a^	ΔG_*T*_1__ (eV)^a^	min $E_{a\left(T_{1}\right)}$ (eV)^b^	τ (μs)
*fac* **-ppy3**	3	8.14	0.55	((2))^c^	1.9^d^
*mer* **-ppy3**	3	3.43	0.62	0.77	0.15^d^
*m* **-ppy2**	4	6.23	0.79	0.98	0.28^e^
*f* **-ppy2**	4	1.73	0.74	1.20	**—**
**bpy2**	5	2.28	0.25	0.40	**—**
**bpy3**	6	4.74	0.51	0.95	**—**

a Between optimized triplet minima, ^3^MC–^3^MLCT or ^3^MC–^3^LC for **bpy3**.

b Difference between the higher-energy switch neighbor and lowest-energy optimized triplet.

c Extrapolated fit, see Methods.

d Luminescent lifetime in MeCN from Tamayo et al.^29^

e Luminescent lifetime in MeCN from ref 62.

Increasing Ir**–**N contacts by exchanging ppy ligands for bpy results in a decrease in E_*a*_ as a result of the decreased $\Delta O_{T_{1}}$ along the triplet surface (Table 4), which therefore suggests deactivation of the photocatalyst. This structural proximity of the deactivating ^3^MC and the lower barrier of populating this state from the catalytic ^3^MLCT propose shorter lifetimes and less opportunities for SET as a course of photoaction. This broad trend agrees with the experimental lifetimes of these complexes, where *fac* **-ppy3** has a longer luminescent lifetime than *m* **-ppy2** (1.90 ^29,61^ vs 0.275 ^62^ μs, respectively). Of note, **bpy2** would be expected to be significantly less active than the more widely used **ppy3** ^10–12^ or **ppy2**.^5,7,9,54,102^ The ^3^MC minimum of **bpy2** is only 0.25 eV relative to that of the ^3^MLCT state**—**the closest energetic triplet pair in this subset and of all nine complexes in this study (Table 4). In addition, the $\Delta O_{T_{1}}$ is only 2.28° for **bpy2**. The energetic and geometric proximity, low activation barrier, and shallow curvature of the excited state wells of **bpy2** (Figure 6) suggest facile population of the deactivating ^3^MC state that would result in depletion of the active catalyst.

**Figure 6:**
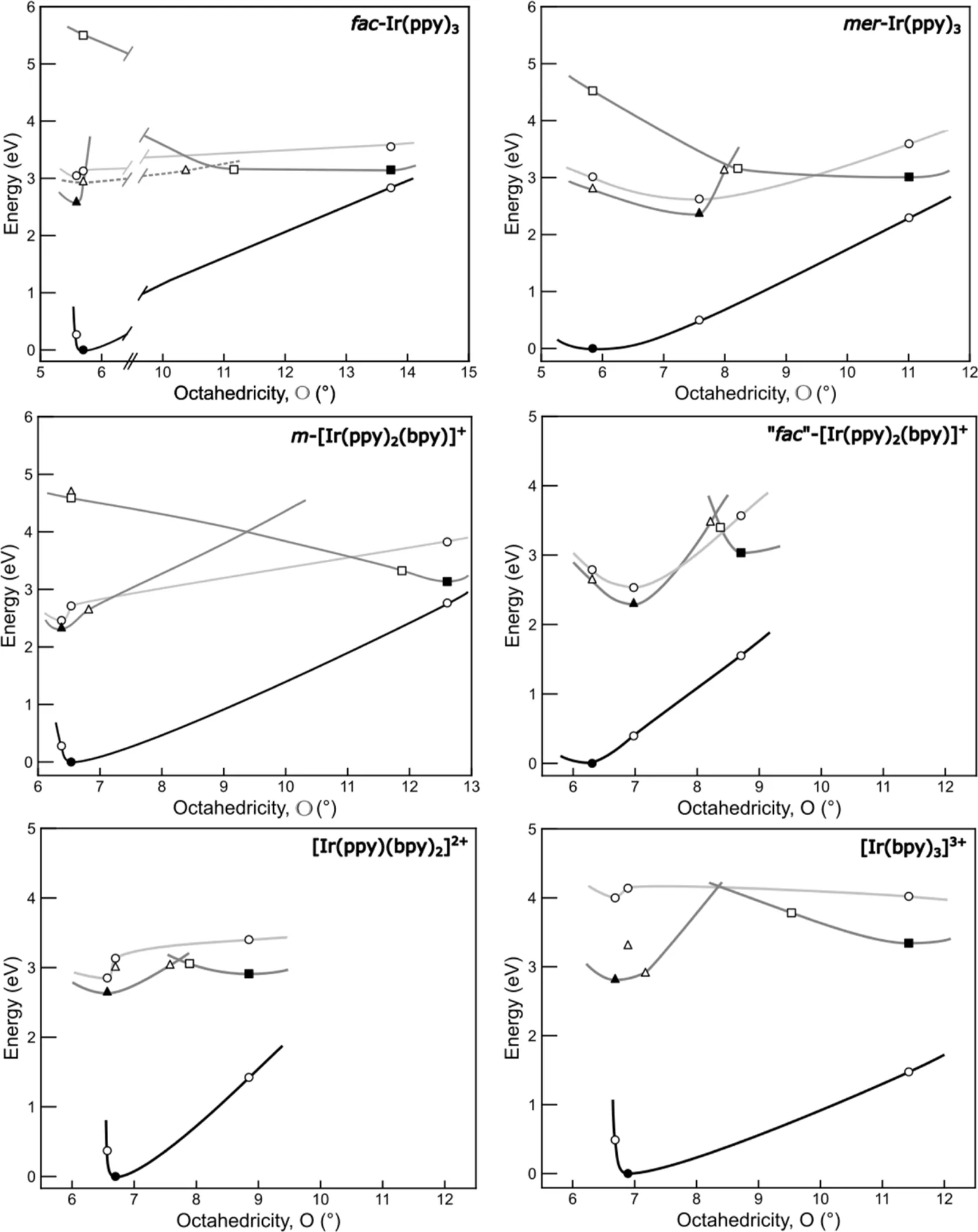
Effects of increasing Ir**–**N contact on PEC projected on O . Circles are singlet species, triangles are triplets with CT character, stars are triplets with LC character, and squares are triplets with MC character. Filled markers are freely optimized minima, while open-marker points are the SCF or TD-DFT excitation energies of that multiplicity. Other PEC projections are provided in the Supporting Information (Figures S69–S78).

### 3.2 Through-Metal C**–**Ir**–**N Coordination Effects

Transition metal complexes with π-accepting ligands and large metal centers exhibit back-bonding from the metal core onto the π* network of the ligand. In the case of ppy ligands, the phenyl portion is more strongly donating than the pyridine portion.^63,64,103^ Moreover, when the density-pushing Ir**–**C contacts are trans to the density-pulling Ir**–**N contacts, this favorable through-metal push–pull relationship enhances the stability of the complex. The number of through-metal push–pull axes is governed by both the ratio of ppy to bpy ligands and the orientation of the ligands about the Ir center. Thus, the *fac*- and *mer*- isomers of a given complex have different amounts, or different orientations, of these favorable push–pull axes. For example, *fac* **-ppy3** has three C**–**Ir**–**N axes, while *mer* **-ppy3** only has one; *f* **-ppy2** and *m* **-ppy2** both have two C**–**Ir**–**N axes, though *m* **-ppy2** has both oriented toward the bpy ligand (Scheme 3).

**Scheme 3:**
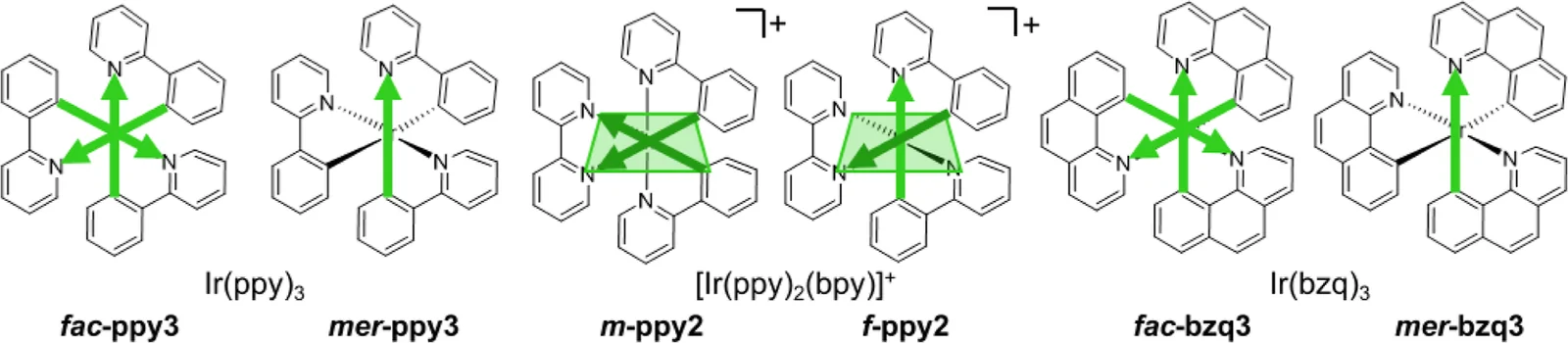
Through-Metal C**–**Ir**–**N Axes Indicated with Green Arrows^a^

a Both *m* **-ppy2** and *f* **-ppy2** have two arrows, distinguished by their relationship to the bpy-containing plane shown in green.

Reducing the amount or symmetry of C**–**Ir**–**N trans stabilizing axes increases the distortion in GS structures (Table 5). The lower-symmetry complexes with more N**–**Ir**–**N pull–pull relationships (*mer* **-ppy3**, *f* **-ppy2**, and *mer* **-bzq3**) have longer average coordination distances and more ligand torsion. Because of the large variation in metal–ligand interactions (Table 5), the average of *all* Ir**–**L bonds (R) and ligand torsion (P) do not adequately capture the distortion around the metal center on the lowest triplet surface (*T*_1_). Instead the individual (Tables S2–S10) distance ( $r_{l}$ ) and ligand torsion ( $p_{l}$ ) of one ligand (highlighted in Table 6) lead to the increase in O observed from *fac*- to *mer*- isomers (Table 5).

**Table 5:** Through-Metal Coordination Effects on GS Structural Parameters, Calculated at the PBE0-GD3/LANL2DZ­[Ir]+6-311G­(d,p)­[C,H,N]/PCM­(MeCN) Level of Theory

complex	C–Ir–N contacts	O (°)^a^	R (Å)^b^	P (°)^c^
*fac* **-ppy3**	3	5.70	2.07 ± 0.03	0.66 ± 0.06
*mer* **-ppy3**	1	5.84	2.07 ± 0.02	0.95 ± 0.54
*m* **-ppy2**	2	6.53	2.07 ± 0.02	1.28 ± 0.81
*f* **-ppy2**	2	6.31	2.07 ± 0.02	1.00 ± 0.47
*fac* **-bzq3**	3	5.00	2.08 ± 0.03	0.39 ± 0.11
*mer* **-bzq3**	1	5.14	2.07 ± 0.02	0.38 ± 0.23

a Average difference of L**–**Ir**–**L angles from 90°.

b Average Ir**–**L distances.

c Average ligand torsion through the bidentate dihedral.

**Table 6:** Through-Metal Coordination Effects on Ligand Torsion and Ir**–**L Distances in ^3^MC Complexes, Calculated at the PBE0-GD3/LANL2DZ­[Ir]+6-311G­(d,p)­[C,H,N]/PCM­(MeCN) Level of Theory

complex	C–Ir–N axes	$p_{l}$ (°)^a^	$r_{l}$ (Å)^b^	Ir-stay (Å)	Ir-twist (Å)
*fac* **-ppy3**	3	43.39	2.64	2.06	3.21
*mer* **-ppy3**	1	18.66	2.28	2.02	2.55
*m* **-ppy2**	2	4.23	2.12	2.02	2.24
*f* **-ppy2**	2	12.26	2.12	2.02	2.22
*fac* **-bzq3** ^c^	3	0.51	2.20	2.11	2.30
*mer* **-bzq3**	1	1.43	2.40	2.07	2.74

a Ligand torsion through a bidentate dihedral.

b Average Ir**–**L distance.

c Lowest-energy SCF structure of ^3^MC character, see Methods.

Altering the number and orientation of C**–**Ir**–**N push–pull axes results in spectral changes in the singlet TD-DFT excitations and broadened UV–vis spectra (Figure 7). Though these effects are difficult to generalize, there are more low-oscillator strength singlet transitions in the lower-symmetry isomers (*mer* **-ppy3**, *f* **-ppy2**, and *mer* **-bzq3**) than in their thermodynamically preferred counterparts (*fac* **-ppy3**, *m* **-ppy2**, and *fac* **-bzq3**). The lower symmetry leads to smaller ligand field splitting and fewer degenerate excitations, resulting in multiple similar, but symmetrically different, excitations each with a low probability, i.e., oscillator strength. Similarly, it is the symmetry of the complex that governs the dispersion of the HOMO in the GS (Figure 1). The high-symmetry homoleptic preferred isomers (*fac* **-ppy3** and *fac* **-bzq3**) have evenly distributed density on all three ligands, while the electron density condenses onto two ligands in the lower-symmetry complexes and isomers (*mer* **-ppy3**, *m* **-ppy2**, *f* **-ppy2**, and *mer* **-bzq3**). Through-metal coordination effects have no impact on GS LUMO distribution (Figure 1) or reduction potentials (Table S11).

**Figure 7:**
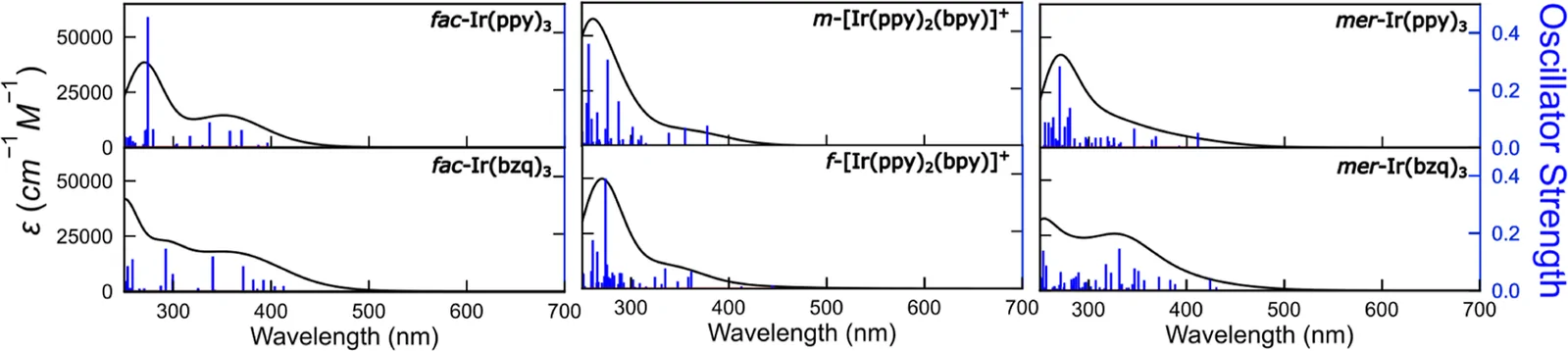
Through-metal coordination effects on GS singlet TD-DFT transitions calculated at the PBE0-GD3/LANL2DZ­[Ir]+6–311G­(d,p)­[C,H,N]/PCM­(MeCN) level of theory (blue stokes) with corresponding Gaussian-broadened absorbance spectrum (black) of three (left), two (middle), and one (right) through-metal C**–**Ir**–**N axes.

The ^3^MLCT SOMO is unaffected by altering through-metal coordination (Figure 2). Energetically, the ^3^MLCT minimum of the homoleptic complexes is 0.20 eV lower for the *fac*- isomer than the *mer*- counterpart (Table 1). There is no change for the **ppy2** complexes. Thus, the number of C**–**Ir**–**N axes governs the energetic stability of a given state more than the unique orientation of push–pull axes. Following GS trends, the less-favored isomers (*mer* **-ppy3**, *f* **-ppy2**, and *mer* **-bzq3**) have more distorted ^3^MLCT structures than their energetically favorable counterparts, which have more C**–**Ir**–**N through-metal coordination (Table 1). Further, with the exception of *fac* **-bzq3**, the ^3^MLCT structure is less distorted than the GS geometry for the preferred isomers (*fac* **-ppy3** and *m* **-ppy2**) but more distorted in the lower-symmetry isomers (*mer* **-ppy3**, *f* **-ppy2**, and *mer* **-bzq3**) (Table S1).

The nodal pattern of the ^3^MC SOMOs is consistent for all complexes (Figure 3), though the density orients along the axis of least push–pull character. In the *fac*- homoleptic complexes, the ^3^MC SOMO density falls along one of the three equivalent through-metal C**–**Ir**–**N axes, a visual demonstration of this push–pull through-metal stabilization. The homoleptic *mer*- isomers, which only have one C**–**Ir**–**N trans axis, have ^3^MC SOMO density along one of the pull–pull N**–**Ir**–**N axes instead. The *f* **-ppy2** and *m* **-ppy2** complexes also orient ^3^MC density along their pull–pull N**–**Ir**–**N axis.

Decreasing the number or symmetry of through-metal C**–**Ir**–**N push–pull axes results in less favorable excited state activity. In the less-preferred isomer (*mer* **-ppy3**, *f* **-ppy2**, and *mer* **-bzq3**), the ^3^MC minimum is both structurally (Table 6) and energetically (Table 7) closer to the GS and ^3^MLCT states (Figure 8). This decrease in $\Delta O_{T_{1}}$ and the moderate decrease in ΔG_*T*_1__ suggest shorter excited state lifetimes for less-favorable isomers. These trends agree with the experimental data of ***fac*-** and *mer* **-ppy3** with luminescent lifetimes of 1.90 ^29,61^ and 0.15^29^ μs, respectively.

**Table 7:** Rigidity and Conjugation Effects on Structural and Energetic Parameters along the Triplet Manifold That Correlate with Prolonged ^3^MLCT Lifetime, Calculated at the PBE0-GD3/LANL2DZ­[Ir]+6-311G­(d,p)­[C,H,N]/PCM­(MeCN) Level of Theory

complex	C–Ir–N axes	$\Delta O_{T_{1}}$ (°)^a^	ΔG_*T*_1__ (eV)^a^	min $E_{a\left(T_{1}\right)}$ (eV)^b^	τ (μs)
*fac* **-ppy3**	3	8.14	0.55	((2))^c^	1.90^d^
*mer* **-ppy3**	1	3.43	0.62	0.77	0.15^d^
*m* **-ppy2**	2	6.23	0.79	0.98	0.28^e^
*f* **-ppy2**	2	1.73	0.74	1.20	—
*fac* **-bzq3** ^f^	3	1.12	1.48	—^g^	—
*mer* **-bzq3**	1	4.77	0.36	0.73	**—**

a Between optimized triplet minima, ^3^MC–^3^MLCT

b Difference between the higher-energy switch neighbor and lowest-energy optimized triplet.

c Extrapolated fit, see Methods.

d Luminescent lifetime in MeCN from Tamayo et al.^29^

e Luminescent lifetime in MeCN from ref 62.

f Differences measured between ^3^MLCT and the lowest-energy SCF structure of ^3^MC character, see the Methods.

g No crossing was found; SCF-^3^MC nested within the ^3^MLCT domain.

**Figure 8:**
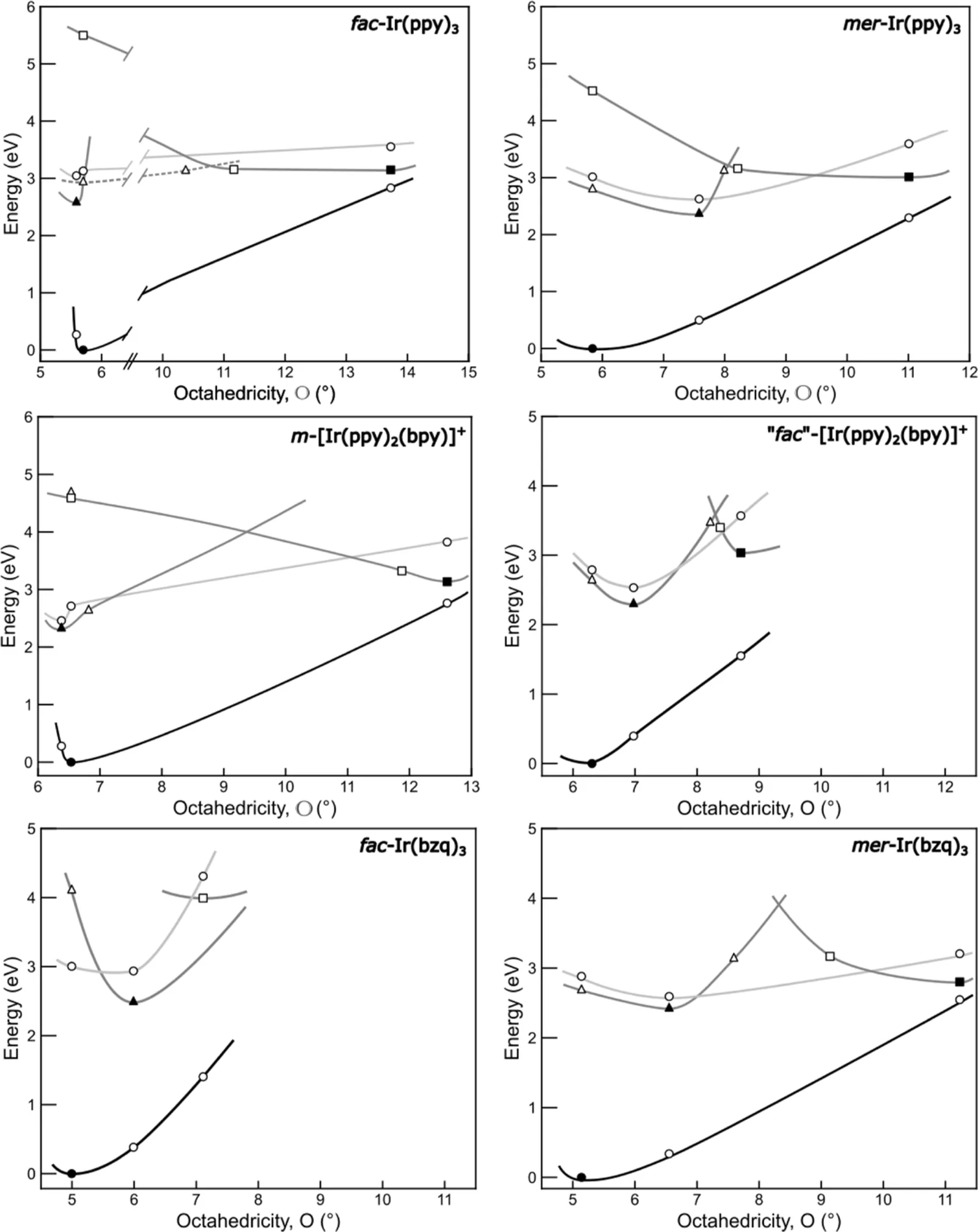
Through-metal coordination effects on PEC projected on O . Circles are singlet species, triangles are triplets with CT character, and squares are triplets with MC character. Filled markers are freely optimized minima, while open-marker points are the SCF or TD-DFT excitation energies of that multiplicity. Other PEC projections are provided in the Supporting Information (Figures S69–S78).

### 3.3 Increasing Rigidity with Fully Aromatic Ligands

Larger aromatic networks on π-accepting ligands stabilize the LUMO, red shifting the UV–visible absorbance of transition metal light harvesters.^40,63–71,104^ Limiting degrees of flexibility of complexes results in smaller geometric distortion of the excited states,^13,14,24,25,98,99,105^ which should in turn improve intersystem crossing rates. Together, more rigid and more conjugated ligands like bzq and phen may improve low-energy absorption and facilitate population of the catalytic *T*_1*a*_ state, thereby resulting in more efficient photoredox catalysis.

Replacing ppy or bpy ligands with bzq or phen, respectively, results in less GS distortion (Table 8). The ppy and bpy complexes exhibit ligand torsion as the source of deviation from traditional O_*h*_ symmetry in all of the optimized minima (Table S1). Homoleptic **bzq3** and **phen3** have longer coordination distances since their rigidity hampers torsion along the ligand backbone, leading to more O_*h*_ symmetry in the GS. Substitution for more rigid ligands does not affect the HOMO distribution or character. The rigidity and conjugation of the ligands have no significant impact on the GS LUMO.

**Table 8:** Rigidity and Conjugation Effects on GS Structural Parameters, Calculated at the PBE0-GD3/LANL2DZ­[Ir]+6-311G­(d,p)­[C,H,N]/PCM­(MeCN) Level of Theory

complex	O (°)^a^	R (Å)^b^	P (°)^c^
*fac* **-ppy3**	5.70	2.07 ± 0.03	0.66 ± 0.06
*fac* **-bzq3**	5.00	2.08 ± 0.03	0.39 ± 0.11
*mer* **-ppy3**	5.84	2.07 ± 0.02	0.95 ± 0.54
*mer* **-bzq3**	5.14	2.07 ± 0.02	0.38 ± 0.23
**bpy3**	6.89	2.05 ± 0.00	1.77 ± 0.11
**phen3**	5.98	2.06 ± 0.00	0.98 ± 0.04

a Average difference of L**–**Ir**–**L angles from 90°.

b Average Ir**–**L distances.

c Average ligand torsion through bidentate dihedral.

Most of the homoleptic complexes exhibit an even distribution of HOMO density onto all ligands, as expected from highly symmetric complexes (Figure 1). However, the freely optimized **phen3** GS has HOMO density on only two ligands, much like that of *m* **-ppy2** or *f* **-ppy2**. At room temperature, the equivalence of the three phen ligands would lead to a Boltzmann distribution that is an evenly weighted sum of the three equivalent **phen3** structures. An SCF of a *C*_3_-symmetrized **phen3** structure (Figure S20) was found to be 1.57 eV higher than the freely optimized, distorted GS. While the large aromatic systems of the phenanthroline backbone reduce additional distortion when comparing GS to ^3^MLCT or ^3^MC structures, this rigidity also reduces the allowed bidentate torsion that would relieve the bite-angle strain in a *C*_3_-symmetrized complex of this size.

The larger π-network on *fac* **-bzq3**, *mer* **-bzq3**, and **phen3** results in greater-overlap between the π to π* orbitals of the ligands. In the 250**–**300 nm region, **bzq3** has more transitions with larger oscillator strength than **ppy3**, so a shoulder peak appears in the broadened spectra (Figure 9). For **phen3**, this shoulder occurs at 200**–**250 nm (Figure 9), as both **phen3** and **bpy3** spectra are blue-shifted compared to the **ppy3** and **bzq3** spectra from the increased Ir**–**N contacts. Reduction potentials are largely unaffected by increased rigidity (Table S11).

**Figure 9:**
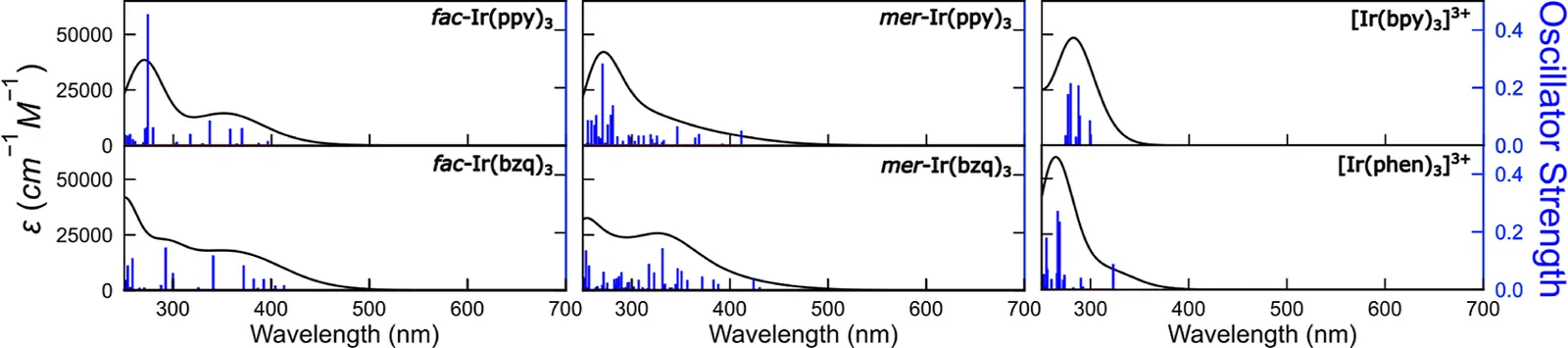
Rigidity and conjugation effects on GS singlet TD-DFT transitions, calculated at the PBE0-GD3/LANL2DZ­[Ir]+6–311G­(d,p)­[C,H,N]/PCM­(MeCN) level of theory (blue stokes) with the corresponding Gaussian-broadened absorbance spectrum (black) of the parent (top row) and rigid complexes (bottom row).

Substituting ppy and bpy for bzq and phen ligands has no quantifiable effect on the SOMO density of the *T*_1*a*_ state (Figure 2). Consistent across all complexes, the SOMO density of the ^3^MLCT state, or ^3^LC for **bpy3** and **phen3**, lies on one ligand. Further, increasing rigidity and conjugation has no significant impact on the energy of the *T*_1*a*_ minimum relative to the GS (Table 1). The ^3^MLCT of **ppy3** complexes is 2.60–2.70 eV, and that of **bzq3** complexes is 2.51–2.70 eV. Similarly the ^3^LC values of **bpy3** and **phen3** are 2.83 and 2.80 eV, respectively, nearly isoenergetic. Reducing the degree of possible ligand torsion reduces the O of the ^3^MC (Table 1). Since the bzq and phen ligands are unable to twist as much as ppy and bpy ligands, they instead push outward from the metal center (Table 9) to accommodate the increase metal density.

**Table 9:** Rigidity and Conjugation Effects on Ligand Torsion and Ir**–**L Distances in ^3^MC Complexes, Calculated at the PBE0-GD3/LANL2DZ­[Ir]+6-311G­(d,p)­[C,H,N]/PCM­(MeCN) Level of Theory

complex	$p_{l}$ (°)^a^	$r_{l}$ (Å)^b^	Ir**–**L_stay_ (Å)	Ir**–**L_twist_ (Å)
*fac* **-ppy3**	43.39	2.64	2.06	3.21
*fac* **-bzq3** ^c^	0.51	2.20	2.11	2.30
*mer* **-ppy3**	18.66	2.28	2.02	2.55
*mer* **-bzq3**	1.43	2.40	2.07	2.74
**bpy3**	13.16	2.24	2.08	2.39
**phen3**	2.29	2.29	2.15	2.43
	3.75	2.16	2.07	2.25

a Ligand torsion through bidentate dihedral.

b Average Ir**–**L distance.

c Lowest-energy SCF structure of ^3^MC character, see the Methods.

Rigidity decreases ΔO between the ^3^MLCT and ^3^MC minima (Table 10). A slight decrease in $E_{a\left(T_{1}\right)}$ from that of the parent complex is observed for **phen3** and *mer* **-bzq3** (Table 10). The shallower curvature of the triplet surfaces and the low $E_{a\left(T_{1}\right)}$ (Figure 10) suggest that increasing rigidity and conjugation may decrease the lifetime of the lowest-energy triplet state, weakening the photocatalytic performance of more rigid and more conjugated bidentate complexes, indicating that it is not worth experimental effort to synthesize these homoleptic complexes. While homoleptic tris-cyclometalated complexes of such rigidity (e.g., *fac* **-bzq3** and **phen3**) do not have reported lifetimes, exchanging the two ppy ligands on *m* **-ppy2** for bzq ligands gives an experimental luminescent lifetime of 0.26 μs^67^ rather than 0.275 μs of the parent complex,^62^ suggesting that ligand rigidity does not increase the photocatalytic lifetime.

**Table 10:** Rigidity and Conjugation Effects on Structural and Energetic Parameters along the Triplet Manifold That Correlate with Prolonged ^3^MLCT Lifetime, Calculated at the PBE0-GD3/LANL2DZ­[Ir]+6-311G­(d,p)­[C,H,N]/PCM­(MeCN) Level of Theory

complex	$\Delta O_{T_{1}}$ (°)^a^	ΔG_*T*_1__ (eV)^a^	min $E_{a\left(T_{1}\right)}$ (eV)^b^
*fac* **-ppy3**	8.14	0.55	((2))^c^
*fac* **-bzq3** ^e^	1.12	1.48	**—** ^d^
*mer* **-ppy3**	3.43	0.62	0.77
*mer* **-bzq3**	4.77	0.36	0.73
**bpy3**	4.74	0.51	0.95
**phen3**	2.52	0.59	0.75

a Between optimized triplet minima, ^3^MC–^3^MLCT or ^3^MC–^3^LC for **bpy3** and **phen3**.

b Difference between the higher-energy switch neighbor and lowest-energy optimized triplet.

c Extrapolated fit, see the Methods.

d Differences measured between ^3^MLCT and the lowest-energy SCF structure of ^3^MC character, see the Methods.

e No crossing was found; SCF-^3^MC nested within the ^3^MLCT domain.

**Figure 10:**
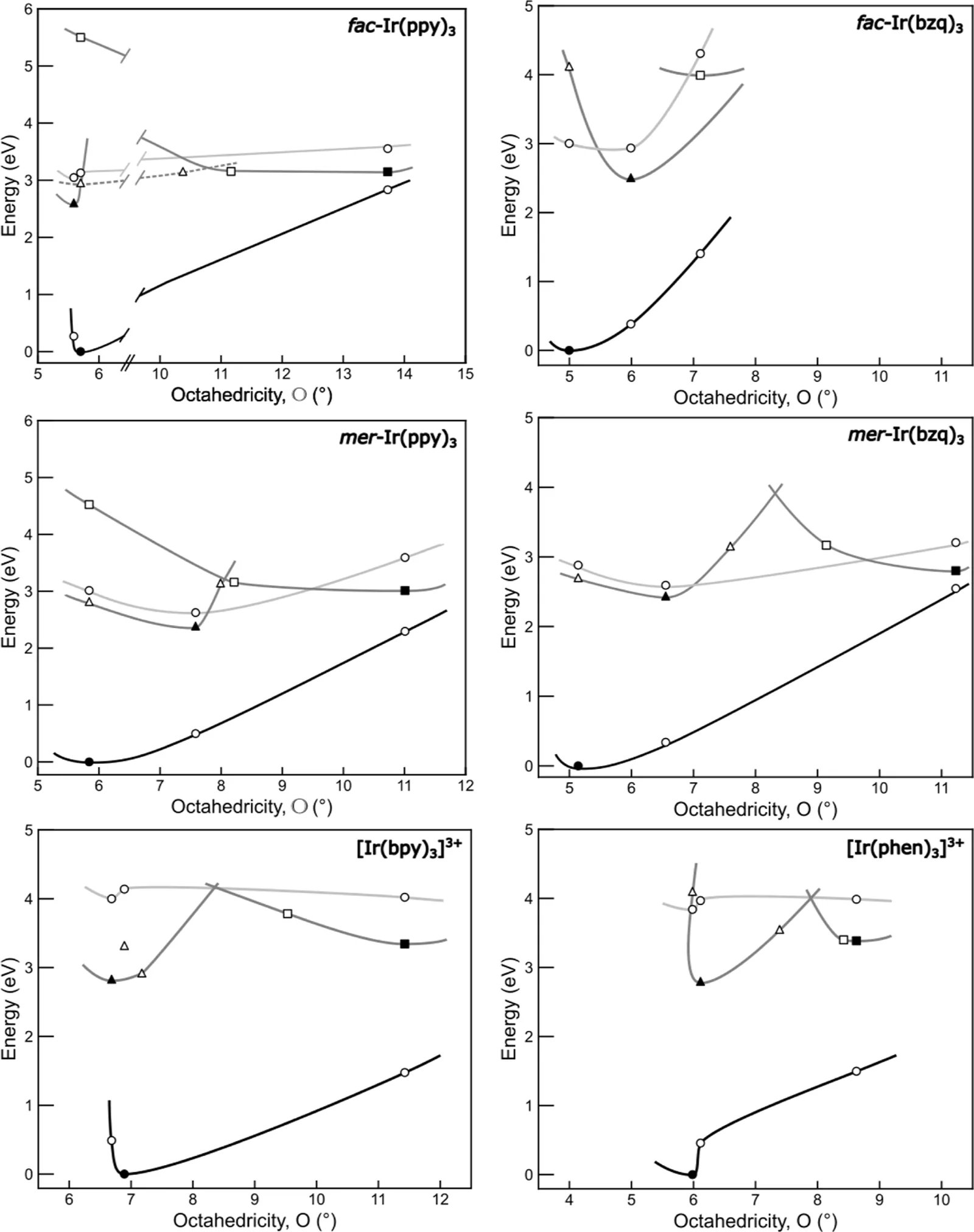
Rigidity and conjugation effects on PEC projected on O . Circles are singlet species, triangles are triplets with CT character, stars are triplets with LC character, and squares are triplets with MC character. Filled markers are freely optimized minima, while open-marker points are the SCF or TD-DFT excitation energies of that multiplicity. Other PEC projections are provided in the Supporting Information (Figures S69 –S78).

## 4 Conclusions

A systematic comparison of the structurally quantitative photophysical DFT surfaces of six archetypal polypyridyl Ir­(III) complexes and their isomers provides insights into the effects of (1) increasing the number of Ir–N contacts, (2) through-metal push–pull axes, and (3) increasing ligand rigidity on the lifetime of the catalytically active triplet. Geometric distortion of the ground and triplet states are primarily driven by torsion of the ligand dihedral backbone. Ligand twisting inflates the structural deviation from pure O_*h*_ symmetry beyond what is typically seen in bidentate metal complexes. For all Ir­(III) complexes, the metal-centered triplets were higher energy than the catalytic MLCT or LC triplets. This consistently lower-lying ^3^MLCT or ^3^LC state rationalizes the wide tunability of Ir­(III) complexes without loss of photoactivity, in contrast to Ru­(II)^13^ and Fe­(II)^20^ photosensitizers where low-lying ^3^MCs are often an issue.

Substituting ppy ligands for bpy increases the number of Ir**–**N contacts on the complex. This leads to greater distortion in GS and ^3^MLCT states, but decreased $\Delta O_{T_{1}}$ and, in turn, decreased $E_{a\left(T_{1}\right)}$ . Lowering the activation barrier of populating the deactivating ^3^MC well suggests faster loss of the catalytically active ^3^MLCT with increasing bpy ligands. However, substituting only one ppy with a bpy ligand to yield ***m*-** and *f*-**ppy2** with four Ir**–**N contacts resulted in localization of electron density onto only the bpy ligand in both the GS LUMO and the ^3^MLCT SOMO, suggesting facile and rapid intersystem crossing that is supported by spin–orbit coupling calculations between these states. These double-quantitative PECs, therefore, provide direct quantum mechanical evidence for the photocatalytic performance of **ppy2** complexes relative to that of the *fac* **-ppy3** or *mer* **-ppy3** parent complexes. The growing popularity of these mixed ligand catalysts^5,7,9,54,102^ is often ascribed to their synthetic ease, and these quantitative PECs show how the excited state reactivity is maintained through balancing push–pull axes and structural flexibility, despite loss of symmetry.

Altering the relationship of the donating Ir**–**C contacts and accepting Ir**–**N contacts impacts the through-metal coordination in a manner that merely changing the number of contacts does not. Decreasing the number of through-metal C**–**Ir**–**N axes with different orientation of ligands, e.g., *fac*- and *mer*- isomers, resulted in less distorted and lower-energy ^3^MC states. Thus, the less thermodynamically preferred isomer that has fewer stabilizing trans C**–**Ir**–**N axes (*mer* **-ppy3**, *f* **-ppy2**, and *mer* **-bzq3**) may more easily populate the deactivating ^3^MC state. Ultimately, these excited state surfaces suggest loss of photoredox activity with loss of trans push–pull electronic donation.

Increasing the rigidity of homoleptic complexes with fused aromatic rings by substituting ppy and bpy ligands for bzq and phen ligands resulted in less distorted ^3^MC structures, with ligands pushing outward from the metal rather than twisting. Less distortion in the ^3^MC brought the competing triplet wells structurally closer to the catalytically active *T*_1*a*_ state, leading to a marginal decrease in the activation energy, suggesting facile deactivation of the catalyst by populating the ^3^MC in these homoleptic complexes.

This energetically and structurally quantitative systematic study provides a quantum mechanical basis that links published experimental works of many polypyridyl Ir­(III) photosensitizes by expanding on the existing theoretical works^37,54–60^ and underrepresented complexes in this family.^37,60,105,106^ The data herein highlights design principles that control the curvature and crossing points between competing triplet domains that lead to prolonged excited state lifetimes for photoactivity. Of the archetypes studied here, the *m* **-ppy2** parent complex has the best balance and alignment of push–pull axes, leading to the slowest deactivation through the metal-centered state. New complexes that maintain coplanar C–Ir–N push–pull axes with other ligand motifs and chelation should be explored.

## Supplementary Material



## Notes

Structures, structural parameters, and molecular orbital diagrams of GS, ^3^MLCT, and ^3^MC points of all complexes; structure–structure potential energy surfaces of search data to find the ^3^MC of all complexes; reduction potentials of ground- and excited-state reduction and oxidation single electron transfers; spin density difference orbitals of ^3^MLCT and ^3^MC states of all complexes; calculated absorbance spectra, TD-DFT tables, and NTO pairings of all complexes; spin–orbit coupling tables of the first 10 singlet and triplet excited states of all complexes; S_1_/S_0_ to T_1*a*_ reorganization energy of all complexes; constrained optimization scans along the triplet T_1*a*_ to ^3^MC vector, projected along all structural axes and structure–structure potential energy surfaces; structure–structure potential energy surfaces of stationary points of all complexes; and PECs of all complexes, projected along all structural axes (PDF)
