# Supplementary material for: Systematic Understanding of Freely Optimized Excited-State Evolution in Ir(III) Photocatalysts
Source: ACS Omega. 2026 Aug 29;11(36):54121–38. doi: 10.1021/acsomega.6c04785 (PMC13584167; doi:10.1021/acsomega.6c04785)
Supplement: Supplementary file 1 [file ao6c04785_si_001.pdf]

Supporting Information

Systematic Understanding of Freely-Optimized Excited-State Evolution in Ir(III) Photocatalysts

Cristabella R. Fortna and Lisa A. Fredin*

Department of Chemistry, Lehigh University, Bethlehem, PA 18015, USA

E-mail: lafredin@lehigh.edu

Contents

Structures and Structural Parameters **S7**

Table S1: Important electronic and structural parameters of all complexes S7

Figure S1: Structures of ***fac*-ppy3** S8

Table S2: Structural parameters of optimized stationary points for ***fac*-ppy3** . . . S8

Figure S2: Structure-structure search data for ***fac*-ppy3** ^{3}MC S8

Figure S3: Structures of ***mer*-ppy3** S10

Table S3: Structural parameters of optimized stationary points for ***mer*-ppy3** . . S10

Figure S4: Structure-structure search data for ***mer*-ppy3** ^{3}MC S10

Figure S5: Structures of ***m*-ppy2** S12

Table S4: Structural parameters of optimized stationary points for ***m*-ppy2** . . . S13

Figure S6: Structure-structure search data for ***m*-ppy2** ^{3}MC S14

Figure S7: Structures of ***f*-ppy2** S16

Table S5: Structural parameters of optimized stationary points for ***f*-ppy2** S16

Figure S8: Structure-structure search data for ***f*-ppy2** ^{3}MC S16

Figure S9: Structures of **bpy2** S18

Table S6: Structural parameters of optimized stationary points for **bpy2** S19

Figure S10: Structure-structure search data for **bpy2** ^{3}MC S19

Figure S11: Structures of **bpy3** S20

Table S7: Structural parameters of optimized stationary points for **bpy3** S21

Figure S12: Structure-structure search data for **bpy3** ^{3}MC S21

Figure S13: Search for optimized ***fac*-bzq3** ^{3}MC S22

Figure S14: Structures of ***fac*-bzq3** S24

Table S8: Structural parameters of optimized stationary points for ***fac*-bzq3** . . . S25

Figure S15: Structure-structure search data for ***fac*-bzq3** ^{3}MC S25

Figures S16: Structures of ***mer*-bzq3** S26

Table S9: Structural parameters of optimized stationary points for ***mer*-bzq3** . . S27

Figure S17: Structure-structure search data for ***mer*-bzq3** ^{3}MC S27

Figure S18: Structures of **phen3** S28

Table S10: Structural parameters of optimized stationary points for **phen3** S29

Figure S19: Structure-structure search data for **phen3** ^{3}MC S29

Figure S20: C_3 symmetrized **phen3** SCF structure S30

Electronic Structure **S31**

Table S11: Ground- and excited-state reduction potentials S31

Figure S21: Frontier orbitals of each GS complex with HOMO-LUMO gap S32

Figure S22: MO diagrams of ***fac*-ppy3** optimized minima S33

Figure S23: MO diagrams of ***mer*-ppy3** optimized minima S33

Figure S24: MO diagrams of ***m*-ppy2** optimized minima S33

Figure S25: MO diagrams of ***f*-ppy2** optimized minima S33

Figure S26: MO diagrams of **bpy2** optimized minima S33

Figure S27: MO diagrams of **bpy3** optimized minima S33

Figure S28: MO diagrams of ***fac*-bzq3** optimized minima S33

Figure S29: MO diagrams of ***mer*-bzq3** optimized minima S33

Figure S30: MO diagrams of **phen3** optimized minima S33

Figure S31: Spin densities of ***fac*-ppy3** triplet structures S42

Figure S32: Spin densities of ***mer*-ppy3** triplet structures S42

Figure S33: Spin densities of ***m*-ppy2** triplet structures S42

Figure S34: Spin densities of ***f*-ppy2** triplet structures S42

Figure S35: Spin densities of **bpy2** triplet structures S42

Figure S36: Spin densities of **bpy3** triplet structures S42

Figure S37: Spin densities of ***fac*-bzq3** triplet structures S42

Figure S38: Spin densities of ***mer*-bzq3** triplet structures S42

Figure S39: Spin densities of **phen3** triplet structures S42

TD-DFT, SOC Matrices, and Reorganization Energies **S47**

Figure S40: Calculated absorbance spectra for all complexes S47

Figure S41: First 10 ***fac*-ppy3** singlet NTOs S49

Table S12: ***fac*-ppy3** TD-DFT table S50

Table S13: Spin-orbit coupling of the first 10 excited states of ***fac*-ppy3** S51

Figure S42: First 10 ***mer*-ppy3** singlet NTOs S52

Table S14: ***mer*-ppy3** TD-DFT table S53

Table S15: Spin-orbit coupling of the first 10 excited states of ***mer*-ppy3**. S54

Figure S43: First 10 ***m*-ppy2** singlet NTOs S55

Table S16: ***m*-ppy2** TD-DFT table S56

Table S17: Spin-orbit coupling of the first 10 excited states of ***m*-ppy2**. S57

Figure S44: First 10 ***f*-ppy2** singlet NTOs S58

Table S18: ***f*-ppy2** TD-DFT table S59

Table S19: Spin-orbit coupling of the first 10 excited states of ***f*-ppy2** S60

Figure S45: First 10 **bpy2** singlet NTOs S61

Table S20: **bpy2** TD-DFT table S62

Table S21: Spin-orbit coupling of the first 10 excited states of **bpy2** S63

Figure S46: First 10 **bpy3** singlet NTOs S65

Table S22: **bpy3** TD-DFT table S65

Table S23: Spin-orbit coupling of the first 10 excited states of **bpy3** S67

Figure S47: First 10 ***fac*-bzq3** singlet NTOs S68

Table S24: ***fac*-bzq3** TD-DFT table S69

Table S25: Spin-orbit coupling of the first 10 excited states of ***fac*-bzq3** S70

Figure S48: First 10 ***mer*-bzq3** singlet NTOs S71

Table S26: ***mer*-bzq3** TD-DFT table S72

Table S27: Spin-orbit coupling of the first 10 excited states of ***mer*-bzq3** S73

Figure S49: First 10 **phen3** singlet NTOs S74

Table S28: **phen3** TD-DFT table S75

Table S29: Spin-orbit coupling of the first 10 excited states of ***mer*-bzq3** S77

Table S30: Reorganization energies (λ) of populating the T_{1a} minimum S78

Triplet-to-triplet scans **S79**

Figure S50: Constrained optimizations along ***fac*-ppy3** triplet manifold S79

Figure S51: Structure-structure projected ***fac*-ppy3** triplet scan S80

Figure S52: Constrained optimizations along ***mer*-ppy3** triplet manifold S81

Figure S53: Structure-structure projected ***mer*-ppy3** triplet scan S82

Figure S54: Constrained optimizations along ***m*-ppy2** triplet manifold S83

Figure S55: Structure-structure projected ***m*-ppy2** triplet scan S84

Figure S56: Constrained optimizations along ***f*-ppy2** triplet manifold S85

Figure S57: Structure-structure projected ***f*-ppy2** triplet scan S86

Figure S58: Constrained optimizations along **bpy2** triplet manifold S87

Figure S59: Structure-structure projected **bpy2** triplet scan S88

Figure S60: Constrained optimizations along **bpy3** triplet manifold S89

Figure S61: Structure-structure projected **bpy3** triplet scan S90

Figure S62: Constrained optimizations along ***fac*-bzq3** triplet manifold S91

Figure S63: Structure-structure projected ***fac*-bzq3** triplet scan S92

Figure S64: Constrained optimizations along ***mer*-bzq3** triplet manifold S93

Figure S65: Structure-structure projected ***mer*-bzq3** triplet scan S94

Figure S66: Constrained optimizations along **phen3** triplet manifold S95

Figure S67: Structure-structure projected **phen3** triplet scan S96

Potential Energy Curves **S97**

Table S31: Structural and energetic parameters along the triplet manifold of all complexes S97

Table S32: Minimum-energy crossing points reported relative to the ^{3}MC S98

Figure S68: Expanded potential energy curves of all complexes S98

Figure S69: Potential energy curves of ***fac*-ppy3** S110

Figure S70: Potential energy curves of ***mer*-ppy3** S110

Figure S71: Potential energy curves of ***m*-ppy2** S110

Figure S72: Potential energy curves of ***f*-ppy2** S110

Figure S73: Potential energy curves of **bpy2** S110

Figure S74: Potential energy curves of **bpy3** S110

Figure S75: Potential energy curves of ***fac*-bzq3** S110

Figure S76: Potential energy curves of ***fac*-bzq3** with other search points S110

Figure S77: Potential energy curves of ***mer*-bzq3** S110

Figure S78: Potential energy curves of **phen3** S110

Structure-structure projected Surfaces **S110**

Figure S79: Twin-axis potential energy surfaces of ***fac*-ppy3** S110

Figure S80: Twin-axis potential energy surfaces of ***mer*-ppy3** S110

Figure S81: Twin-axis potential energy surfaces of ***m*-ppy2** S110

Figure S82: Twin-axis potential energy surfaces of ***f*-ppy2** S110

Figure S83: Twin-axis potential energy surfaces of **bpy2** S110

Figure S84: Twin-axis potential energy surfaces of **bpy3** S110

Figure S85: Twin-axis potential energy surfaces of ***fac*-bzq3** S110

Figure S86: Twin-axis potential energy surfaces of ***mer*-bzq3** S110

Figure S87: Twin-axis potential energy surfaces of **phen3** S110

Structures and Structural Parameters

Table S1: Summary of important electronic and structural parameters of freely-optimized minima calculated at the PBE0-D3/LANL2DZ[Ir]+6-311G(d,p)[C,H,N]/PCM(MeCN) level of theory.

Complex	State	E[a] (eV)	$\mathbb{O}^b$ (°)	$\mathbb{P}^c$ (°)	$\mathbb{R}^d$ (Å)	Spin on Ir[e]
***fac*-ppy3**	GS	0.00	5.70	0.66 ± 0.06	2.07 ± 0.03	—
	^{3}MLCT	2.60	5.59	0.83 ± 0.51	2.07 ± 0.03	0.40 (0.39)
	^{3}MC	3.15	13.73	14.55 ± 24.98	2.25 ± 0.33	1.08 (1.12)
***mer*-ppy3**	GS	0.31	5.84	0.95 ± 0.54	2.07 ± 0.02	—
	^{3}MLCT	2.70	7.58	2.06 ± 1.32	2.08 ± 0.04	0.42 (0.45)
	^{3}MC	3.32	11.01	7.50 ± 9.82	2.26 ± 0.18	1.38 (1.36)
***m*-ppy2**	GS	0.00	6.53	1.28 ± 0.81	2.07 ± 0.02	—
	^{3}MLCT	2.35	6.38	0.60 ± 0.23	2.06 ± 0.03	0.54 (0.53)
	^{3}MC	3.14	12.61	3.06 ± 1.87	2.20 ± 0.19	1.53 (1.49)
***f*-ppy2**	GS	0.06	6.31	1.00 ± 0.47	2.07 ± 0.02	—
	^{3}MLCT	2.35	6.98	1.65 ± 1.39	2.06 ± 0.03	0.57 (0.55)
	^{3}MC	3.09	8.71	10.01 ± 7.25	2.18 ± 0.06	1.39 (1.35)
**bpy2**	GS	0.00	6.70	1.25 ± 0.54	2.06 ± 0.02	—
	^{3}MLCT	2.66	6.57	2.32 ± 1.47	2.05 ± 0.03	0.45 (0.44)
	^{3}MC	2.91	8.85	11.03 ± 9.25	2.16 ± 0.06	1.34 (1.29)
**bpy3**	GS	0.00	6.89	1.77 ± 0.11	2.05 ± 0.00	—
	^{3}LC	2.83	6.69	1.58 ± 0.16	2.05 ± 0.00	0.03 (0.04)
	^{3}MC	3.34	11.43	6.70 ± 6.57	2.18 ± 0.06	1.66 (1.59)
***fac*-bzq3**	GS	0.00	5.00	0.39 ± 0.11	2.08 ± 0.03	—
	^{3}MLCT	2.51	5.99	0.52 ± 0.27	2.08 ± 0.03	0.57 (0.56)
	^{3}MC*	3.99	7.11	0.39 ± 0.11	2.16 ± 0.07	1.11 (1.06)
***mer*-bzq3**	GS	0.33	5.14	0.38 ± 0.23	2.07 ± 0.02	—
	^{3}MLCT	2.70	6.55	0.37 ± 0.40	2.08 ± 0.03	0.40 (0.42)
	^{3}MC	3.13	11.32	0.56 ± 0.76	2.21 ± 0.10	1.30 (1.31)
**phen3**	GS	0.00	5.98	0.98 ± 0.04	2.06 ± 0.00	—
	^{3}LC	2.80	6.11	0.92 ± 0.10	2.05 ± 0.00	0.00 (0.00)
	^{3}MC	3.39	8.63	2.37 ± 1.34	2.17 ± 0.06	1.54 (1.47)

[a]Relative to GS of lowest-energy isomer of that complex. [b]Average difference of L–Ir–L angles from 90°. [c]Average ligand torsion across bidentate dihedral. [d]Average of all Ir–L distances. [e]Mulliken spin populations reported with Löwdin spin populations in parentheses. *Lowest-energy SCF structure of ^{3}MC character, see Methods.

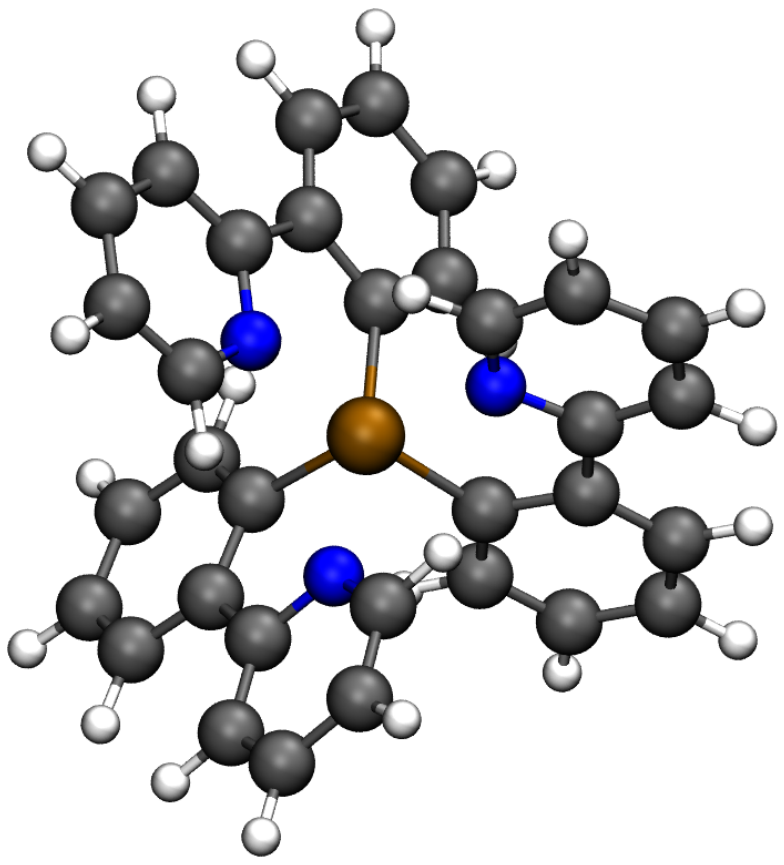

(a) Ground State

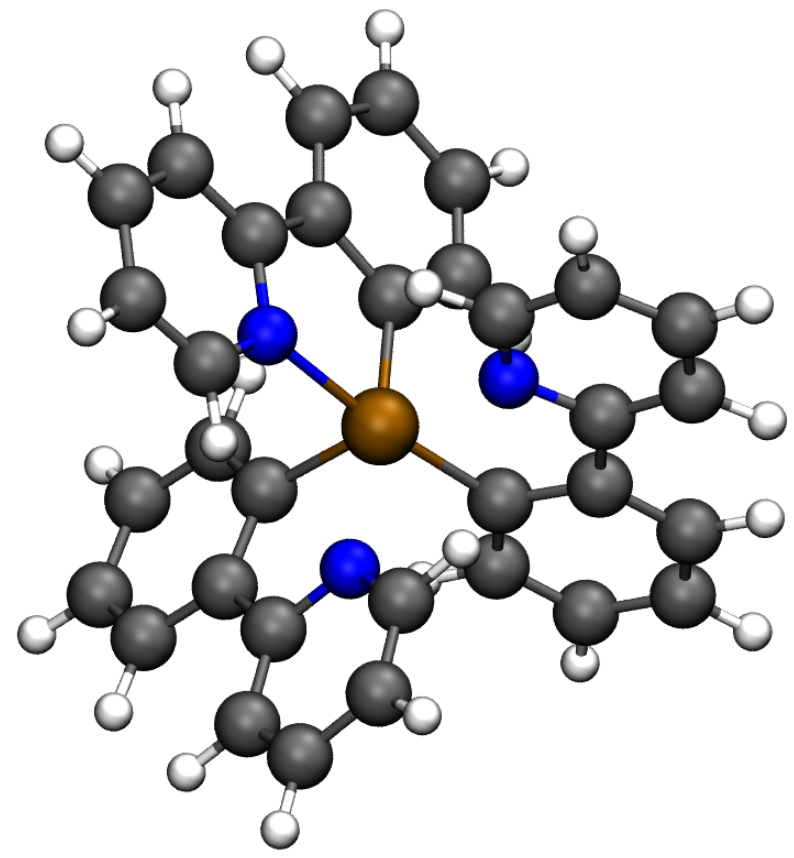

(b) ^{3}MLCT

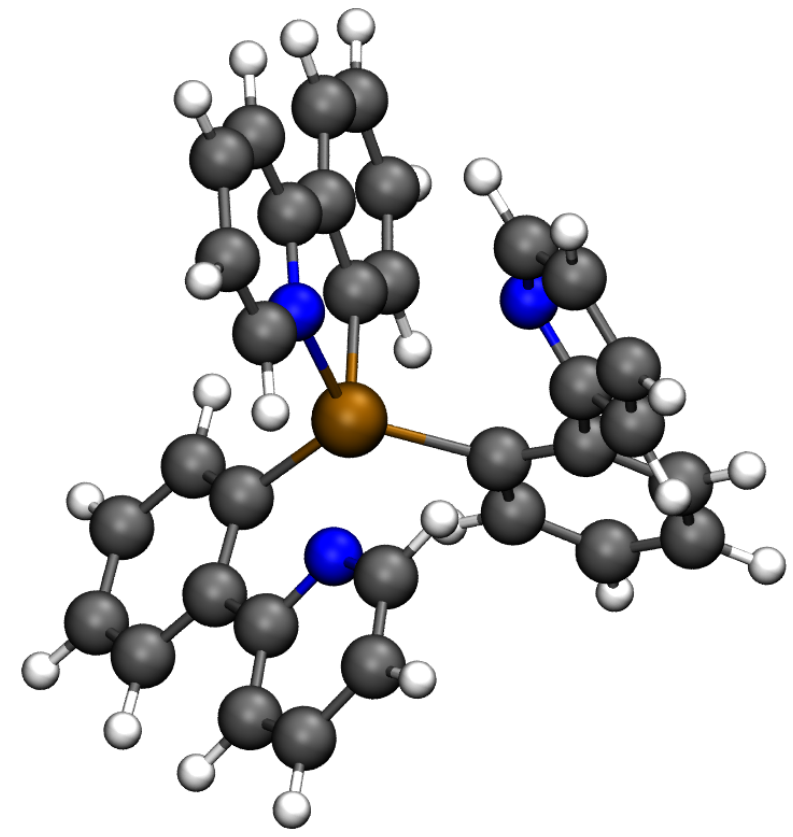

(c) ^{3}MC

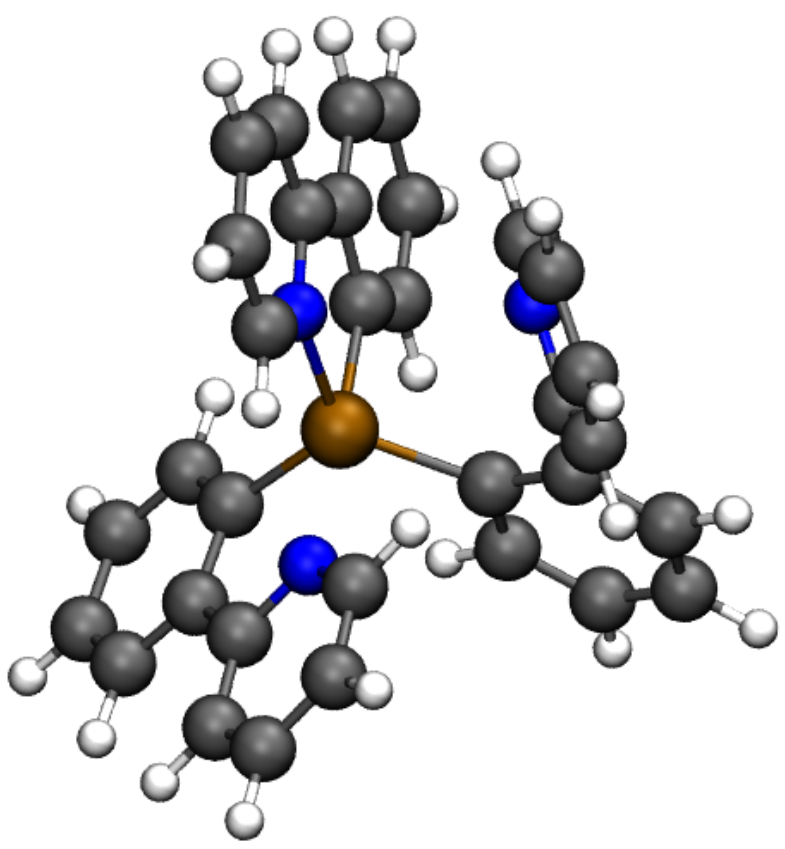

(d) MECP

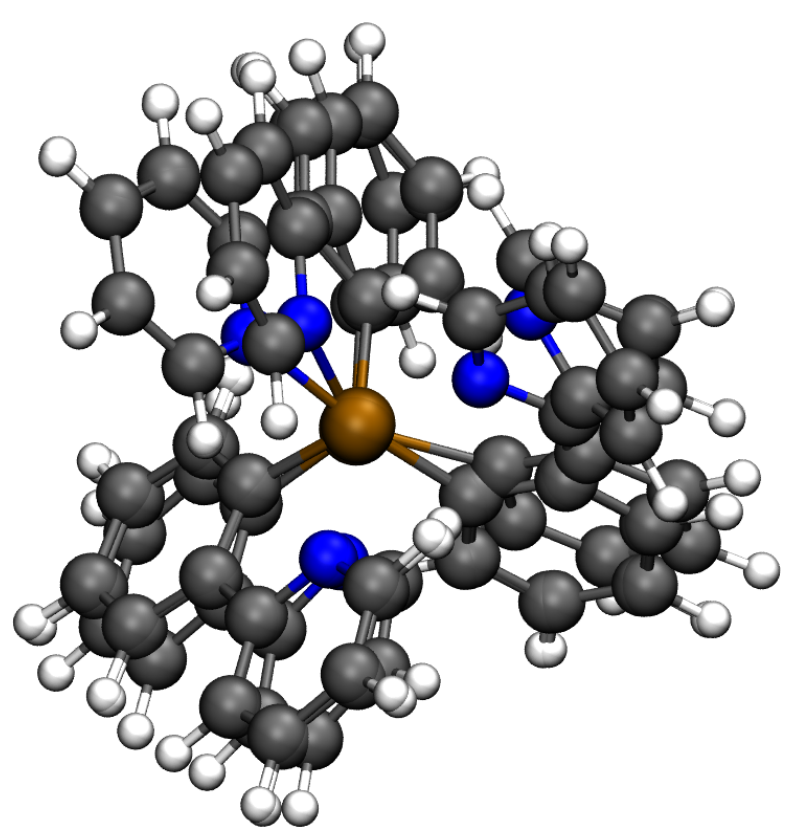

(e) GS/^{3}MLCT/^{3}MC

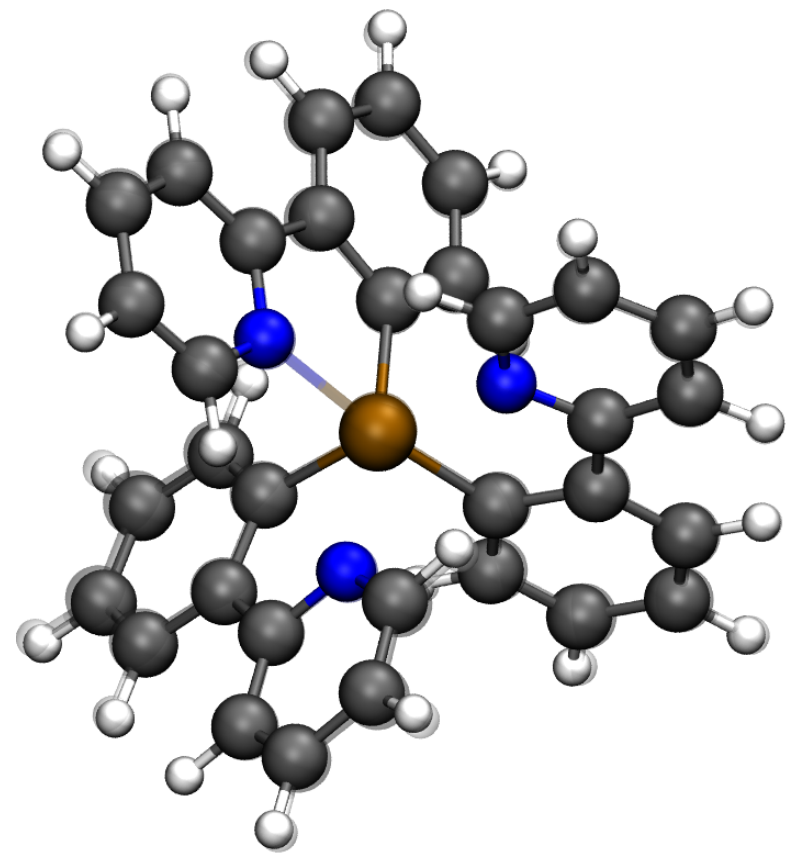

(f) GS/^{3}MLCT

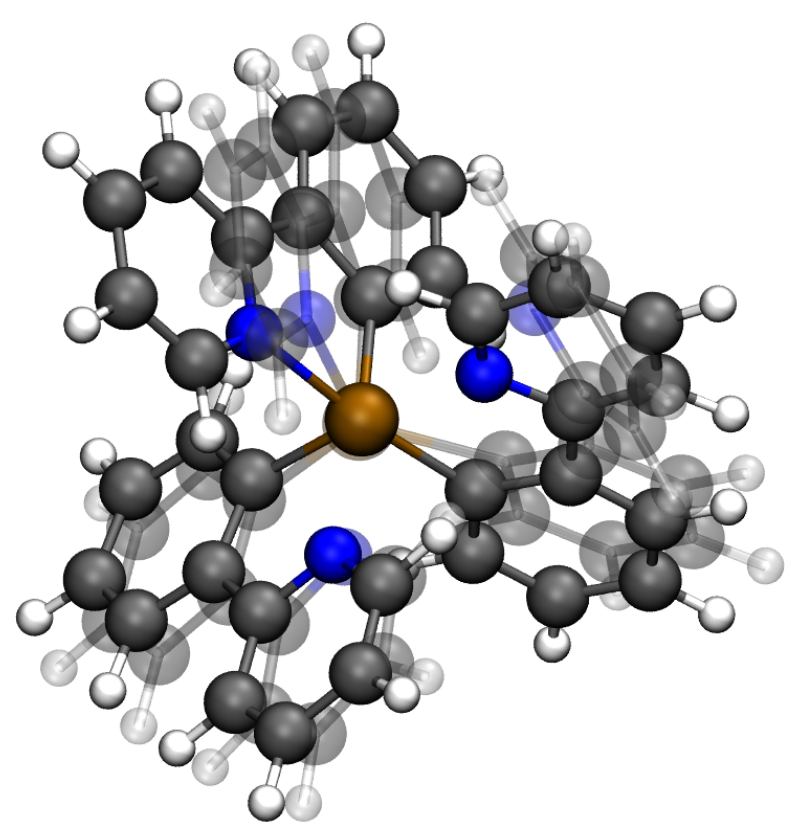

(g) ^{3}MLCT/^{3}MC

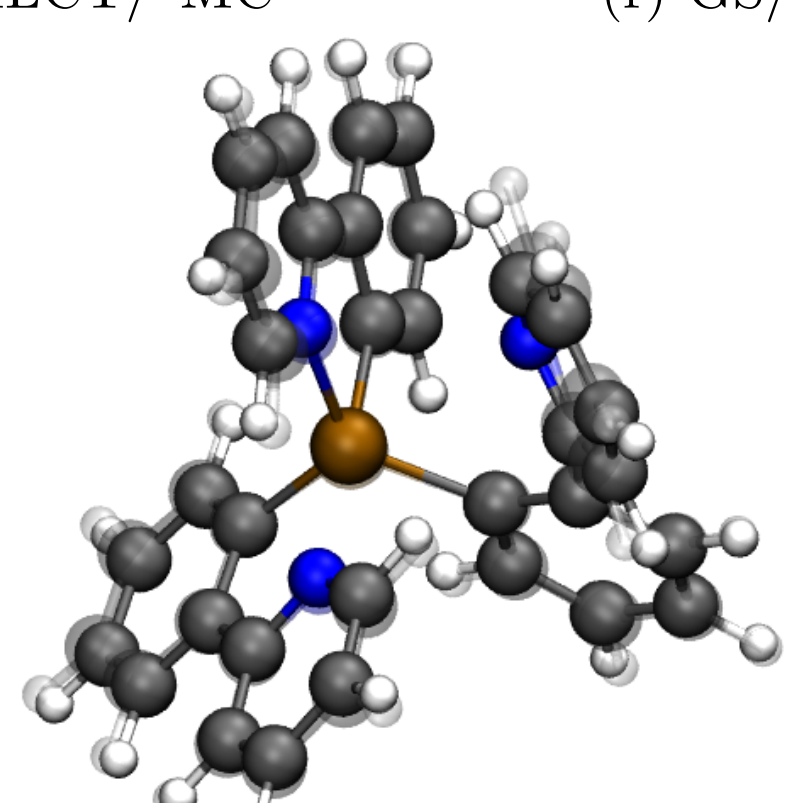

(h) ^{3}MC/MECP

Figure S1: Optimized structures (a−d) and structural overlays (e−h) of ***fac*-ppy3** calculated at the PBE0-D3/LANL2DZ[Ir]+6-311G(d,p)[C,H,N]/PCM(MeCN) level of theory.

Table S2: Structural parameters of optimized stationary points for ***fac*-ppy3** calculated at the PBE0-D3/LANL2DZ[Ir]+6-311G(d,p)[C,H,N]/PCM(MeCN) level of theory.

	Exp.[a]	GS	^{3}MLCT	^{3}MC	MECP
E (eV)	—	0.00	2.60	3.15	3.16
Spin[b] on Ir	—	—	0.40(0.39)	1.08(1.12)	—
Spin[b] on L1	—	—	0.40	1.08	—
Spin[b] on L2	—	—	0.00	0.31	—
Spin[b] on L3	—	—	0.30	0.35	—
r_1[c] (Å)	2.06	2.07	2.08	2.64	2.71
r_2[c] (Å)	2.06	2.07	2.04	2.04	2.04
r_3[c] (Å)	2.06	2.07	2.09	2.08	2.09
$\mathbb{R}$[d] (Å)	2.06 ± 0.03	2.07 ± 0.03	2.07 ± 0.03	2.25 ± 0.33	2.28 ± 0.38
p_1[e] (°)	0.20	0.65	0.31	43.39	51.58
p_2[e] (°)	0.20	0.61	1.34	0.17	0.11
p_3[e] (°)	0.20	0.72	0.83	0.10	0.18
$\mathbb{P}$[f] (°)	0.20 ± 0.00	0.66 ± 0.06	0.83 ± 0.51	14.55 ± 24.98	17.29
$\mathbb{O}$[g] (°)	5.68	5.70	5.59	13.73	15.16

[a]Measured parameters from *fac*-Ir(ppy)$_3$ crystal by Breu et al.[1] [b]Mulliken spin populations reported, with Löwdin spin populations in parentheses. [c]Average Ir–L distance of one ligand. [d]Average of all Ir–L contacts ± standard deviation. [e]Ligand torsion through bidentate dihedral angle. [f]Average ligand torsion ± standard deviation. [g]Average difference of L–Ir–L angles from 90°.

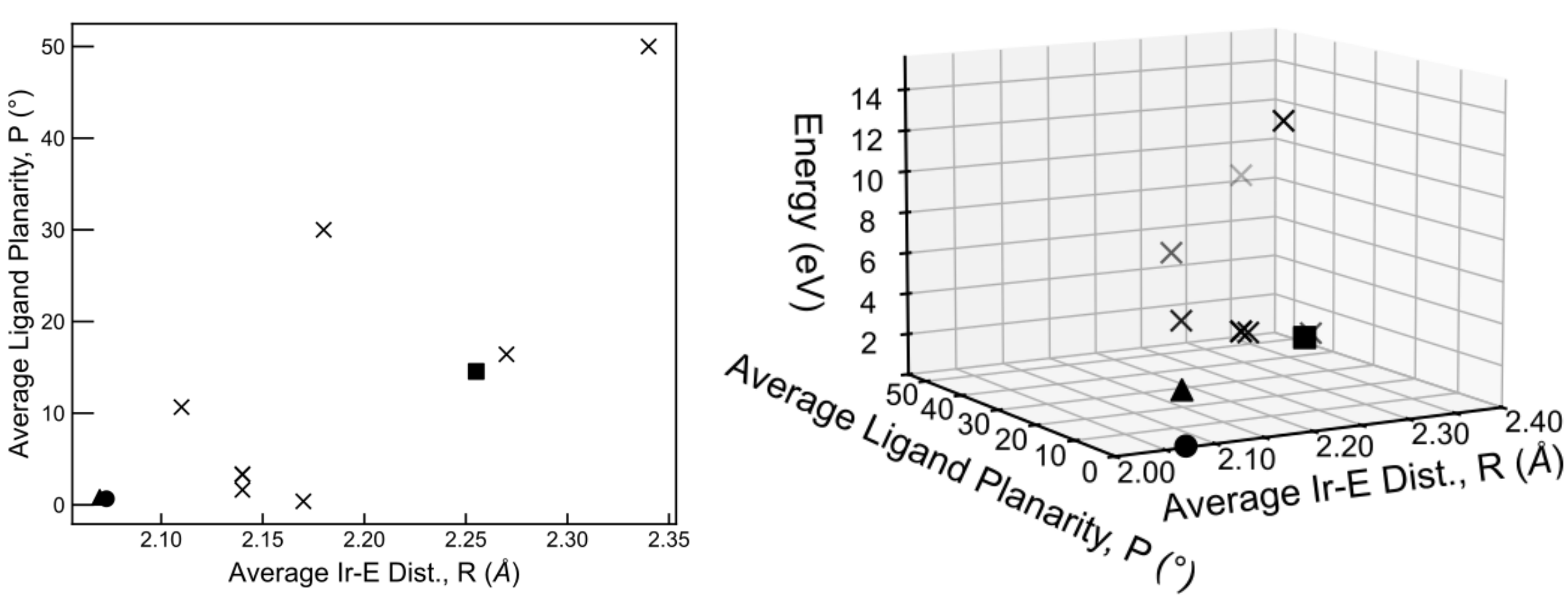

Figure S2: Structure-structure projected surfaces of guess structures surveyed in the search for a ***fac*-ppy3** ^{3}MC minimum. Freely-optimized GS, ^{3}MLCT, and ^{3}MC minima are indicated with a filled circle, triangle, and square respectively. Calculated at the PBE0-D3/LANL2DZ[Ir]+6-311G(d,p)[C,H,N]/PCM(MeCN) level of theory

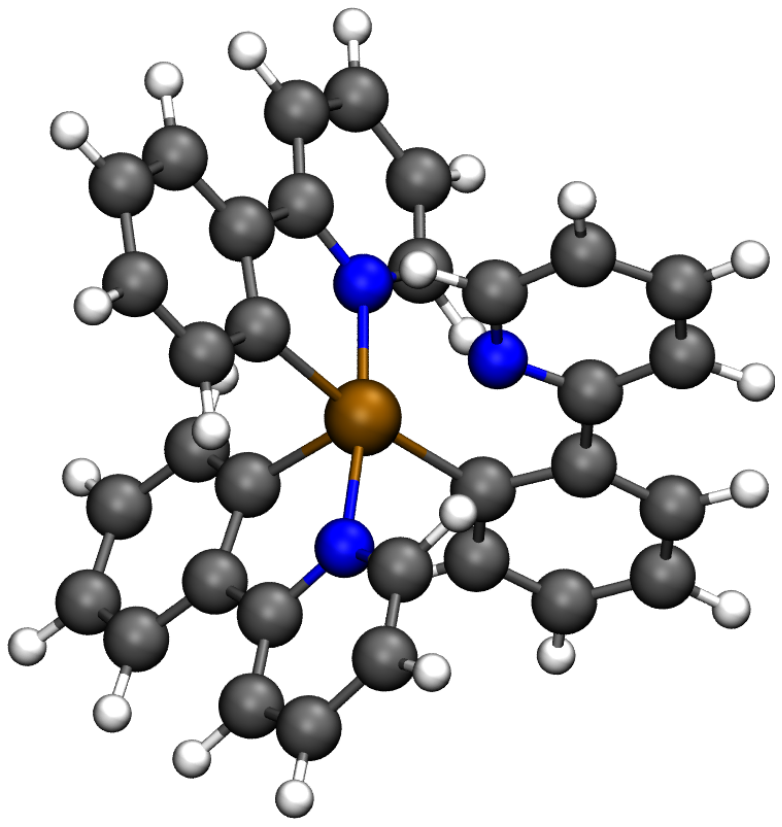

(a) Ground State

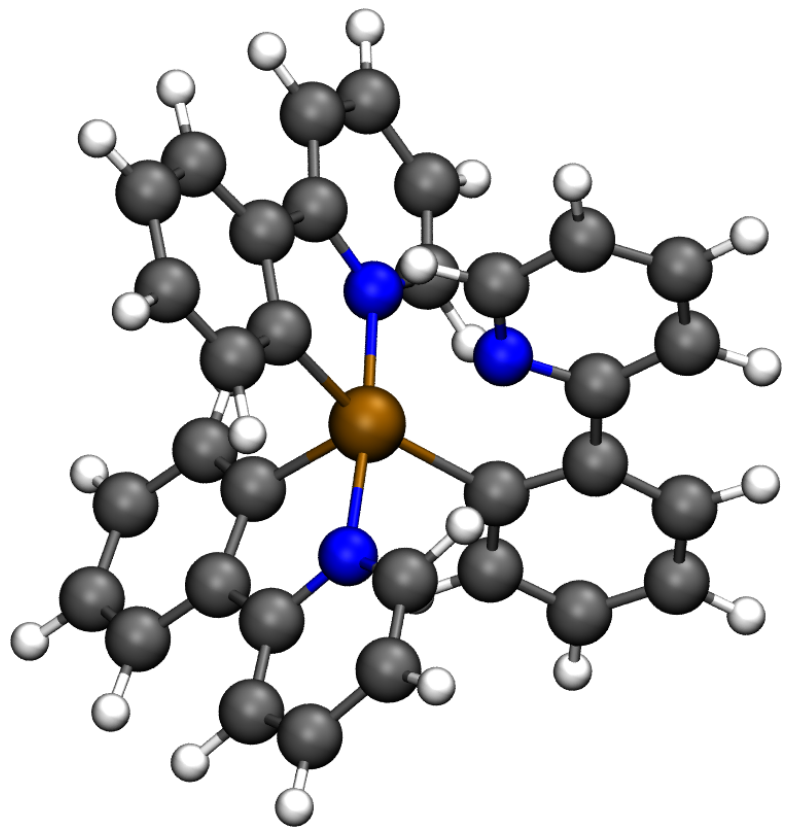

(b) ^{3}MLCT

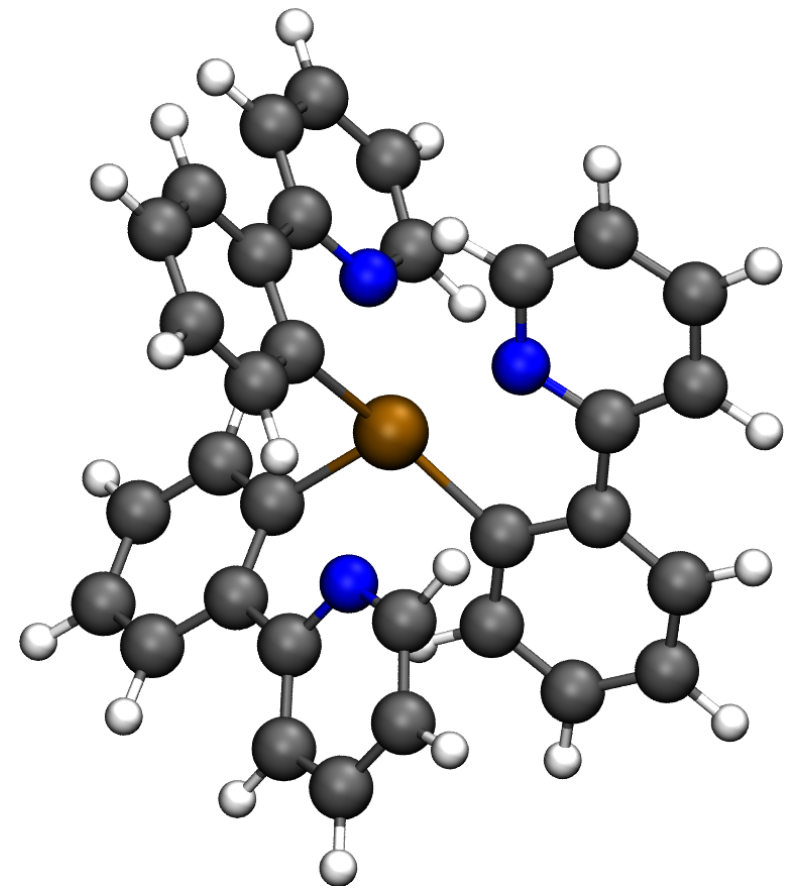

(c) ^{3}MC

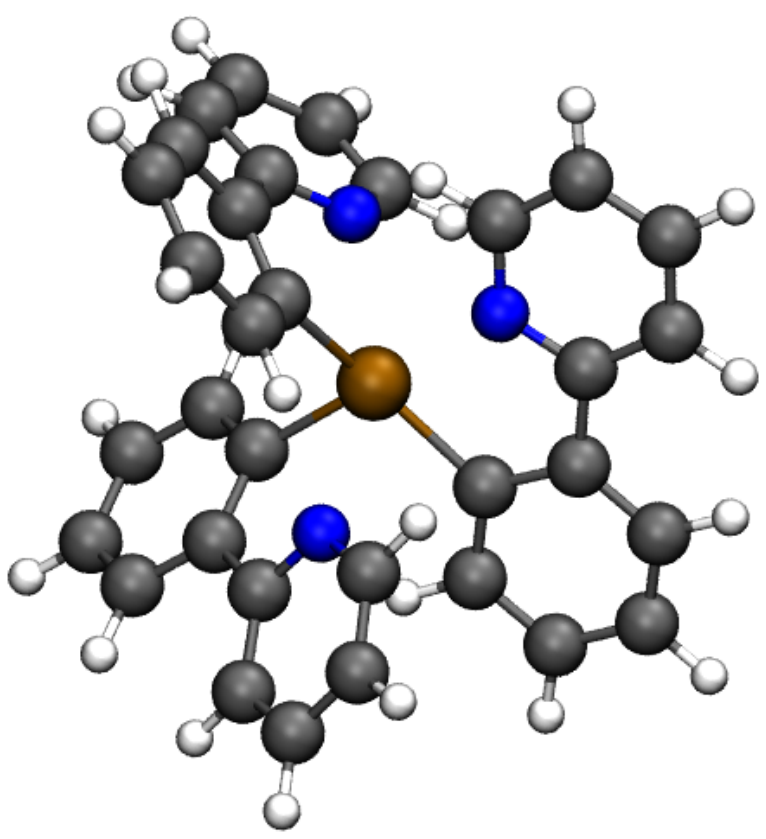

(d) MECP

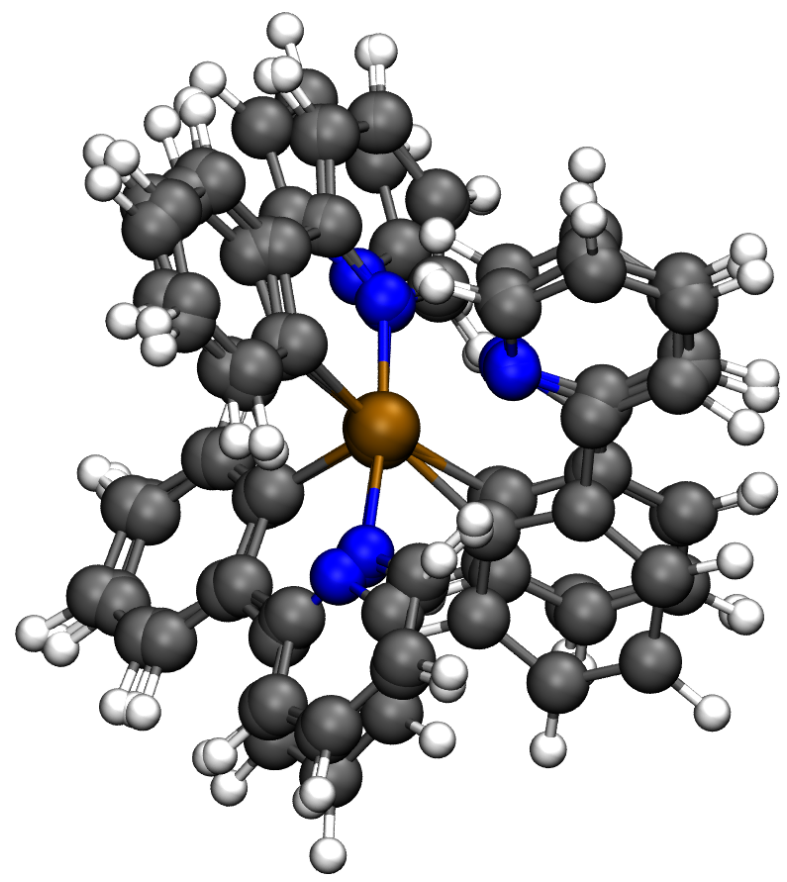

(e) GS/^{3}MLCT/^{3}MC

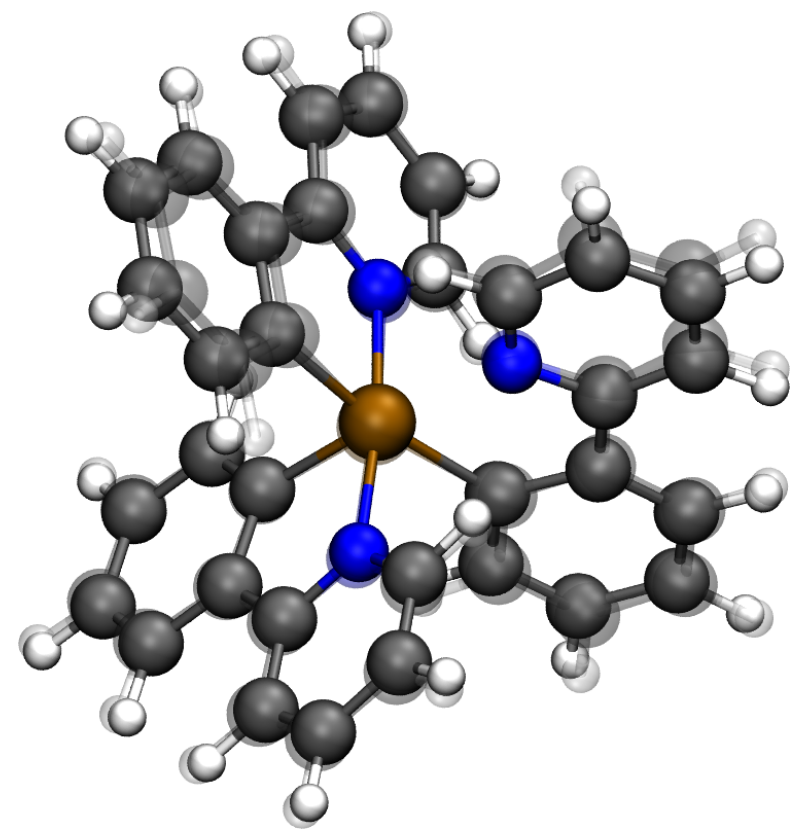

(f) GS/^{3}MLCT

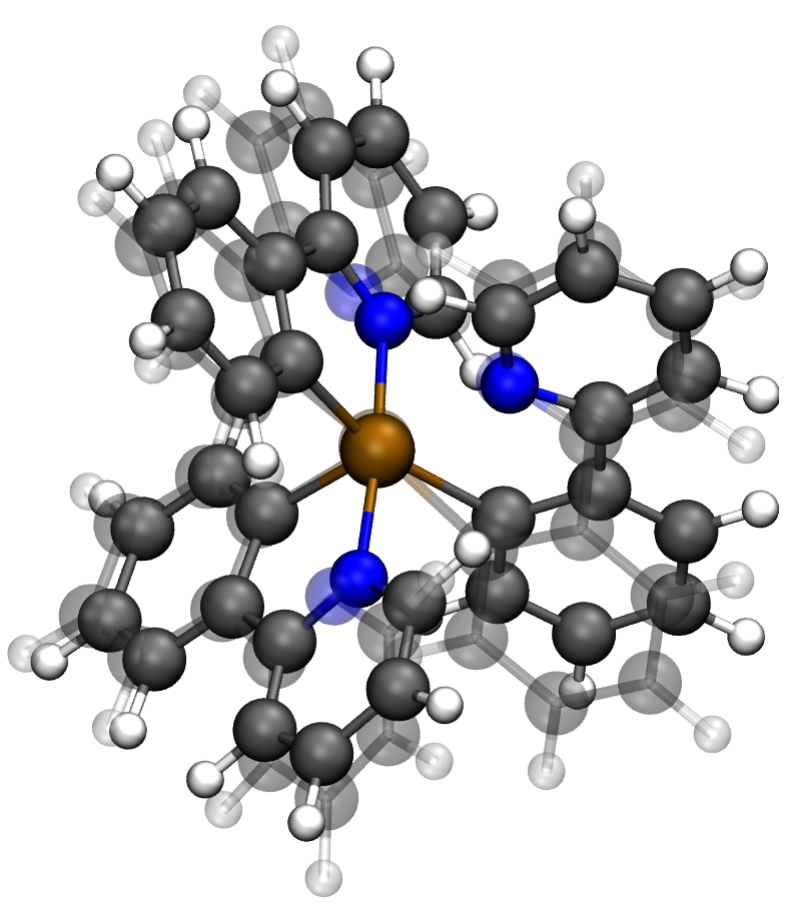

(g) ^{3}MLCT/^{3}MC

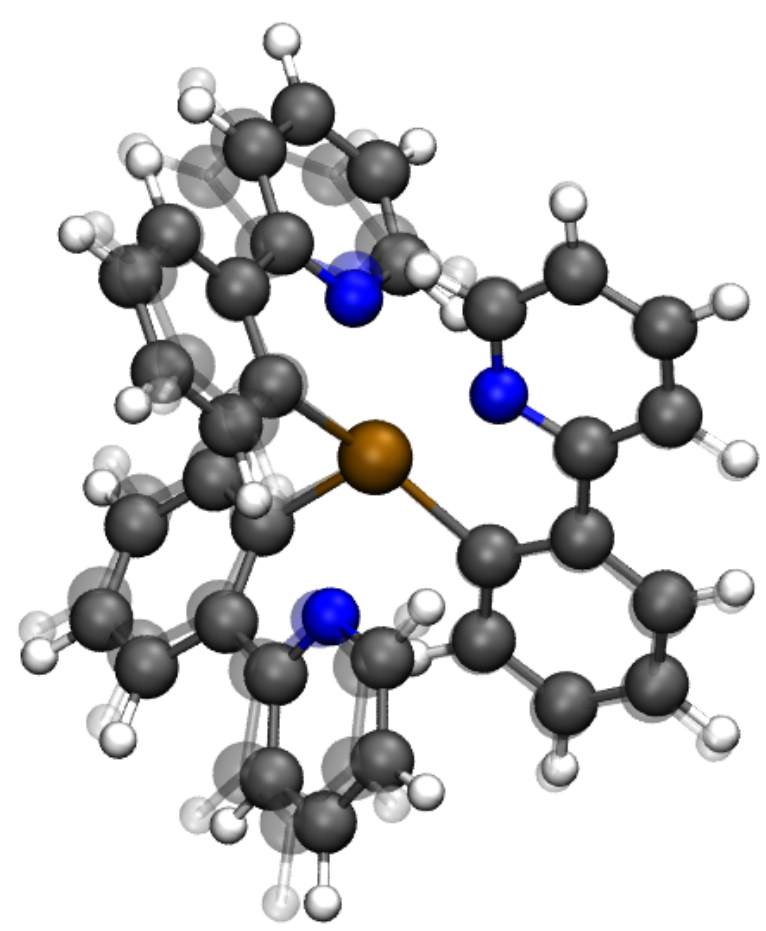

(h) ^{3}MC/MECP

Figure S3: Optimized structures (a−d) and structural overlays (e−h) of ***mer*-ppy3** calculated at the PBE0-D3/LANL2DZ[Ir]+6-311G(d,p)[C,H,N]/PCM(MeCN) level of theory.

Table S3: Structural parameters of optimized stationary points for ***mer*-ppy3** calculated at the PBE0-D3/LANL2DZ[Ir]+6-311G(d,p)[C,H,N]/PCM(MeCN) level of theory.

	Exp.[a]	GS	^{3}MLCT	^{3}MC	MECP
E (eV)	—	0.00	2.39	3.01	3.09
Spin[b] on Ir	—	—	0.42(0.45)	1.38(1.36)	—
Spin[b] on L1	—	—	0.17	0.20	—
Spin[b] on L2	—	—	1.25	0.25	—
Spin[b] on L3	—	—	0.18	0.18	—
r_1[c] (Å)	2.13	2.12	2.17	2.10	2.10
r_2[c] (Å)	2.06	2.06	2.04	2.21	2.29
r_3[c] (Å)	2.04	2.02	2.01	2.28	2.31
$\mathbb{R}$[d] (Å)	2.07 ± 0.02	2.07 ± 0.02	2.08 ± 0.04	2.26 ± 0.18	2.23 ± 0.25
p_1[e] (°)	3.68	1.58	2.71	0.19	0.06
p_2[e] (°)	0.13	0.63	2.93	3.65	15.72
p_3[e] (°)	1.19	0.64	0.55	18.66	23.02
$\mathbb{P}$[f] (°)	1.67 ± 1.82	0.95 ± 0.54	2.06 ± 1.32	7.50 ± 9.82	12.93 ± 11.73
$\mathbb{O}$[g] (°)	5.91	5.84	7.58	11.01	12.28

[a]Measured parameters from $Ir(CF_3ppy)_3$ crystal by Teng et al.[2] [b]Mulliken spin populations reported, with Löwdin spin populations in parentheses. [c]Average Ir–L distance of one ligand. [d]Average of all Ir–L contacts ± standard deviation. [e]Ligand torsion through bidentate dihedral angle. [f]Average ligand torsion ± standard deviation. [g]Average difference of L–Ir–L angles from 90°.

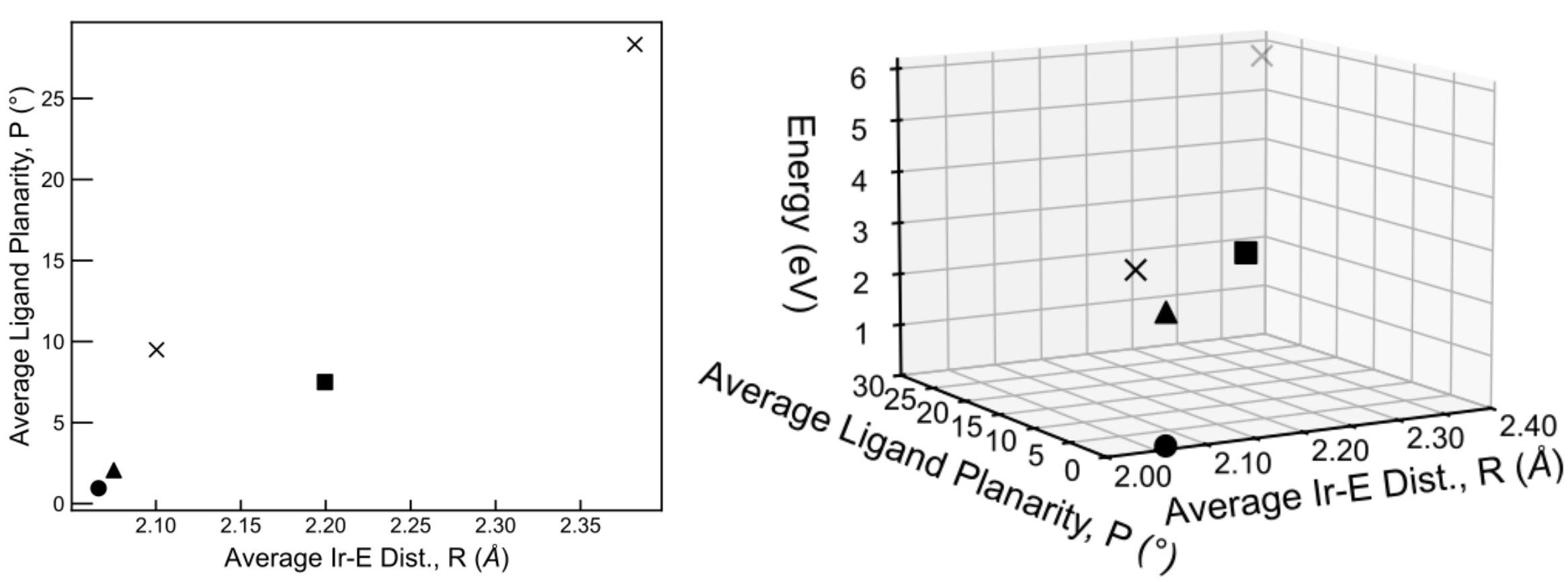

Figure S4: Structure-structure projected surfaces of guess structures surveyed in the search for a ***mer*-ppy3** ^{3}MC minimum. Freely-optimized GS, ^{3}MLCT, and ^{3}MC minima are indicated with a filled circle, triangle, and square respectively. Calculated at the PBE0-D3/LANL2DZ[Ir]+6-311G(d,p)[C,H,N]/PCM(MeCN) level of theory

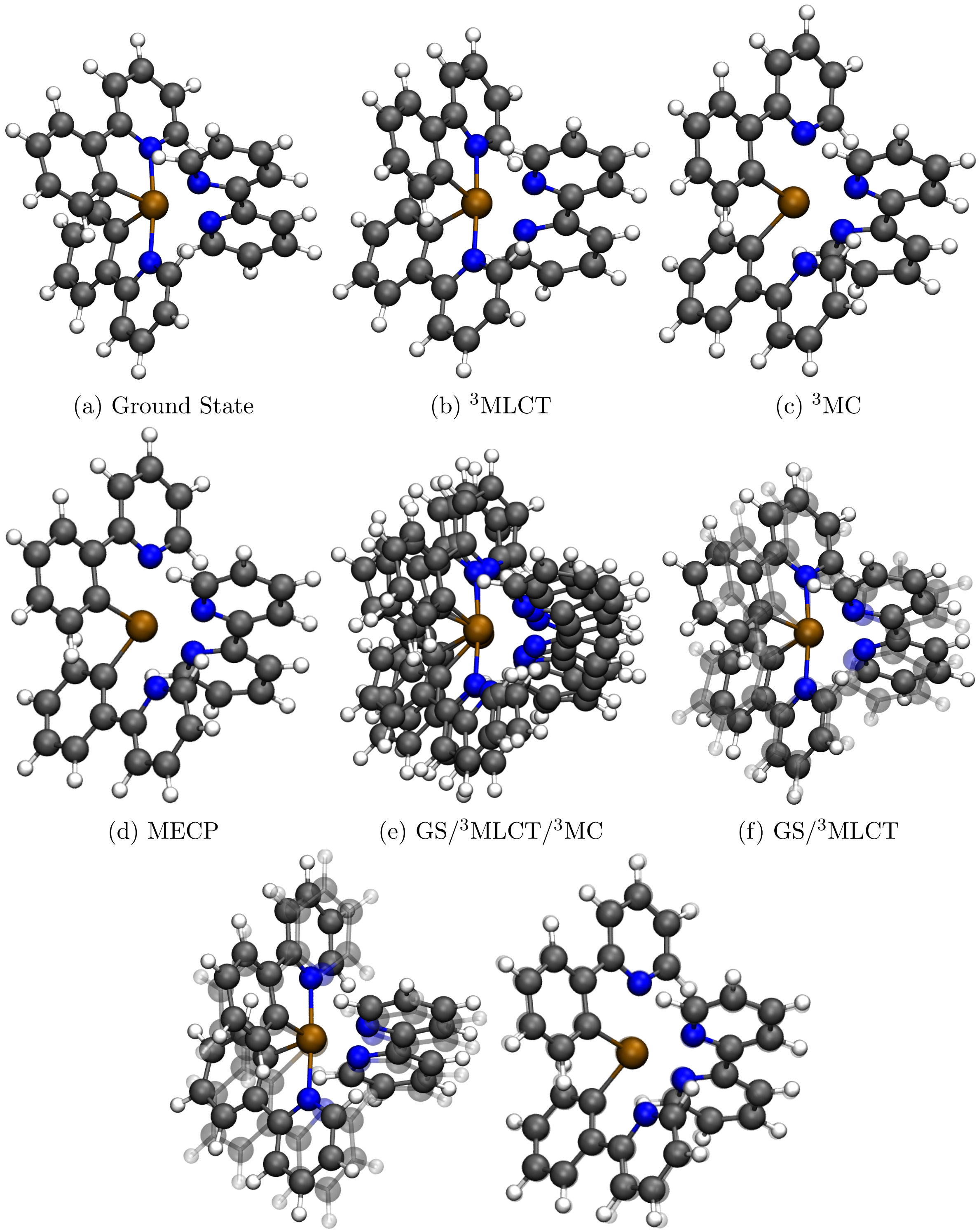

(a) Ground State (b) ^{3}MLCT (c) ^{3}MC

(d) MECP (e) GS/^{3}MLCT/^{3}MC (f) GS/^{3}MLCT

(g) ^{3}MLCT/^{3}MC (h) ^{3}MC/MECP

Figure S5: Optimized structures (a−d) and structural overlays (e−h) of ***m*-ppy2** calculated at the PBE0-D3/LANL2DZ[Ir]+6-311G(d,p)[C,H,N]/PCM(MeCN) level of theory.

Despite the synthetic prevalence of the ***m*-ppy2** isomer, a true "mer"-$[Ir(ppy)_2(bpy)]^+$ crystal was not found in crystallographic databases. Instead, a structural analogue[3] was found that maintained the *m*-$[Ir(C–N)_2(N–N)]$ nature of the complex. The bond lengths agree quite well, and the overall structural trends capture the different relationship of these "mer" ligands in a way that the ***f*-ppy2** crystal[4] cannot.

Table S4: Structural parameters of optimized stationary points for ***m*-ppy2** calculated at the PBE0-D3/LANL2DZ[Ir]+6-311G(d,p)/PCM(MeCN) level of theory.

	Exp.^{a}	GS	^{3}MLCT	^{3}MC	MECP
E (eV)	—	0.00	2.35	3.14	3.16
Spin^b on Ir	—	—	0.54(0.53)	1.53(1.49)	—
Spin^b on ppy1	—	—	0.23	0.13	—
Spin^b on ppy2	—	—	0.23	0.22	—
Spin^b on bpy	—	—	1.01	0.12	—
$r_1{}^c$ (Å)	2.02	2.03	2.02	2.27	2.12
$r_2{}^c$ (Å)	2.02	2.03	2.02	2.12	2.30
$r_3{}^c$ (Å)	2.14	2.15	2.15	2.20	2.20
$\mathbb{R}^d$ (Å)	2.06 ± 0.02	2.07 ± 0.02	2.06 ± 0.03	2.20 ± 0.19	2.21 ± 0.22
$p_1{}^e$ (°)	0.64	0.81	0.73	0.90	4.04
$p_2{}^e$ (°)	2.90	0.82	0.73	4.23	4.32
$p_3{}^e$ (°)	1.02	2.22	0.34	4.06	5.16
$\mathbb{P}^f$ (°)	1.52 ± 1.21	1.28 ± 0.81	0.60 ± 0.23	3.06 ± 1.87	4.50 ± 0.59
$\mathbb{O}^g$ (°)	5.86	6.53	6.38	12.61	13.28

^{a}Measured parameters from $Ir(Me\text{-}ppy)_2(phen^*)$ crystal by Yao et al,[3] where phen* = 1,10-phenanthrolino[5,6-c]pyrrole. ^{b}Mulliken spin populations reported, with Löwdin spin populations in parentheses. ^{c}Average Ir–L distance of one ligand. ^{d}Average of all Ir–L contacts ± standard deviation. ^{e}Ligand torsion through bidentate dihedral angle. ^{f}Average ligand torsion ± standard deviation. ^{g}Average difference of L–Ir–L angles from 90°.

The $[Ir(ppy)_2(bpy)]^+$ archetype complex is by far the most popular in both experimental and computational literature. As such, this is one of the few complexes in this study that can be directly compared to other calculated states. The Orti group has studied this particular complex and optimized various metal-centered states which they describe as ”axial” or ”equatorial,” characterized by the orientation of the longest bond(s) with respect to the bpy plane.[5] Mirroring the assignments of Orti group,[5] our search data has been colored to indicate the character of guess structure, where red points are considered axial and blue are considered equatorial (Figure S6). The open-face colored squares represent their ^{3}MCs using the coordinates found in their SI[5] for a single-point energy in our method (PBE0-D3/LANL2DZ[Ir]+6-311G(d,p)[C,H,N]/PCM(MeCN)). The filled colored squares are the output structures of those same states after re-optimization in our method. In this search, many guesses were equatorial in nature. Indeed, many equatorial input structures converged to axial outputs in this method as these axial and equatorial regions are superimposed at lower energies. The lowest-energy, freely-optimized ^{3}MC state reported in this study can be characterized as axial by the elongation of the N–Ir–N through-metal axis.

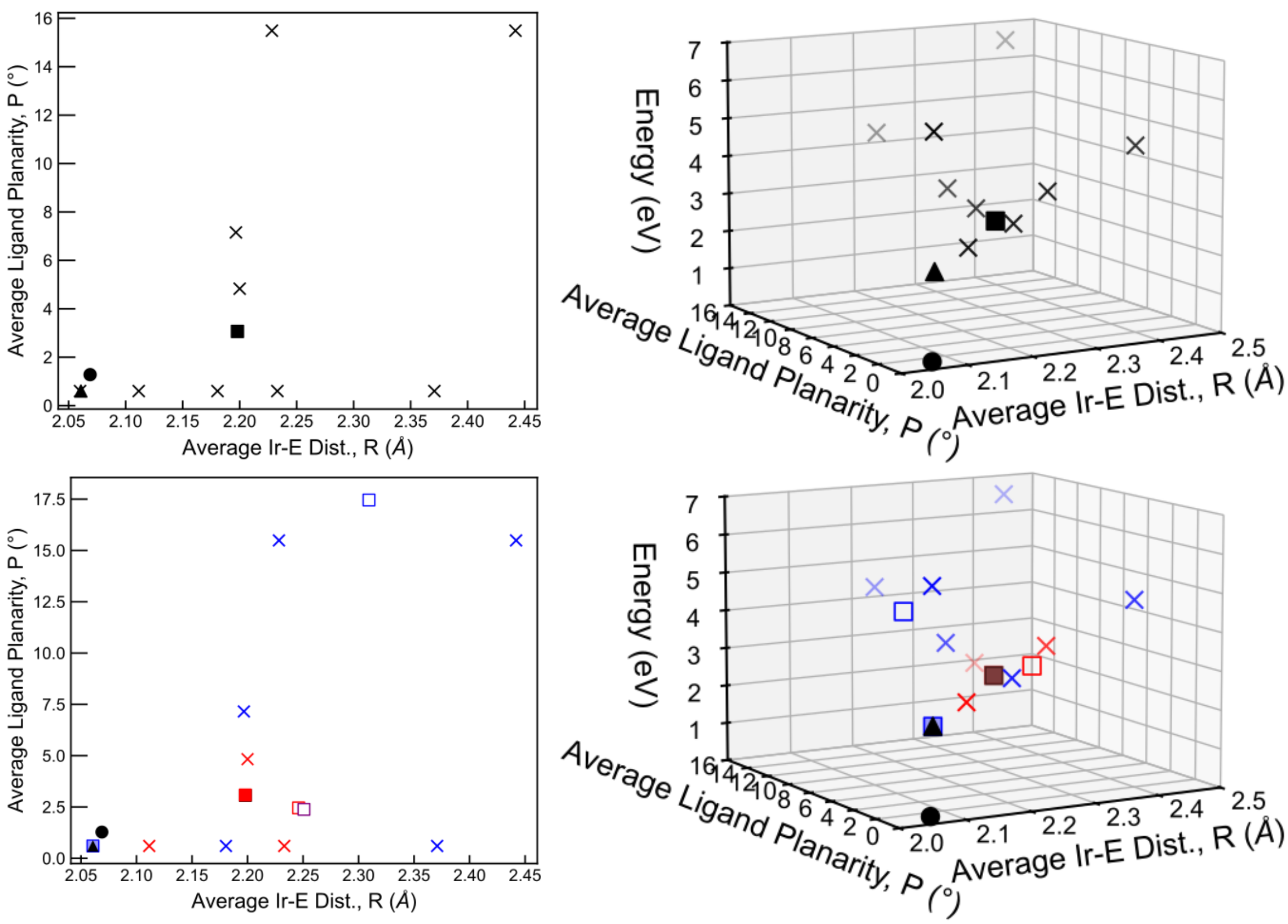

Figure S6: Structure-structure projected surfaces of guess structures surveyed in the search for a ***m*-ppy2** ^{3}MC minimum. Structures have been colored based on their assignment as "axial" (red), where the longest bonds are the trans Ir–N of the ppy ligands, or "equatorial" (blue), where the dominant change is in the plane of the bpy ligand. Freely-optimized GS, ^{3}MLCT, and ^{3}MC minima are indicated with a filled circle, triangle, and square respectively. Calculated at the PBE0-D3/LANL2DZ[Ir]+6-311G(d,p)[C,H,N]/PCM(MeCN) level of theory

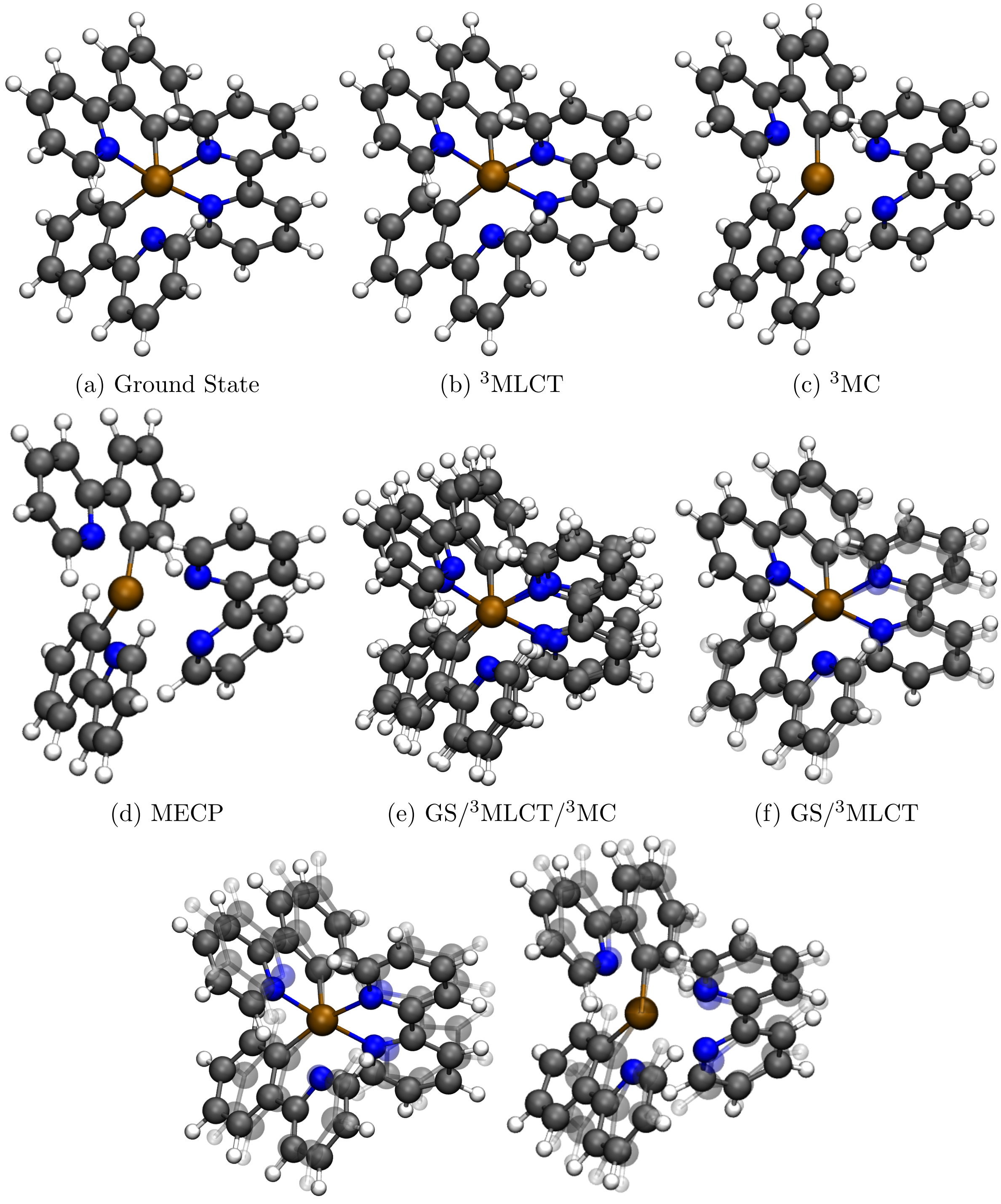

(a) Ground State (b) ^{3}MLCT (c) ^{3}MC (d) MECP (e) GS/^{3}MLCT/^{3}MC (f) GS/^{3}MLCT (g) ^{3}MLCT/^{3}MC (h) ^{3}MC/MECP

Figure S7: Optimized structures (a−d) and structural overlays (e−h) ***f*-ppy2** calculated at the PBE0-D3/LANL2DZ[Ir]+6-311G(d,p)[C,H,N]/PCM(MeCN) level of theory.

Table S5: Structural parameters of optimized stationary points for ***f*-ppy2** calculated at the PBE0-D3/LANL2DZ[Ir]+6-311G(d,p)[C,H,N]/PCM(MeCN) level of theory.

	Exp.[a]	GS	^{3}MLCT	^{3}MC	MECP
E (eV)	—	0.00	2.32	3.03	3.15
Spin[b] on Ir	—	—	0.57(0.55)	1.39(1.35)	—
Spin[b] on ppy1	—	—	0.33	0.17	—
Spin[b] on ppy2	—	—	0.72	0.05	—
Spin[b] on bpy	—	—	0.39	0.39	—
r_1[b] (Å)	2.04	2.03	2.01	2.12	2.07
r_2[b] (Å)	2.10	2.09	2.12	2.07	2.12
r_3[b] (Å)	2.11	2.09	2.06	2.34	2.50
$\mathbb{R}$[c] (Å)	2.08 ± 0.02	2.07 ± 0.02	2.06 ± 0.03	2.18 ± 0.06	2.23 ± 0.28
p_1[d] (°)	1.72	0.46	0.05	12.26	4.09
p_2[d] (°)	4.00	1.29	2.41	1.90	7.33
p_3[d] (°)	3.09	1.25	2.49	15.86	27.14
$\mathbb{P}$[e] (°)	2.94 ± 1.15	1.00 ± 0.47	1.65 ± 1.39	10.01 ± 7.25	12.85
$\mathbb{O}$[f] (°)	6.15	6.31	6.98	8.71	11.66

[a]Measured parameters from $Ir(ppy)_2(bpy)$ crystal by Ma et al.[4] [b]Mulliken spin populations reported, with Löwdin spin populations in parentheses. [c]Average Ir–L distance of one ligand. [d]Average of all Ir–L contacts ± standard deviation. [e]Ligand torsion through bidentate dihedral angle. [f]Average ligand torsion ± standard deviation. [g]Average difference of L–Ir–L angles from 90°.

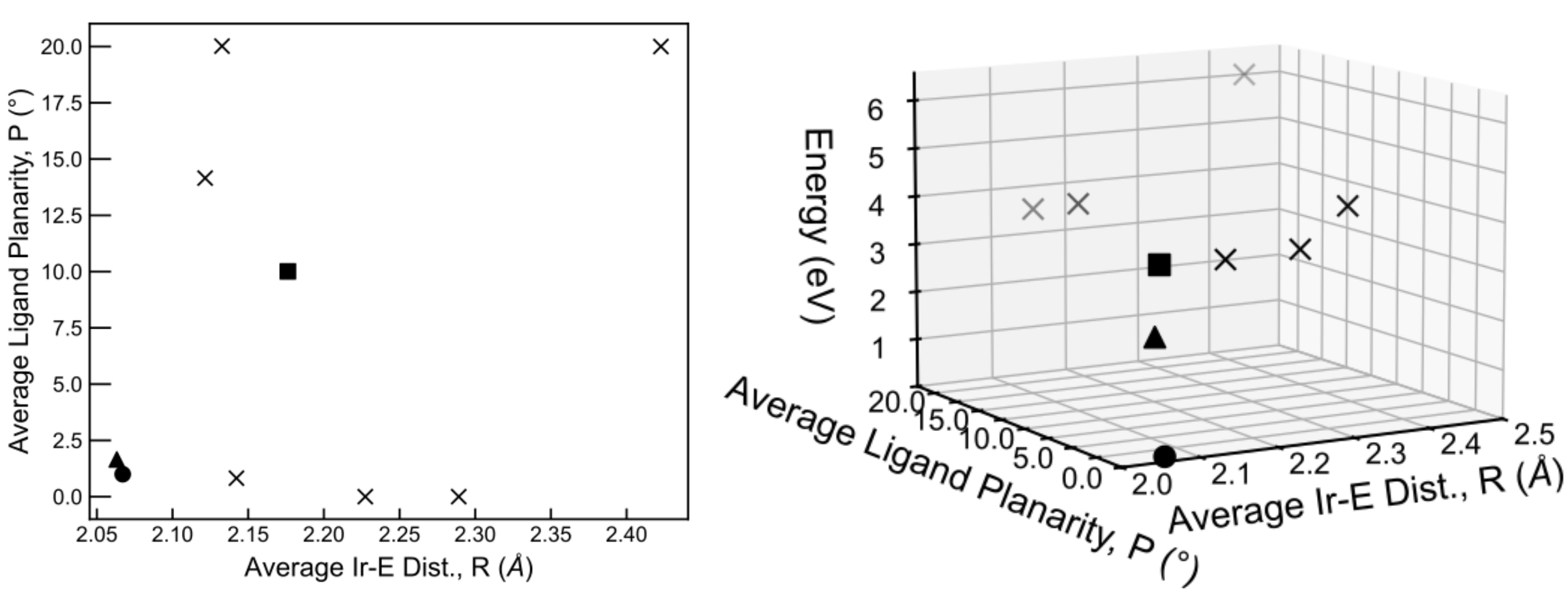

Figure S8: Structure-structure projected surfaces of guess structures surveyed in the search for a ***f*-ppy2** ^{3}MC minimum. Freely-optimized GS, ^{3}MLCT, and ^{3}MC minima are indicated with filled circle, triangle, and square respectively. Calculated at the PBE0-D3/LANL2DZ[Ir]+6-311G(d,p)[C,H,N]/PCM(MeCN) level of theory

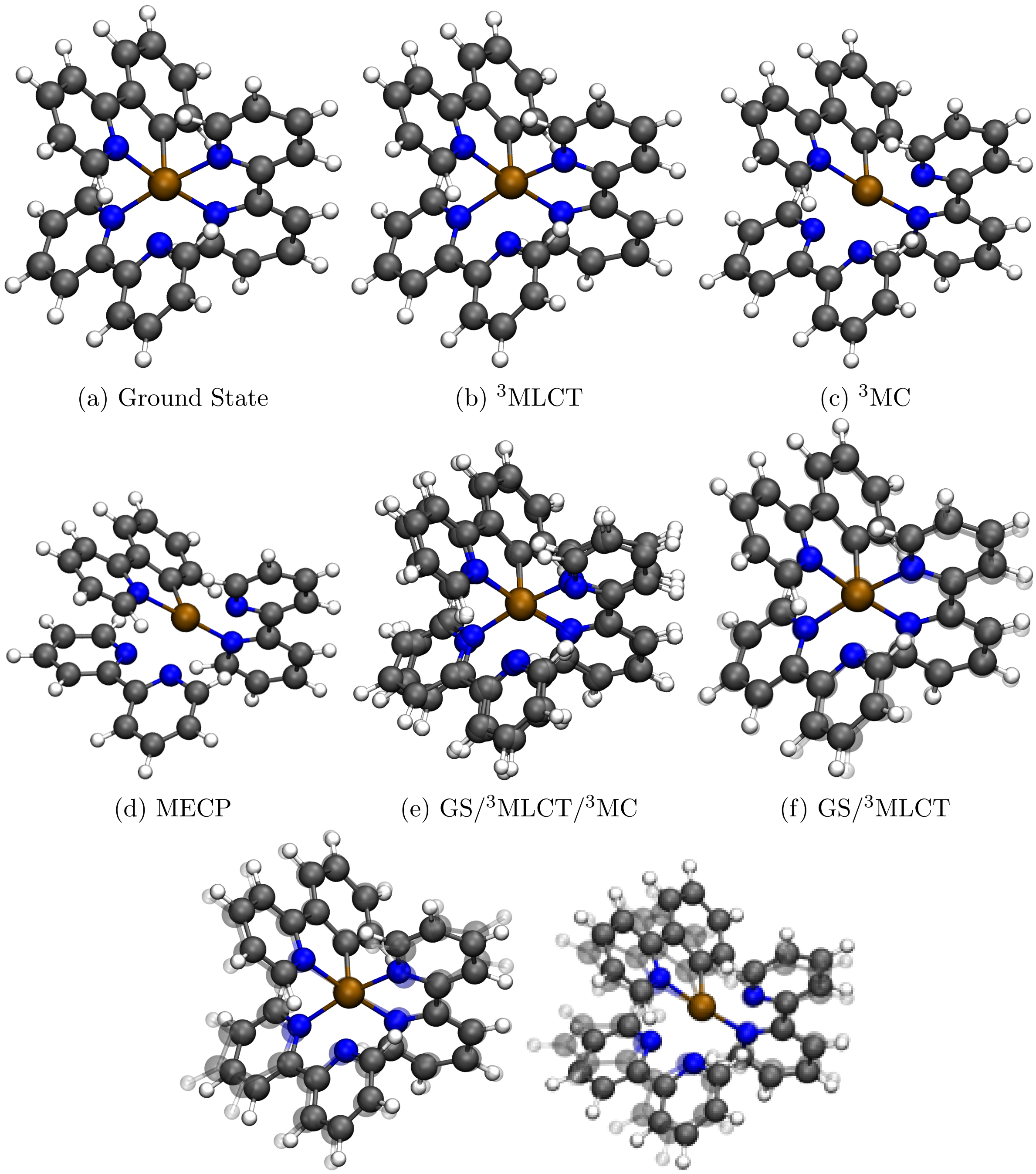

(a) Ground State (b) ^{3}MLCT (c) ^{3}MC

(d) MECP (e) GS/^{3}MLCT/^{3}MC (f) GS/^{3}MLCT

(g) ^{3}MLCT/^{3}MC (h) ^{3}MC/MECP

Figure S9: Optimized structures (a−d) and structural overlays (e−h) of **bpy2** calculated at the PBE0-D3/LANL2DZ[Ir]+6-311G(d,p)[C,H,N]/PCM(MeCN) level of theory.

Table S6: Structural parameters of optimized stationary points for **bpy2** calculated at the PBE0-D3/LANL2DZ[Ir]+6-311G(d,p)[C,H,N]/PCM(MeCN) level of theory.

	Exp.^{a}	GS	^{3}MLCT	^{3}MC	MECP
E (eV)	—	0.00	2.66	2.91	3.09
Spin^b on Ir	—	—	0.45(0.44)	1.34(1.29)	—
Spin^b on ppy	—	—	0.33	0.13	—
Spin^b on bpy1	—	—	0.01	0.14	—
Spin^b on bpy2	—	—	1.21	0.39	—
$r_1{}^c$ (Å)	2.03	2.04	2.01	2.01	2.01
$r_2{}^c$ (Å)	2.06	2.10	2.13	2.30	2.15
$r_3{}^c$ (Å)	2.04	2.04	2.02	2.17	2.47
$\mathbb{R}^d$ (Å)	2.05 ± 0.01	2.06 ± 0.02	2.05 ± 0.03	2.16 ± 0.06	2.21 ± 0.29
$p_1{}^e$ (°)	2.75	1.08	1.02	0.84	4.86
$p_2{}^e$ (°)	4.06	1.86	3.91	13.33	10.31
$p_3{}^e$ (°)	0.12	0.81	2.03718.91	31.08	
$\mathbb{P}^f$ (°)	2.31 ± 2.01	1.25 ± 0.54	2.32 ± 1.47	11.03 ± 9.25	15.42 ± 13.84
$\mathbb{O}^g$ (°)	6.48	6.70	6.57	8.85	12.07

^{a}Measured parameters from $[Ir(bpy)_2(H_2O)(bpy)]^{3+}$ crystal by Wickramasinghe et al.[6]
^{b}Mulliken spin populations reported, with Löwdin spin populations in parentheses.
^{c}Average Ir–L distance of one ligand. ^{d}Average of all Ir–L contacts ± standard deviation.
^{e}Ligand torsion through bidentate dihedral angle. ^{f}Average ligand torsion ± standard deviation. ^{g}Average difference of L–Ir–L angles from 90°.

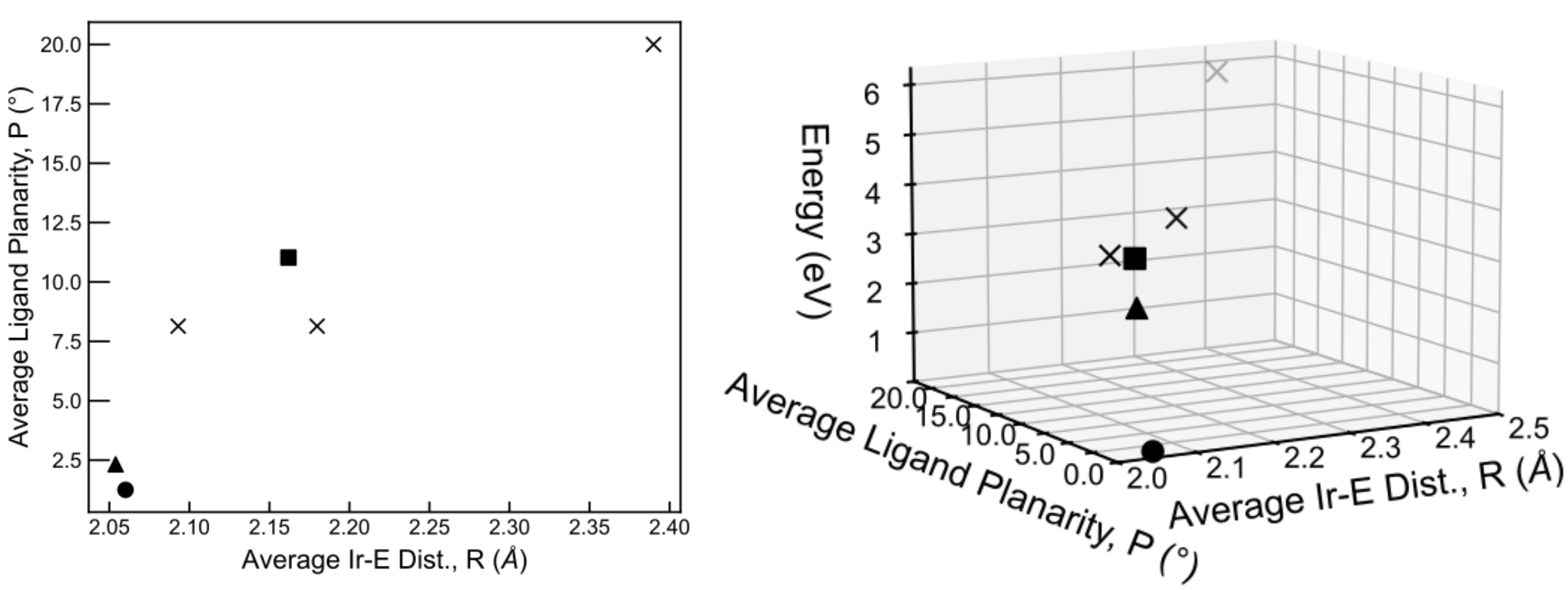

Figure S10: Structure-structure projected surfaces of guess structures surveyed in the search for a **bpy2** ^{3}MC minimum. Freely-optimized GS, ^{3}MLCT, and ^{3}MC minima are indicated with filled circle, triangle, and square respectively. Calculated at the PBE0-D3/LANL2DZ[Ir]+6-311G(d,p)[C,H,N]/PCM(MeCN) level of theory

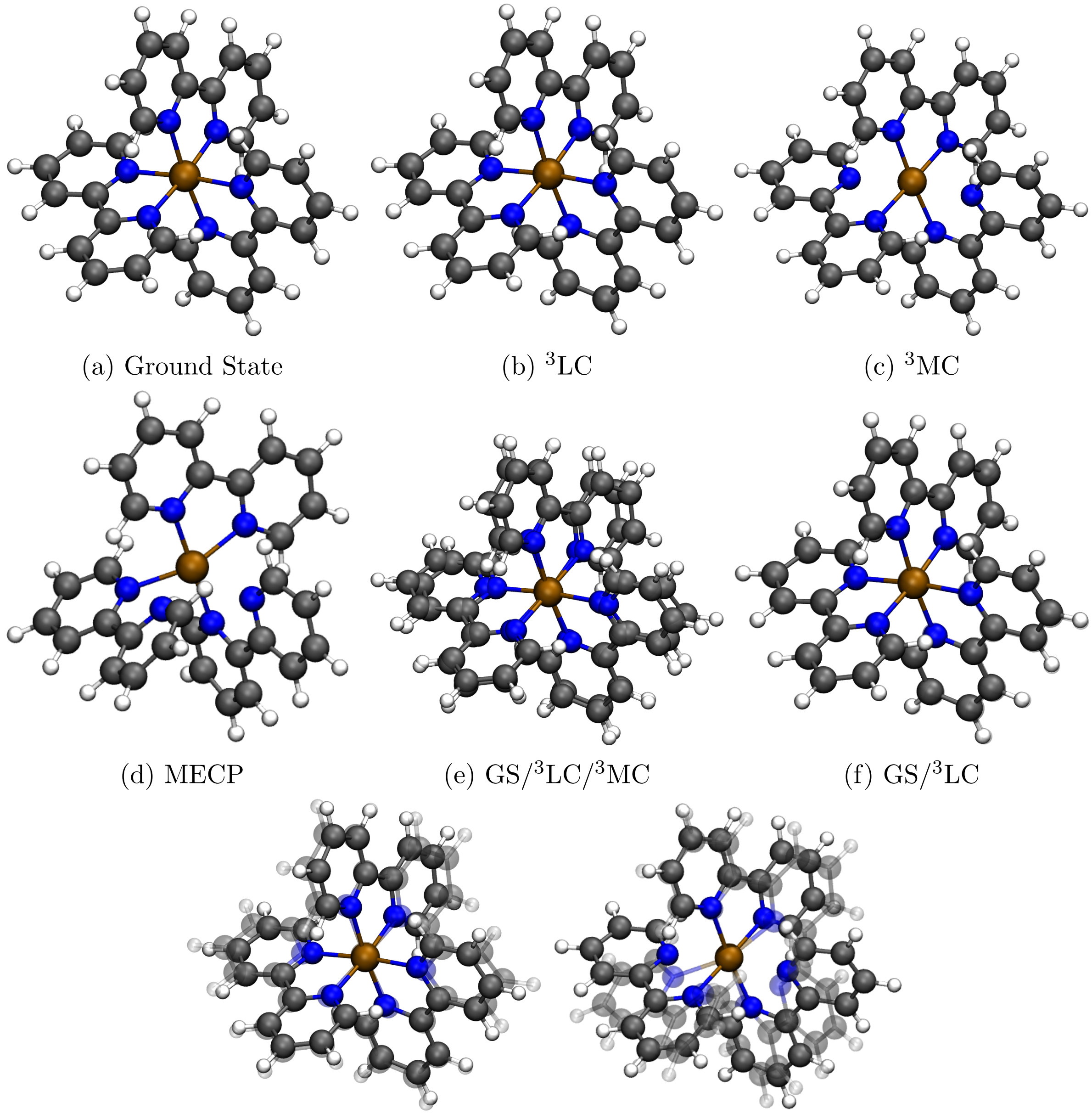

(a) Ground State (b) ^{3}LC (c) ^{3}MC

(d) MECP (e) GS/^{3}LC/^{3}MC (f) GS/^{3}LC

(g) ^{3}LC/^{3}MC (h) ^{3}MC/MECP

Figure S11: Optimized structures (a–d) and structural overlays (e–h) of **bpy3** calculated at the PBE0-D3/LANL2DZ[Ir]+6-311G(d,p)[C,H,N]/PCM(MeCN) level of theory.

Table S7: Structural parameters of optimized stationary points for **bpy3** calculated at the PBE0-D3/LANL2DZ[Ir]+6-311G(d,p)[C,H,N]/PCM(MeCN) level of theory.

	Exp.^{a}	GS	^{3}LC	^{3}MC	MECP
E (eV)	—	0.00	2.83	3.34	3.34
Spin^b on Ir	—	—	0.03(0.04)	1.66(1.59)	—
Spin^b on L1	—	—	0.00	0.17	—
Spin^b on L2	—	—	1.97	0.15	—
Spin^b on L3	—	—	0.00	0.02	—
$r_1{}^b$ (Å)	—	2.05	2.05	2.24	2.05
$r_2{}^b$ (Å)	2.06	2.05	2.04	2.24	2.26
$r_3{}^b$ (Å)	2.04	2.05	2.05	2.05	2.21
$\mathbb{R}^c$ (Å)	—	2.05 ± 0.00	2.05 ± 0.00	2.18 ± 0.06	2.17 ± 0.16
$p_1{}^d$ (°)	—	1.74	1.67	6.90	2.31
$p_2{}^d$ (°)	4.06	1.89	1.39	13.16	7.54
$p_3{}^d$ (°)	0.12	1.68	1.67	0.03	0.40
$\mathbb{P}^e$ (°)	—	1.77 ± 0.11	1.58 ± 0.16	6.70 ± 6.57	3.42 ± 3.70
$\mathbb{O}^f$ (°)	—	6.89	6.69	11.43	15.61

^{a}Measured parameters from $[Ir(bpy)_2(H_2O)(bpy)]^{3+}$ crystal by Wickramasinghe et al.[6]
^{b}Mulliken spin populations reported, with Löwdin spin populations in parentheses.
^{c}Average Ir–L distance of one ligand. ^{d}Average of all Ir–L contacts ± standard deviation.
^{e}Ligand torsion through bidentate dihedral angle. ^{f}Average ligand torsion ± standard deviation. ^{g}Average difference of L–Ir–L angles from 90°.

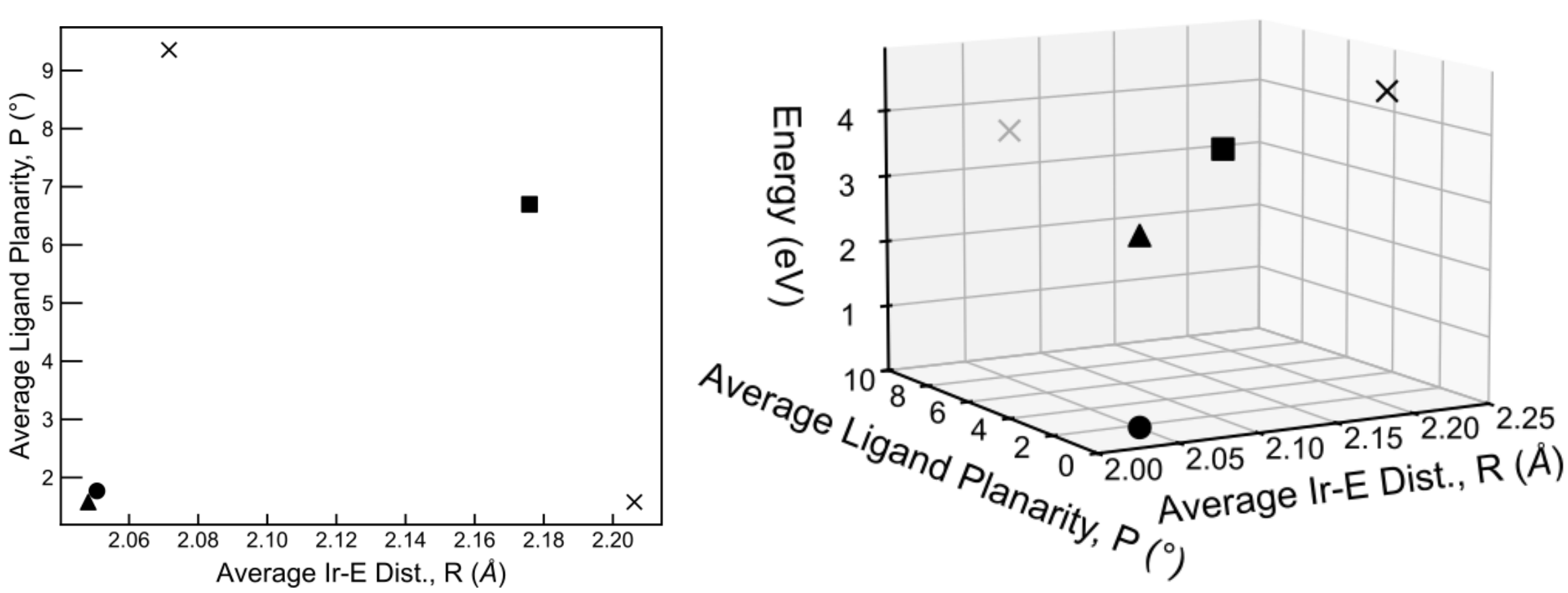

Figure S12: Structure-structure projected surfaces of guess structures surveyed in the search for a **bpy3** ^{3}MC minimum. Freely-optimized GS, ^{3}MLCT, and ^{3}MC minima are indicated with filled circle, triangle, and square respectively. Calculated at the PBE0-D3/LANL2DZ[Ir]+6-311G(d,p)[C,H,N]/PCM(MeCN) level of theory

In the case of ***fac*-bzq3**, both GS and ^{3}MLCT structures were freely-optimized, but no optimized metal-centered structure was found. Over 50 distinct complexes were sampled, but any attempts to optimize the structures of metal-centered character converged to the ^{3}MLCT complex for both free and constrained optimizations (Figures S13 and S15). These plots demonstrate the area covered within this search, and also begin to highlight why this failed search may be unsurprising; along every projected axis, the ^{3}MLCT curve (triangles) lies along, or entirely surrounds, the ^{3}MC curve (squares). Even constrained optimization attempts from the lowest-energy ^{3}MC point, which was used as the ^{3}MC state for this study, lost metal-centered spin character within one step of the calculation. In absence of a freely-optimized minimum, the lowest-energy SCF-structure with Ir spin >1.00 was chosen to act as a ^{3}MC complex for this study. Though this complex is not an optimized minimum (four imaginary modes), this structure follows trends observed for other complexes within this study, supporting it's reasonable selection.

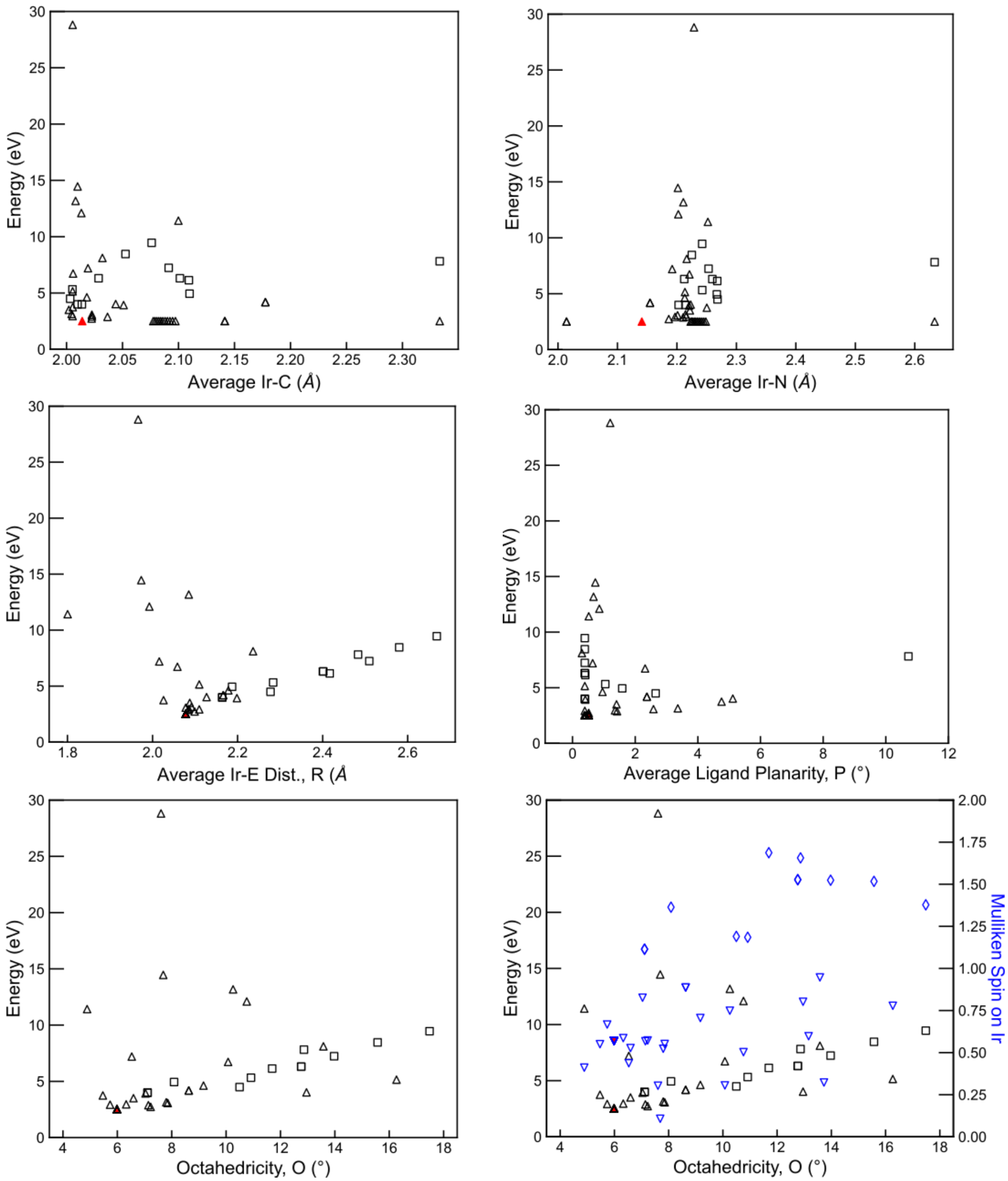

Figure S13: Guess structures surveyed in the search for a ***fac*-bzq3** ^{3}MC minimum at the PBE0-D3/LANL2DZ[Ir]+6-311G(d,p)[C,H,N]/PCM(MeCN) level of theory. Triangles are triplets with CT character, squares are those with MC character. The freely-optimized ^{3}MLCT is emphasized in red.

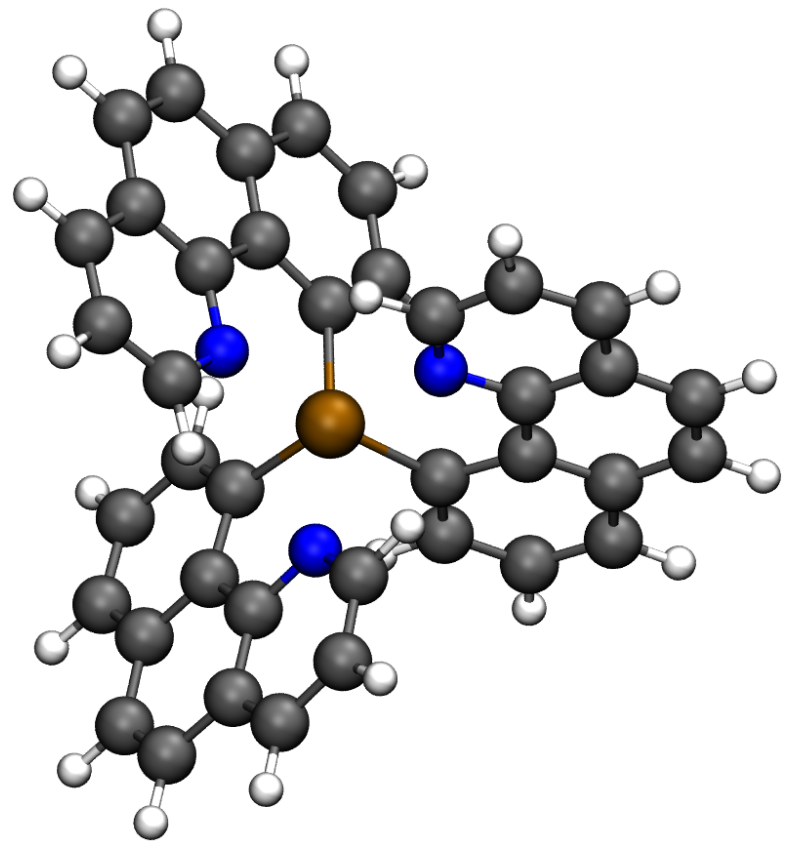

(a) Ground State

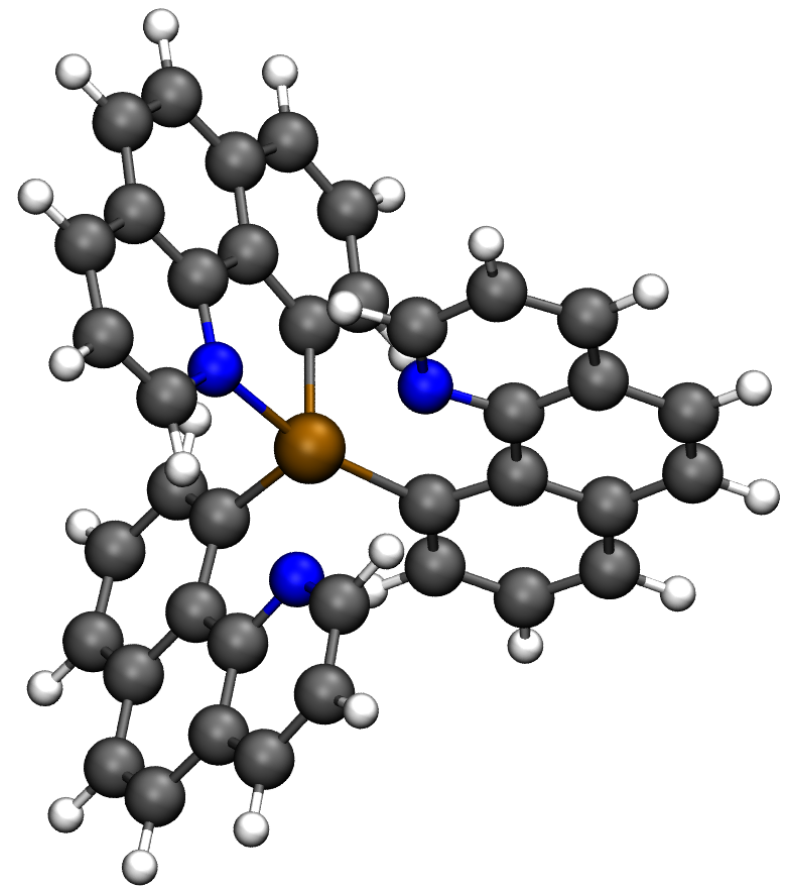

(b) ^{3}MLCT

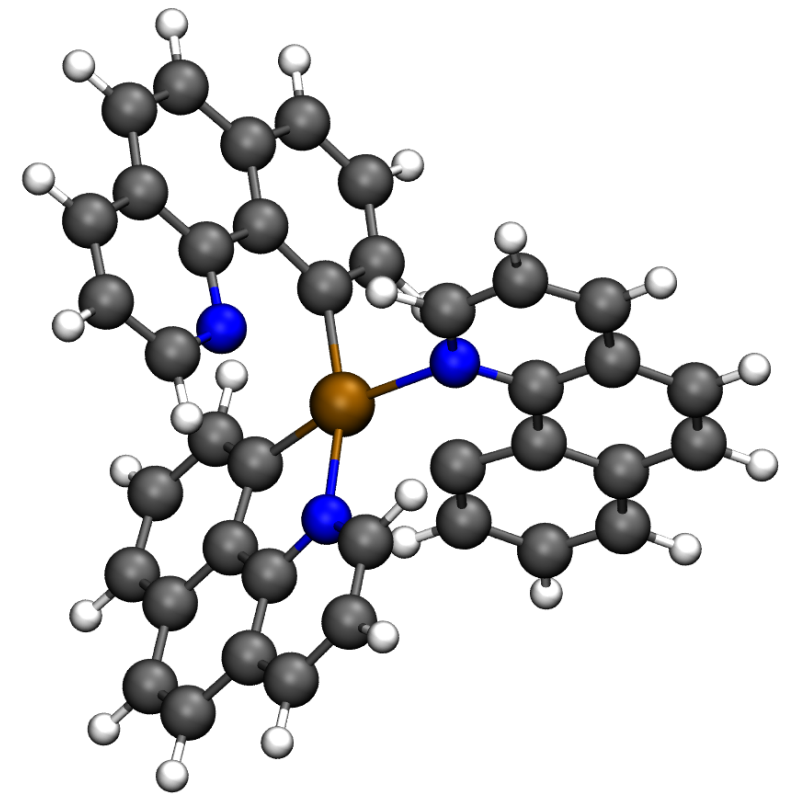

(c) ^{3}MC*

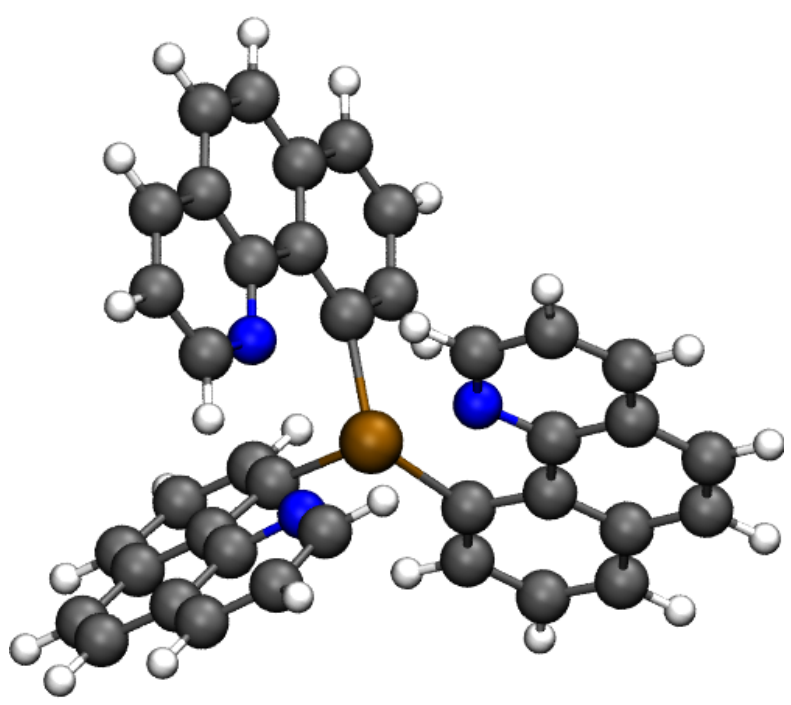

(d) MECP

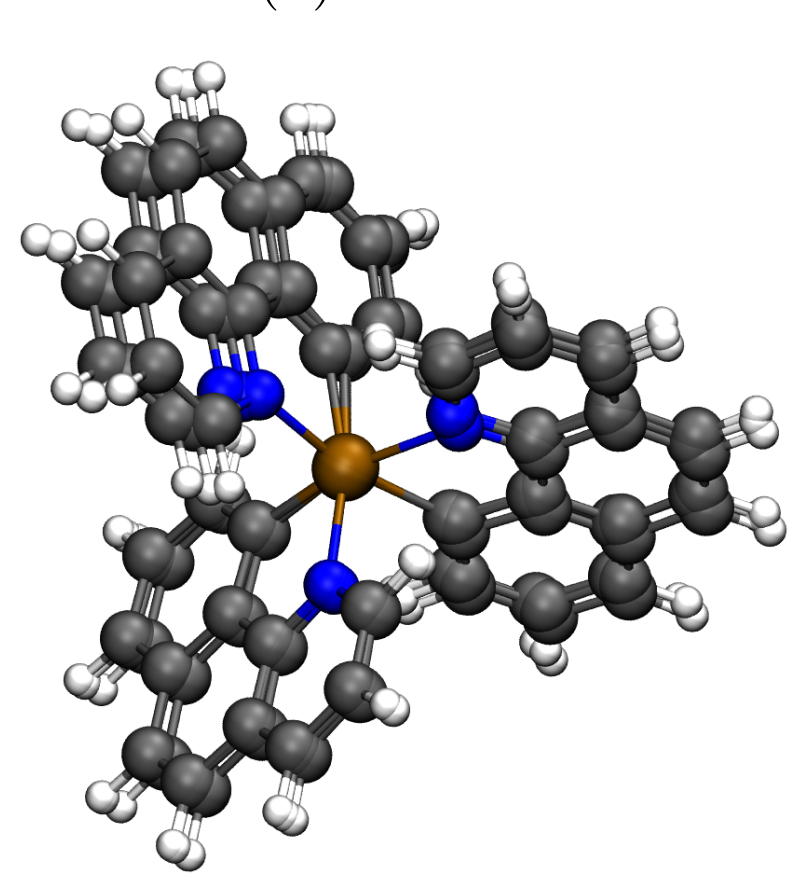

(e) GS/^{3}MLCT/^{3}MC*

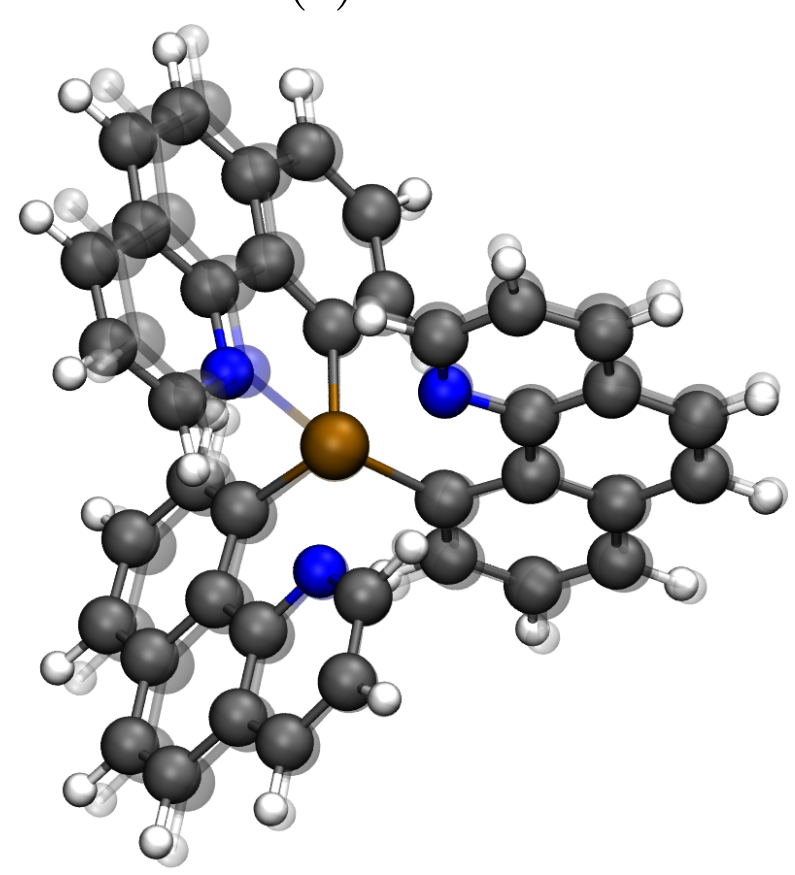

(f) GS/^{3}MLCT

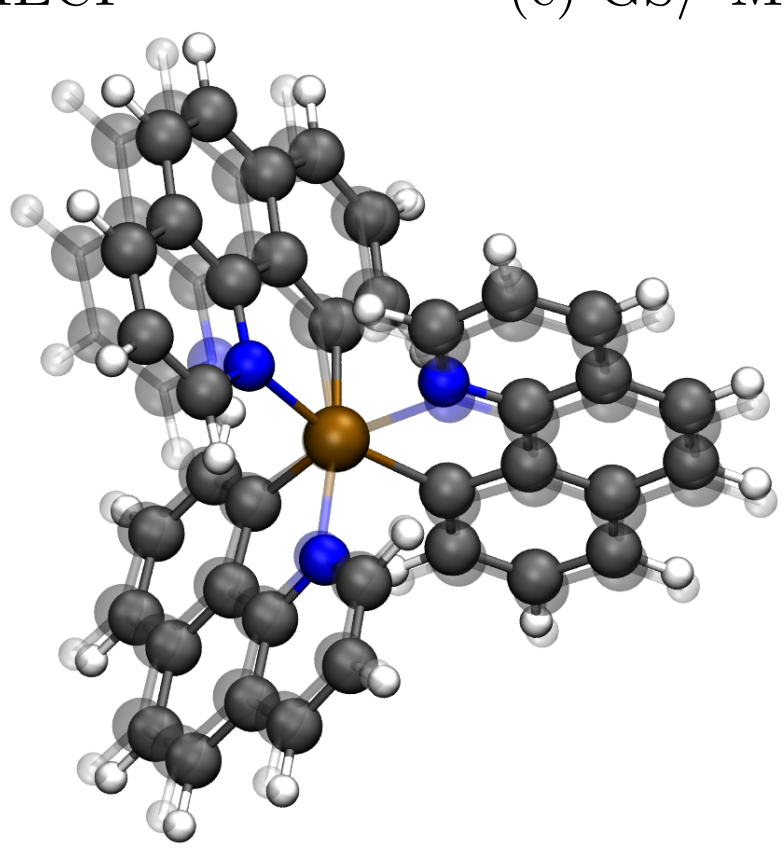

(g) ^{3}MLCT/^{3}MC*

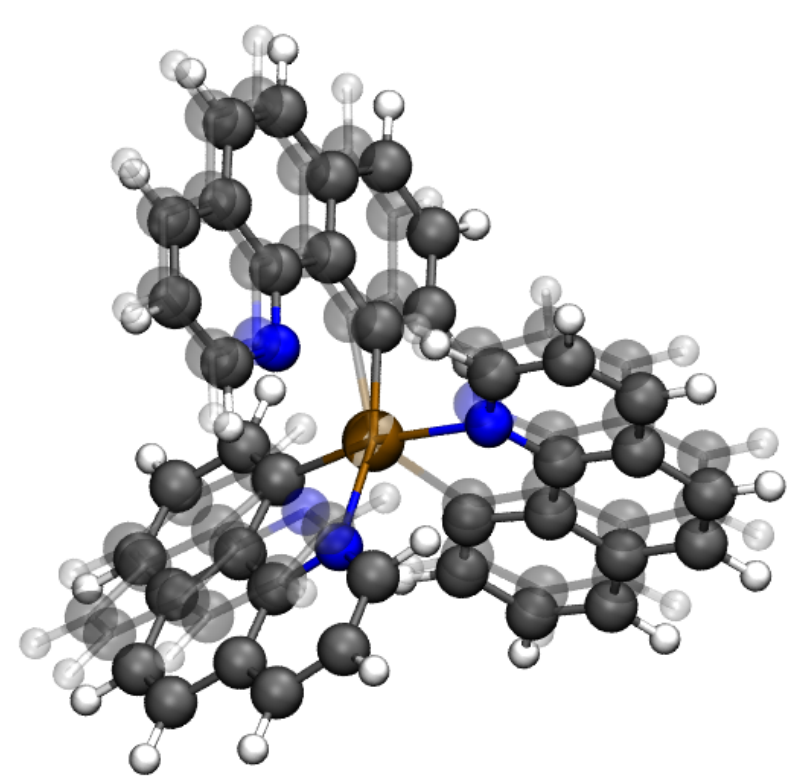

(h) ^{3}MC*/MECP

Figure S14: Optimized structures (a–d) and structural overlays (e–h) of ***fac*-bzq3** calculated at the PBE0-D3/LANL2DZ[Ir]+6-311G(d,p)[C,H,N]/PCM(MeCN) level of theory. *Lowest-energy ^{3}MC. See Methods.

Table S8: Structural parameters of optimized stationary points for ***fac*-bzq3** calculated at the PBE0-D3/LANL2DZ[Ir]+6-311G(d,p)[C,H,N]/PCM(MeCN) level of theory.

	Exp.[a]	GS	^{3}MLCT	^{3}MC*	MECP
E (eV)	—	0.00	2.51	3.99	3.29
Spin[b] on Ir	—	—	0.57(0.56)	1.11 (1.06)	—
Spin[b] on L1	—	—	1.16	0.10	—
Spin[b] on L2	—	—	0.03	0.05	—
Spin[b] on L3	—	—	0.24	0.73	—
r_1[c] (Å)	2.03	2.08	2.04	2.24	2.44
r_2[c] (Å)	2.03	2.08	2.12	2.20	2.13
r_3[c] (Å)	—	2.08	2.07	2.05	2.09
$\mathbb{R}$[d] (Å)	—	2.08 ± 0.03	2.08 ± 0.03	2.16 ± 0.07	2.22 ± 0.30
p_1[e] (°)	1.36	0.31	0.69	0.31	3.69
p_2[e] (°)	0.15	0.51	0.66	0.51	0.17
p_3[e] (°)	—	0.35	0.20	0.35	0.62
$\mathbb{P}$[f] (°)	—	0.39 ± 0.11	0.52 ± 0.27	0.39 ± 0.11	1.49 ± 1.92
$\mathbb{O}$[g] (°)	—	5.00	5.99	7.11	12.20

[a]Measured parameters from Ir(bzq)$_2$(H-FQ) crystal by Qin et al.[7] [b]Mulliken spin populations reported, with Löwdin spin populations in parentheses. [c]Average Ir–L distance of one ligand. [d]Average of all Ir–L contacts ± standard deviation. [e]Ligand torsion through bidentate dihedral angle. [f]Average ligand torsion ± standard deviation. [g]Average difference of L–Ir–L angles from 90°.
*Lowest-energy SCF structure of ^{3}MC character, see Methods.

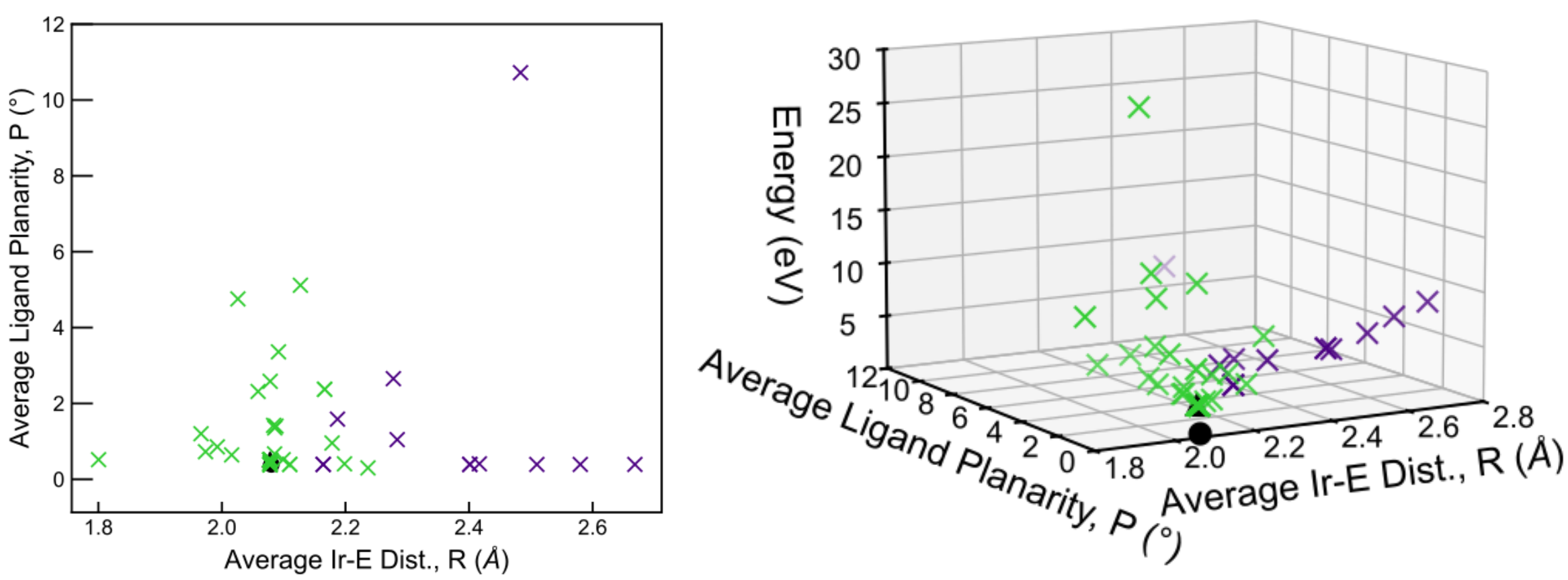

Figure S15: Structure-structure projected surfaces of guess structures surveyed in the search for a ***fac*-bzq3** ^{3}MC minimum. Freely-optimized GS and ^{3}MLCT minima are indicated with a filled circle and triangle respectively. Green X's are triplets with MLCT character and purple X's are triplets with MC character. Calculated at the PBE0-D3/LANL2DZ[Ir]+6-311G(d,p)[C,H,N]/PCM(MeCN) level of theory

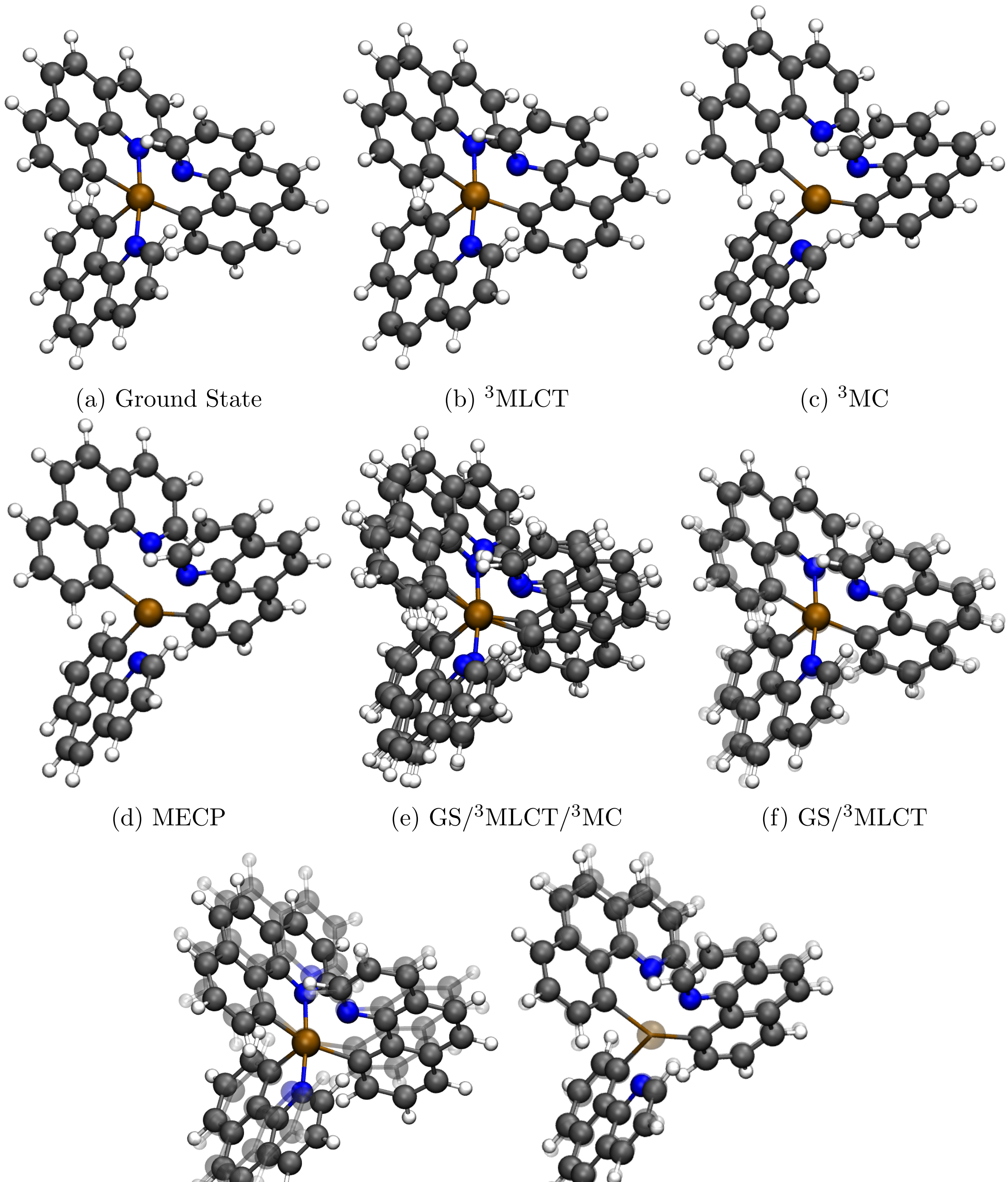

(a) Ground State (b) ^{3}MLCT (c) ^{3}MC

(d) MECP (e) GS/^{3}MLCT/^{3}MC (f) GS/^{3}MLCT

(g) ^{3}MLCT/^{3}MC (h) ^{3}MC/MECP

Figure S16: Optimized structures (a−d) and structural overlays (e−h) of ***mer*-bzq3** calculated at the PBE0-D3/LANL2DZ[Ir]+6-311G(d,p)[C,H,N]/PCM(MeCN) level of theory.

Table S9: Structural parameters of optimized stationary points for ***mer*-bzq3** calculated at the PBE0-D3/LANL2DZ[Ir]+6-311G(d,p)[C,H,N]/PCM(MeCN) level of theory.

	Exp.[a]	GS	^{3}MLCT	^{3}MC	MECP
E (eV)	—	0.00	2.44	2.80	2.81
Spin[b] on Ir	—	—	0.40(0.42)	1.30(1.31)	—
Spin[b] on L1	—	—	0.74	0.16	—
Spin[b] on L2	—	—	0.72	0.26	—
Spin[b] on L3	—	—	0.14	0.28	—
r_1[c] (Å)	2.03	2.02	2.01	2.14	2.13
r_2[c] (Å)	2.03	2.12	2.16	2.10	2.44
r_3[c] (Å)	—	2.07	2.06	2.40	2.09
$\mathbb{R}$[d] (Å)	—	2.07 ± 0.02	2.08 ± 0.03	2.21 ± 0.10	2.22 ± 0.30
p_1[e] (°)	1.36	0.24	0.26	0.02	0.16
p_2[e] (°)	0.15	0.64	0.81	0.24	3.70
p_3[e] (°)	—	0.25	0.04	1.43	0.64
$\mathbb{P}$[f] (°)	—	0.38 ± 0.23	0.37 ± 0.40	0.56 ± 0.76	1.50 ± 1.92
$\mathbb{O}$[g] (°)	—	5.14	6.55	11.23	12.20

[a]Measured parameters from $Ir(bzq)_2$(H-FQ) crystal by Qin et al.[7] [b]Mulliken spin populations reported, with Löwdin spin populations in parentheses. [c]Average Ir–L distance of one ligand. [d]Average of all Ir–L contacts ± standard deviation. [e]Ligand torsion through bidentate dihedral angle. [f]Average ligand torsion ± standard deviation. [g]Average difference of L–Ir–L angles from 90°.

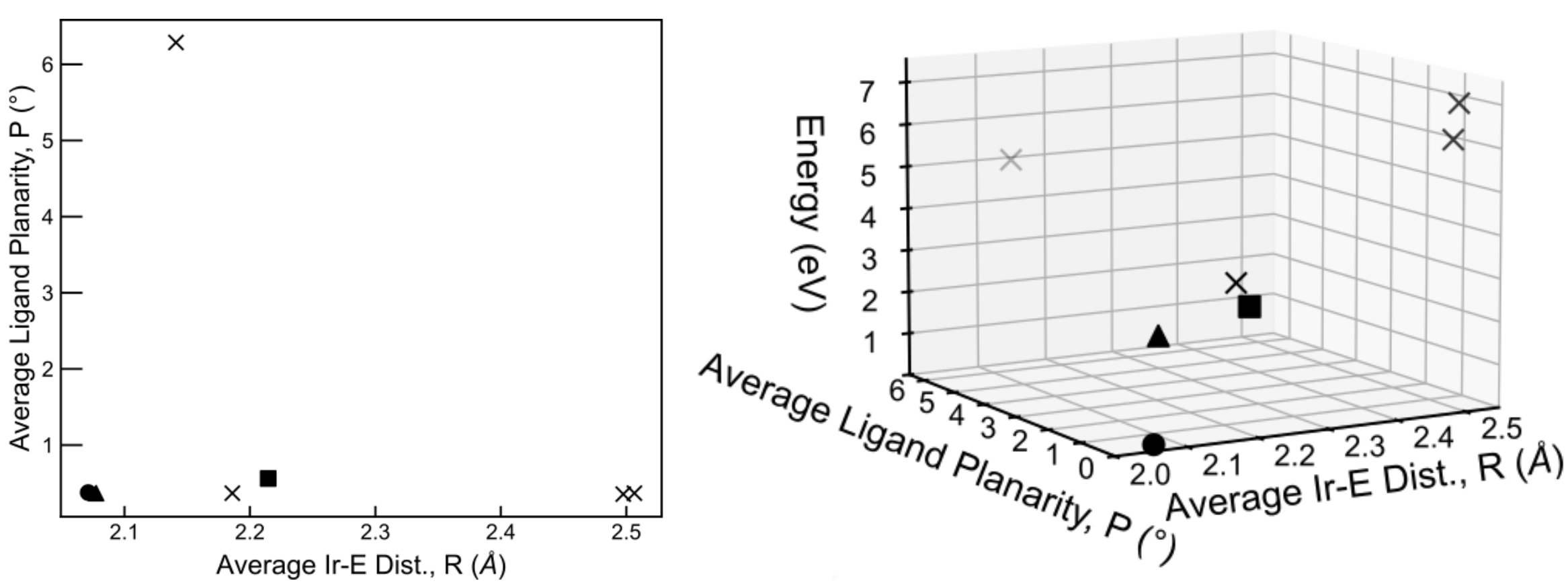

Figure S17: Structure-structure projected surfaces of guess structures surveyed in the search for a ***mer*-bzq3** ^{3}MC minimum. Freely-optimized GS, ^{3}MLCT, and ^{3}MC structures are indicated with a filled circle, triangle, and square respectively. Calculated at the PBE0-D3/LANL2DZ[Ir]+6-311G(d,p)[C,H,N]/PCM(MeCN) level of theory

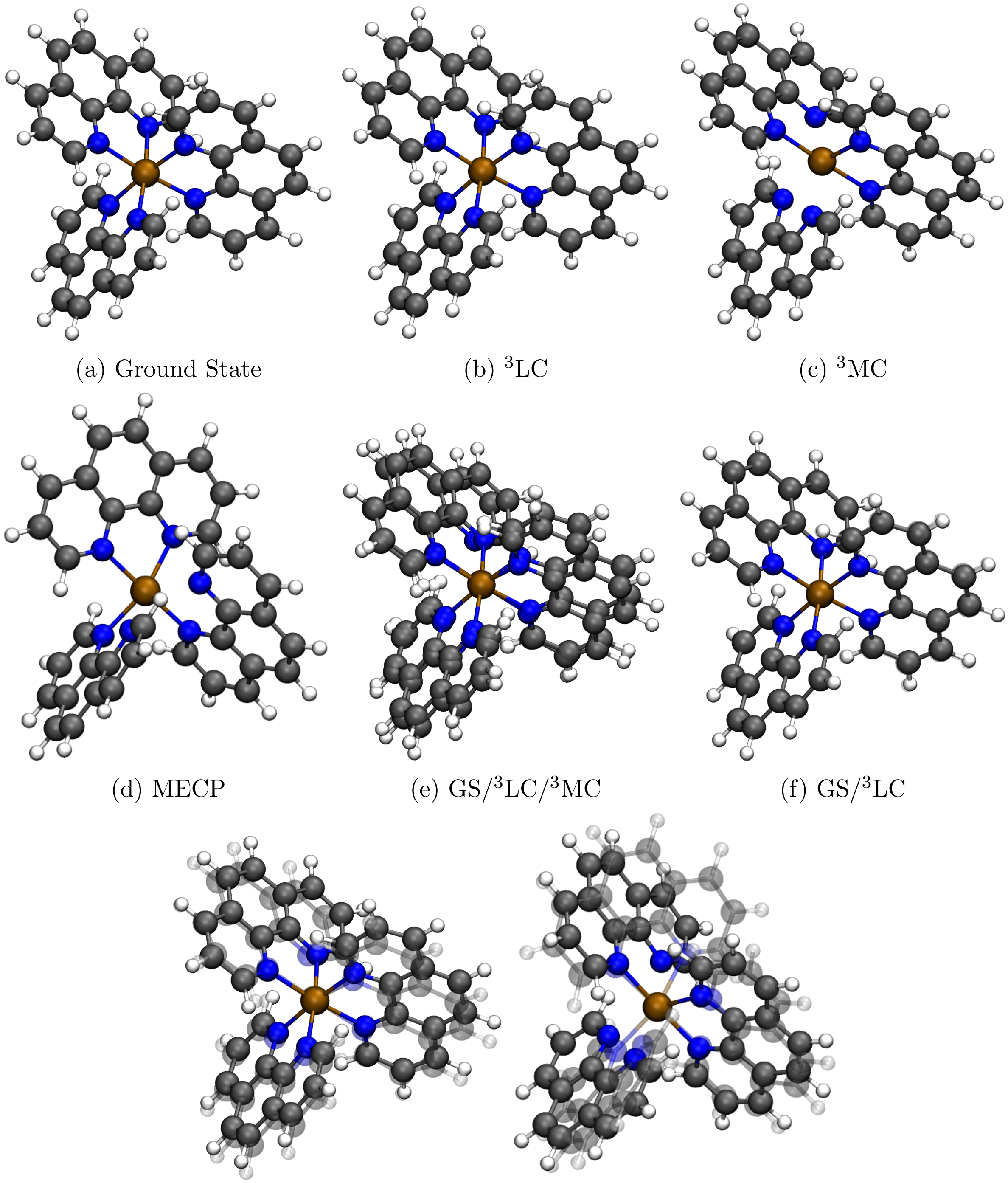

Figure S18: Optimized structures (a−d) and structural overlays (e-h) of **phen3** calculated at the PBE0-D3/LANL2DZ[Ir]+6-311G(d,p)[C,H,N]/PCM(MeCN) level of theory.

Table S10: Structural parameters of optimized stationary points for **phen3** calculated at the PBE0-D3/LANL2DZ[Ir]+6-311G(d,p)[C,H,N]/PCM(MeCN) level of theory.

	Exp.[a]	GS	^{3}LC	^{3}MC	MECP
E (eV)	—	0.00	2.80	3.39	3.33
Spin[b] on Ir	—	—	0.00(0.00)	1.54(1.47)	—
Spin[b] on L1	—	—	0.00	0.19	—
Spin[b] on L2	—	—	2.00	0.11	—
Spin[b] on L3	—	—	0.00	0.16	—
r_1[c] (Å)	2.18	2.06	2.06	2.29	2.07
r_2[c] (Å)	—	2.06	2.05	2.05	2.21
r_3[c] (Å)	—	2.06	2.06	2.16	2.29
$\mathbb{R}$[d] (Å)	—	2.06 ± 0.00	2.05 ± 0.00	2.17 ± 0.06	2.19 ± 0.18
p_1[e] (°)	4.42	0.93	0.84	2.29	1.02
p_2[e] (°)	—	1.01	1.04	1.08	1.15
p_3[e] (°)	—	1.00	0.88	3.75	1.86
$\mathbb{P}$[f] (°)	—	0.98 ± 0.04	0.92 ± 0.10	2.37 ± 1.34	1.34 ± 0.45
$\mathbb{O}$[g] (°)	—	5.98	6.11	8.63	14.41

[a]Measured parameters from Ir(qba)$_2$(phen) crystal by Lee et al.[8] [b]Mulliken spin populations reported, with Löwdin spin populations in parentheses. [c]Average Ir–L distance of one ligand. [d]Average of all Ir–L contacts ± standard deviation. [e]Ligand torsion through bidentate dihedral angle. [f]Average ligand torsion ± standard deviation. [g]Average difference of L–Ir–L angles from 90°.

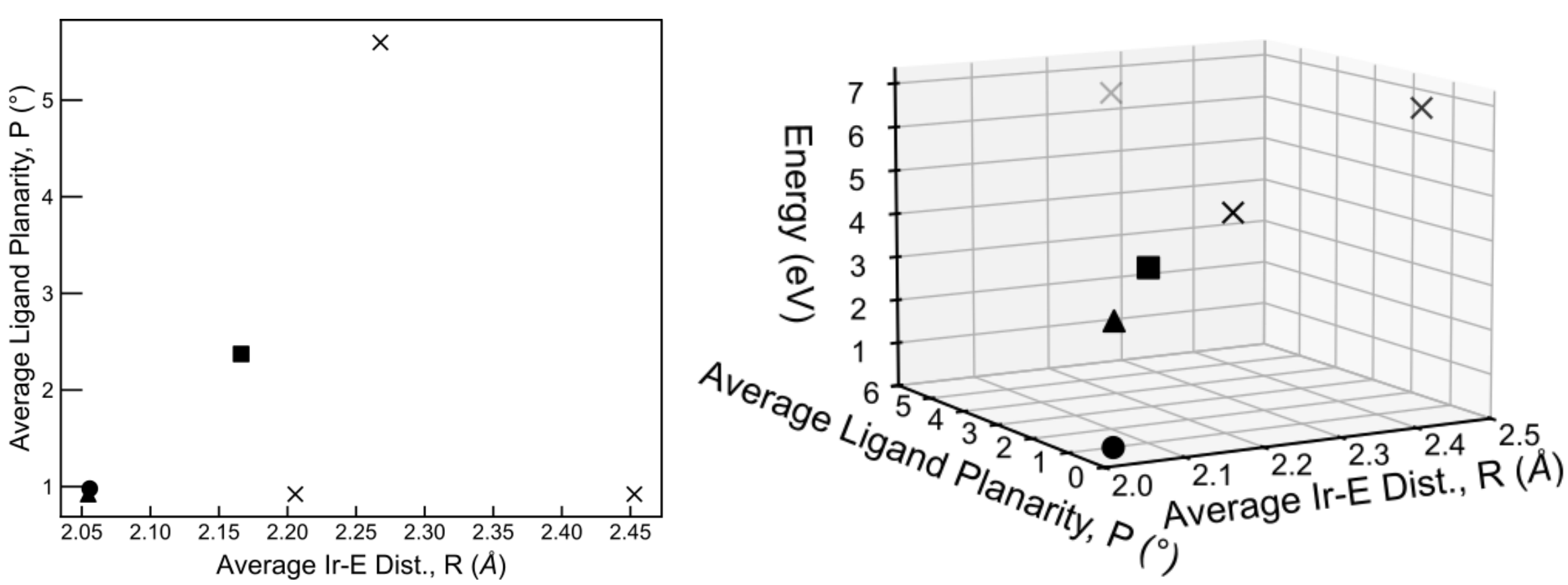

Figure S19: Structure-structure projected surfaces of guess structures surveyed in the search for a **phen3** ^{3}MC minimum. Freely-optimized GS, ^{3}MLCT, and ^{3}MC structures are indicated with a filled circle, triangle, and square respectively. Calculated at the PBE0-D3/LANL2DZ[Ir]+6-311G(d,p)[C,H,N]/PCM(MeCN) level of theory

Four attempts to freely optimize a symmetrized **phen3** structure were made using the same optimization parameters as described in Methods but with various combinations of symmetry keywords; (1) "symmetry=on" from the previously freely-optimized coordinates of the GS structure, (2) "symmetry=on" from a C_3 symmetrized structure built from scratch, (3) "symmetry=(Follow,PG=C3)" from a C_3 symmetrized structure built from scratch, and (4) "symmetry=(PG=C3)" using the input coordinates from attempt (2). All optimization attempts failed to keep C_3 symmetry, many failing to recognize the forced symmetry within any SCF cycles, and ultimately converging to the same structure as was originally freely optimized (GS). Structure (2) was most successful in that the first SCF iteration recognized the full point group as C3 in it's self-consistent minimization (Figure S20), but this calculation also lost symmetry immediately thereafter. Structural parameters indicate that the phen ligands must distort to give a symmetrical distribution of the six N atoms to meet C_3 symmetry with respect to the Ir center. This results in symmetrical bond lengths that are much shorter than the freely-optimized GS ($\mathbb{R} = 1.98 \pm 0.00$ Å) but dihedral distortions greater than even the ^{3}MC structure ($\mathbb{P} = 12.51°$). These short Ir–N distances and extreme distortion result in a structure that does meet C_3 symmetry requirements, but was found to be 1.57 eV higher than the freely-optimized GS structure.

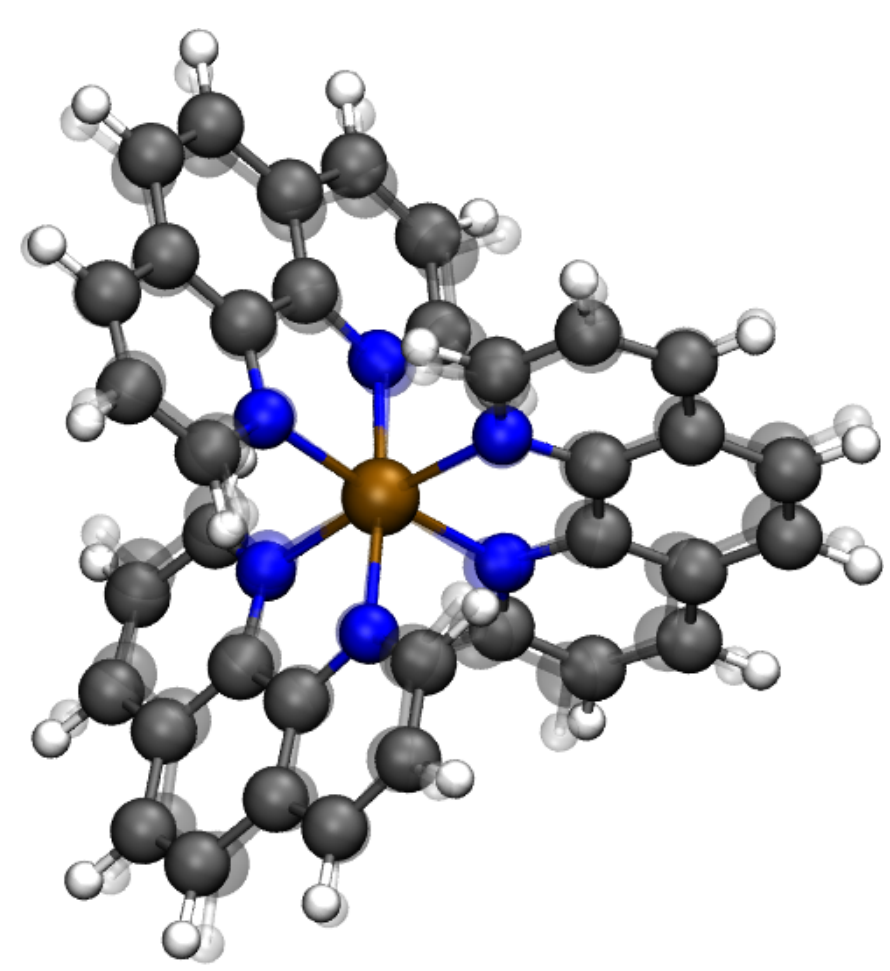

Figure S20: Overlay of freely-optimized **phen3** GS and C_3 symmetrized SCF structure (translucent) calculated at the PBE0-D3/LANL2DZ[Ir]+6-311G(d,p)[C,H,N]/PCM(MeCN) level of theory.

Electronic Structure

Reduced and oxidized doublets were optimized at the same level of theory, and confirmed as minima with frequency calculations. Ground- and excited-state reduction potentials were calculated as

$$E = \frac{-\Delta G}{nF} - E_{ref}$$

where $-\Delta G$ is the difference in electronic energies in J/mol, F is Faraday's constant and n is the number of electrons. As the dominant photoredox function of these complexes is SET, n is taken to be unity. E_{ref} is the half-cell potential of the standard calomel electrode, or 4.429 V in acetonitrile.[9] When available, experimental reduction potentials[10,11] for complexes measured in MeCN vs SCE are reported.

Table S11: Ground- and excited-state reduction potentials vs SCE reference electrode in MeCN reported in eV. Calculated at the PBE0-D3/LANL2DZ[Ir]+6-311G(d,p)[C,H,N]/PCM(MeCN) level of theory. Experimental values listed in parentheses.

	Ground State				Excited State	
	Reduction (eV)		Oxidation (eV)		Reduction (eV)	Oxidation (eV)
***fac*-ppy3**	-2.53	(-2.19)[a]	0.78	(0.77)[a]	0.08	-1.82
***mer*-ppy3**	-2.48		0.51		-0.09	-1.88
***m*-ppy2**	-1.46	(-1.38)[b]	1.27	(1.27)[b]	0.88	-1.07
***f*-ppy2**	-1.44		1.40		0.87	-0.91
**bpy2**	-1.09		2.08		1.58	-0.59
**bpy3**	-0.72	(-1.1)[a]	3.54	(2.10)[a]	2.11	0.71
***fac*-bzq3**	-2.28		0.80		0.22	-1.71
***mer*-bzq3**	-2.27		0.56		0.16	-1.88
**phen3**	-0.81		3.20		1.99	0.40

[a]Experimental values recorded in MeCN by Flamigni et al.[10] [b]Experimental values recorded in MeCN by Ladouceur at al.[11]

Exchanging the coordinating atom on the polypyridyl ligands from a carbon to a nitrogen results in an increasing overall charge on the complex. Such an increase in charge results in decreasing absolute energy of each molecular orbital. The character of the density of each molecular orbital is unaffected with increasing charge, but rather is altered more significantly by breaking C_3 symmetry (Figures S22−S30). Though a discussion of overall charge and heteroatom substitution cannot be entirely segregated, these complexes are all d^6 low-spin Ir(III) complexes and therefore changes in character of density or excitation can more strongly be attributed to changes in structure (Figures S22−S30).

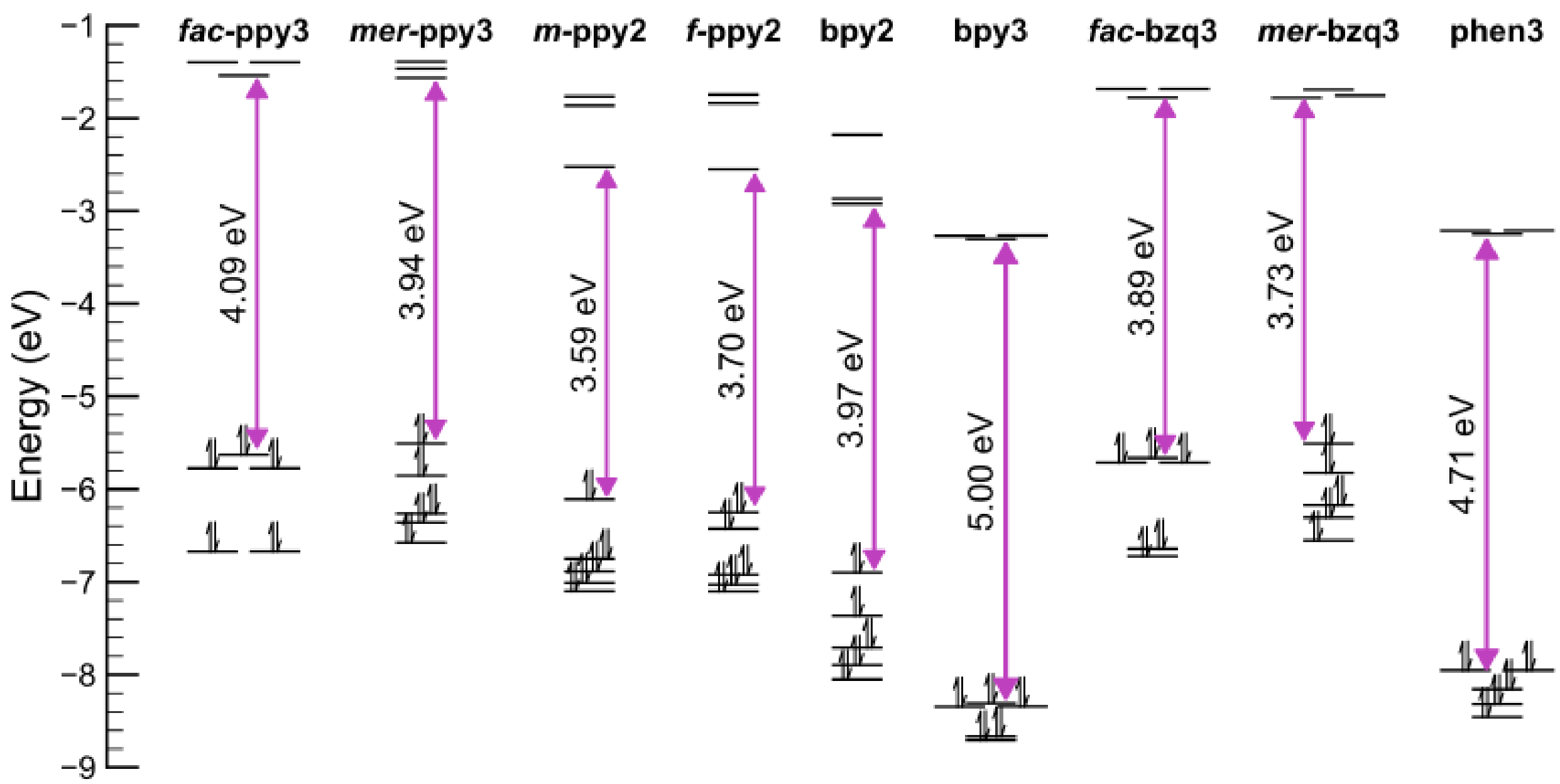

Figure S21: Frontier orbitals of each GS complex with HOMO-LUMO gap indicated. Calculated at the PBE0-D3/LANL2DZ[Ir]+6-311G(d,p)[C,H,N]/PCM(MeCN) level of theory.

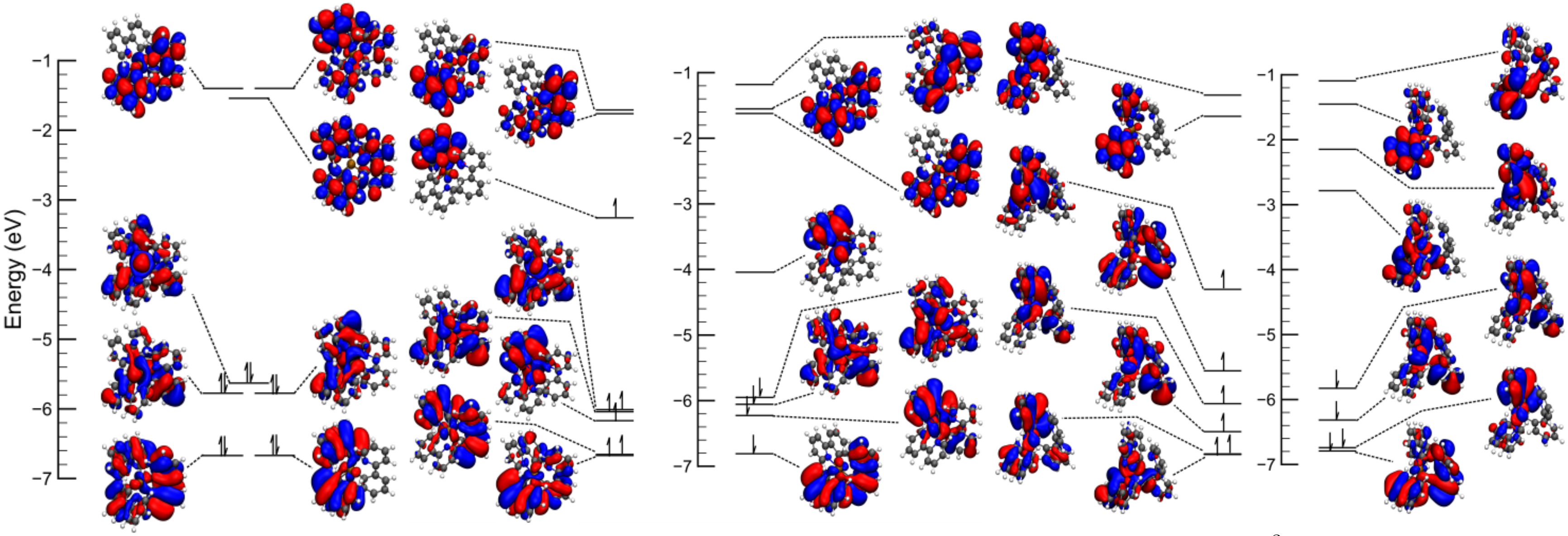

(a) GS

(b) ^{3}MLCT

(c) ^{3}MC

Figure S22: Molecular orbital diagrams of ***fac*-ppy3** calculated at the PBE0-D3/LANL2DZ[Ir]+6-311G(d,p)[C,H,N]/PCM(MeCN) level of theory (Isolvalue ± 0.02).

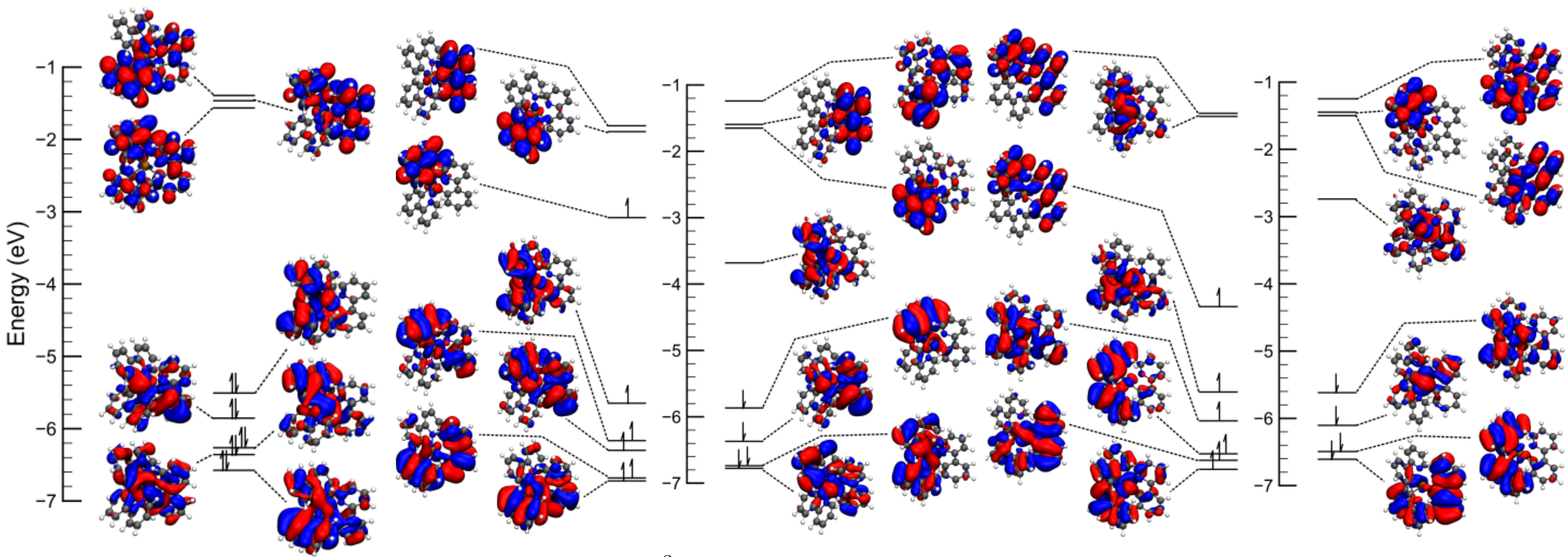

(a) GS (b) ^{3}MLCT (c) ^{3}MC

Figure S23: Molecular orbital diagrams of ***mer*-ppy3** calculated at the PBE0-D3/LANL2DZ[Ir]+6-311G(d,p)[C,H,N]/PCM(MeCN) level of theory (Isolvalue ± 0.02).

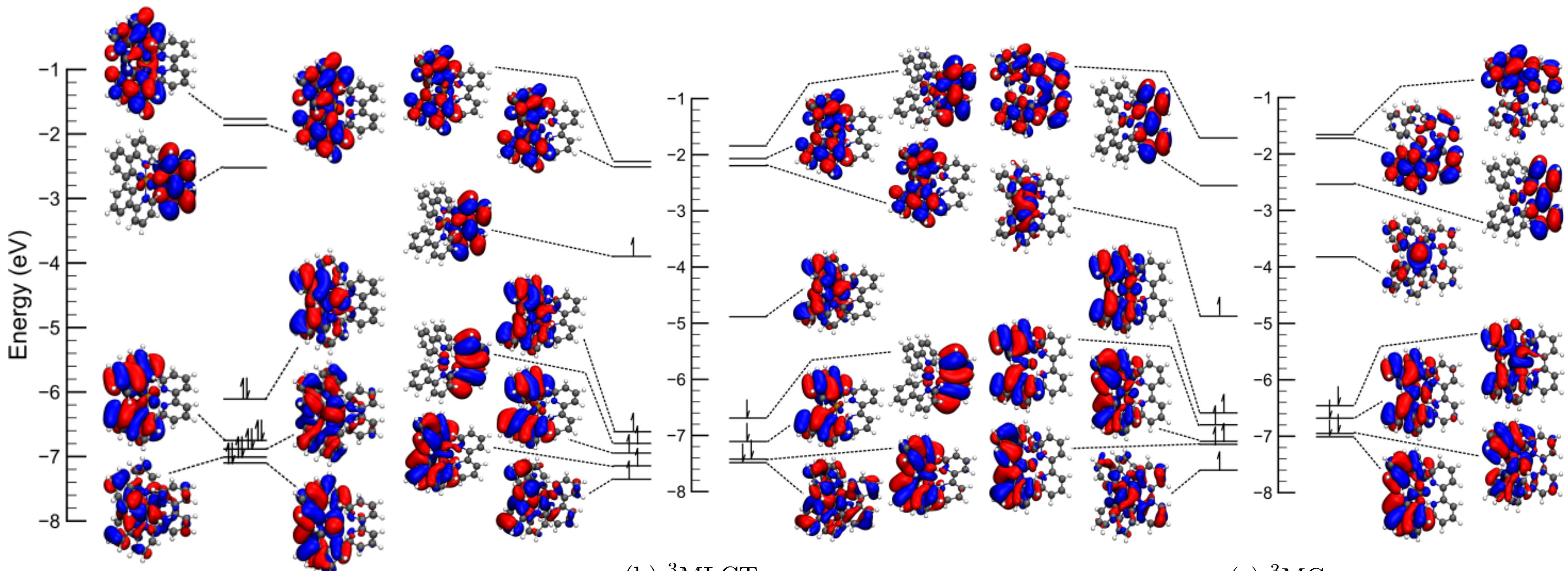

(a) GS (b) ^{3}MLCT (c) ^{3}MC

Figure S24: Molecular orbital diagrams of ***m*-ppy2** calculated at the PBE0-D3/LANL2DZ[Ir]+6-311G(d,p)[C,H,N]/PCM(MeCN) level of theory (Isolvalue ± 0.02).

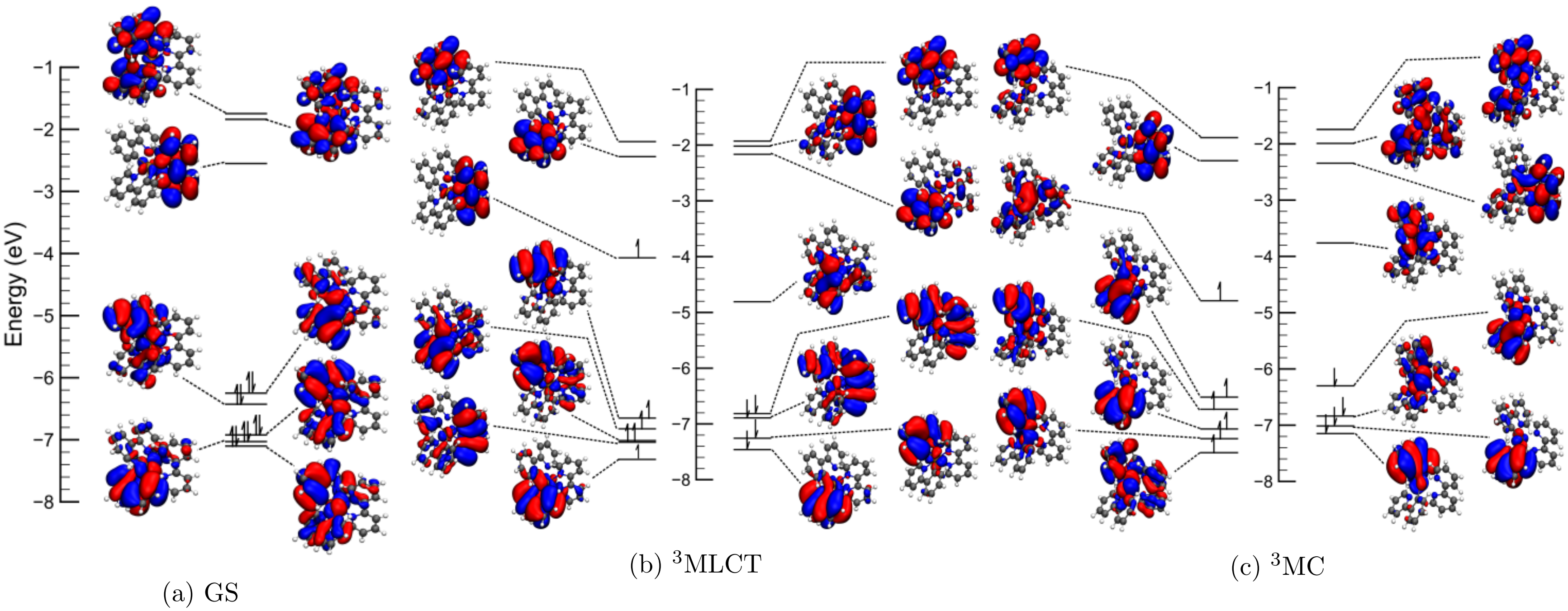

Figure S25: Molecular orbital diagrams of ***f*-ppy2** calculated at the PBE0-D3/LANL2DZ[Ir]+6-311G(d,p)[C,H,N]/PCM(MeCN) level of theory (Isolvalue ± 0.02).

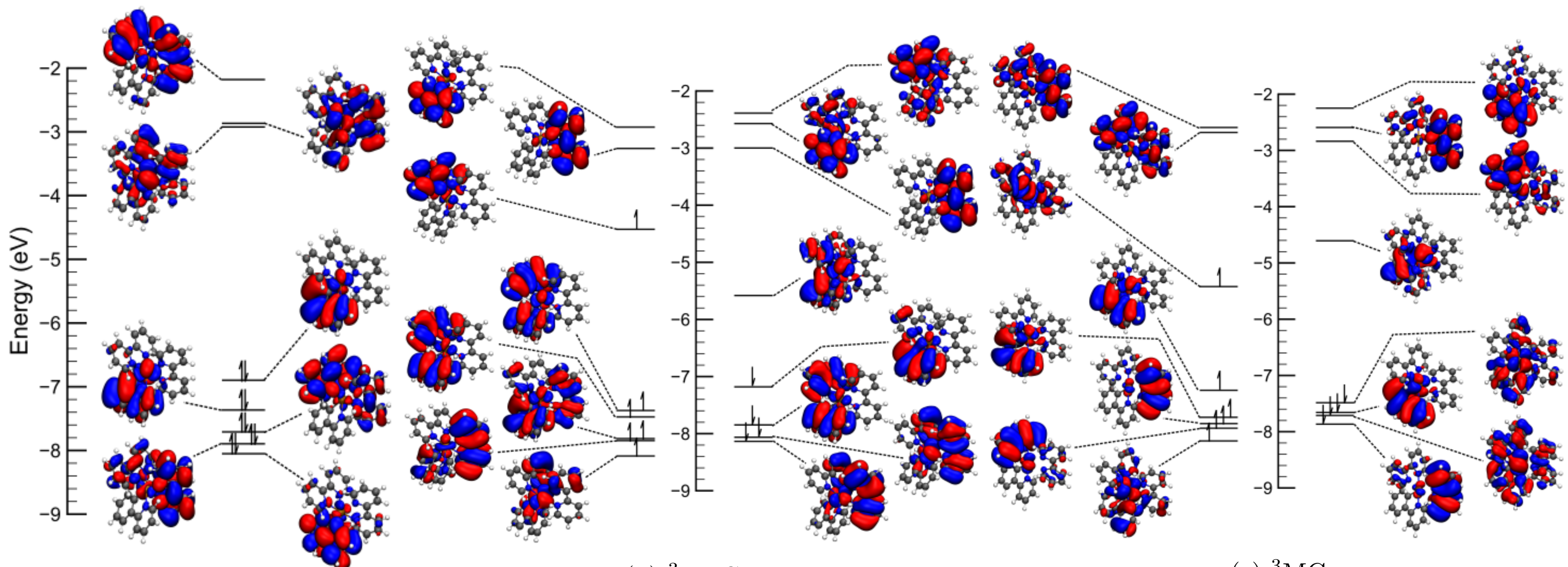

(a) GS

(b) ^{3}MLCT

(c) ^{3}MC

Figure S26: Molecular orbital diagrams of **bpy2** calculated at the PBE0-D3/LANL2DZ[Ir]+6-311G(d,p)[C,H,N]/PCM(MeCN) level of theory (Isolvalue ± 0.02).

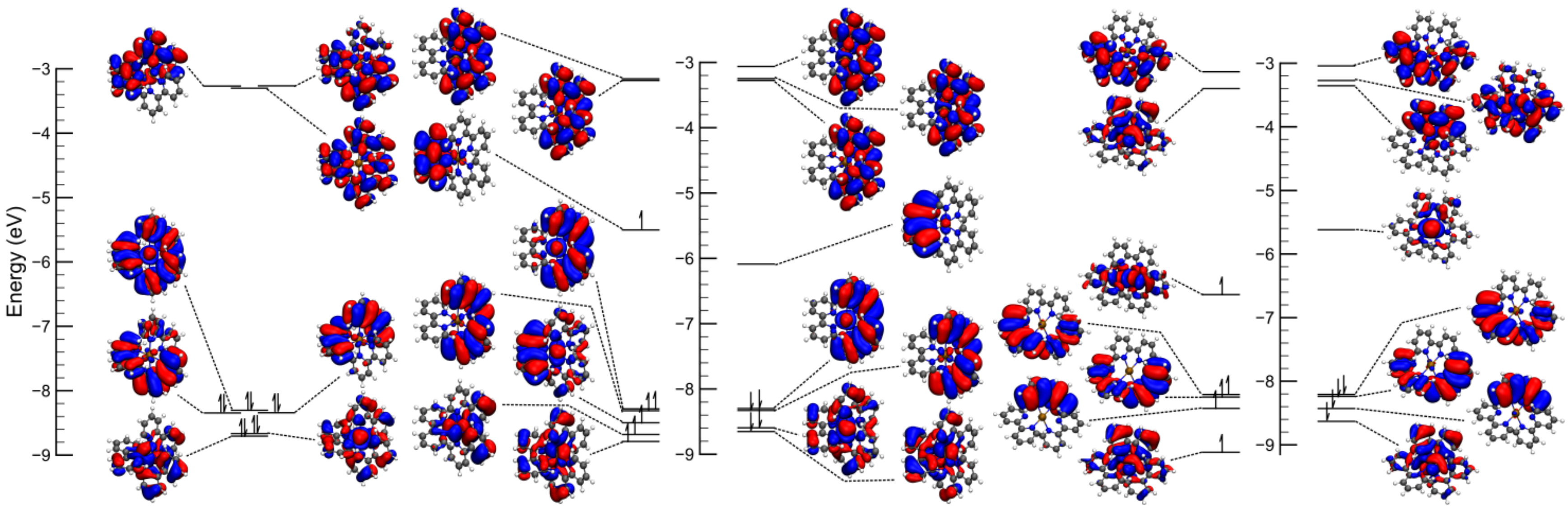

(a) GS (b) ^{3}LC (c) ^{3}MC

Figure S27: Molecular orbital diagrams of **bpy3** calculated at the PBE0-D3/LANL2DZ[Ir]+6-311G(d,p)[C,H,N]/PCM(MeCN) level of theory (Isolvalue ± 0.02).

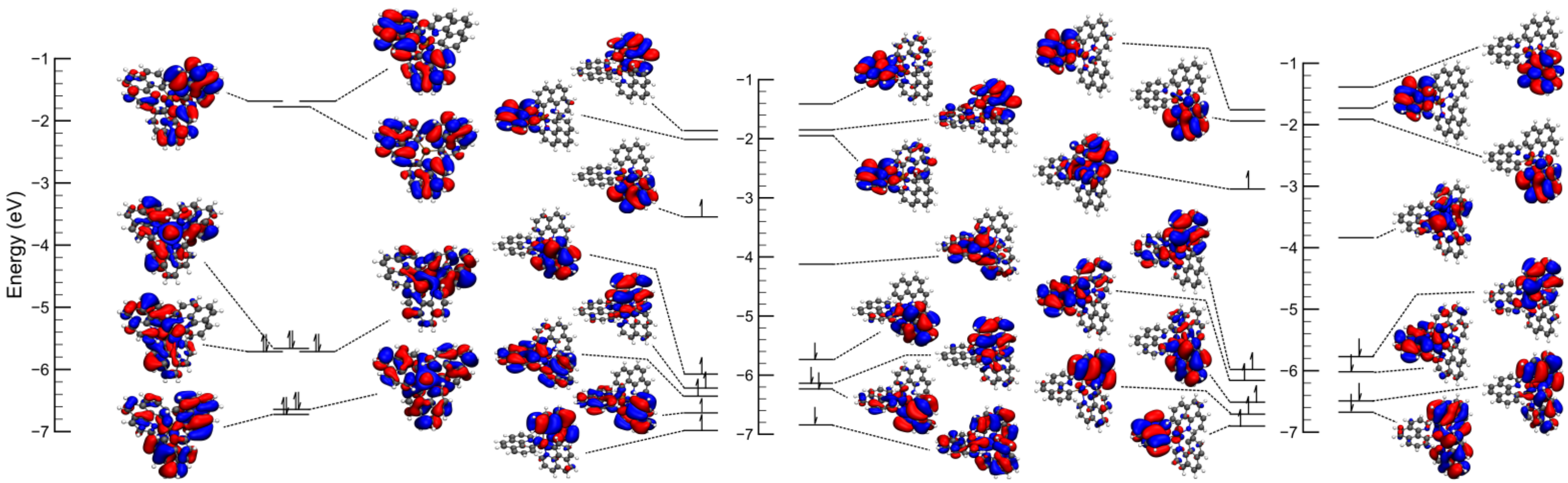

(a) GS (b) ^{3}MLCT (c) ^{3}MC

Figure S28: Molecular orbital diagrams of ***fac*-bzq3** calculated at the PBE0-D3/LANL2DZ[Ir]+6-311G(d,p)[C,H,N]/PCM(MeCN) level of theory (Isolvalue ± 0.02).

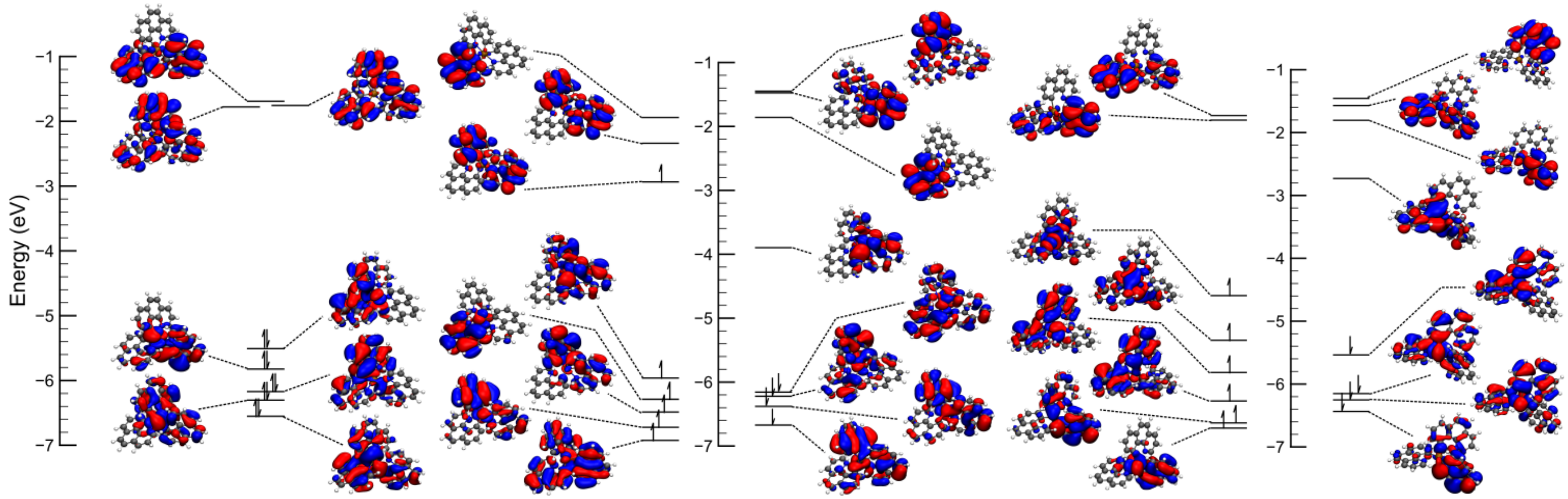

(a) GS (b) ^{3}MLCT (c) ^{3}MC

Figure S29: Molecular orbital diagrams of ***mer*-bzq3** calculated at the PBE0-D3/LANL2DZ[Ir]+6-311G(d,p)[C,H,N]/PCM(MeCN) level of theory (Isolvalue ± 0.02).

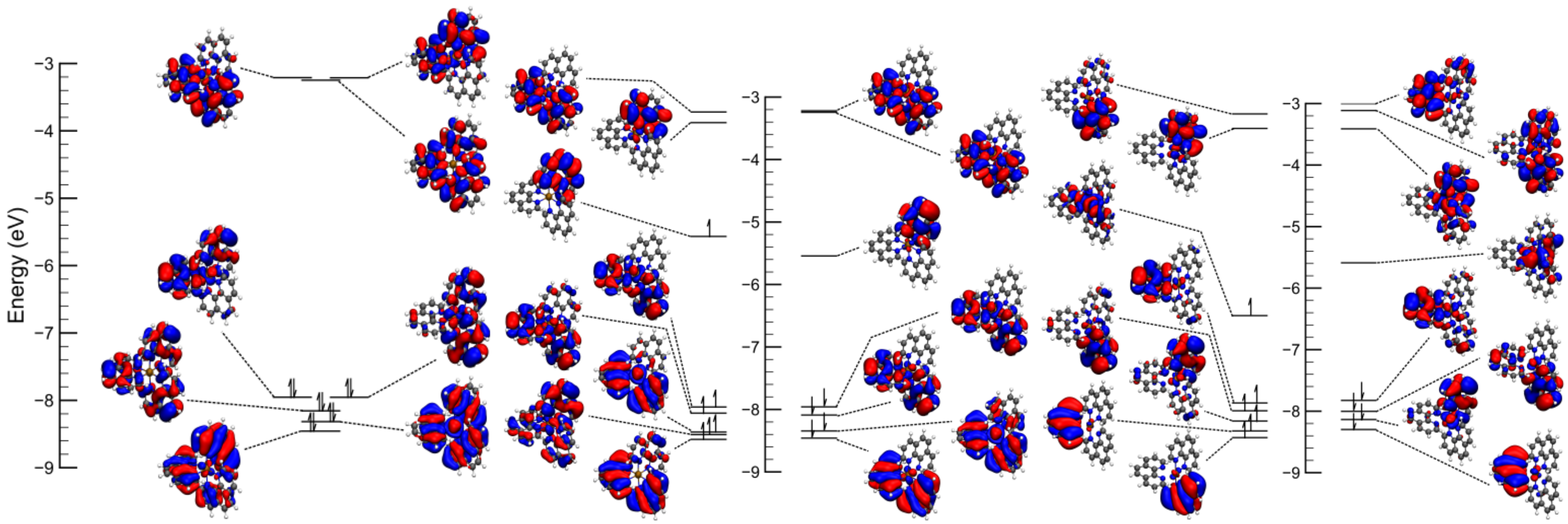

(a) GS (b) ^{3}LC (c) ^{3}MC

Figure S30: Molecular orbital diagrams of **phen3** calculated at the PBE0-D3/LANL2DZ[Ir]+6-311G(d,p)[C,H,N]/PCM(MeCN) level of theory (Isolvalue ± 0.02).

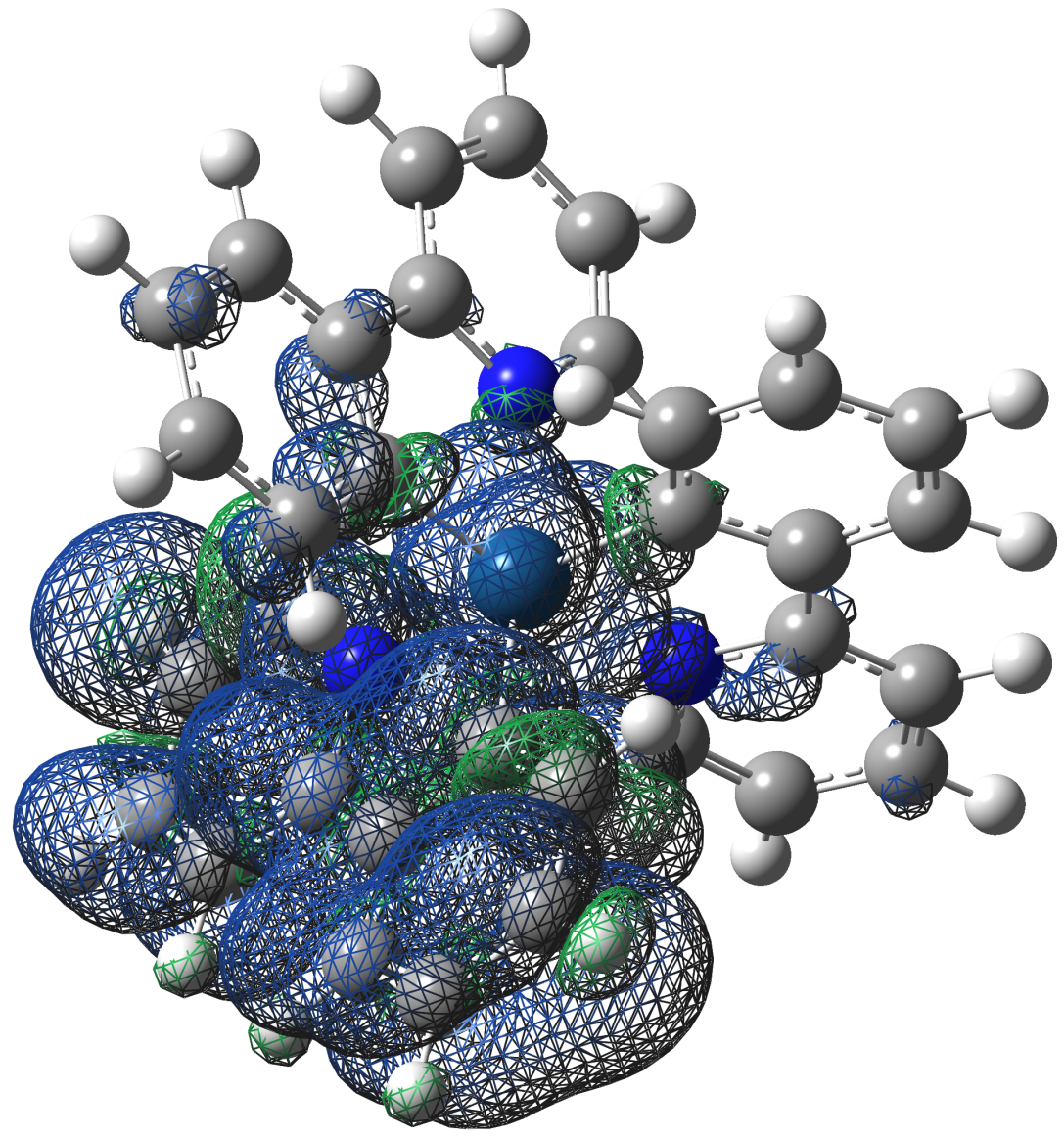

(a) ^{3}MLCT, Ir spin = 0.40

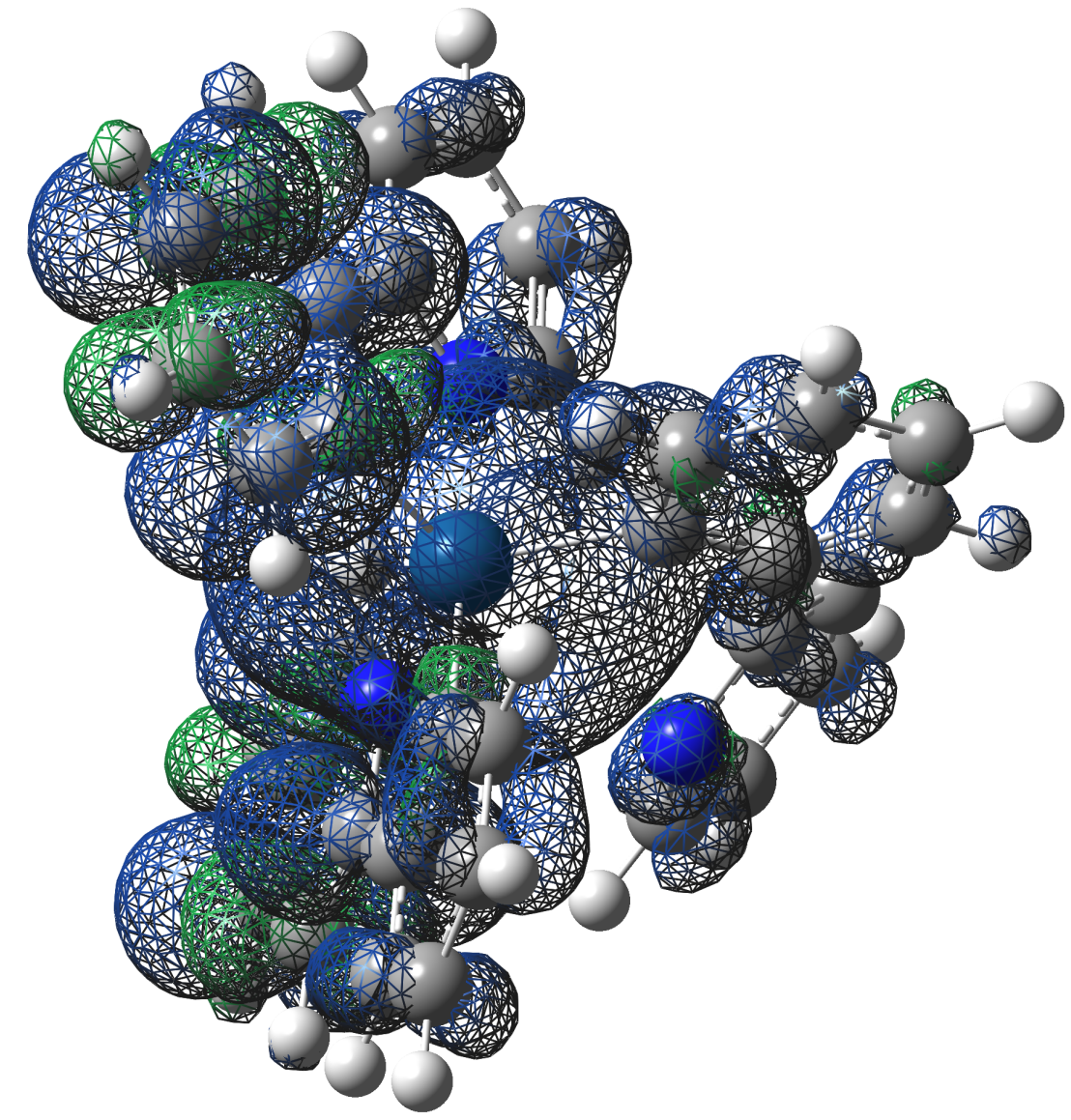

(b) ^{3}MC, Ir spin = 1.08

Figure S31: Spin density difference orbitals of ***fac*-ppy3** ^{3}MLCT and ^{3}MC structures calculated at the PBE0-D3/LANL2DZ[Ir]+6-311G(d,p)[C,H,N]/PCM(MeCN) level of theory (Isovalue ± 0.004).

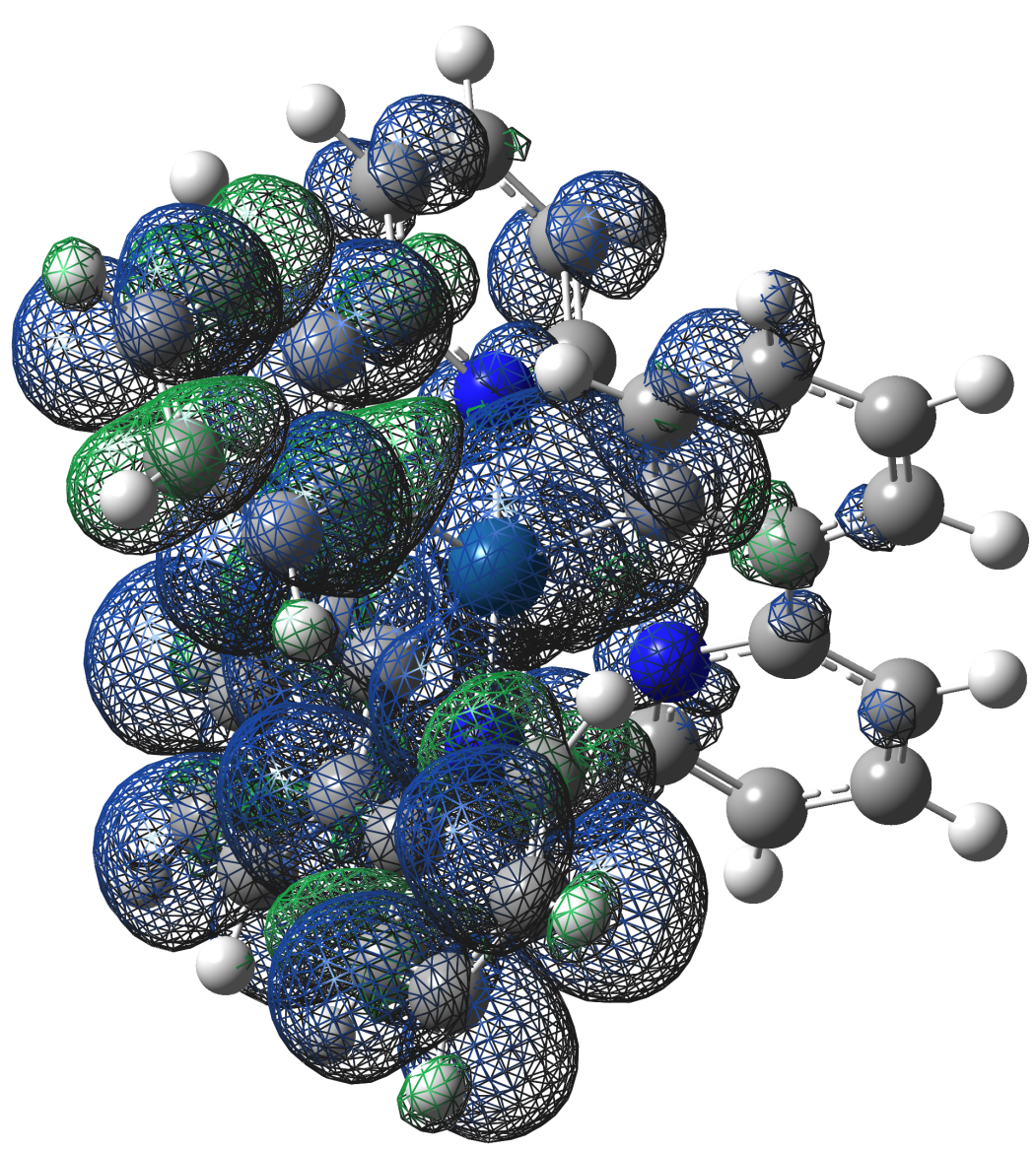

(a) ^{3}MLCT, Ir spin = 0.42

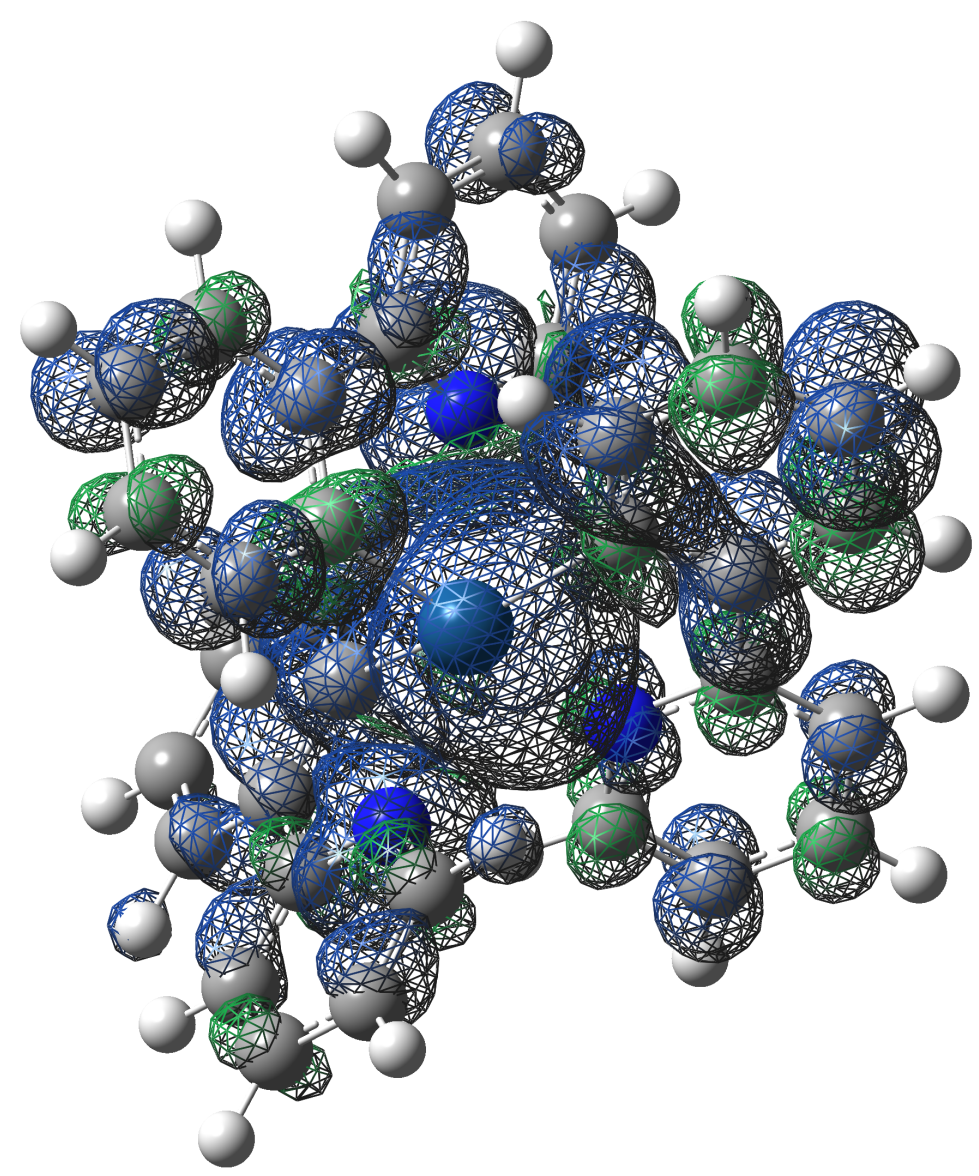

(b) ^{3}MC, Ir spin = 1.38

Figure S32: Spin density difference orbitals of ***mer*-ppy3** ^{3}MLCT and ^{3}MC structures calculated at the PBE0-D3/LANL2DZ[Ir]+6-311G(d,p)[C,H,N]/PCM(MeCN) level of theory (Isovalue ± 0.004).

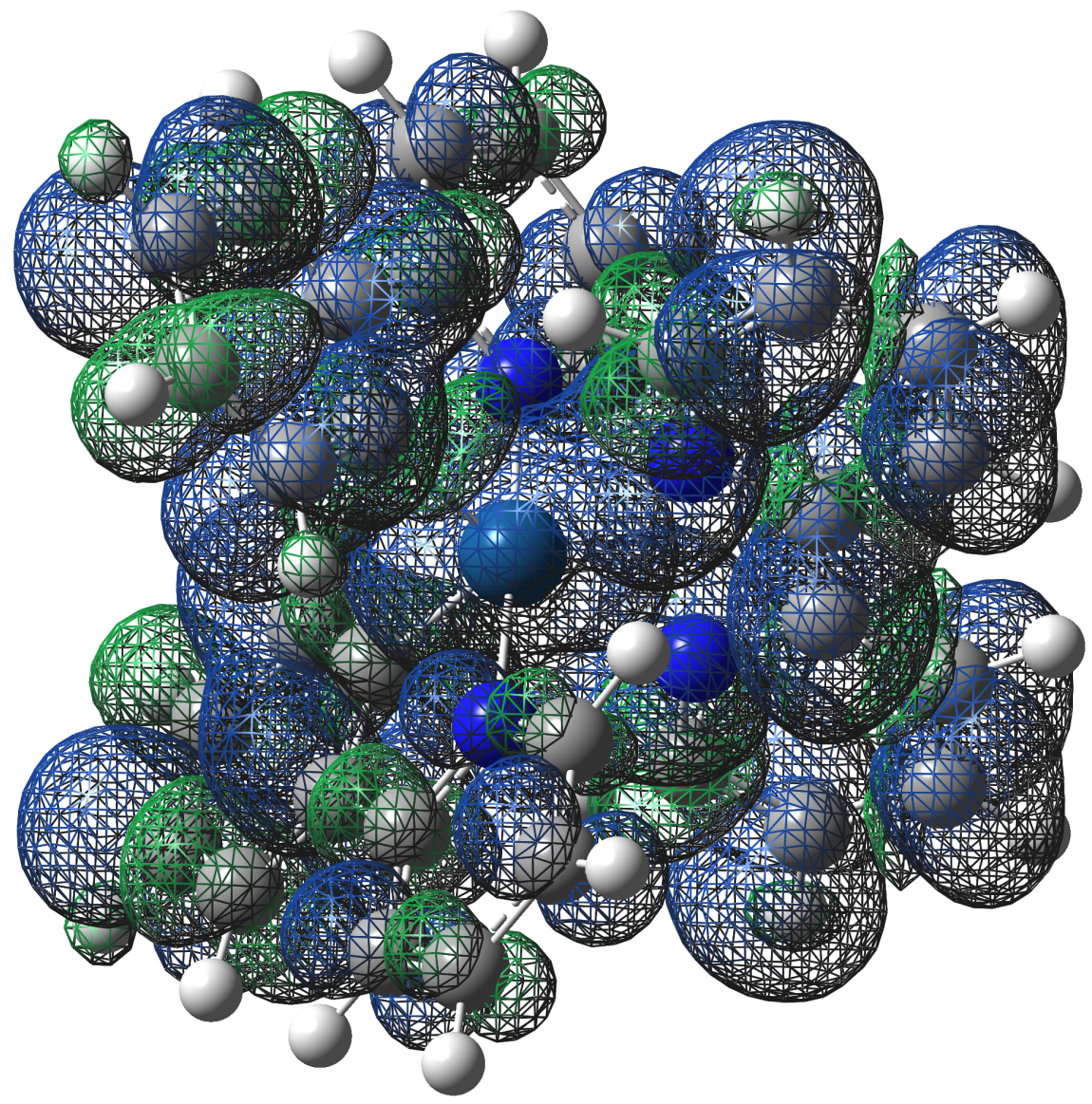

(a) ^{3}MLCT, Ir spin = 0.54

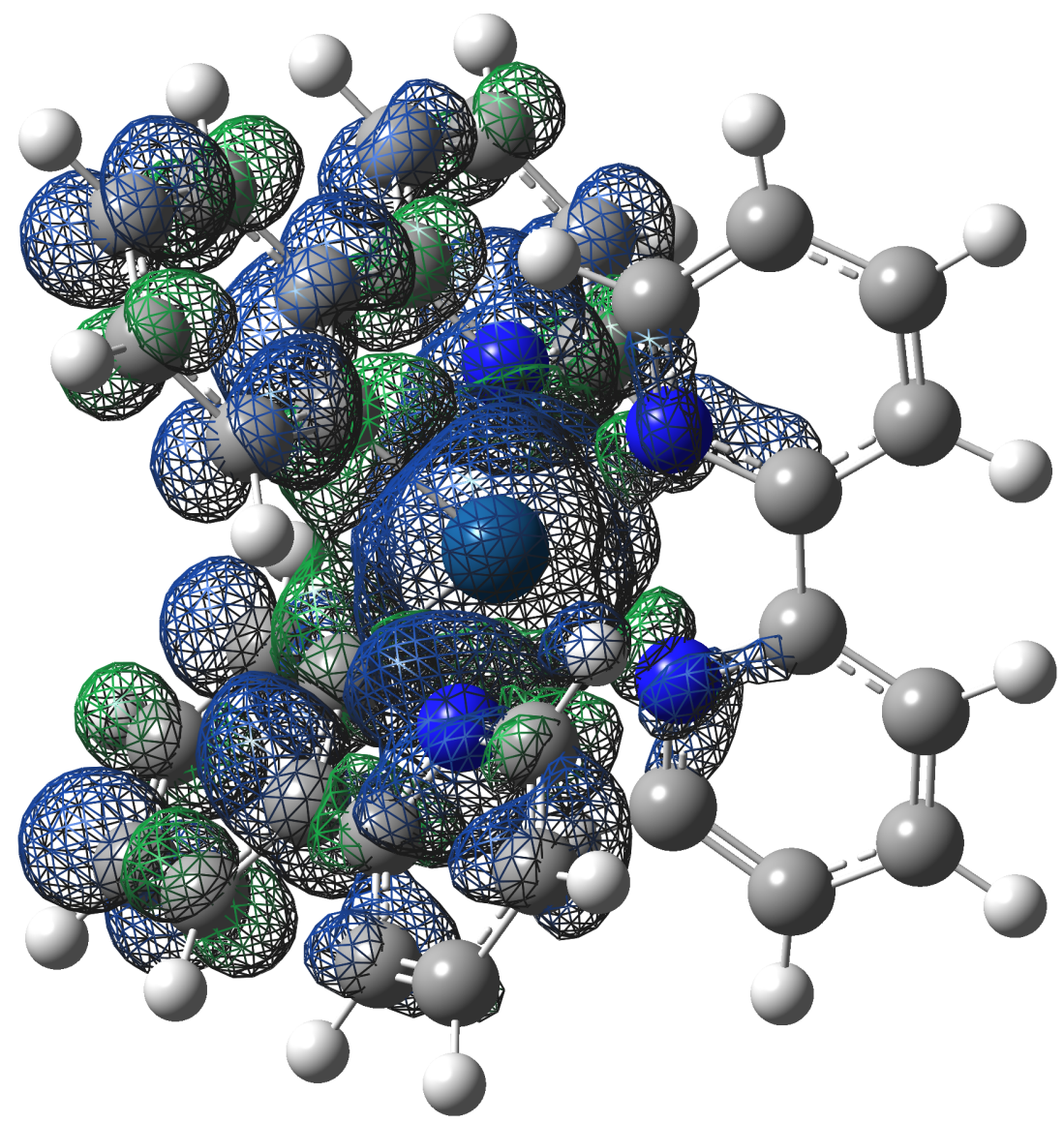

(b) ^{3}MC, Ir spin = 1.64

Figure S33: Spin density difference orbitals of ***m*-ppy2** ^{3}MLCT and ^{3}MC structures calculated at the PBE0-D3/LANL2DZ[Ir]+6-311G(d,p)[C,H,N]/PCM(MeCN) level of theory (Isovalue ± 0.004).

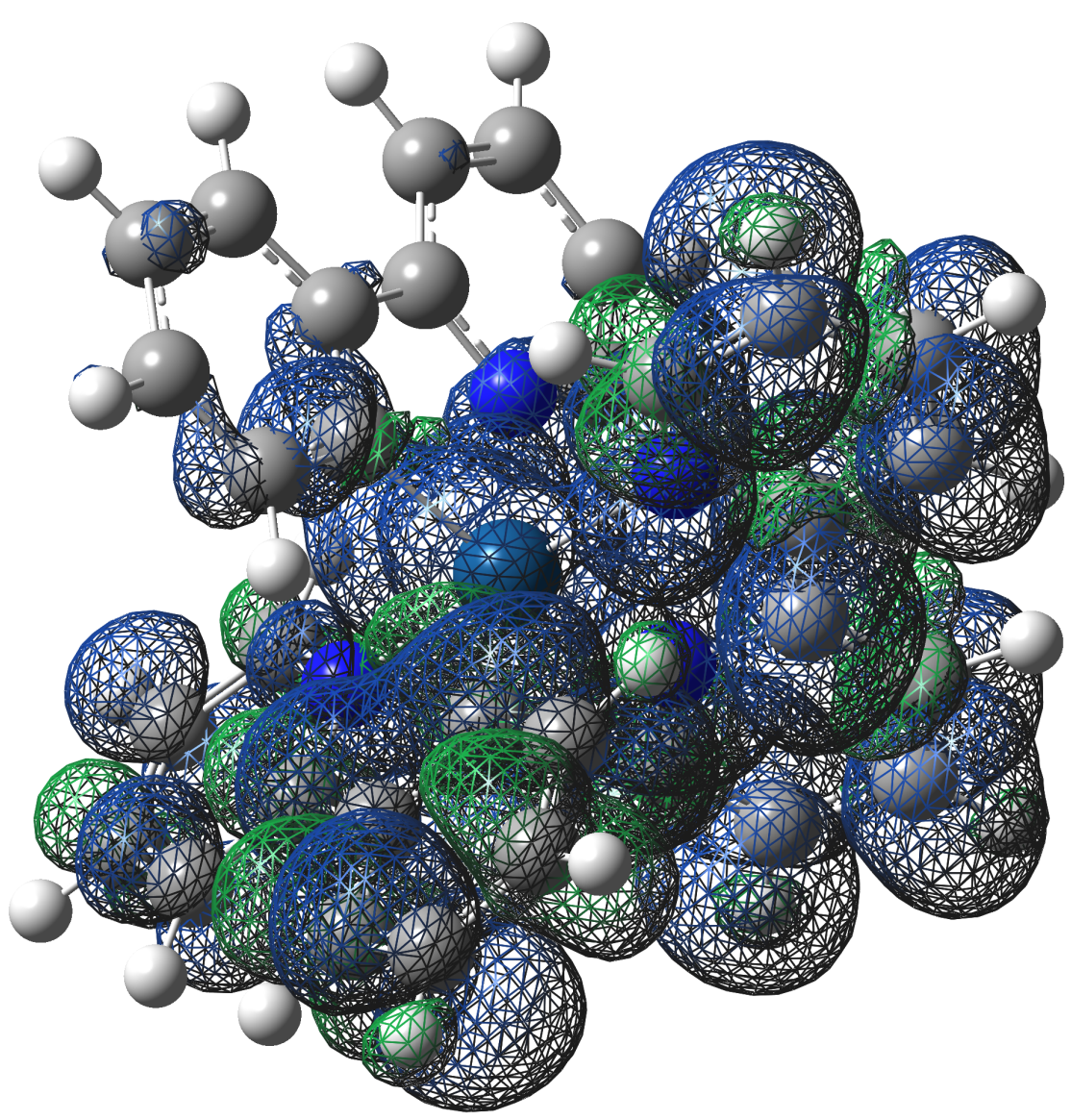

(a) ^{3}MLCT, Ir spin = 0.57

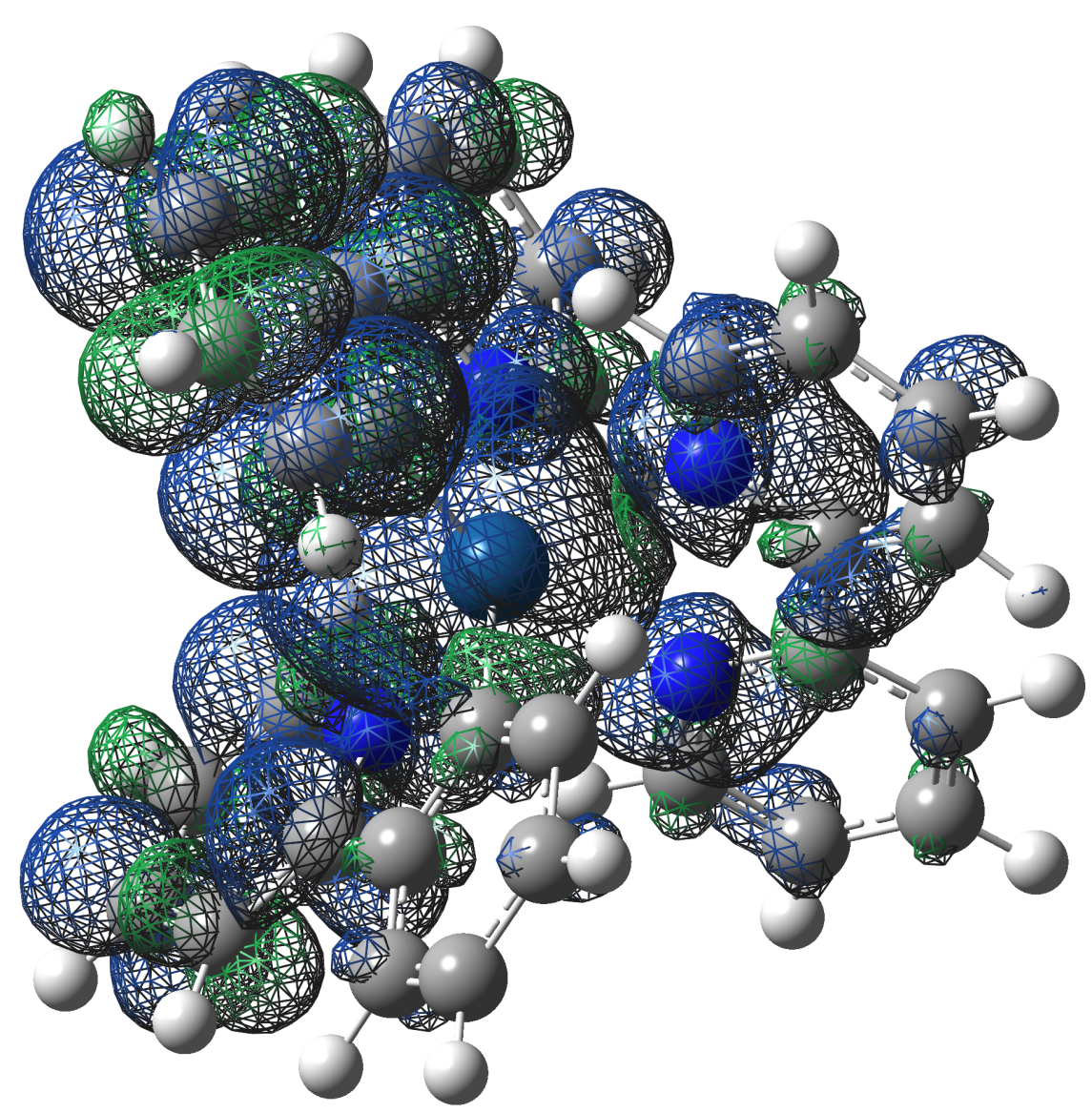

(b) ^{3}MC, Ir spin = 1.39

Figure S34: Spin density difference orbitals of ***f*-ppy2** ^{3}MLCT and ^{3}MC structures calculated at the PBE0-D3/LANL2DZ[Ir]+6-311G(d,p)[C,H,N]/PCM(MeCN) level of theory (Isovalue ± 0.004).

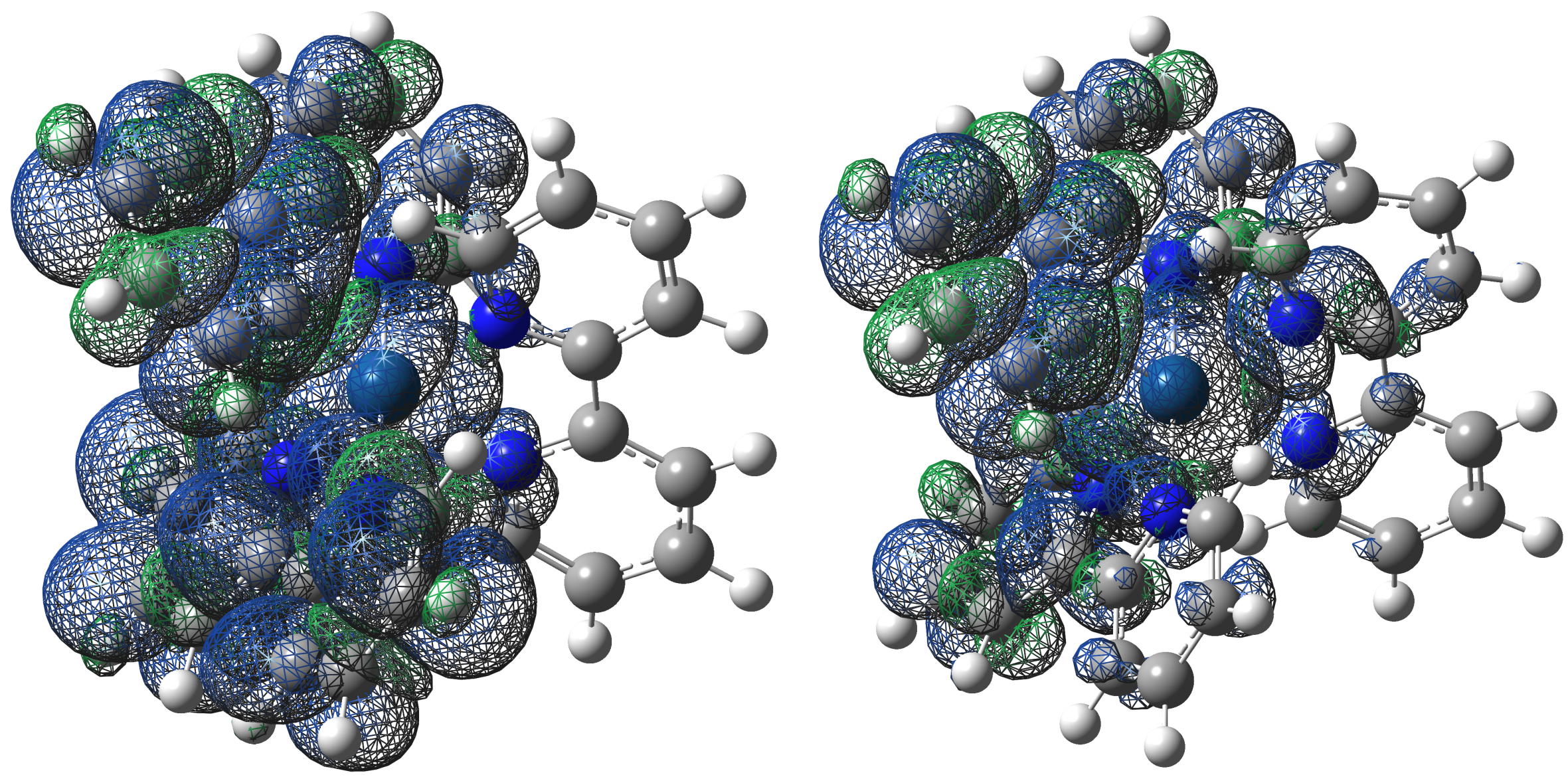

(a) ^{3}MLCT, Ir spin = 0.45

(b) ^{3}MC, Ir spin = 1.34

Figure S35: Spin density difference orbitals of **bpy2** ^{3}MLCT and ^{3}MC structures calculated at the PBE0-D3/LANL2DZ[Ir]+6-311G(d,p)[C,H,N]/PCM(MeCN) level of theory (Isovalue ± 0.004).

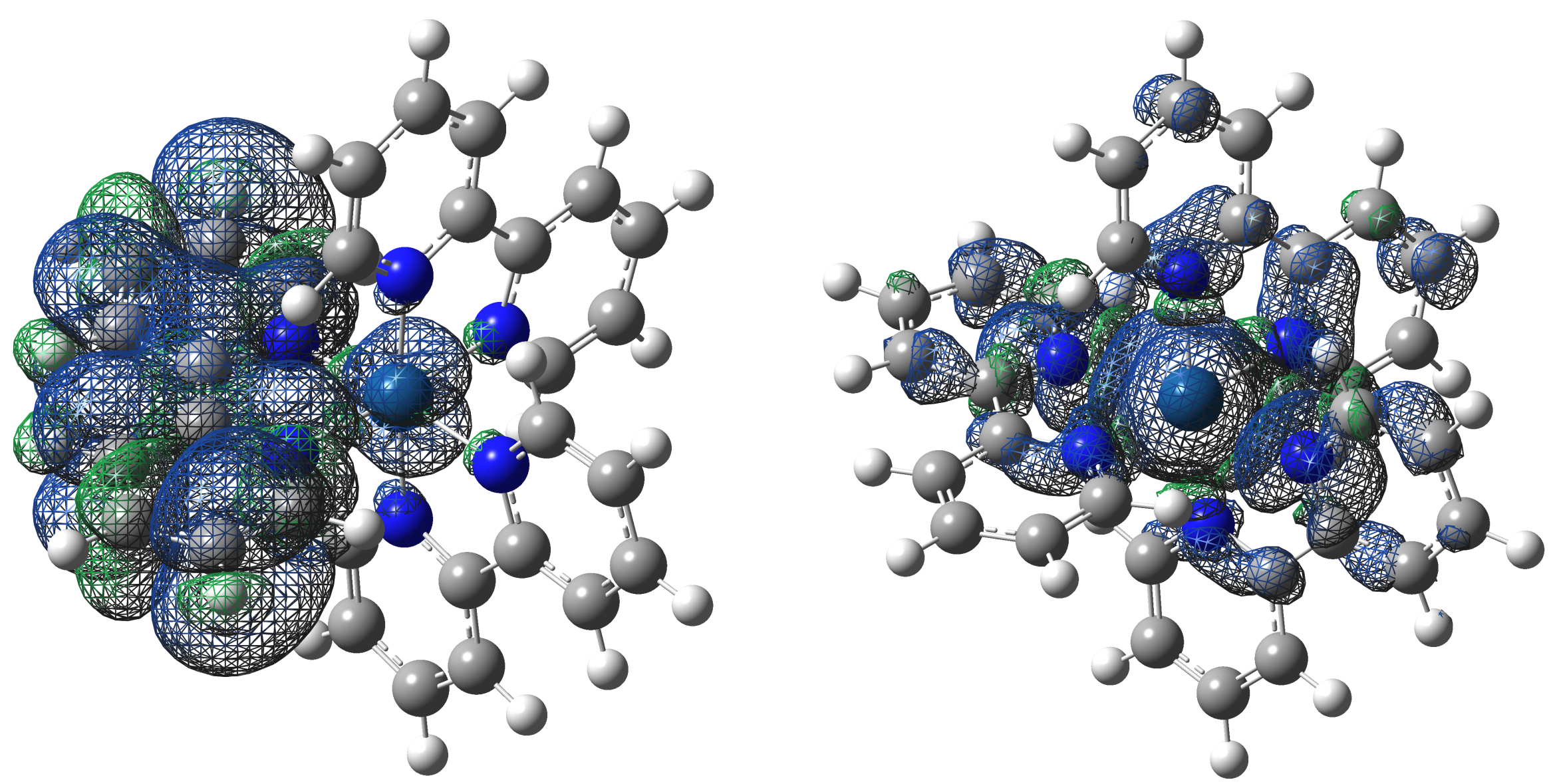

(a) ^{3}LC, Ir spin = 0.03

(b) ^{3}MC, Ir spin = 1.66

Figure S36: Spin density difference orbitals of **bpy3** ^{3}LC and ^{3}MC structures calculated at the PBE0-D3/LANL2DZ[Ir]+6-311G(d,p)[C,H,N]/PCM(MeCN) level of theory (Isovalue ± 0.004).

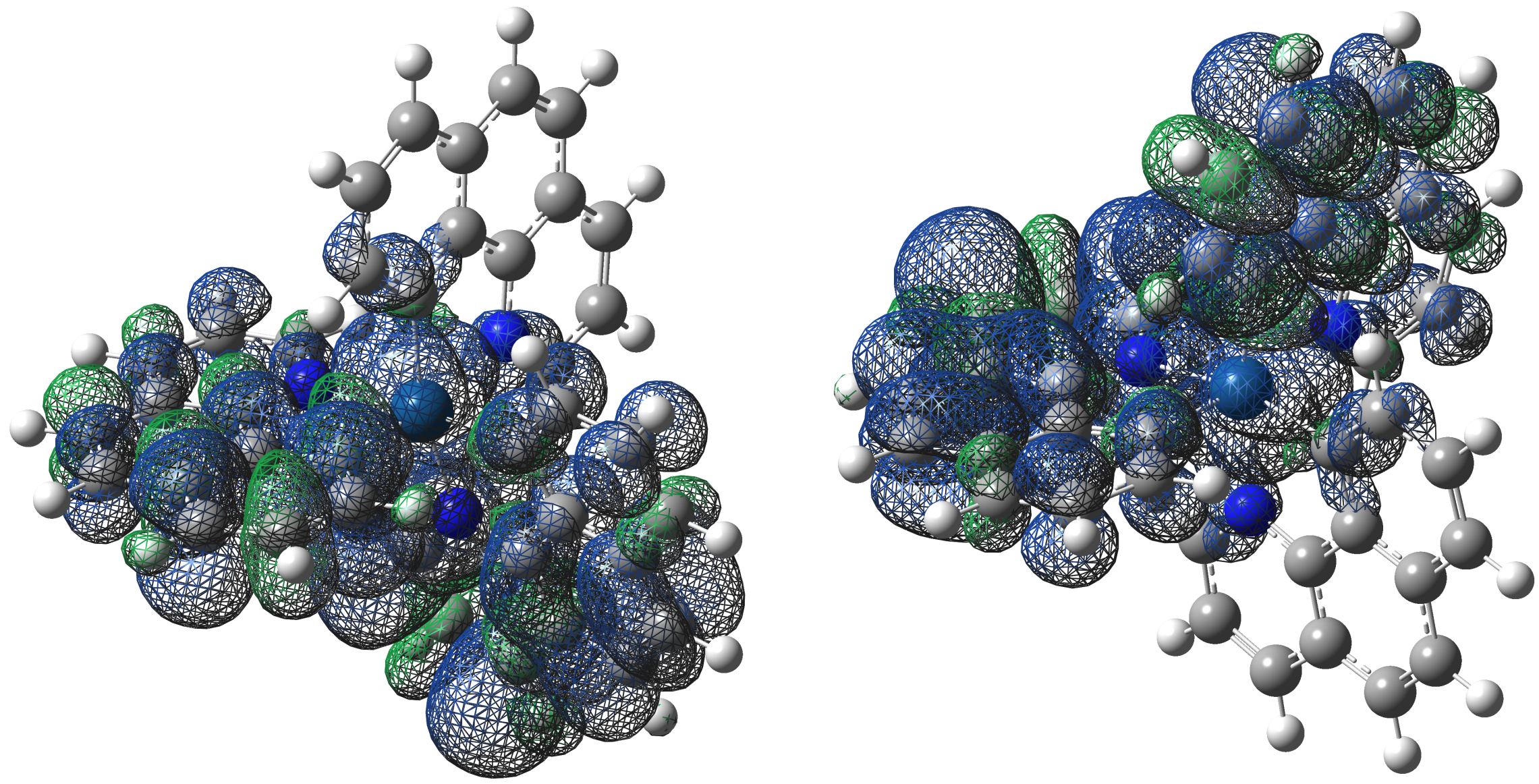

(a) ^{3}MLCT, Ir spin = 0.57 (b) ^{3}MC*, Ir spin = 1.11

Figure S37: Spin density difference orbitals of ***fac*-bzq3** ^{3}MLCT and ^{3}MC structures calculated at the PBE0-D3/LANL2DZ[Ir]+6-311G(d,p)[C,H,N]/PCM(MeCN) level of theory (Isovalue ± 0.004). *Lowest-energy ^{3}MC. See Methods.

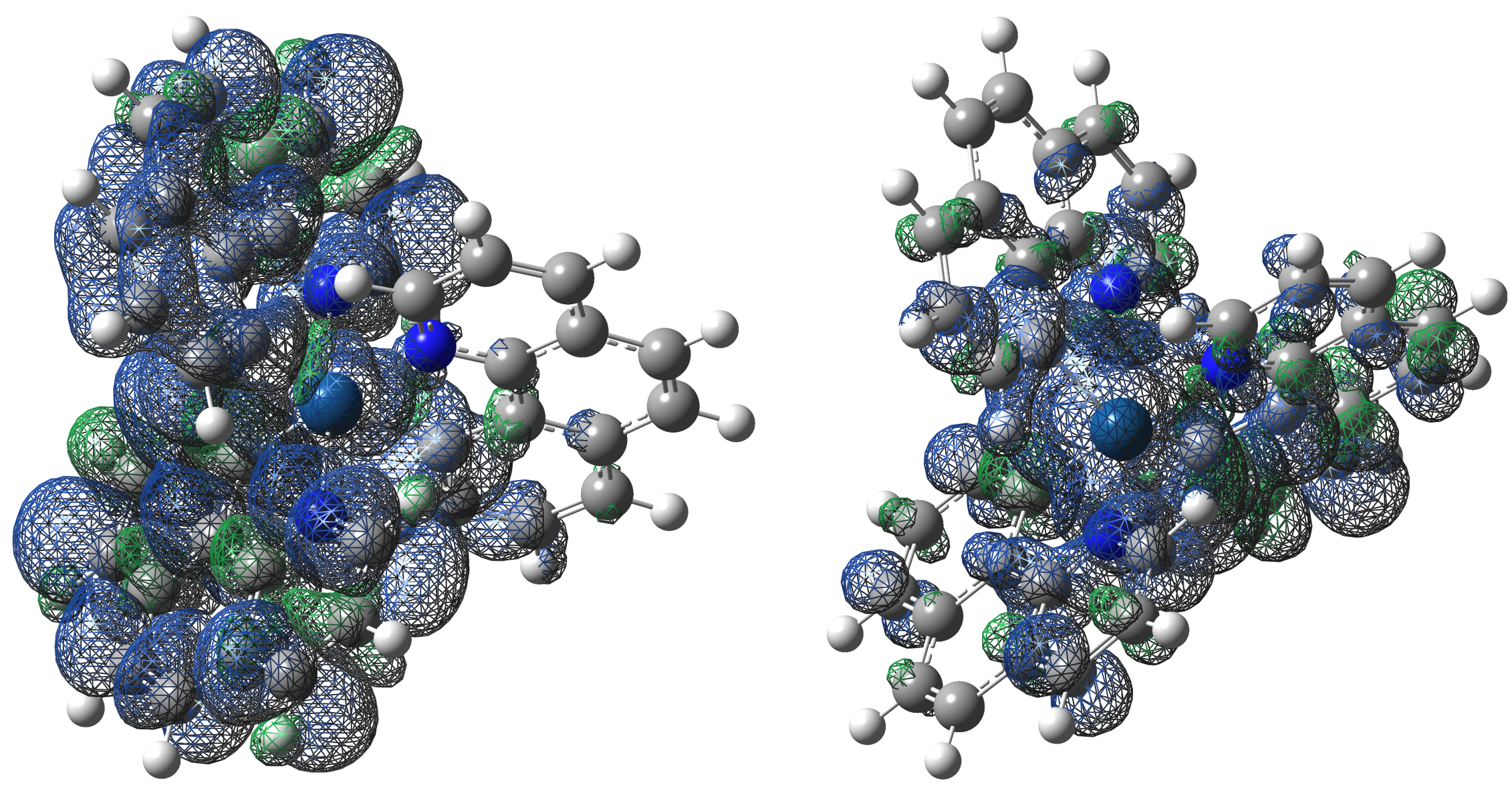

(a) ^{3}MLCT, Ir spin = 0.40 (b) ^{3}MC, Ir spin = 1.30

Figure S38: Spin density difference orbitals of ***mer*-bzq3** ^{3}MLCT and ^{3}MC structures calculated at the PBE0-D3/LANL2DZ[Ir]+6-311G(d,p)[C,H,N]/PCM(MeCN) level of theory (Isovalue ± 0.004).

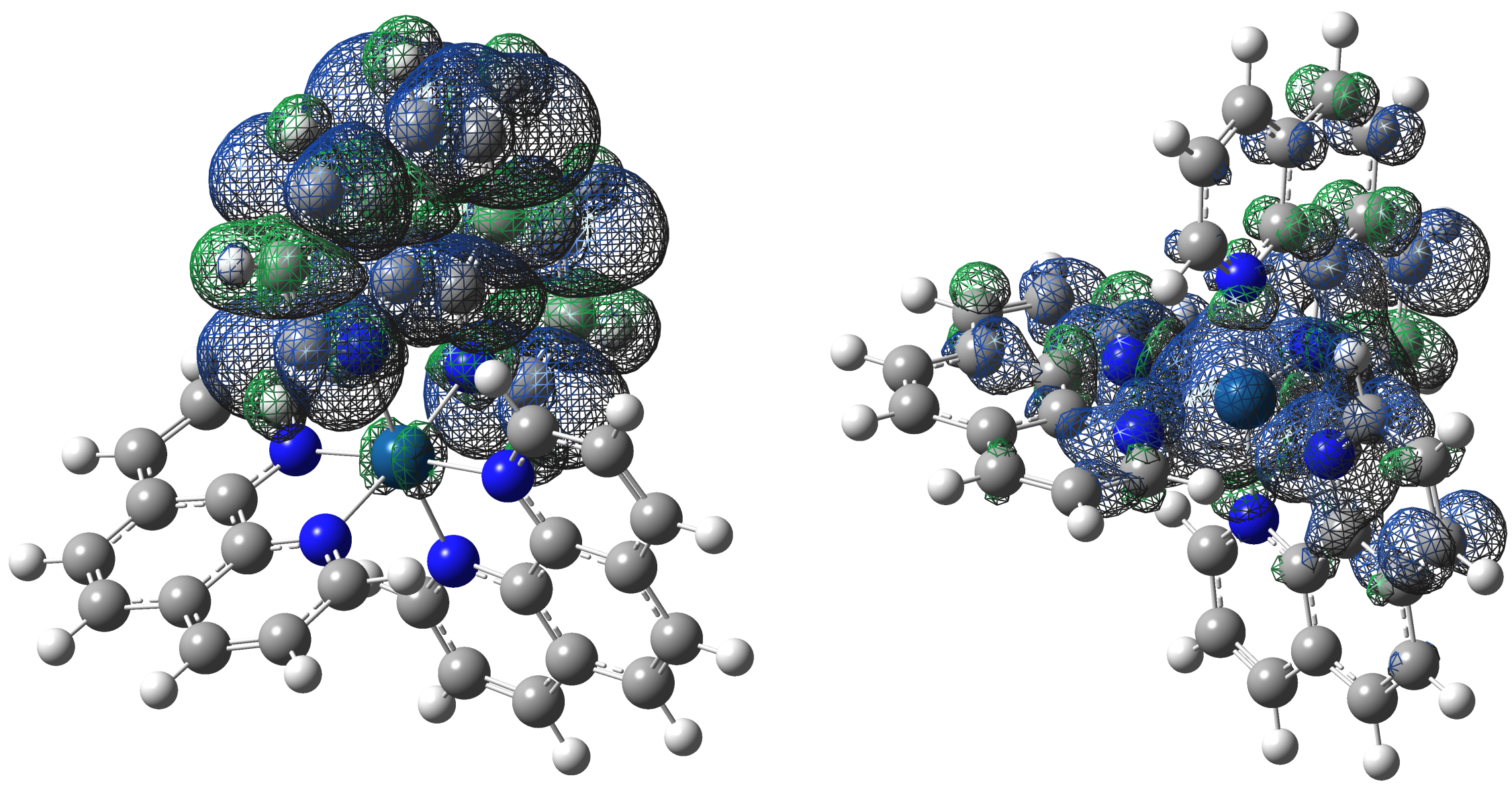

(a) ^{3}LC, Ir spin = 0.00

(b) ^{3}MC, Ir spin = 1.54

Figure S39: Spin density difference orbitals of **phen3** ^{3}LC and ^{3}MC structures calculated at the PBE0-D3/LANL2DZ[Ir]+6-311G(d,p)[C,H,N]/PCM(MeCN) level of theory (Isovalue $\pm$ 0.004)

.

TD-DFT, SOC Matrices, and Reorganization Energies

Singlet TD-DFT of at least 40 states results in broadened spectra that agree with experimental spectra. These excitations are mixed-orbital transitions (even Tables S12−S28). The first 10 excited states of each complex were therefore characterized as natural transition orbitals (Figures S41−S49). Spin-orbit coupling (SOC) of the first 10 singlet and triplet states at the ground state geometry were calculated with the PySOC program.[12] The root-square-sum (RSS) elements of the SOC matrix are reported in cm^{-1} (odd Tables S13−S29). The reorganization energies (λ) of populating the T_{1a} minimum from the S_1 state at the ground state geometry (S_1/S_0) are also reported (Table S30).

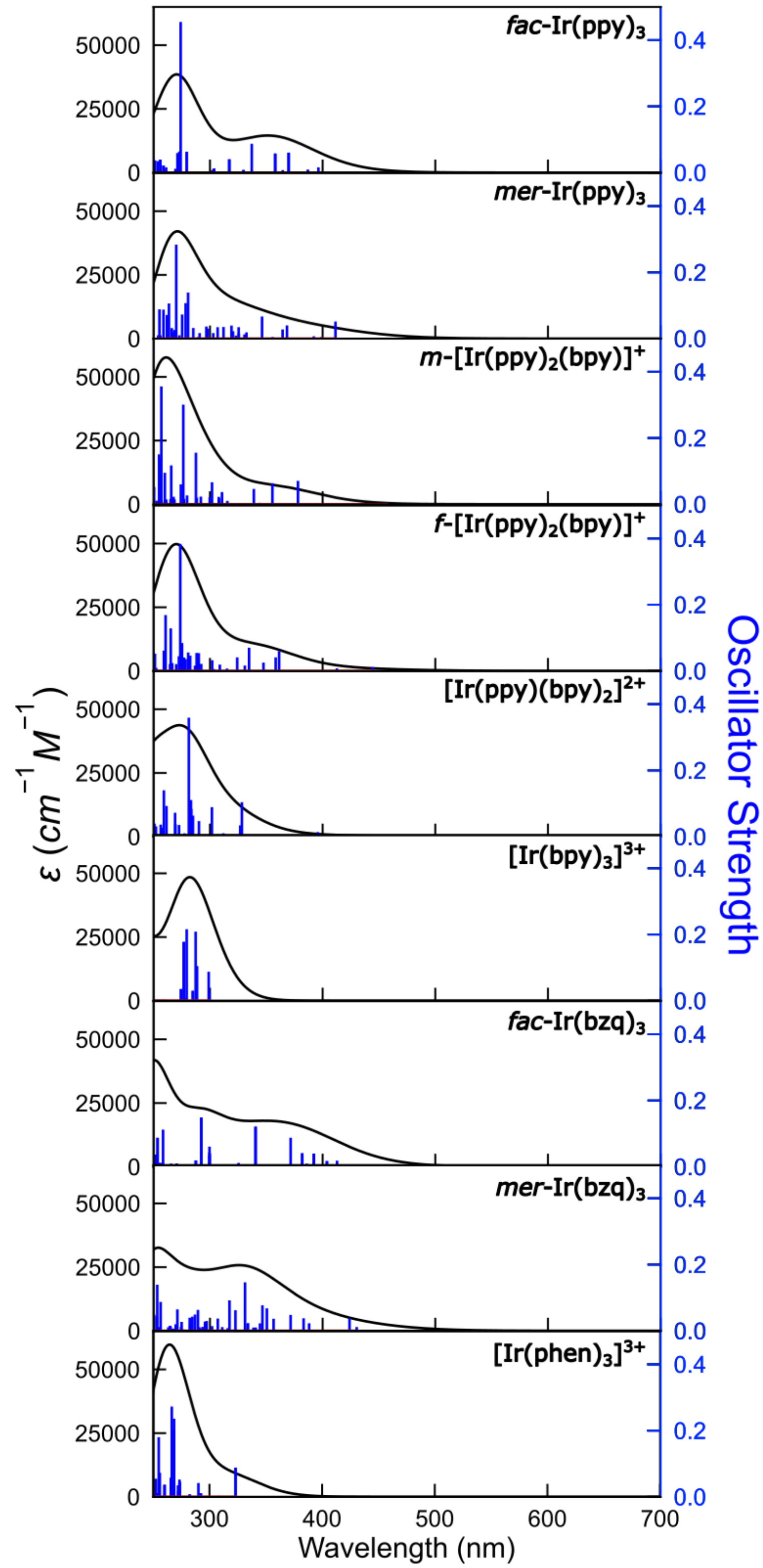

Figure S40: Ground state singlet TD-DFT transitions calculated at the PBE0-D3/LANL2DZ[Ir]+6-311G(d,p)[C,H,N]/PCM(MeCN) level of theory (blue stokes) and corresponding Gaussian-broadened absorbance spectrum (black).

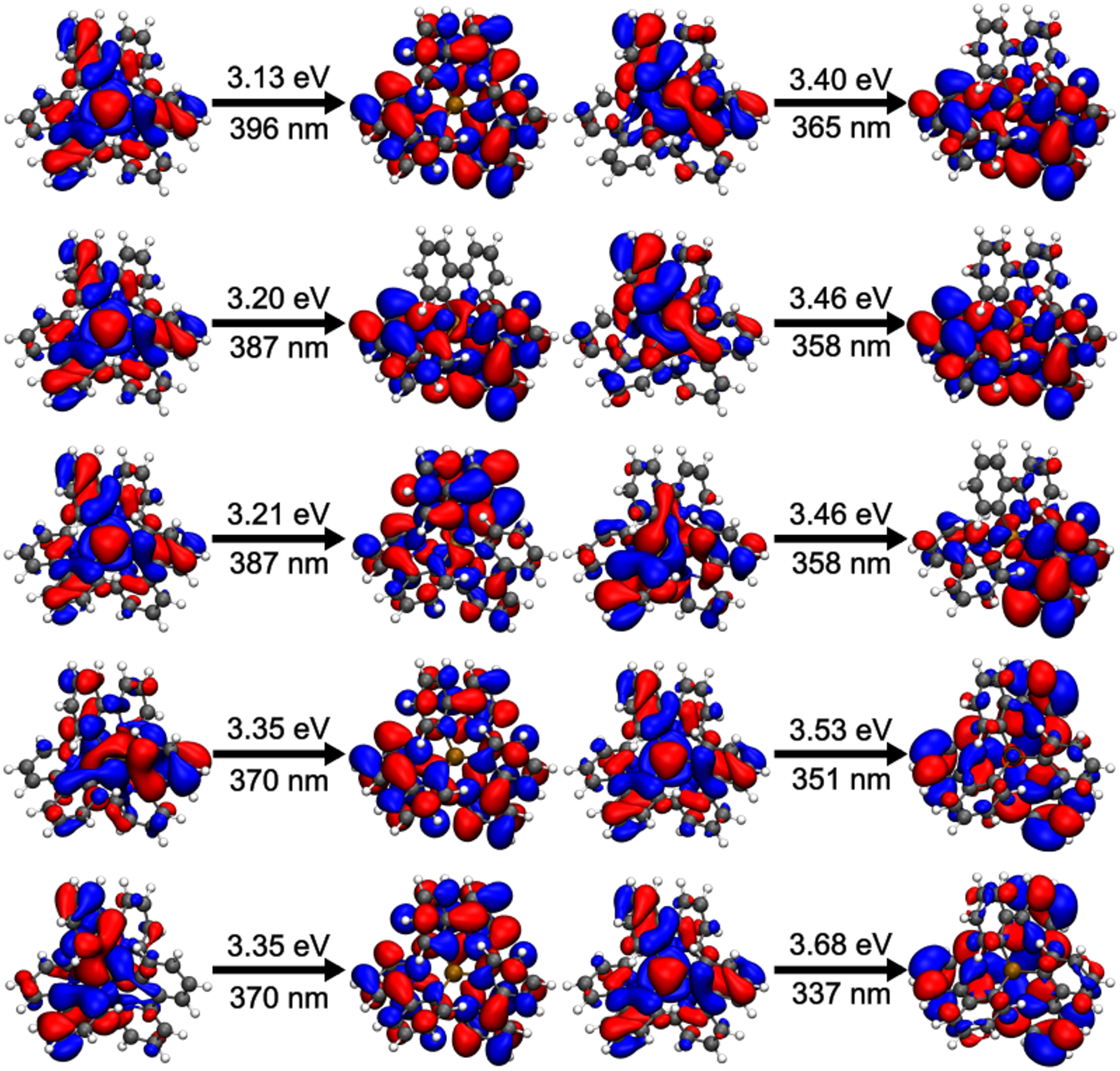

Figure S41: First 10 singlet TD-DFT transitions of ***fac*-ppy3** described as natural transition orbitals (NTOs) (Isovalue ±0.02). Calculated at the PBE0-D3/LANL2DZ[Ir]+6-311G(d,p)[C,H,N]/PCM(MeCN) level of theory.

Table S12: First 10 TD-DFT singlet transitions of ***fac*-ppy3** calculated at the PBE0-D3/LANL2DZ[Ir]+6-311G(d,p)[C,H,N]/PCM(MeCN) level of theory.

Excitation	Energy (eV)	λ (nm)	f	Transitions			
1	3.1288	396.27	0.016	HOMO	→	LUMO	0.692
2	3.2048	386.87	0.0084	HOMO-2	→	LUMO	-0.119
				HOMO	→	LUMO+1	0.679
3	3.2051	386.83	0.0084	HOMO-1	→	LUMO	-0.119
				HOMO	→	LUMO+2	0.679
4	3.352	369.88	0.0594	HOMO-2	→	LUMO	0.402
				HOMO-1	→	LUMO	0.541
				HOMO	→	LUMO+1	0.145
5	3.3524	369.84	0.0605	HOMO-2	→	LUMO	0.541
				HOMO-1	→	LUMO	-0.402
				HOMO	→	LUMO+2	-0.145
6	3.4008	364.57	0.0074	HOMO-2	→	LUMO+1	0.154
				HOMO-2	→	LUMO+2	-0.458
				HOMO-1	→	LUMO+1	0.464
				HOMO-1	→	LUMO+2	0.156
7	3.464	357.92	0.0566	HOMO-2	→	LUMO+1	-0.212
				HOMO-2	→	LUMO+2	0.441
				HOMO-1	→	LUMO	-0.113
				HOMO-1	→	LUMO+1	0.436
				HOMO-1	→	LUMO+2	0.210
8	3.4644	357.88	0.0581	HOMO-2	→	LUMO	-0.114
				HOMO-2	→	LUMO+1	-0.438
				HOMO-2	→	LUMO+2	-0.212
				HOMO-1	→	LUMO+1	-0.210
				HOMO-1	→	LUMO+2	0.439
9	3.5345	350.78	0.0019	HOMO-2	→	LUMO+1	-0.315
				HOMO-2	→	LUMO+2	-0.147
				HOMO-1	→	LUMO+1	0.145
				HOMO-1	→	LUMO+2	-0.314
				HOMO	→	LUMO+3	0.492
10	3.6782	337.08	0.0872	HOMO-2	→	LUMO+1	0.336
				HOMO-1	→	LUMO+2	0.336
				HOMO	→	LUMO+3	0.483

Table S13: Spin-orbit coupling of the first 10 singlet and triplet excited states of ***fac*-ppy3**, with the energy of each state in eV provided in parentheses. Root-square-sum (RSS) matrix values reported in cm^{-1}.

	$\mathbf{T}_1$ (2.72)	$\mathbf{T}_2$ (2.74)	$\mathbf{T}_3$ (2.74)	$\mathbf{T}_4$ (3.09)	$\mathbf{T}_5$ (3.12)	$\mathbf{T}_6$ (3.12)	$\mathbf{T}_7$ (3.25)	$\mathbf{T}_8$ (3.25)	$\mathbf{T}_9$ (3.28)	$\mathbf{T}_{10}$ (3.38)
$\mathbf{S}_0$ (0.00)	99.00	249.88	249.55	188.57	198.72	197.87	453.73	454.94	281.82	97.43
$\mathbf{S}_1$ (3.12)	8.94	201.07	203.21	71.73	777.20	771.09	432.14	425.54	22.90	506.71
$\mathbf{S}_2$ (3.20)	89.75	144.18	129.45	771.91	509.06	515.24	388.56	377.78	502.54	369.83
$\mathbf{S}_3$ (3.20)	91.94	129.49	144.59	772.36	514.10	516.25	368.05	383.31	513.86	373.80
$\mathbf{S}_4$ (3.34)	663.82	159.28	136.49	134.42	578.88	259.72	60.85	213.16	674.97	106.05
$\mathbf{S}_5$ (3.34)	663.80	132.66	162.23	132.78	259.89	574.38	217.99	52.09	674.61	321.75
$\mathbf{S}_6$ (3.39)	25.08	453.22	451.05	706.13	163.07	163.48	532.95	532.72	40.85	217.30
$\mathbf{S}_7$ (3.46)	81.66	424.33	472.08	55.77	177.67	512.76	691.58	556.46	138.71	471.44
$\mathbf{S}_8$ (3.46)	81.90	470.96	424.27	55.41	506.52	174.32	553.76	691.06	165.62	154.24
$\mathbf{S}_9$ (3.53)	125.88	359.21	357.36	88.44	143.76	143.62	419.09	420.99	236.18	235.20
$\mathbf{S}_{10}$ (3.67)	113.61	197.20	196.99	27.82	36.98	35.96	340.84	339.90	231.65	130.59

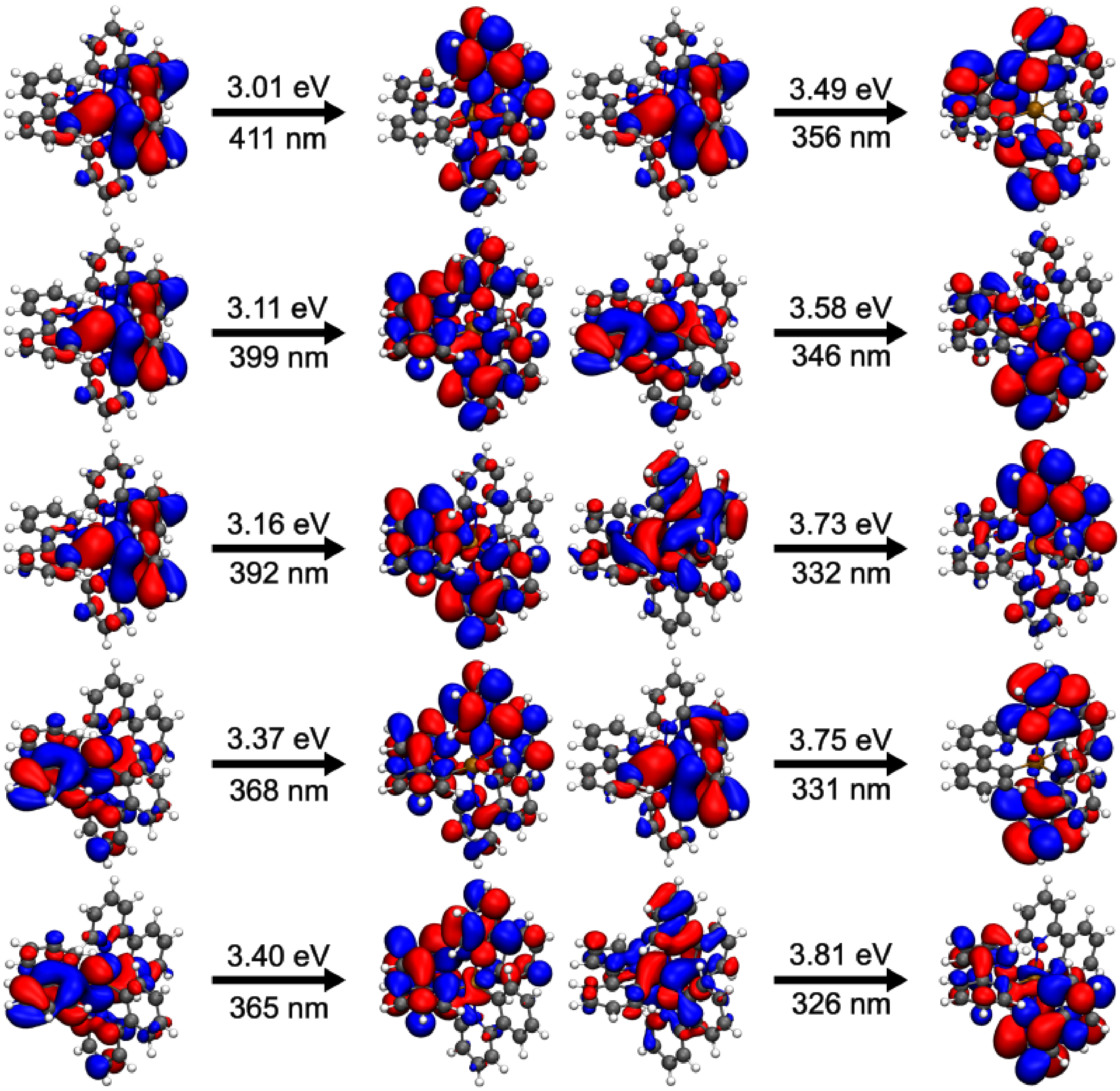

Figure S42: First 10 singlet TD-DFT transitions of ***mer*-ppy3** described as natural transition orbitals (NTOs) (Isovalue ±0.02). Calculated at the PBE0-D3/LANL2DZ[Ir]+6-311G(d,p)[C,H,N]/PCM(MeCN) level of theory.

Table S14: First 10 TD-DFT singlet transitions of ***mer*-ppy3** calculated at the PBE0-D3/LANL2DZ[Ir]+6-311G(d,p)[C,H,N]/PCM(MeCN) level of theory.

**Excitation**	**Energy (eV)**	**λ (nm)**	**f**	**Transitions**			
1	3.0136	411.41	0.0516	HOMO	→	LUMO	0.648
				HOMO	→	LUMO+1	-0.239
2	3.1088	398.82	0.0046	HOMO	→	LUMO	0.202
				HOMO	→	LUMO+1	0.629
				HOMO	→	LUMO+2	0.208
3	3.1617	392.15	0.0072	HOMO-1	→	LUMO+2	0.111
				HOMO	→	LUMO	-0.161
				HOMO	→	LUMO+1	-0.176
				HOMO	→	LUMO+2	0.648
4	3.366	368.34	0.0398	HOMO-1	→	LUMO	0.692
5	3.4019	364.46	0.0277	HOMO-1	→	LUMO+1	0.693
6	3.487	355.56	0.0048	HOMO	→	LUMO+3	0.692
7	3.5811	346.21	0.0669	HOMO-1	→	LUMO+2	0.662
				HOMO	→	LUMO+2	-0.124
8	3.7299	332.4	0.0189	HOMO-2	→	LUMO	0.476
				HOMO-2	→	LUMO+1	-0.175
				HOMO-1	→	LUMO+3	0.142
				HOMO	→	LUMO+4	0.430
9	3.75	330.63	0.0128	HOMO-2	→	LUMO	-0.378
				HOMO-2	→	LUMO+1	0.177
				HOMO	→	LUMO+4	0.532
10	3.8078	325.61	0.0157	HOMO-3	→	LUMO	0.382
				HOMO-3	→	LUMO+2	0.188
				HOMO-2	→	LUMO	-0.122
				HOMO-2	→	LUMO+1	-0.375
				HOMO-2	→	LUMO+2	-0.264
				HOMO-1	→	LUMO+3	-0.216
				HOMO	→	LUMO+4	0.111

Table S15: Spin-orbit coupling of the first 10 singlet and triplet excited states of ***mer*-ppy3**, with the energy of each state in eV provided in parentheses. Root-square-sum (RSS) matrix values reported in cm^{-1}.

	$\mathbf{T}_1$ (2.68)	$\mathbf{T}_2$ (2.72)	$\mathbf{T}_3$ (2.82)	$\mathbf{T}_4$ (2.98)	$\mathbf{T}_5$ (3.05)	$\mathbf{T}_6$ (3.10)	$\mathbf{T}_7$ (3.30)	$\mathbf{T}_8$ (3.30)	$\mathbf{T}_9$ (3.23)	$\mathbf{T}_{10}$ (3.39)
$\mathbf{S}_0$ (0.00)	253.01	235.08	157.25	40.71	230.48	326.74	228.17	134.01	260.87	280.11
$\mathbf{S}_1$ (3.01)	63.60	158.07	75.91	316.52	446.90	149.89	345.32	117.82	731.12	191.88
$\mathbf{S}_2$ (3.10)	78.00	72.72	296.73	418.81	303.32	59.04	404.27	478.60	275.34	309.18
$\mathbf{S}_3$ (3.15)	31.73	68.23	283.85	481.49	90.90	229.44	153.68	437.04	347.18	298.02
$\mathbf{S}_4$ (3.36)	539.91	259.54	128.54	162.09	399.07	488.20	136.96	203.19	90.66	128.54
$\mathbf{S}_5$ (3.39)	185.01	418.48	187.67	130.56	553.85	570.37	163.26	259.70	211.77	96.67
$\mathbf{S}_6$ (3.48)	120.79	82.38	56.19	55.98	70.38	24.30	57.55	107.23	23.72	213.52
$\mathbf{S}_7$ (3.57)	351.66	421.64	137.29	293.03	106.68	405.43	153.85	232.29	33.77	309.91
$\mathbf{S}_8$ (3.72)	400.31	128.84	100.96	202.64	270.62	198.76	261.64	55.62	492.00	192.05
$\mathbf{S}_9$ (3.74)	210.40	114.57	99.08	87.70	280.97	205.40	218.22	92.60	393.99	98.93
$\mathbf{S}_{10}$ (3.80)	645.62	341.92	156.60	599.15	92.76	231.59	160.19	169.39	232.18	683.12

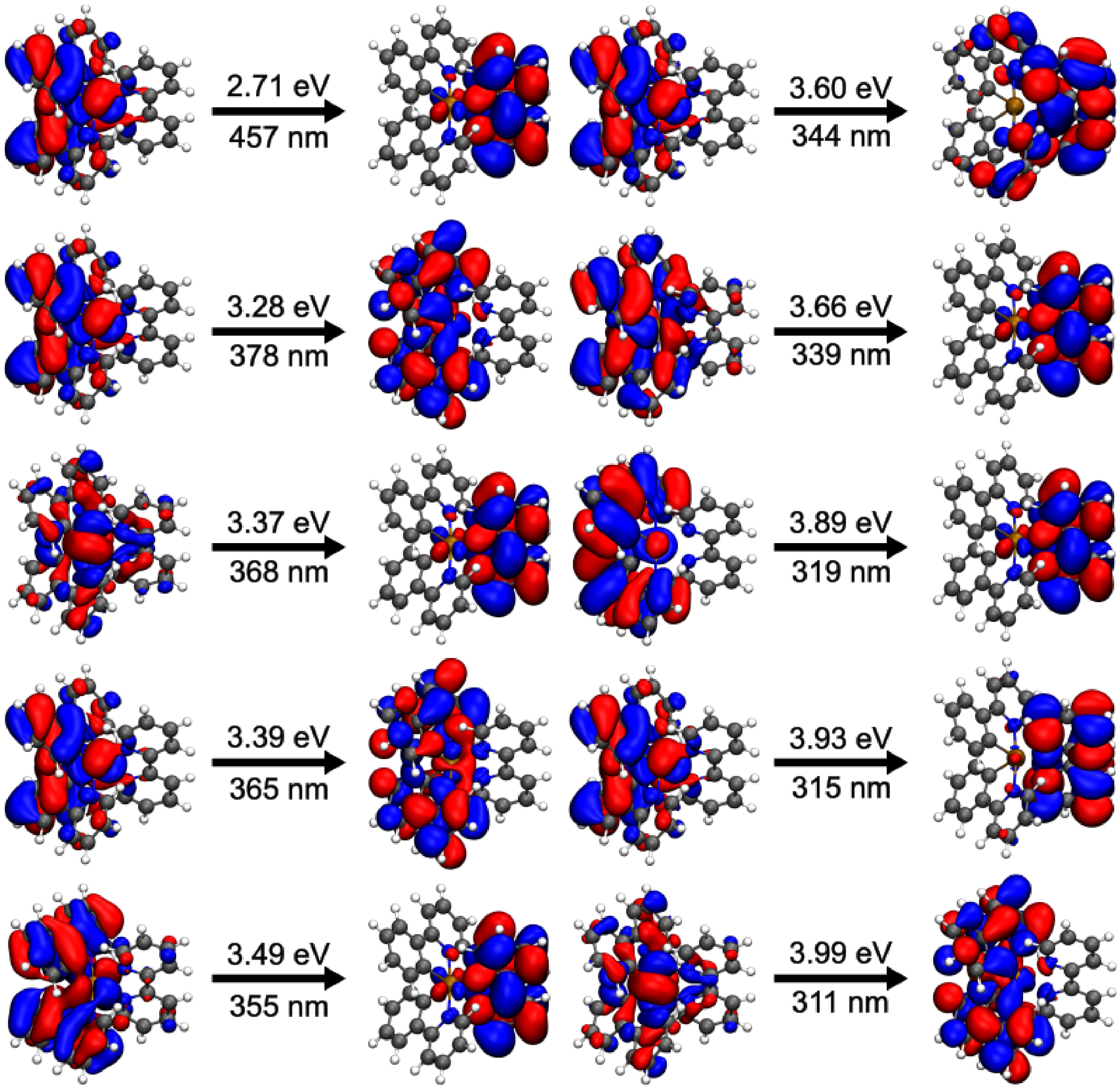

Figure S43: First 10 singlet TD-DFT transitions of ***m*-ppy2** described as natural transition orbitals (NTOs) (Isovalue ±0.02). Calculated at the PBE0-D3/LANL2DZ[Ir]+6-311G(d,p)[C,H,N]/PCM(MeCN) level of theory.

Table S16: First 10 TD-DFT singlet transitions of ***m*-ppy2** calculated at the PBE0-D3/LANL2DZ[Ir]+6-311G(d,p)[C,H,N]/PCM(MeCN) level of theory.

**Excitation**	**Energy (eV)**	**λ (nm)**	**f**	**Transitions**			
1	2.7124	457.1	0.0004	HOMO	→	LUMO	0.700
2	3.2791	378.1	0.0707	HOMO	→	LUMO+1	0.692
3	3.3691	368.01	0.0001	HOMO-4	→	LUMO	0.247
				HOMO-2	→	LUMO	0.657
4	3.3939	365.31	0.0005	HOMO	→	LUMO+2	0.684
5	3.4886	355.4	0.0636	HOMO-5	→	LUMO	0.113
				HOMO-3	→	LUMO	-0.285
				HOMO-1	→	LUMO	0.618
				HOMO	→	LUMO+2	-0.118
6	3.6039	344.03	0.0044	HOMO	→	LUMO+3	0.698
7	3.6582	338.92	0.0465	HOMO-5	→	LUMO	-0.118
				HOMO-3	→	LUMO	0.605
				HOMO-1	→	LUMO	0.320
8	3.8854	319.11	0.0001	HOMO-4	→	LUMO	0.653
				HOMO-2	→	LUMO	-0.251
9	3.9307	315.43	0.0104	HOMO	→	LUMO+4	0.689
10	3.9909	310.67	0.037	HOMO-4	→	LUMO+1	0.211
				HOMO-3	→	LUMO+2	0.166
				HOMO-2	→	LUMO+1	0.615
				HOMO-1	→	LUMO+2	-0.145

Table S17: Spin-orbit coupling of the first 10 singlet and triplet excited states of ***m*-ppy2**, with the energy of each state in eV provided in parentheses. Root-square-sum (RSS) matrix values reported in cm^{-1}.

	**T**$_1$ (2.66)	**T**$_2$ (2.77)	**T**$_3$ (2.80)	**T**$_4$ (2.93)	**T**$_5$ (3.17)	**T**$_6$ (3.26)	**T**$_7$ (3.29)	**T**$_8$ (3.39)	**T**$_9$ (3.45)	**T**$_{10}$ (3.58)
**S**$_0$ (0.00)	335.11	406.13	97.58	127.45	35.09	356.36	227.70	333.39	193.56	44.21
**S**$_1$ (2.70)	106.71	5.26	50.45	540.40	1061.61	6.29	162.74	916.95	45.32	62.74
**S**$_2$ (3.27)	2.32	85.40	170.14	30.74	154.14	312.60	261.11	81.64	125.40	201.99
**S**$_3$ (3.36)	1142.22	96.00	23.89	174.50	1173.01	50.75	149.91	21.28	10.84	66.24
**S**$_4$ (3.39)	282.52	86.79	94.25	116.71	74.26	287.76	315.89	171.84	103.11	287.87
**S**$_5$ (3.48)	839.28	36.98	2.89	462.90	67.41	110.71	126.57	772.45	58.30	39.80
**S**$_6$ (3.59)	4.88	41.10	27.71	44.01	96.76	13.54	49.76	13.88	57.90	109.05
**S**$_7$ (3.65)	883.35	65.42	28.60	270.87	111.92	75.92	92.89	570.73	75.67	25.00
**S**$_8$ (3.88)	124.22	37.56	23.52	156.42	363.23	42.92	43.38	220.09	24.01	12.23
**S**$_9$ (3.92)	44.03	71.39	17.75	122.04	82.47	111.16	74.89	75.60	17.44	39.11
**S**$_{10}$ (3.98)	46.32	541.25	286.51	37.69	111.73	686.10	366.89	8.97	22.78	355.69

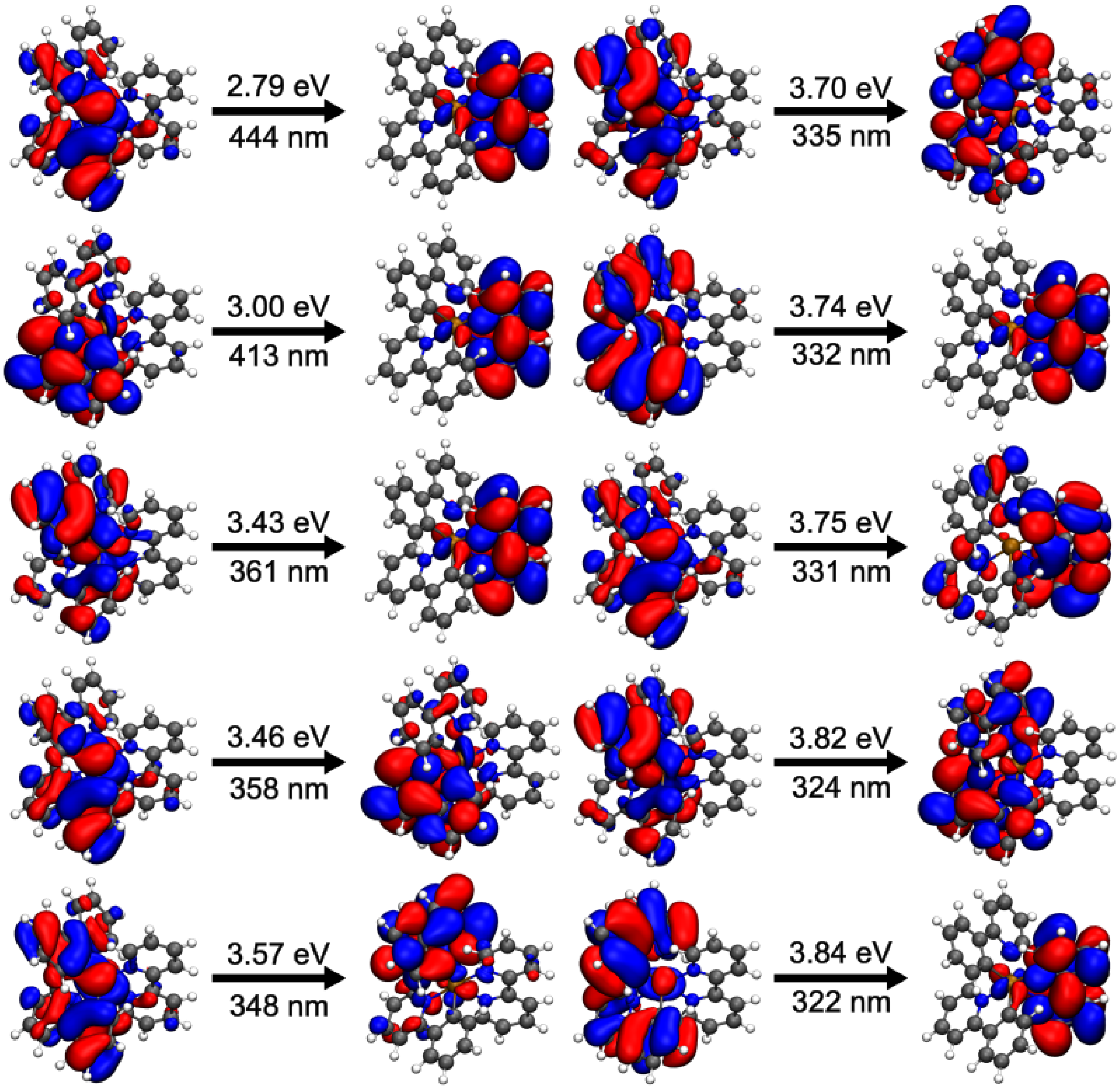

Figure S44: First 10 singlet TD-DFT transitions of ***f*-ppy2** described as natural transition orbitals (NTOs) (Isovalue ±0.02). Calculated at the PBE0-D3/LANL2DZ[Ir]+6-311G(d,p)[C,H,N]/PCM(MeCN) level of theory.

Table S18: First 10 TD-DFT singlet transitions of ***f*-ppy2** calculated at the PBE0-D3/LANL2DZ[Ir]+6-311G(d,p)[C,H,N]/PCM(MeCN) level of theory.

**Excitation**	**Energy (eV)**	**λ (nm)**	**f**	**Transitions**			
1	2.7897	444.44	0.0117	HOMO	→	LUMO	0.694
2	3.0044	412.68	0.0082	HOMO-1	→	LUMO	0.693
3	3.4309	361.38	0.0602	HOMO-4	→	LUMO	-0.104
				HOMO-3	→	LUMO	-0.125
				HOMO-2	→	LUMO	0.217
				HOMO	→	LUMO+1	0.599
				HOMO	→	LUMO+2	0.221
4	3.4602	358.32	0.0413	HOMO-4	→	LUMO	-0.223
				HOMO-3	→	LUMO	-0.267
				HOMO-2	→	LUMO	0.522
				HOMO	→	LUMO+1	-0.218
				HOMO	→	LUMO+2	-0.171
5	3.5681	347.48	0.0254	HOMO-1	→	LUMO+1	0.168
				HOMO	→	LUMO+1	-0.242
				HOMO	→	LUMO+2	0.617
6	3.7044	334.7	0.0703	HOMO-1	→	LUMO+1	0.627
				HOMO-1	→	LUMO+2	-0.214
				HOMO	→	LUMO+1	0.111
				HOMO	→	LUMO+2	-0.160
7	3.7352	331.93	0.0008	HOMO-3	→	LUMO	0.316
				HOMO-2	→	LUMO	0.250
				HOMO	→	LUMO+3	0.549
8	3.7464	330.94	0.0166	HOMO-3	→	LUMO	0.488
				HOMO-2	→	LUMO	0.255
				HOMO-1	→	LUMO+2	-0.193
				HOMO	→	LUMO+3	-0.362
9	3.8248	324.16	0.0411	HOMO-3	→	LUMO	0.118
				HOMO-1	→	LUMO+1	0.205
				HOMO-1	→	LUMO+2	0.606
				HOMO	→	LUMO+3	-0.190
				HOMO	→	LUMO+4	-0.118
10	3.8449	322.47	0.0008	HOMO-4	→	LUMO	0.641
				HOMO-3	→	LUMO	-0.207
				HOMO-2	→	LUMO	0.166

Table S19: Spin-orbit coupling of the first 10 singlet and triplet excited states of ***f*-ppy2**, with the energy of each state in eV provided in parentheses. Root-square-sum (RSS) matrix values reported in cm^{-1}.

	$\mathbf{T}_1$ (2.63)	$\mathbf{T}_2$ (2.79)	$\mathbf{T}_3$ (2.85)	$\mathbf{T}_4$ (2.90)	$\mathbf{T}_5$ (2.94)	$\mathbf{T}_6$ (3.28)	$\mathbf{T}_7$ (3.32)	$\mathbf{T}_8$ (3.46)	$\mathbf{T}_9$ (3.50)	$\mathbf{T}_{10}$ (3.54)
$\mathbf{S}_0$ (0.00)	371.02	219.26	103.15	71.10	327.85	270.71	113.65	209.74	334.50	386.13
$\mathbf{S}_1$ (2.78)	157.11	65.62	96.64	903.12	821.66	675.96	260.87	50.87	28.95	75.30
$\mathbf{S}_2$ (3.00)	885.28	71.09	54.33	572.33	270.94	553.85	238.22	50.81	35.94	82.65
$\mathbf{S}_3$ (3.42)	456.67	80.23	109.95	223.61	368.33	182.75	546.34	131.91	294.58	349.44
$\mathbf{S}_4$ (3.45)	971.85	60.05	116.92	381.48	783.75	193.05	277.27	88.32	130.21	134.71
$\mathbf{S}_5$ (3.56)	134.76	85.94	235.09	90.61	54.40	92.02	99.15	375.48	323.18	215.70
$\mathbf{S}_6$ (3.70)	61.81	260.25	252.55	43.81	92.63	88.78	251.07	494.82	143.91	58.03
$\mathbf{S}_7$ (3.73)	83.83	127.92	31.35	90.02	40.82	85.69	63.20	21.24	203.34	125.13
$\mathbf{S}_8$ (3.74)	327.65	76.27	43.17	117.09	236.78	186.45	66.22	43.55	118.85	99.56
$\mathbf{S}_9$ (3.82)	118.05	303.18	179.57	49.64	24.47	64.65	240.11	297.28	184.15	375.40
$\mathbf{S}_{10}$ (3.84)	233.99	21.54	38.34	274.90	225.45	236.07	91.92	24.13	14.56	22.54

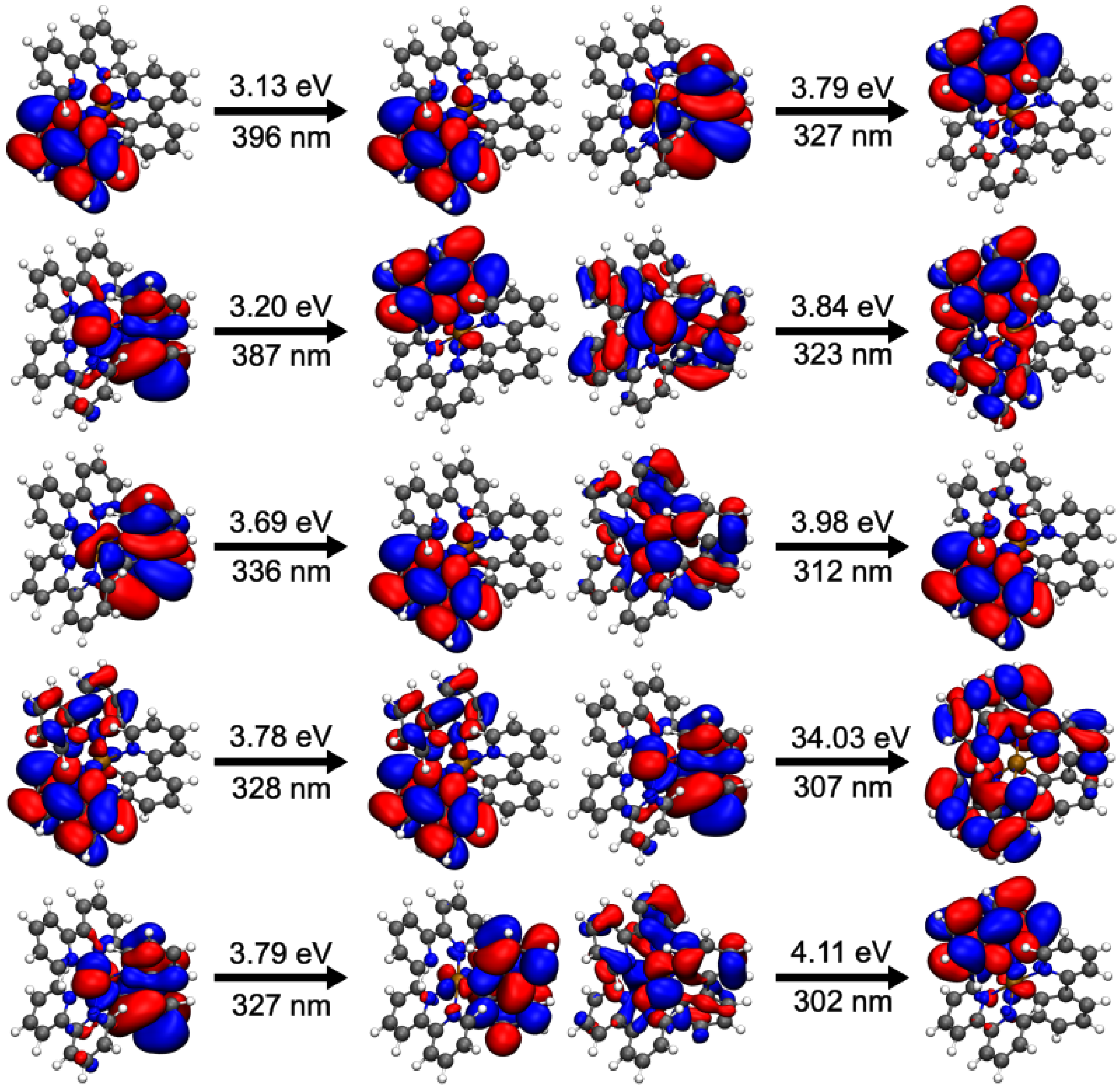

Figure S45: First 10 singlet TD-DFT transitions of **bpy2** described as natural transition orbitals (NTOs) (Isovalue ±0.02). Calculated at the PBE0-D3/LANL2DZ[Ir]+6-311G(d,p)[C,H,N]/PCM(MeCN) level of theory.

Table S20: First 10 TD-DFT singlet transitions of **bpy2** calculated at the PBE0-D3/LANL2DZ[Ir]+6-311G(d,p)[C,H,N]/PCM(MeCN) level of theory.

Excitation	Energy (eV)	λ (nm)	f	Transitions			
1	3.1331	395.72	0.014	HOMO	→	LUMO	0.646
				HOMO	→	LUMO+1	-0.243
2	3.2025	387.14	0.0001	HOMO	→	LUMO	0.249
				HOMO	→	LUMO+1	0.648
3	3.694	335.64	0.0044	HOMO-3	→	LUMO	0.130
				HOMO-1	→	LUMO	0.642
				HOMO-1	→	LUMO+1	-0.217
4	3.7764	328.32	0.1043	HOMO-2	→	LUMO	0.567
				HOMO	→	LUMO+2	0.393
5	3.7933	326.85	0.0342	HOMO-2	→	LUMO	-0.356
				HOMO-2	→	LUMO+1	-0.236
				HOMO-1	→	LUMO	0.126
				HOMO-1	→	LUMO+1	0.180
				HOMO	→	LUMO+2	0.490
6	3.7949	326.71	0.01	HOMO-3	→	LUMO+1	0.116
				HOMO-2	→	LUMO	0.141
				HOMO-2	→	LUMO+1	-0.102
				HOMO-1	→	LUMO	0.187
				HOMO-1	→	LUMO+1	0.601
				HOMO	→	LUMO+2	-0.208
7	3.8419	322.72	0.0034	HOMO-3	→	LUMO+1	-0.115
				HOMO-2	→	LUMO+1	0.625
				HOMO-1	→	LUMO+1	0.181
				HOMO	→	LUMO+2	0.141
8	3.975	311.91	0.0104	HOMO-4	→	LUMO	0.137
				HOMO-3	→	LUMO	0.629
				HOMO-3	→	LUMO+1	-0.178
				HOMO-1	→	LUMO	-0.133
9	4.0342	307.34	0.0066	HOMO	→	LUMO+2	0.108
				HOMO	→	LUMO+3	0.661
10	4.1093	301.71	0.0892	HOMO-4	→	LUMO+1	0.120
				HOMO-3	→	LUMO	0.196
				HOMO-3	→	LUMO+1	0.609
				HOMO-2	→	LUMO+1	0.118
				HOMO	→	LUMO+3	0.101

Table S21: Spin-orbit coupling of the first 10 singlet and triplet excited states of **bpy2**, with the energy of each state in eV provided in parentheses. Root-square-sum (RSS) matrix values reported in cm^{-1}.

	$\mathbf{T}_1$ (2.84)	$\mathbf{T}_2$ (2.86)	$\mathbf{T}_3$ (2.95)	$\mathbf{T}_4$ (3.12)	$\mathbf{T}_5$ (3.18)	$\mathbf{T}_6$ (3.46)	$\mathbf{T}_7$ (3.54)	$\mathbf{T}_8$ (3.55)	$\mathbf{T}_9$ (3.63)	$\mathbf{T}_{10}$ (3.68)
$\mathbf{S}_0$ (0.00)	247.35	166.27	112.53	248.72	292.53	314.77	148.05	89.92	65.12	176.37
$\mathbf{S}_1$ (3.13)	53.44	99.29	15.83	404.62	67.11	678.09	215.11	64.32	106.77	413.39
$\mathbf{S}_2$ (3.19)	13.02	42.76	266.38	92.22	73.25	249.82	642.12	333.55	30.93	133.10
$\mathbf{S}_3$ (3.69)	211.24	195.28	20.58	301.98	33.20	369.12	116.33	36.38	22.51	52.39
$\mathbf{S}_4$ (3.76)	386.37	336.35	146.30	587.88	405.01	19.89	265.64	152.33	161.15	499.44
$\mathbf{S}_5$ (3.78)	106.33	111.27	166.01	111.26	494.77	77.22	341.54	257.35	86.85	160.40
$\mathbf{S}_6$ (3.79)	110.29	134.07	204.24	185.51	391.55	180.24	314.36	157.71	37.73	200.25
$\mathbf{S}_7$ (3.83)	223.92	235.99	179.65	421.16	688.97	252.09	589.55	252.97	48.58	219.73
$\mathbf{S}_8$ (3.96)	502.31	486.95	48.21	582.08	170.10	841.70	365.30	97.57	89.87	359.36
$\mathbf{S}_9$ (4.03)	39.31	47.57	59.77	17.29	155.76	74.71	139.04	72.95	59.62	70.00
$\mathbf{S}_{10}$ (4.10)	100.65	120.39	398.00	138.94	826.75	278.27	333.28	268.01	40.03	121.47

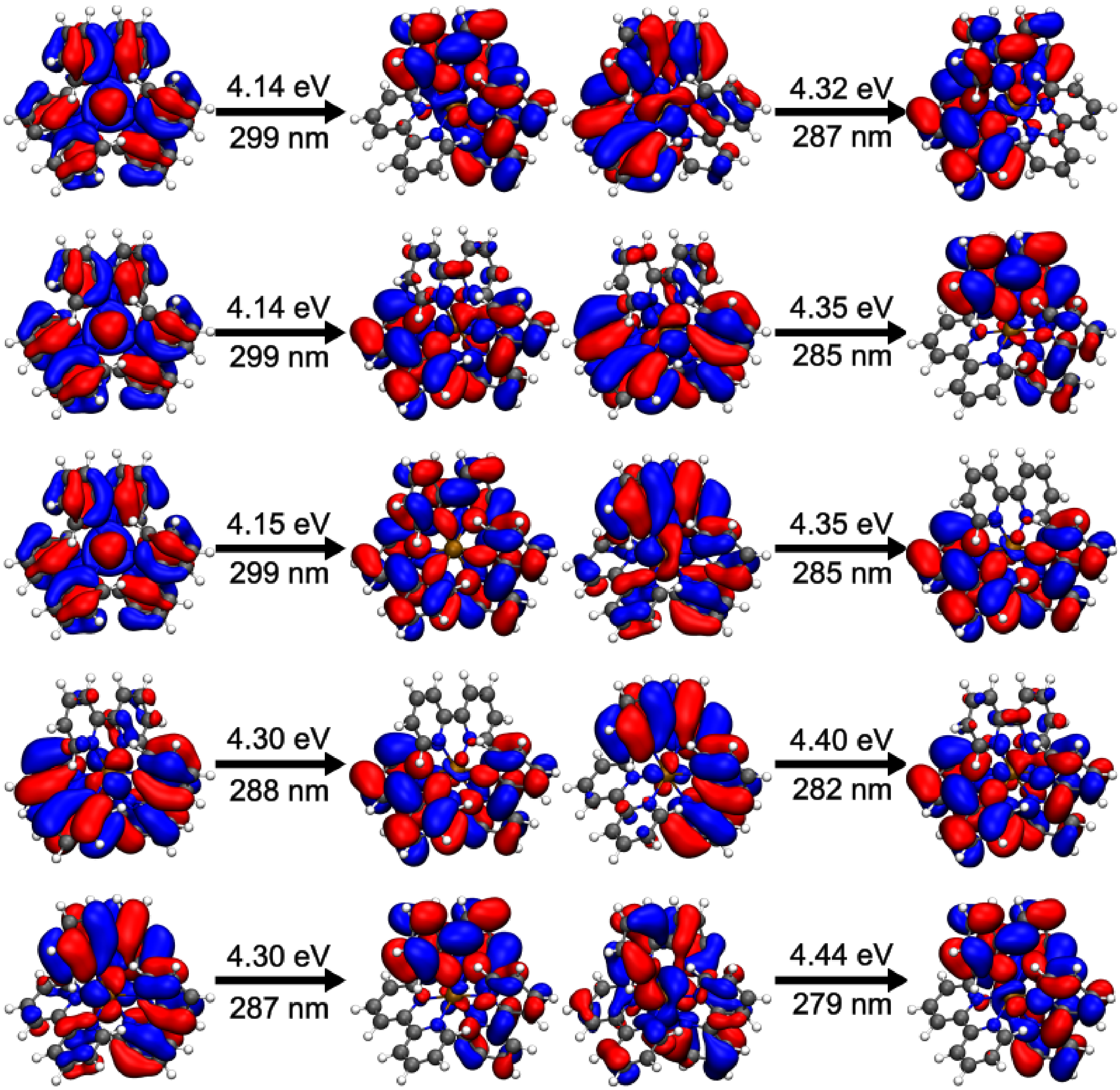

Figure S46: First 10 singlet TD-DFT transitions of **bpy3** described as natural transition orbitals (NTOs) (Isovalue ±0.02). Calculated at the PBE0-D3/LANL2DZ[Ir]+6-311G(d,p)[C,H,N]/PCM(MeCN) level of theory.

Table S22: First 10 TD-DFT singlet transitions of **bpy3** calculated at the PBE0-D3/LANL2DZ[Ir]+6-311G(d,p)[C,H,N]/PCM(MeCN) level of theory.

Excitation	Energy (eV)	λ (nm)	f	Transitions			
1	4.1403	299.45	0.0242	HOMO-3	→	LUMO+1	0.322
				HOMO	→	LUMO+1	0.607
2	4.1438	299.21	0.0246	HOMO-3	→	LUMO+2	0.321
				HOMO	→	LUMO+2	0.608
3	4.1486	298.86	0.0875	HOMO-3	→	LUMO	0.300
				HOMO	→	LUMO	0.620
4	4.2988	288.41	0.1043	HOMO-5	→	LUMO	0.119
				HOMO-5	→	LUMO+2	0.106
				HOMO-4	→	LUMO+1	0.120
				HOMO-3	→	LUMO+2	-0.145
				HOMO-2	→	LUMO+1	-0.255
				HOMO-2	→	LUMO+2	-0.168
				HOMO-1	→	LUMO	0.474
				HOMO-1	→	LUMO+1	-0.180
				HOMO-1	→	LUMO+2	0.237
5	4.3002	288.32	0.1033	HOMO-5	→	LUMO+1	-0.116
				HOMO-4	→	LUMO	-0.117
				HOMO-4	→	LUMO+2	0.115
				HOMO-3	→	LUMO+1	-0.146
				HOMO-2	→	LUMO	0.465
				HOMO-2	→	LUMO+1	0.178
				HOMO-2	→	LUMO+2	-0.244
				HOMO-1	→	LUMO+1	-0.258
				HOMO-1	→	LUMO+2	-0.178
6	4.3157	287.29	0.2087	HOMO-5	→	LUMO+2	0.236
				HOMO-4	→	LUMO+1	-0.238
				HOMO-2	→	LUMO+1	0.416
				HOMO-1	→	LUMO+2	0.424
7	4.353	284.82	0.0281	HOMO-5	→	LUMO	0.234
				HOMO-5	→	LUMO+2	-0.121
				HOMO-4	→	LUMO+1	-0.120
				HOMO-2	→	LUMO+1	0.253
				HOMO-2	→	LUMO+2	0.184
				HOMO-1	→	LUMO	0.423
				HOMO-1	→	LUMO+1	0.212
				HOMO-1	→	LUMO+2	-0.263

8	4.3542	284.74	0.0306	HOMO-5	→	LUMO+1	0.119
				HOMO-4	→	LUMO	-0.241
				HOMO-4	→	LUMO+2	-0.117
				HOMO-2	→	LUMO	0.431
				HOMO-2	→	LUMO+1	-0.194
				HOMO-2	→	LUMO+2	0.235
				HOMO-1	→	LUMO+1	0.270
				HOMO-1	→	LUMO+2	0.195
9	4.3957	282.06	0	HOMO-2	→	LUMO+2	0.514
				HOMO-1	→	LUMO+1	-0.479
10	4.4387	279.33	0.2157	HOMO-5	→	LUMO+2	-0.385
				HOMO-4	→	LUMO+1	0.439
				HOMO-3	→	LUMO	-0.194
				HOMO-2	→	LUMO+1	0.212
				HOMO-1	→	LUMO+2	0.196
				HOMO	→	LUMO+3	-0.118

Table S23: Spin-orbit coupling of the first 10 singlet and triplet excited states of **bpy3**, with the energy of each state in eV provided in parentheses. Root-square-sum (RSS) matrix values reported in cm^{-1}.

	$\mathbf{T}_1$ (2.95)	$\mathbf{T}_2$ (2.95)	$\mathbf{T}_3$ (2.95)	$\mathbf{T}_4$ (3.73)	$\mathbf{T}_5$ (3.75)	$\mathbf{T}_6$ (3.75)	$\mathbf{T}_7$ (3.84)	$\mathbf{T}_8$ (3.84)	$\mathbf{T}_9$ (3.85)	$\mathbf{T}_{10}$ (4.05)
$\mathbf{S}_0$ (0.00)	58.08	57.12	11.52	4.78	22.84	22.06	394.56	385.09	54.04	608.57
$\mathbf{S}_1$ (4.13)	64.53	64.68	81.68	508.12	370.79	354.78	84.20	85.29	121.97	32.56
$\mathbf{S}_2$ (4.13)	67.78	69.85	73.37	486.38	371.31	376.19	85.28	77.95	122.47	51.50
$\mathbf{S}_3$ (4.14)	68.97	67.42	6.05	15.05	421.10	426.79	166.60	165.64	3.23	39.83
$\mathbf{S}_4$ (4.29)	92.70	101.71	99.42	115.92	195.19	267.08	205.41	185.47	217.81	241.73
$\mathbf{S}_5$ (4.30)	91.27	86.60	113.72	116.90	267.50	194.78	200.42	197.46	211.53	238.78
$\mathbf{S}_6$ (4.31)	138.96	138.33	14.21	394.22	25.33	30.66	321.91	323.30	6.39	400.12
$\mathbf{S}_7$ (4.35)	65.89	100.18	122.11	6.06	29.95	28.35	206.61	170.57	252.02	223.58
$\mathbf{S}_8$ (4.35)	106.98	77.50	110.88	4.32	28.26	28.58	170.99	198.26	258.99	213.41
$\mathbf{S}_9$ (4.40)	45.80	36.79	14.23	2.43	16.07	10.74	85.09	106.75	56.32	106.51
$\mathbf{S}_{10}$ (4.43)	106.30	127.73	23.79	420.79	124.46	121.02	302.92	247.40	24.86	443.09

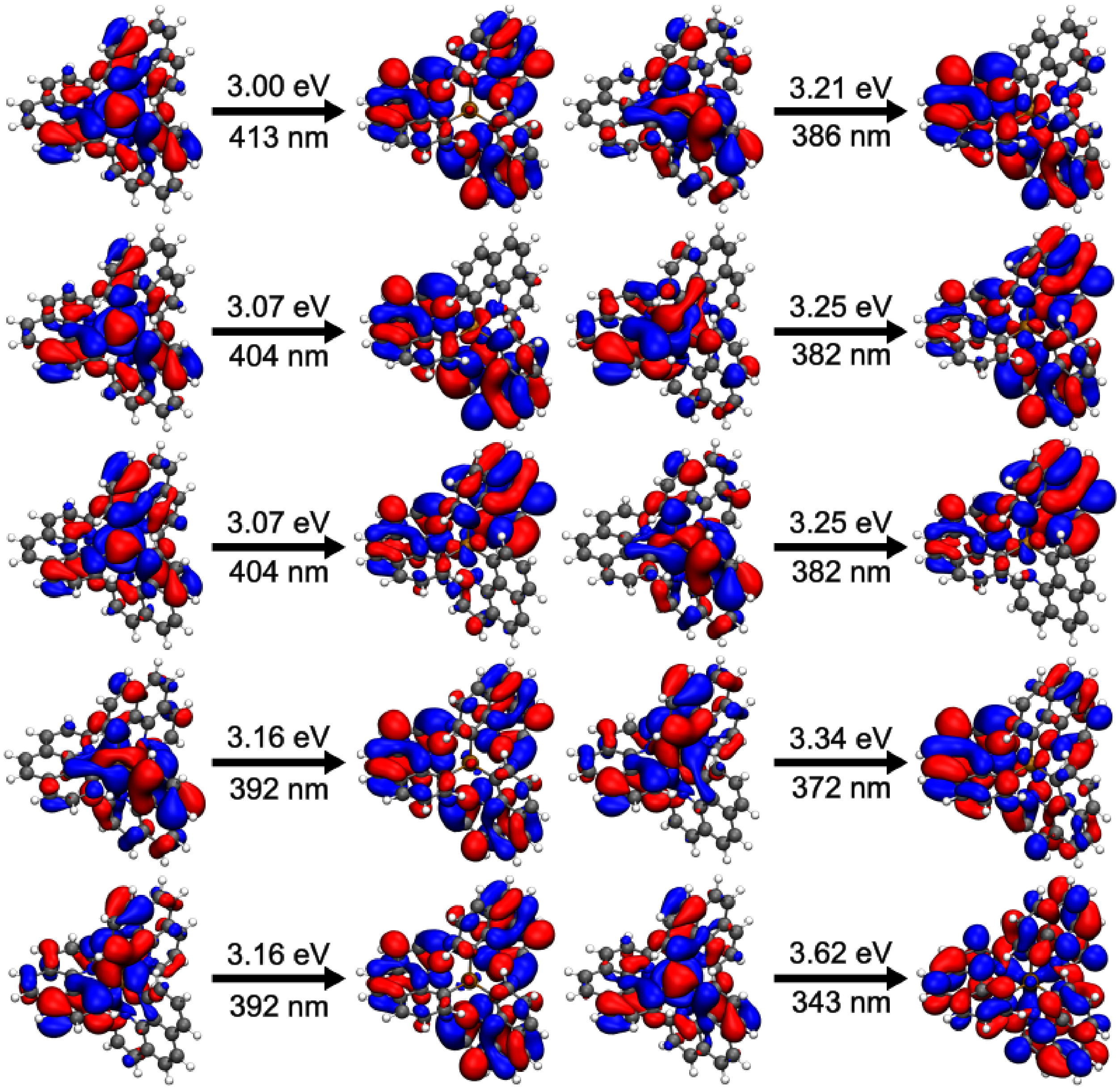

Figure S47: First 10 singlet TD-DFT transitions of ***fac*-bzq3** described as natural transition orbitals (NTOs) (Isovalue ±0.02). Calculated at the PBE0-D3/LANL2DZ[Ir]+6-311G(d,p)[C,H,N]/PCM(MeCN) level of theory.

Table S24: First 10 TD-DFT singlet transitions of ***fac*-bzq3** calculated at the PBE0-D3/LANL2DZ[Ir]+6-311G(d,p)[C,H,N]/PCM(MeCN) level of theory.

Excitation	Energy (eV)	λ (nm)	f	Transitions			
1	3.0035	412.8	0.0186	HOMO	→	LUMO	0.680
2	3.0703	403.81	0.017	HOMO-1	→	LUMO	0.248
				HOMO	→	LUMO+1	0.570
				HOMO	→	LUMO+2	0.285
3	3.0705	403.79	0.017	HOMO-2	→	LUMO	0.247
				HOMO	→	LUMO+1	-0.285
				HOMO	→	LUMO+2	0.570
4	3.161	392.23	0.0399	HOMO-1	→	LUMO	0.641
				HOMO	→	LUMO+1	-0.214
				HOMO	→	LUMO+2	-0.139
5	3.1614	392.18	0.0397	HOMO-2	→	LUMO	0.642
				HOMO	→	LUMO+1	0.139
				HOMO	→	LUMO+2	-0.213
6	3.2125	385.94	0.0094	HOMO-2	→	LUMO+1	-0.419
				HOMO-2	→	LUMO+2	0.226
				HOMO-1	→	LUMO+1	0.231
				HOMO-1	→	LUMO+2	0.424
				HOMO	→	LUMO	-0.129
7	3.2465	381.9	0.0409	HOMO-2	→	LUMO+2	0.477
				HOMO-1	→	LUMO+1	-0.477
8	3.2467	381.88	0.0408	HOMO-2	→	LUMO+1	0.479
				HOMO-1	→	LUMO+2	0.476
9	3.3365	371.6	0.0875	HOMO-2	→	LUMO+1	0.234
				HOMO-2	→	LUMO+2	0.417
				HOMO-1	→	LUMO+1	0.414
				HOMO-1	→	LUMO+2	-0.233
				HOMO	→	LUMO+3	-0.155
10	3.6173	342.75	0.0055	HOMO	→	LUMO+3	0.670

Table S25: Spin-orbit coupling of the first 10 singlet and triplet excited states of ***fac*-bzq3**, with the energy of each state in eV provided in parentheses. Root-square-sum (RSS) matrix values reported in cm^{-1}.

	$\mathbf{T}_1$ (2.52)	$\mathbf{T}_2$ (2.52)	$\mathbf{T}_3$ (2.52)	$\mathbf{T}_4$ (2.78)	$\mathbf{T}_5$ (2.80)	$\mathbf{T}_6$ (2.80)	$\mathbf{T}_7$ (2.98)	$\mathbf{T}_8$ (3.00)	$\mathbf{T}_9$ (3.00)	$\mathbf{T}_{10}$ (3.14)
$\mathbf{S}_0$ (0.00)	87.99	89.58	50.72	138.96	286.66	286.52	221.53	188.17	187.36	257.39
$\mathbf{S}_1$ (2.99)	303.72	302.72	48.23	1.76	201.99	201.11	62.49	529.52	529.24	608.03
$\mathbf{S}_2$ (3.06)	196.59	205.32	285.23	12.89	134.23	156.25	471.79	266.40	252.22	522.23
$\mathbf{S}_3$ (3.06)	205.01	197.65	288.05	12.59	156.77	134.32	468.58	256.23	270.18	525.18
$\mathbf{S}_4$ (3.15)	81.05	248.79	93.87	764.70	127.98	213.89	342.38	292.74	305.48	336.49
$\mathbf{S}_5$ (3.15)	248.84	80.36	92.04	764.45	214.76	127.64	343.58	304.89	295.36	181.97
$\mathbf{S}_6$ (3.20)	69.00	71.15	276.42	106.14	513.38	506.88	402.16	345.20	345.57	235.11
$\mathbf{S}_7$ (3.24)	170.71	86.32	31.41	80.70	504.33	478.56	70.72	359.72	375.61	396.20
$\mathbf{S}_8$ (3.24)	85.35	171.67	30.45	79.11	472.35	510.34	70.37	372.75	361.47	576.88
$\mathbf{S}_9$ (3.33)	51.41	52.79	3.44	168.77	431.50	431.46	7.53	289.17	287.47	311.55
$\mathbf{S}_{10}$ (3.61)	122.19	121.29	8.38	26.22	65.43	64.46	32.14	260.90	259.88	68.43

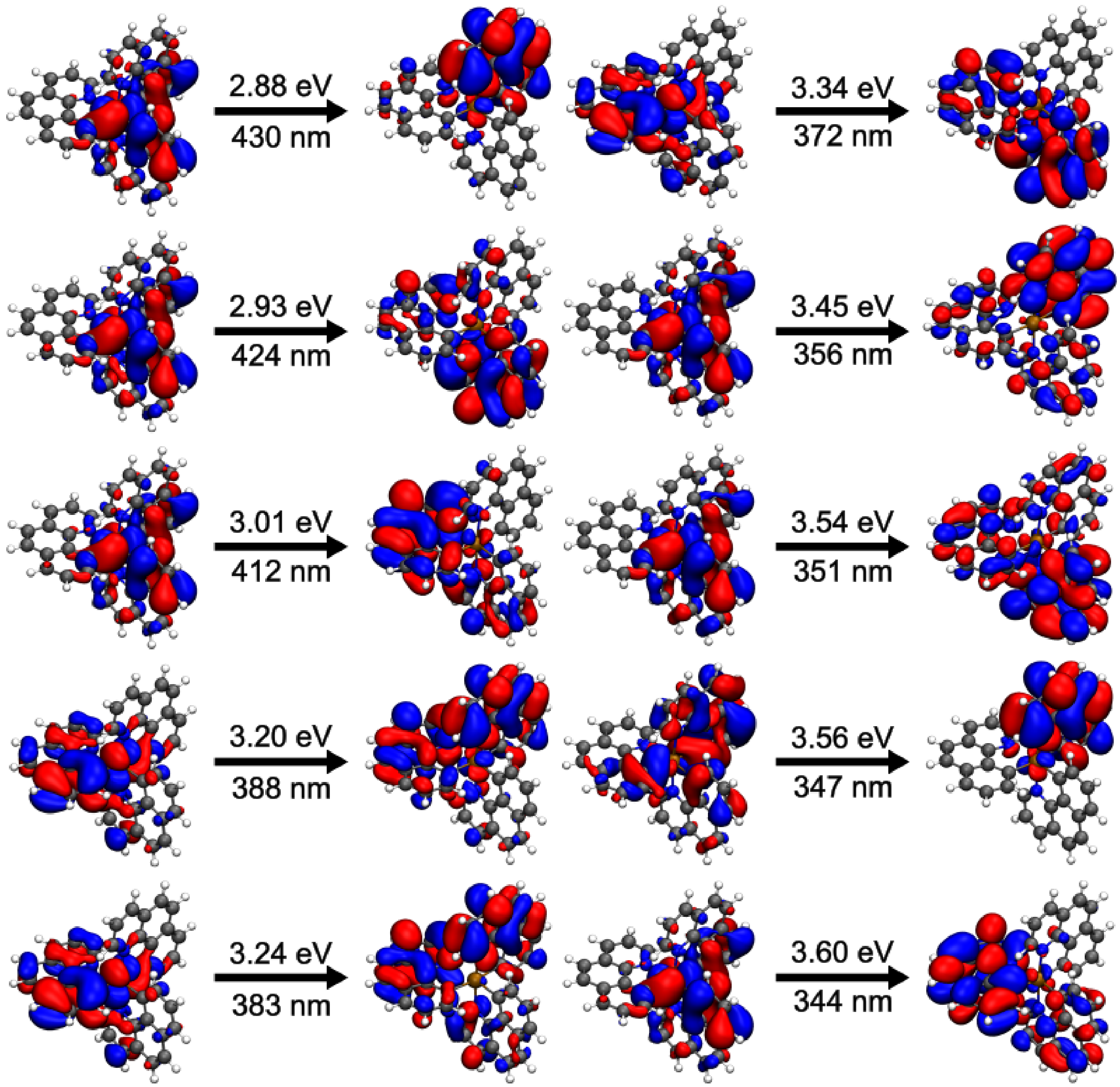

Figure S48: First 10 singlet TD-DFT transitions of ***mer*-bzq3** described as natural transition orbitals (NTOs) (Isovalue ±0.02). Calculated at the PBE0-D3/LANL2DZ[Ir]+6-311G(d,p)[C,H,N]/PCM(MeCN) level of theory.

Table S26: First 10 TD-DFT singlet transitions of ***mer*-bzq3** calculated at the PBE0-D3/LANL2DZ[Ir]+6-311G(d,p)[C,H,N]/PCM(MeCN) level of theory.

Excitation	Energy (eV)	λ (nm)	f	Transitions			
1	2.8817	430.24	0.0116	HOMO	→	LUMO	0.634
				HOMO	→	LUMO+1	0.277
2	2.9251	423.86	0.0376	HOMO	→	LUMO	-0.265
				HOMO	→	LUMO+1	0.584
				HOMO	→	LUMO+2	-0.237
3	3.0078	412.21	0.0034	HOMO	→	LUMO+1	0.242
				HOMO	→	LUMO+2	0.642
4	3.1954	388.01	0.0228	HOMO-1	→	LUMO	0.667
				HOMO-1	→	LUMO+2	-0.152
5	3.2364	383.09	0.0378	HOMO-1	→	LUMO+1	0.670
				HOMO-1	→	LUMO+2	0.149
6	3.3368	371.57	0.0481	HOMO-1	→	LUMO	0.134
				HOMO-1	→	LUMO+1	-0.128
				HOMO-1	→	LUMO+2	0.608
				HOMO	→	LUMO+2	-0.125
				HOMO	→	LUMO+4	-0.108
				HOMO	→	LUMO+5	-0.170
7	3.4781	356.47	0.0366	HOMO-3	→	LUMO	-0.122
				HOMO-2	→	LUMO	-0.111
				HOMO	→	LUMO+3	0.604
				HOMO	→	LUMO+4	0.251
8	3.5372	350.51	0.0679	HOMO-3	→	LUMO	-0.288
				HOMO-2	→	LUMO	-0.210
				HOMO-2	→	LUMO+1	-0.126
				HOMO-1	→	LUMO+2	0.129
				HOMO	→	LUMO+3	-0.242
				HOMO	→	LUMO+4	0.392
				HOMO	→	LUMO+5	0.292
9	3.5776	346.56	0.0771	HOMO-2	→	LUMO	0.473
				HOMO-2	→	LUMO+1	0.311
				HOMO	→	LUMO+4	0.254
				HOMO	→	LUMO+5	0.211
10	3.6001	344.39	0.0225	HOMO	→	LUMO+3	0.214
				HOMO		LUMO+4	-0.358
				HOMO		LUMO+5	0.538

Table S27: Spin-orbit coupling of the first 10 singlet and triplet excited states of ***mer*-bzq3**, with the energy of each state in eV provided in parentheses. Root-square-sum (RSS) matrix values reported in cm^{-1}.

	$\mathbf{T}_1$ (2.43)	$\mathbf{T}_2$ (2.53)	$\mathbf{T}_3$ (2.58)	$\mathbf{T}_4$ (2.72)	$\mathbf{T}_5$ (2.73)	$\mathbf{T}_6$ (2.90)	$\mathbf{T}_7$ (2.98)	$\mathbf{T}_8$ (3.01)	$\mathbf{T}_9$ (3.13)	$\mathbf{T}_{10}$ (3.18)
$\mathbf{S}_0$ (0.00)	27.44	42.93	21.47	301.41	284.94	216.18	187.22	50.51	58.80	314.17
$\mathbf{S}_1$ (2.87)	81.69	318.70	86.54	92.39	129.58	193.08	127.23	69.59	571.39	746.05
$\mathbf{S}_2$ (2.92)	321.29	78.37	105.96	125.02	167.21	158.65	311.13	394.64	142.44	211.98
$\mathbf{S}_3$ (3.00)	159.91	19.53	228.50	23.41	89.15	398.98	210.45	332.61	22.07	183.05
$\mathbf{S}_4$ (3.19)	109.20	292.70	146.44	308.91	544.18	188.39	357.01	153.16	454.57	82.89
$\mathbf{S}_5$ (3.23)	87.03	200.41	152.22	494.16	124.25	281.47	405.41	136.40	289.49	209.23
$\mathbf{S}_6$ (3.33)	207.53	6.09	121.91	330.76	336.98	250.88	362.22	45.13	40.83	37.74
$\mathbf{S}_7$ (3.47)	34.84	84.49	49.78	133.76	116.77	22.82	59.14	49.12	90.74	198.94
$\mathbf{S}_8$ (3.53)	67.68	173.56	63.16	176.56	293.87	132.95	161.28	77.33	135.41	357.32
$\mathbf{S}_9$ (3.58)	23.52	98.33	32.27	96.05	32.66	98.15	101.76	50.21	113.01	123.32
$\mathbf{S}_{10}$ (3.59)	17.32	24.34	137.72	34.50	77.48	134.17	60.17	63.95	36.73	84.03

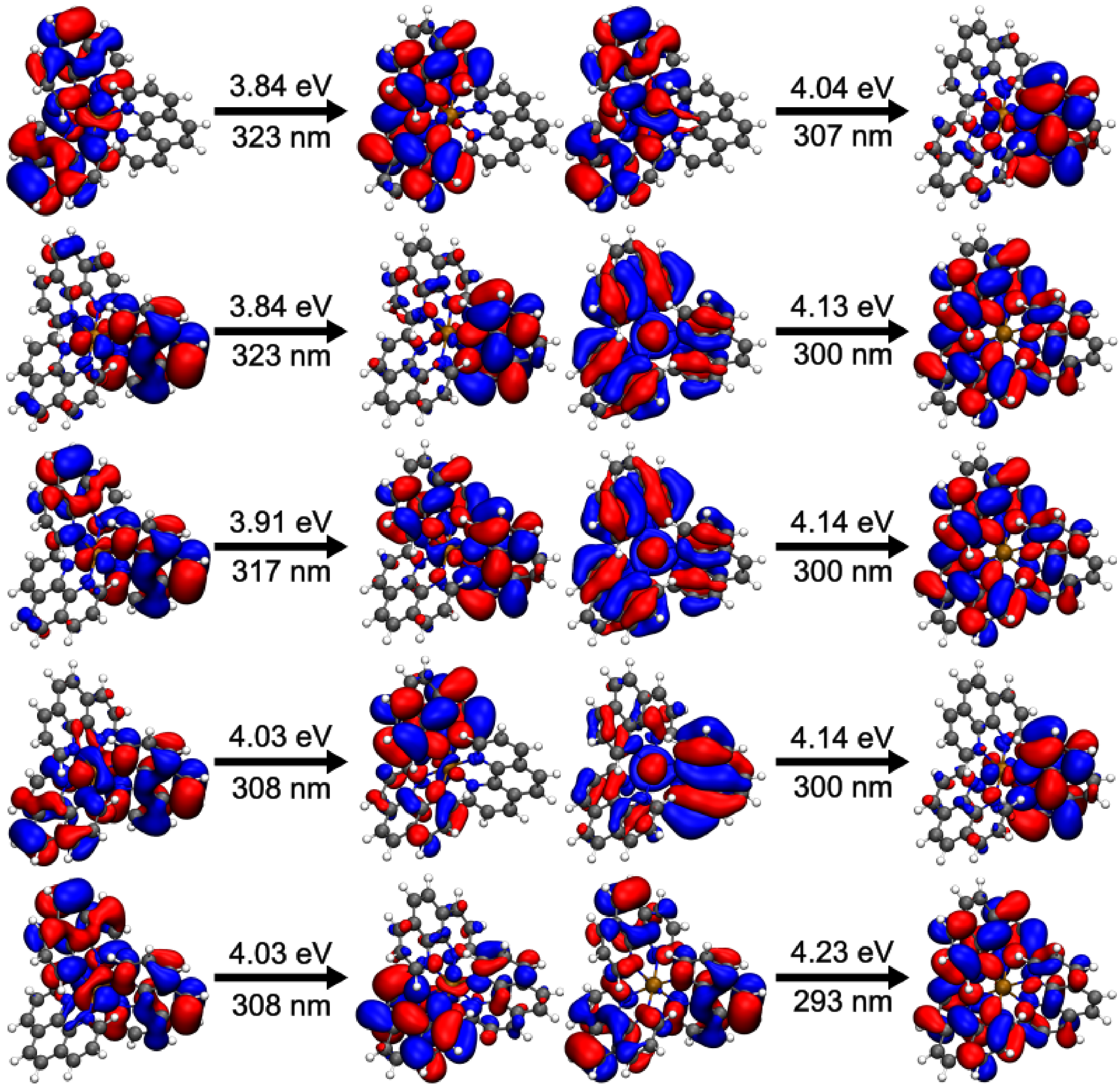

Figure S49: First 10 singlet TD-DFT transitions of **phen3** described as natural transition orbitals (NTOs) (Isovalue ±0.02). Calculated at the PBE0-D3/LANL2DZ[Ir]+6-311G(d,p)[C,H,N]/PCM(MeCN) level of theory.

Table S28: First 10 TD-DFT singlet transitions of **phen3** calculated at the PBE0-D3/LANL2DZ[Ir]+6-311G(d,p)[C,H,N]/PCM(MeCN) level of theory.

Excitation	Energy (eV)	λ (nm)	f	Transitions			
1	3.8409	322.8	0.0879	HOMO-5	→	LUMO+3	0.101
				HOMO-3	→	LUMO+4	-0.104
				HOMO-2	→	LUMO+1	0.209
				HOMO-1	→	LUMO	-0.358
				HOMO-1	→	LUMO+1	-0.229
				HOMO-1	→	LUMO+2	-0.155
				HOMO	→	LUMO	0.359
				HOMO	→	LUMO+1	-0.181
				HOMO	→	LUMO+2	0.225
2	3.8426	322.66	0.0881	HOMO-3	→	LUMO+5	0.106
				HOMO-2	→	LUMO+2	-0.204
				HOMO-1	→	LUMO	0.337
				HOMO-1	→	LUMO+1	0.165
				HOMO-1	→	LUMO+2	-0.269
				HOMO	→	LUMO	0.316
				HOMO	→	LUMO+1	-0.261
				HOMO	→	LUMO+2	-0.192
3	3.9097	317.12	0	HOMO-5	→	LUMO+4	-0.117
				HOMO-4	→	LUMO+5	0.118
				HOMO-3	→	LUMO+3	0.185
				HOMO-2	→	LUMO	-0.246
				HOMO-1	→	LUMO+1	0.379
				HOMO-1	→	LUMO+2	0.197
				HOMO	→	LUMO+1	-0.193
				HOMO	→	LUMO+2	0.379
4	4.027	307.89	0.0006	HOMO-1	→	LUMO	0.253
				HOMO-1	→	LUMO+2	0.252
				HOMO	→	LUMO	0.411
				HOMO	→	LUMO+1	0.379
				HOMO	→	LUMO+2	0.134
5	4.0309	307.58	0.0009	HOMO-1	→	LUMO	-0.365
				HOMO-1	→	LUMO+1	0.410
				HOMO	→	LUMO	0.195
				HOMO	→	LUMO+1	0.248
				HOMO	→	LUMO+2	-0.257
6	4.0353	307.25	0.0028	HOMO-1	→	LUMO+1	-0.105
				HOMO-1	→	LUMO+2	0.496
				HOMO	→	LUMO	0.110
				HOMO	→	LUMO+1	-0.318
				HOMO	→	LUMO+2	-0.314

7	4.1325	300.02	0.0007	HOMO-5	→	LUMO+1	-0.211
				HOMO-4	→	LUMO+2	0.175
				HOMO-3	→	LUMO	0.529
				HOMO-2	→	LUMO+3	0.157
				HOMO-1	→	LUMO+4	-0.170
				HOMO-1	→	LUMO+5	-0.154
				HOMO	→	LUMO+4	0.178
				HOMO	→	LUMO+5	-0.134
8	4.1388	299.57	0.0017	HOMO-5	→	LUMO	-0.210
				HOMO-5	→	LUMO+2	-0.105
				HOMO-4	→	LUMO+1	0.121
				HOMO-3	→	LUMO+1	0.463
				HOMO-2	→	LUMO+4	0.141
				HOMO-1	→	LUMO+3	-0.261
				HOMO-1	→	LUMO+5	-0.101
				HOMO	→	LUMO+3	0.197
				HOMO	→	LUMO+4	-0.115
				HOMO	→	LUMO+5	0.108
9	4.1396	299.51	0.0017	HOMO-4	→	LUMO	0.187
				HOMO-4	→	LUMO+2	-0.151
				HOMO-3	→	LUMO+2	0.463
				HOMO-2	→	LUMO+5	0.137
				HOMO-1	→	LUMO+3	-0.186
				HOMO-1	→	LUMO+5	0.138
				HOMO	→	LUMO+3	-0.254
				HOMO	→	LUMO+5	0.139
10	4.2326	292.93	0.0004	HOMO-2	→	LUMO	0.619
				HOMO-1	→	LUMO+1	0.198
				HOMO	→	LUMO+1	-0.108
				HOMO	→	LUMO+2	0.158

Table S29: Spin-orbit coupling of the first 10 singlet and triplet excited states of **phen3**, with the energy of each state in eV provided in parentheses. Root-square-sum (RSS) matrix values reported in cm^{-1}.

	$\mathbf{T}_1$ (2.76)	$\mathbf{T}_2$ (2.76)	$\mathbf{T}_3$ (2.77)	$\mathbf{T}_4$ (3.09)	$\mathbf{T}_5$ (3.09)	$\mathbf{T}_6$ (3.09)	$\mathbf{T}_7$ (3.43)	$\mathbf{T}_8$ (3.43)	$\mathbf{T}_9$ (3.43)	$\mathbf{T}_{10}$ (3.83)
$\mathbf{S}_0$ (0.00)	60.29	61.50	20.30	7.56	15.05	16.85	51.12	45.50	31.11	6.22
$\mathbf{S}_1$ (3.84)	58.02	49.26	61.68	7.82	6.66	5.89	56.28	65.10	35.06	3.23
$\mathbf{S}_2$ (3.84)	48.22	57.70	60.36	7.65	8.43	8.53	35.99	31.46	75.17	3.00
$\mathbf{S}_3$ (3.91)	66.18	65.90	35.71	7.05	7.50	6.28	34.40	34.69	31.13	2.70
$\mathbf{S}_4$ (4.02)	55.67	69.78	80.53	7.37	148.84	88.73	105.00	91.99	44.76	3.69
$\mathbf{S}_5$ (4.03)	78.21	53.98	69.17	69.18	84.36	127.00	86.96	75.41	92.54	2.51
$\mathbf{S}_6$ (4.03)	63.32	75.56	28.47	136.95	32.53	73.87	78.21	98.25	106.25	9.53
$\mathbf{S}_7$ (4.13)	28.29	30.20	5.40	34.93	162.46	139.64	39.82	32.66	15.12	14.69
$\mathbf{S}_8$ (4.13)	42.84	40.58	42.27	144.50	94.50	86.99	47.84	67.07	37.36	43.70
$\mathbf{S}_9$ (4.13)	39.85	43.94	39.27	130.37	93.94	119.49	36.04	34.03	84.70	41.93
$\mathbf{S}_{10}$ (4.23)	39.45	39.16	13.88	48.33	15.69	30.01	60.56	37.35	29.61	5.77

Table S30: Reorganization energies (λ) of populating the T_{1a} minimum from the S_1 at the ground state geometry (S_1/S_0).

	S_1/S_0 (eV)	T_{1a} (eV)	λ (eV)
***fac*-ppy3**	3.13	2.60	0.53
***mer*-ppy3**	3.01	2.39	0.64
***m*-ppy2**	2.71	2.35	0.37
***f*-ppy2**	2.79	2.32	0.47
**bpy2**	3.13	2.66	0.40
**bpy3**	4.14	2.83	1.31
***fac*-bzq3**	3.00	2.51	0.50
***mer*-bzq3**	2.88	2.44	0.44
**phen3**	3.84	2.79	1.04

Triplet-to-triplet scans

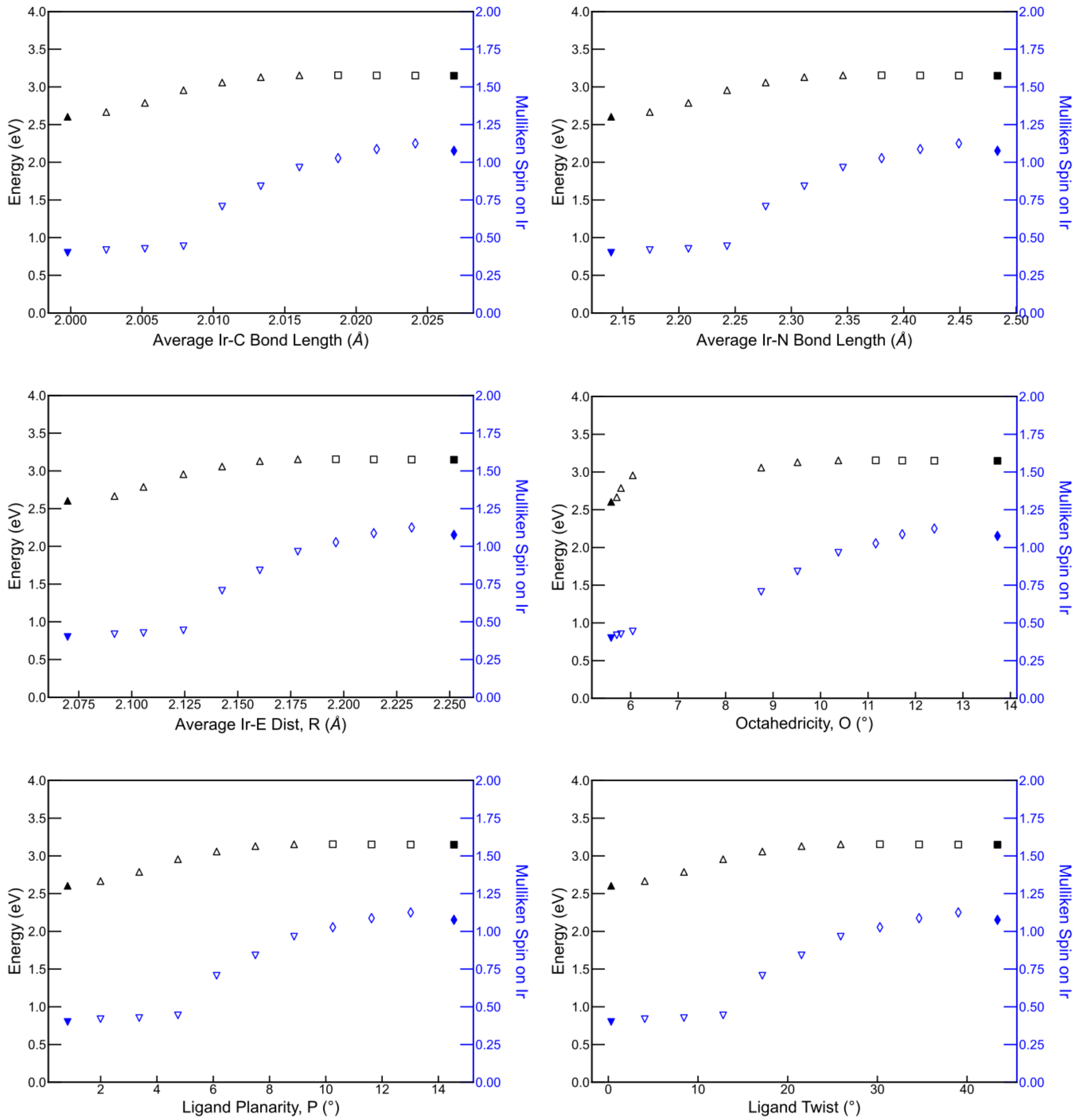

Figure S50: Constrained optimizations along the triplet manifold of ***fac*-ppy3** calculated at the PBE0-D3/LANL2DZ[Ir]+6-311G(d,p)[C,H,N]/PCM(MeCN) level of theory. Triangles represent triplets with CT character (spin = inverted triangle) while squares are those with MC character (spin = diamond).

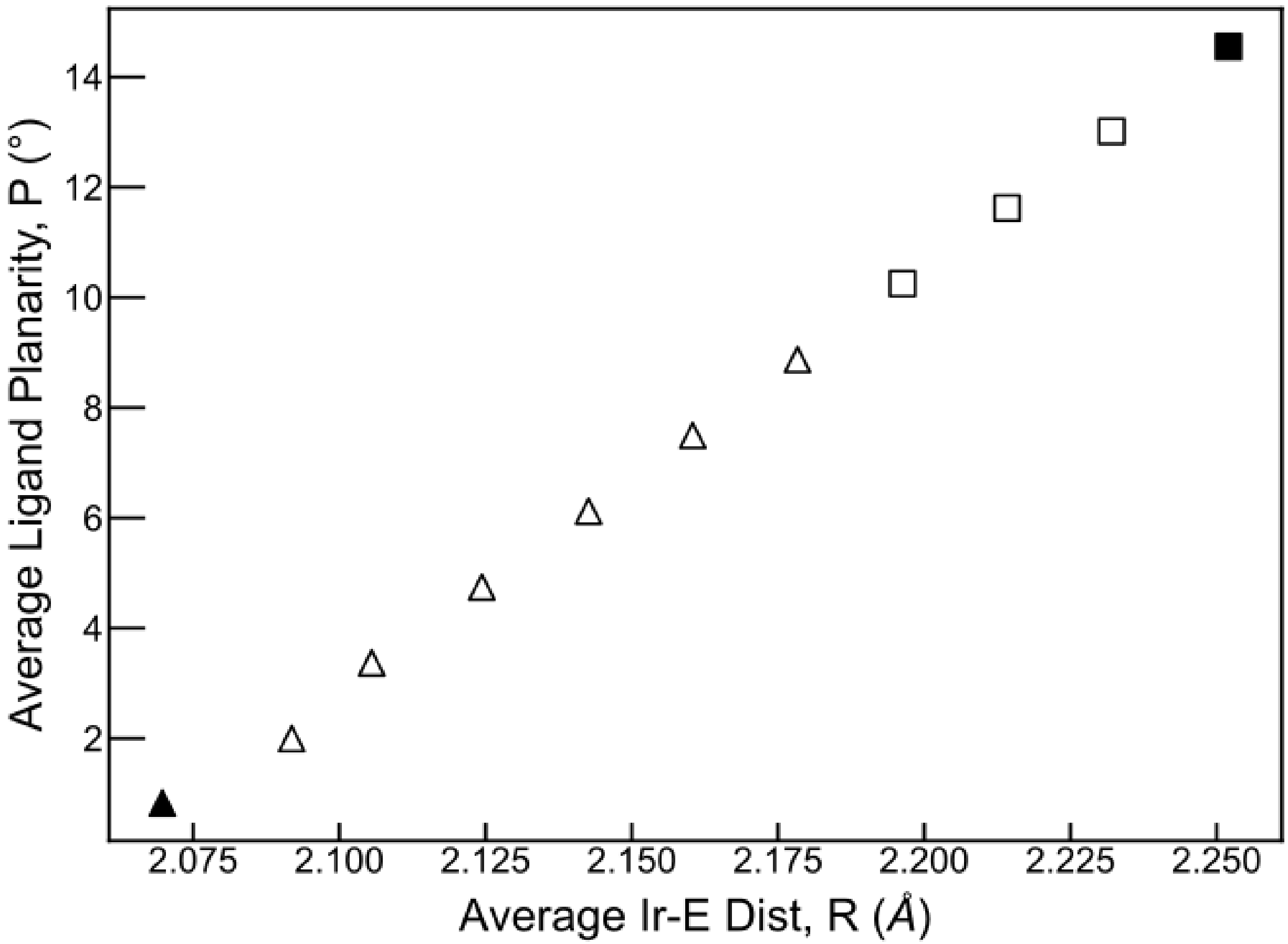

Figure S51: Constrained optimization scans projected onto a flat Structure-structure surface calculated at the PBE0-D3/LANL2DZ[Ir]+6-311G(d,p)[C,H,N]/PCM(MeCN) level of theory. Triangles represent triplets with MLCT character, and squares represent those with MC character.

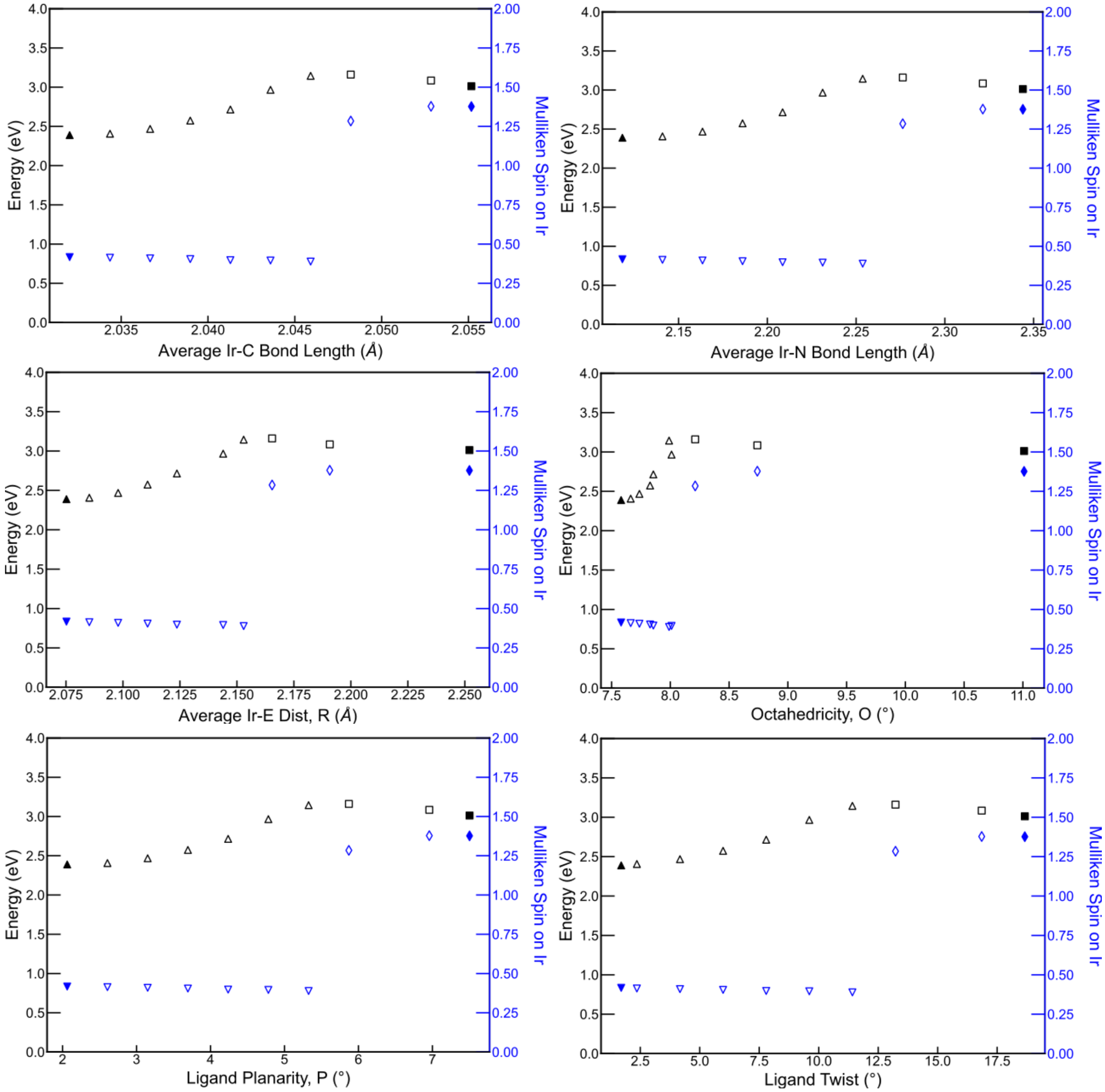

Figure S52: Constrained optimizations along the triplet manifold of ***mer*-ppy3** calculated at the PBE0-D3/LANL2DZ[Ir]+6-311G(d,p)[C,H,N]/PCM(MeCN) level of theory. Triangles represent triplets with CT character (spin = inverted triangle) while squares are those with MC character (spin = diamond).

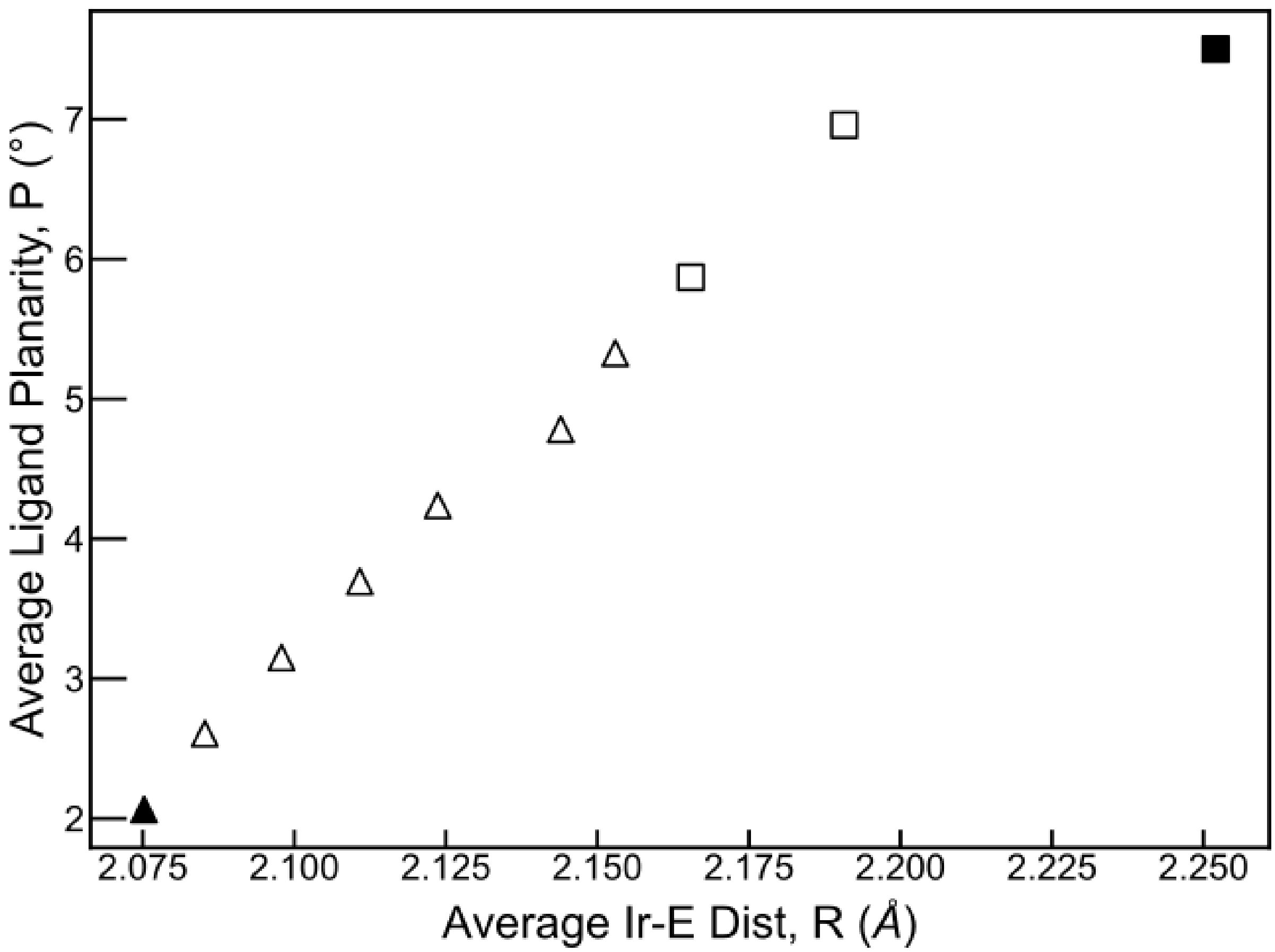

Figure S53: Constrained optimization scans projected onto a flat Structure-structure surface calculated at the PBE0-D3/LANL2DZ[Ir]+6-311G(d,p)[C,H,N]/PCM(MeCN) level of theory. Triangles represent triplets with MLCT character, and squares represent those with MC character.

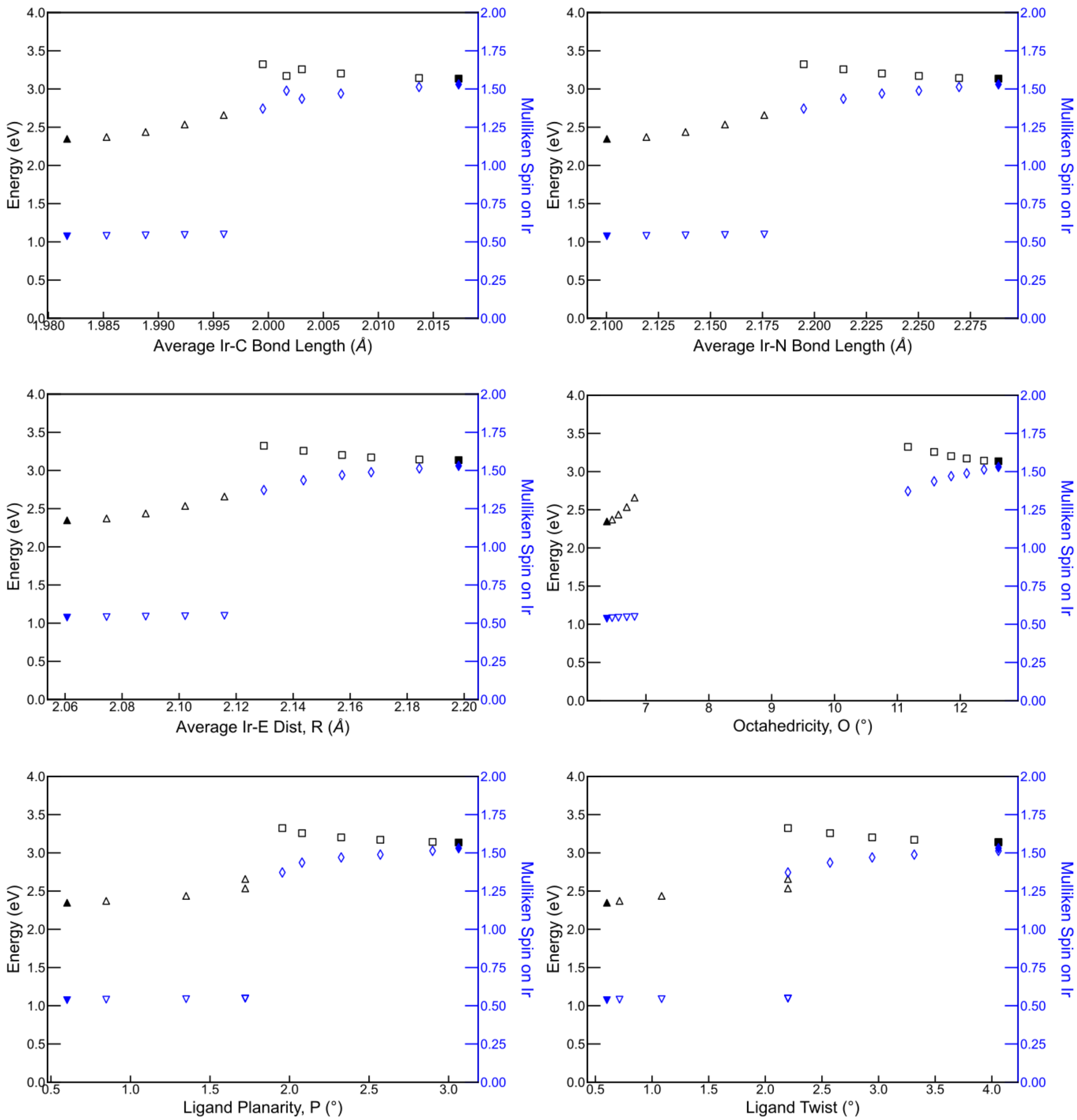

Figure S54: Constrained optimizations along the triplet manifold of ***m*-ppy2** calculated at the PBE0-D3/LANL2DZ[Ir]+6-311G(d,p)[C,H,N]/PCM(MeCN) level of theory. Triangles represent triplets with CT character (spin = inverted triangle) while squares are those with MC character (spin = diamond).

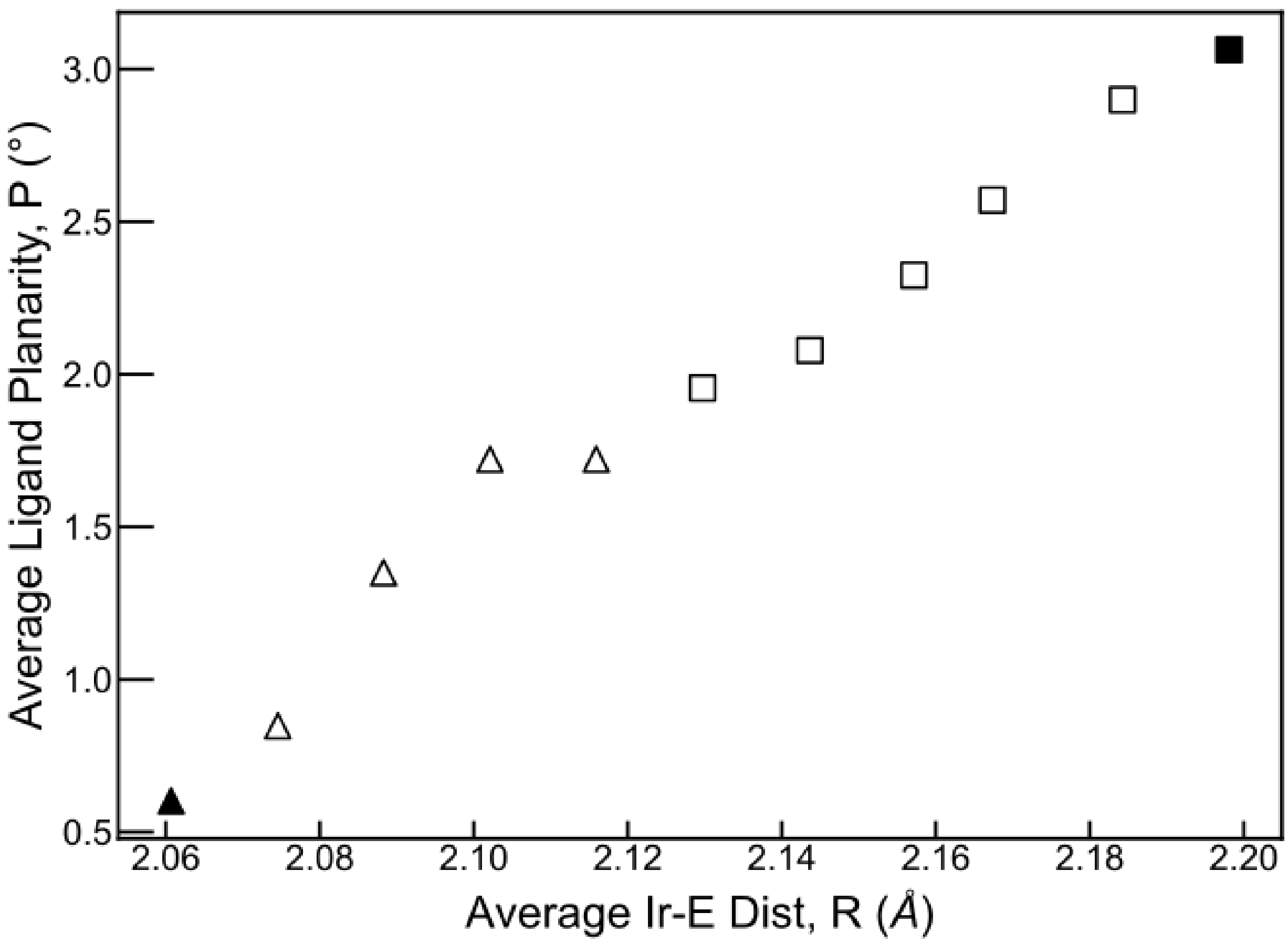

Figure S55: Constrained optimization scans projected onto a flat Structure-structure surface calculated at the PBE0-D3/LANL2DZ[Ir]+6-311G(d,p)[C,H,N]/PCM(MeCN) level of theory. Triangles represent triplets with MLCT character, and squares represent those with MC character.

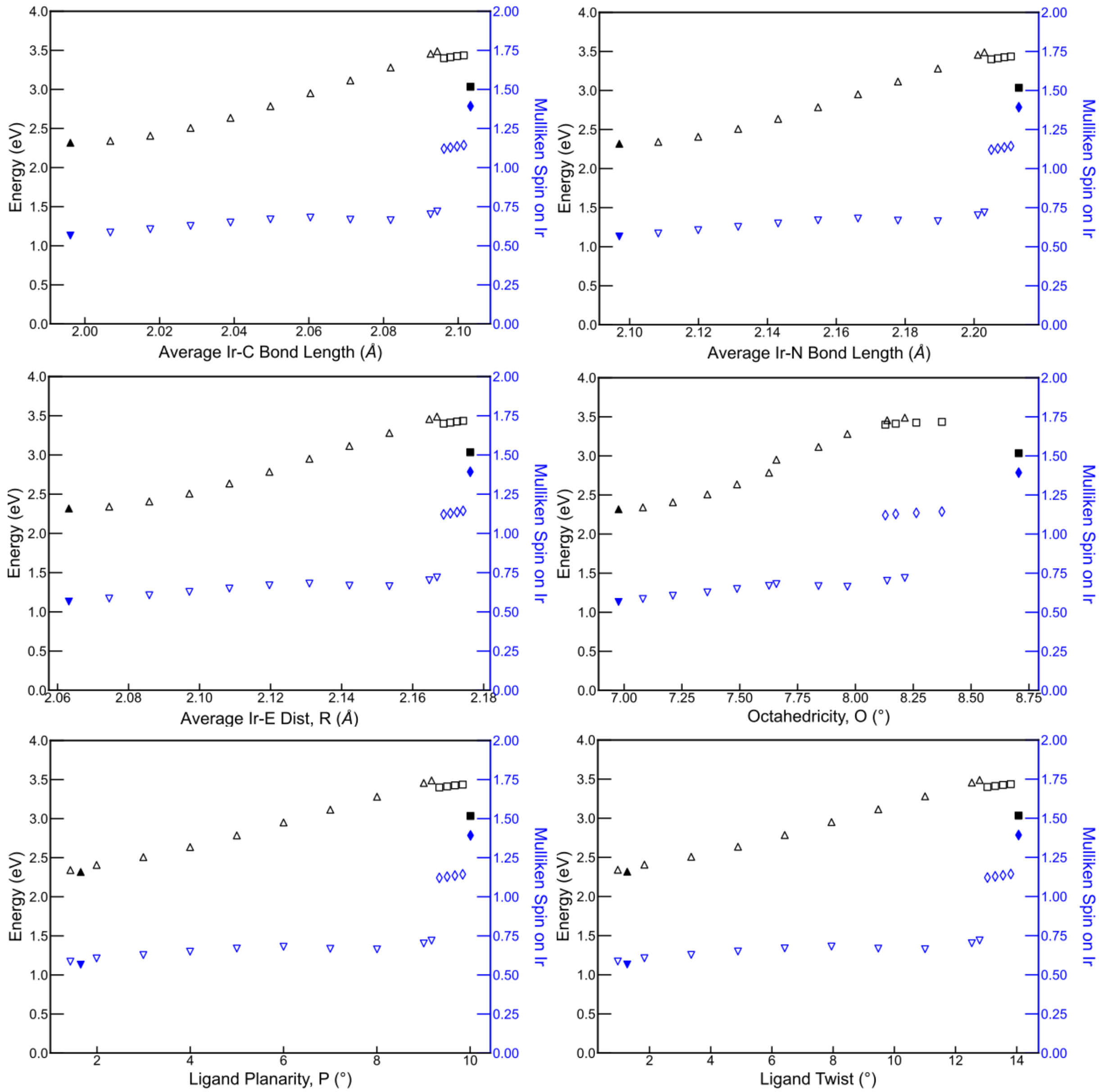

Figure S56: Constrained optimizations along the triplet manifold of ***f*-ppy2** calculated at the PBE0-D3/LANL2DZ[Ir]+6-311G(d,p)[C,H,N]/PCM(MeCN) level of theory. Triangles represent triplets with CT character (spin = inverted triangle) while squares are those with MC character (spin = diamond).

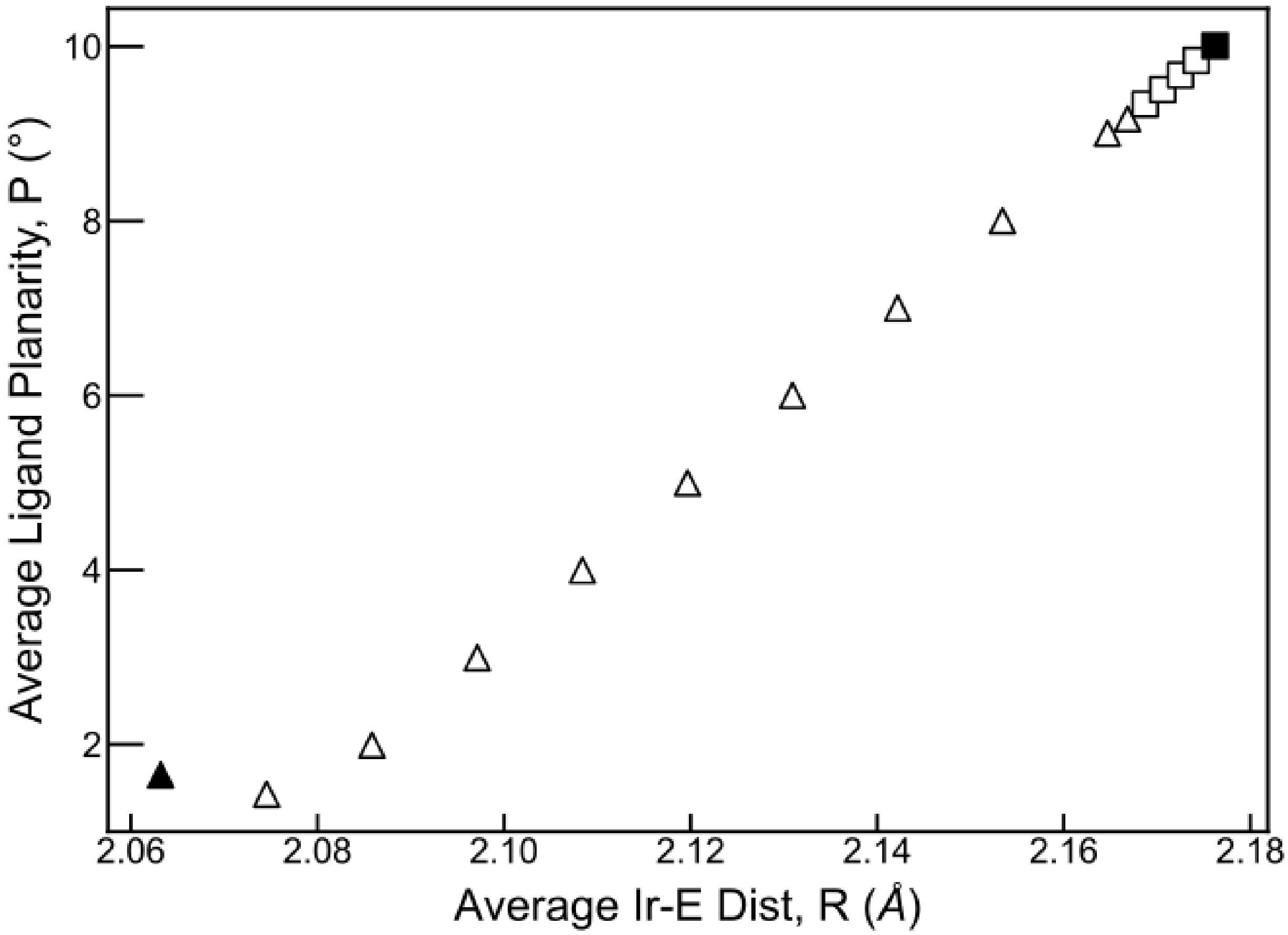

Figure S57: Constrained optimization scans projected onto a flat Structure-structure surface calculated at the PBE0-D3/LANL2DZ[Ir]+6-311G(d,p)[C,H,N]/PCM(MeCN) level of theory. Triangles represent triplets with MLCT character, and squares represent those with MC character.

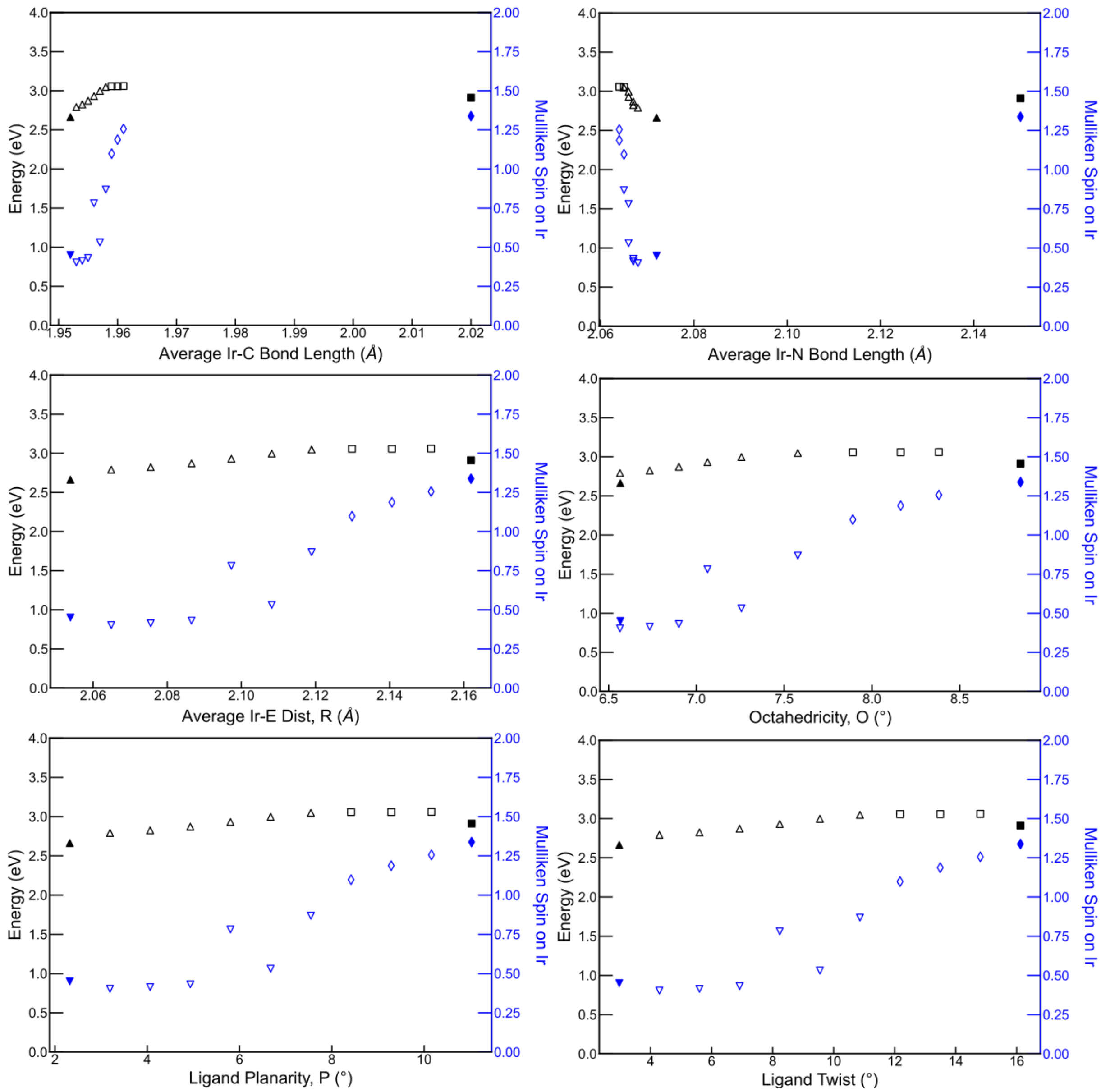

Figure S58: Constrained optimizations along the triplet manifold of **bpy2** calculated at the PBE0-D3/LANL2DZ[Ir]+6-311G(d,p)[C,H,N]/PCM(MeCN) level of theory. Triangles represent triplets with CT character (spin = inverted triangle) while squares are those with MC character (spin = diamond).

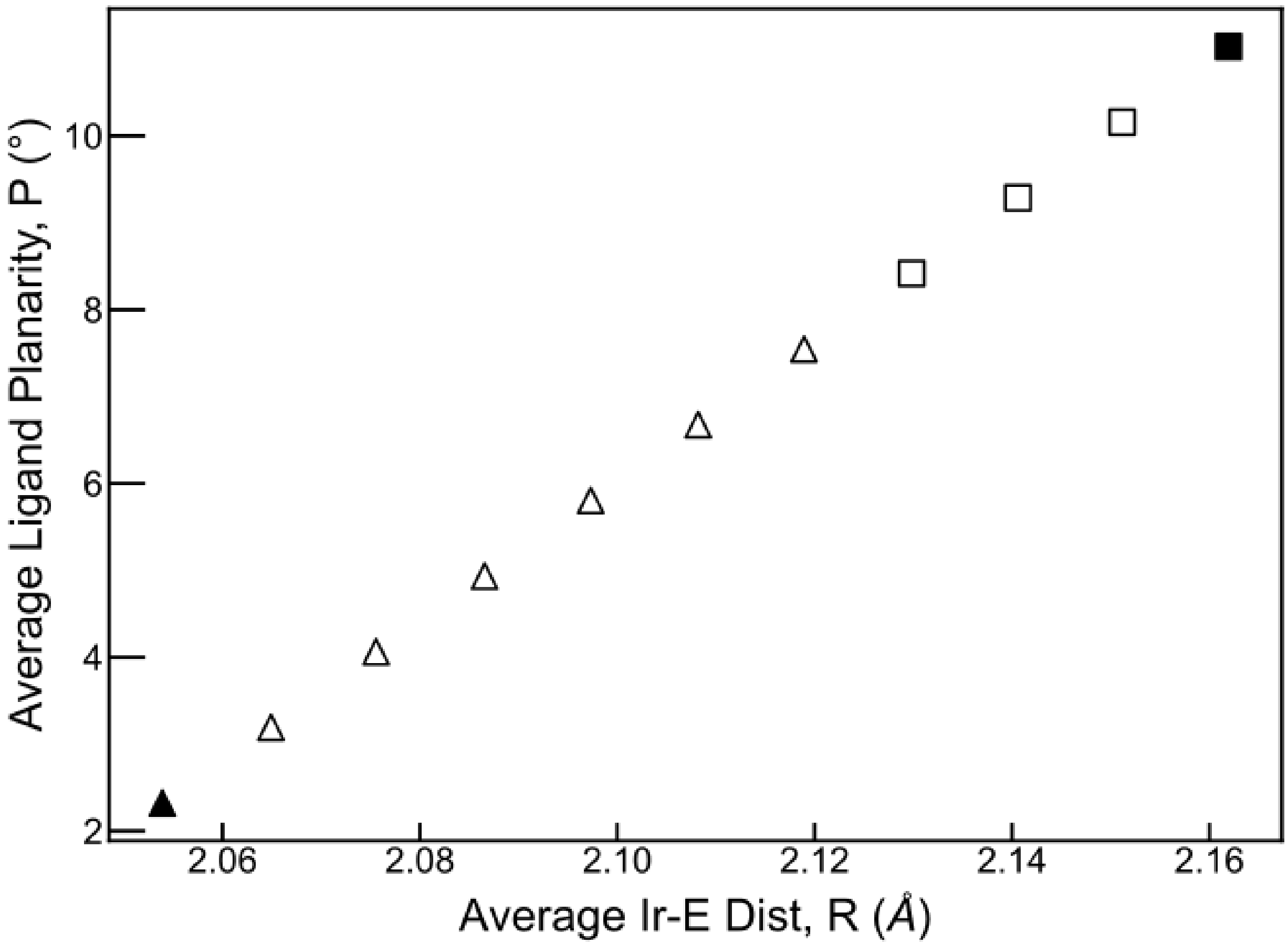

Figure S59: Constrained optimization scans projected onto a flat Structure-structure surface calculated at the PBE0-D3/LANL2DZ[Ir]+6-311G(d,p)[C,H,N]/PCM(MeCN) level of theory. Triangles represent triplets with MLCT character, and squares represent those with MC character.

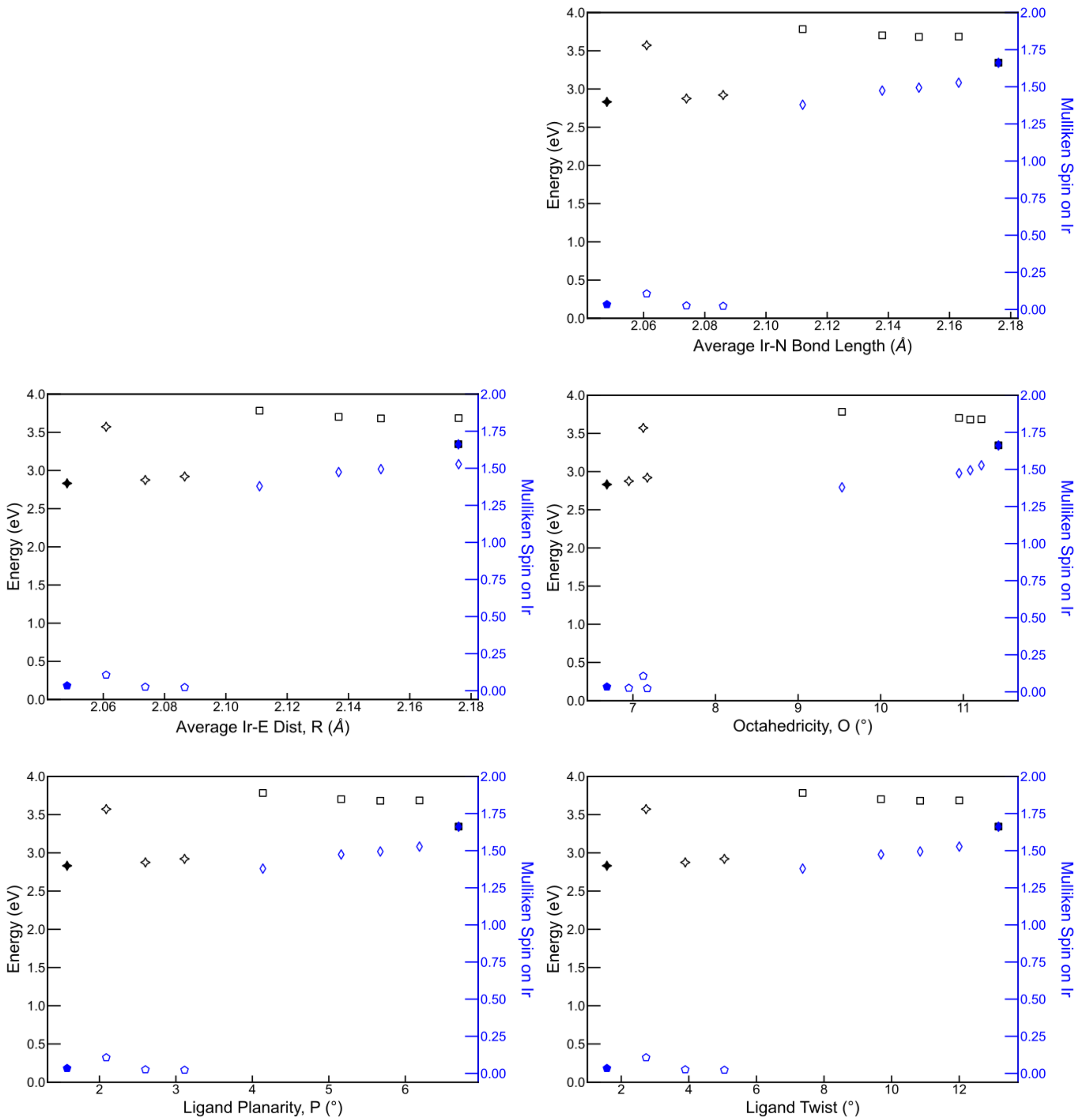

Figure S60: Constrained optimizations along the triplet manifold of **bpy3** calculated at the PBE0-D3/LANL2DZ[Ir]+6-311G(d,p)[C,H,N]/PCM(MeCN) level of theory. Stars represent triplets with LC character (spin = pentagon) while squares are those with MC character (spin = diamond).

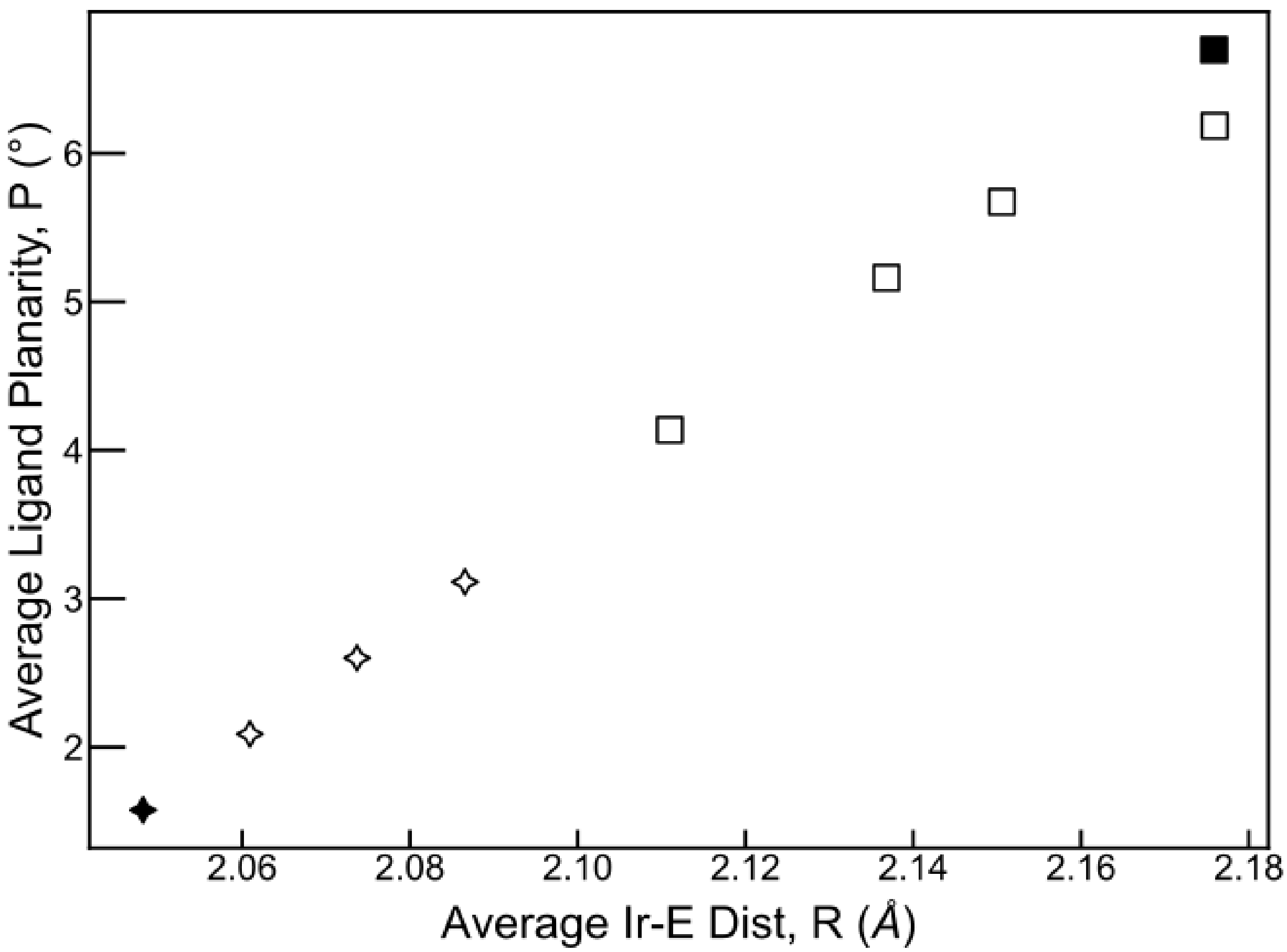

Figure S61: Constrained optimization scans projected onto a flat Structure-structure surface calculated at the PBE0-D3/LANL2DZ[Ir]+6-311G(d,p)[C,H,N]/PCM(MeCN) level of theory. Stars represent triplets with LC character, and squares represent those with MC character.

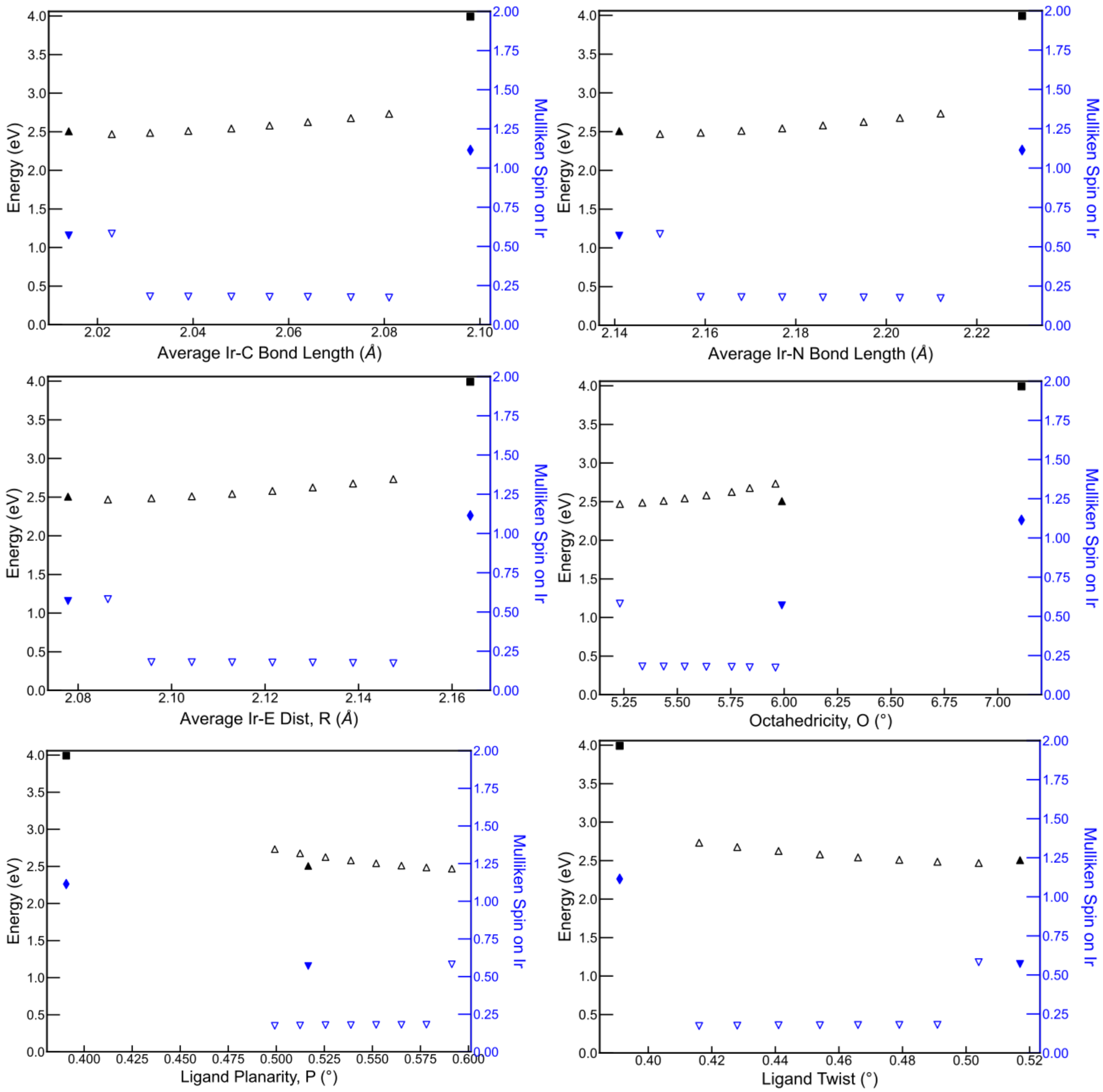

Figure S62: Constrained optimizations along the triplet manifold of ***fac*-bzq3** calculated at the PBE0-D3/LANL2DZ[Ir]+6-311G(d,p)[C,H,N]/PCM(MeCN) level of theory. Triangles represent triplets with CT character (spin = inverted triangle) while squares are those with MC character (spin = diamond).

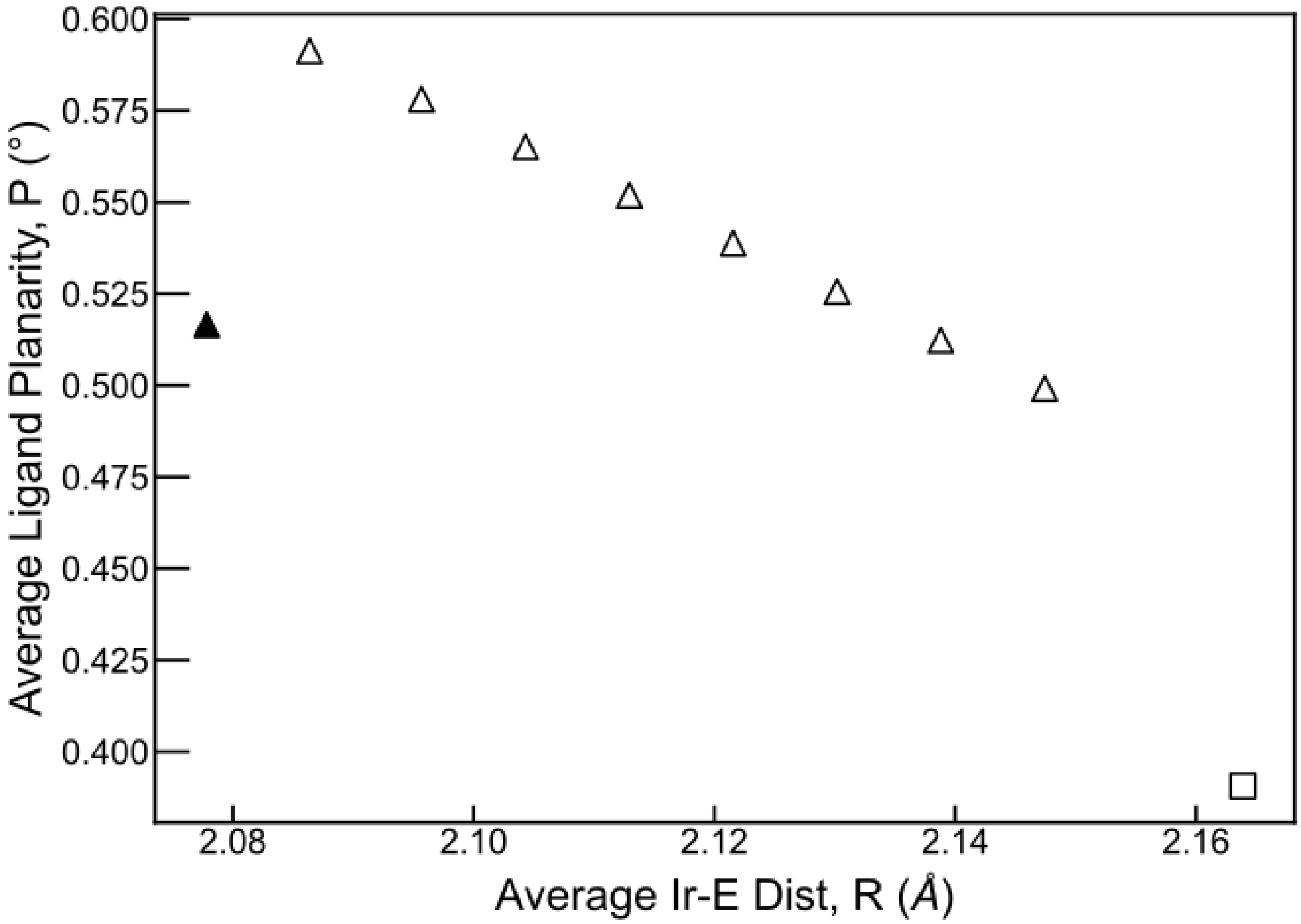

Figure S63: Constrained optimization scans projected onto a flat Structure-structure surface calculated at the PBE0-D3/LANL2DZ[Ir]+6-311G(d,p)[C,H,N]/PCM(MeCN) level of theory. Triangles represent triplets with MLCT character, and squares represent those with MC character.

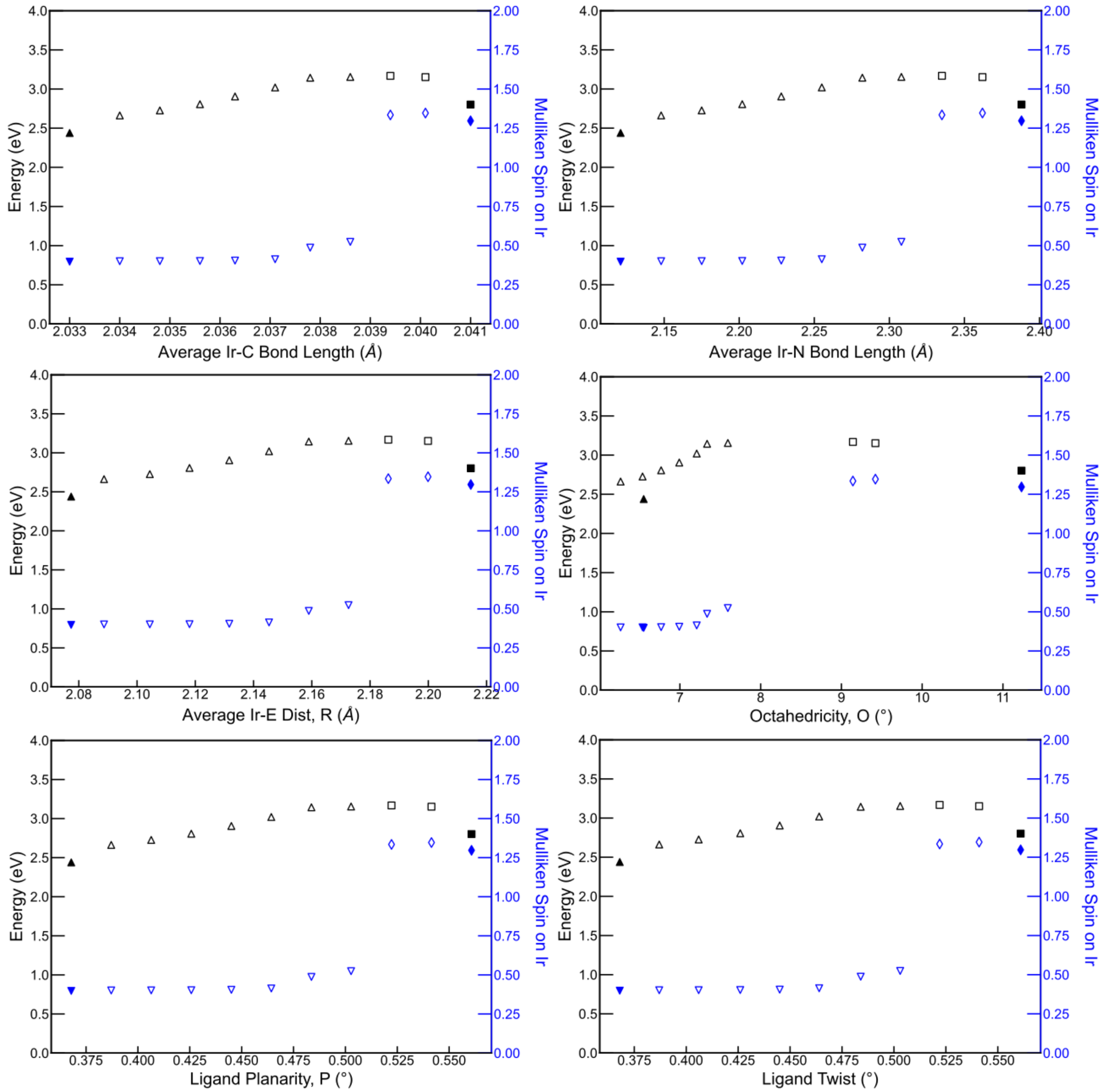

Figure S64: Constrained optimizations along the triplet manifold of ***mer*-bzq3** calculated at the PBE0-D3/LANL2DZ[Ir]+6-311G(d,p)[C,H,N]/PCM(MeCN) level of theory. Triangles represent triplets with CT character (spin = inverted triangle) while squares are those with MC character (spin = diamond).

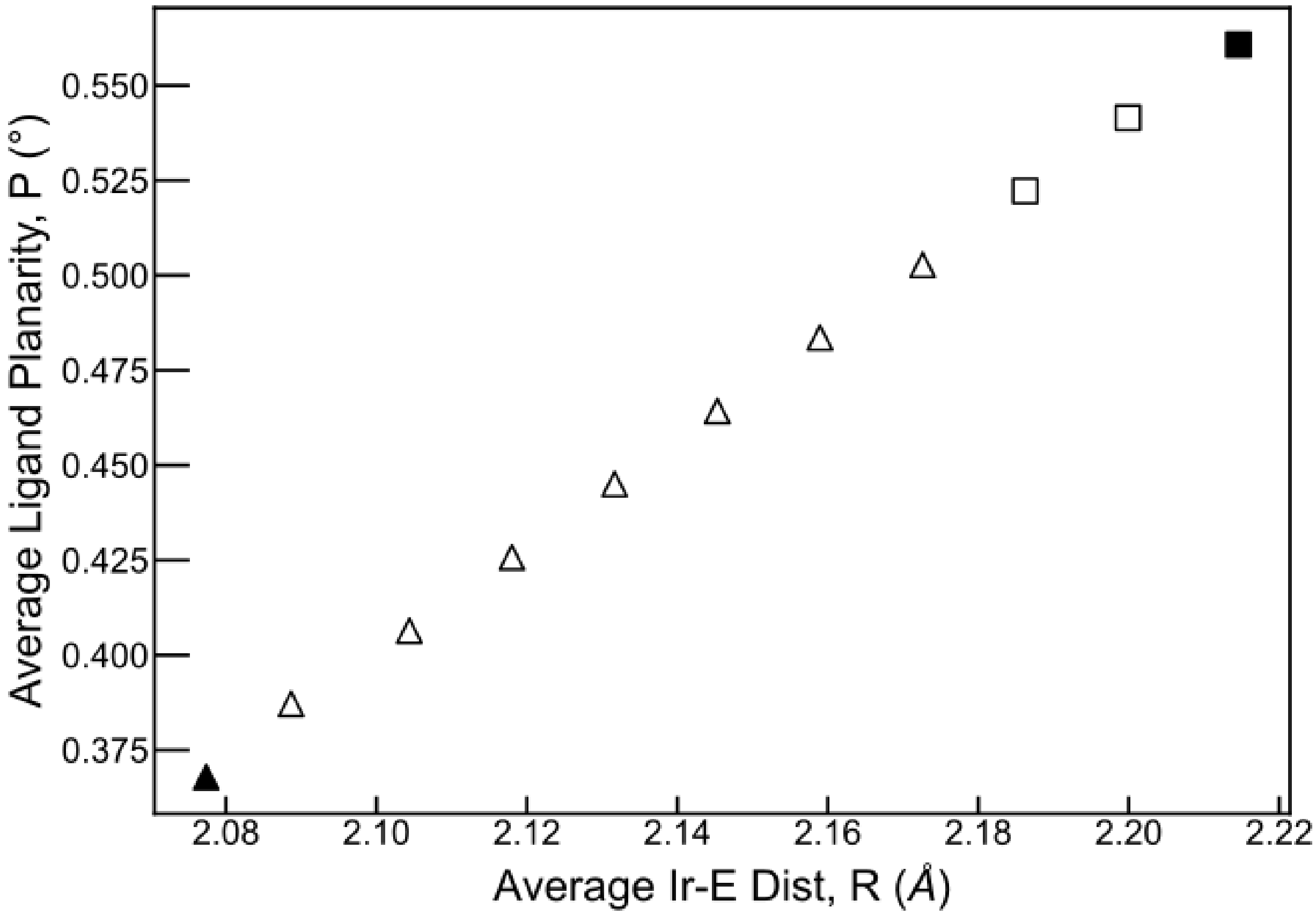

Figure S65: Constrained optimization scans projected onto a flat Structure-structure surface calculated at the PBE0-D3/LANL2DZ[Ir]+6-311G(d,p)[C,H,N]/PCM(MeCN) level of theory. Triangles represent triplets with MLCT character, and squares represent those with MC character.

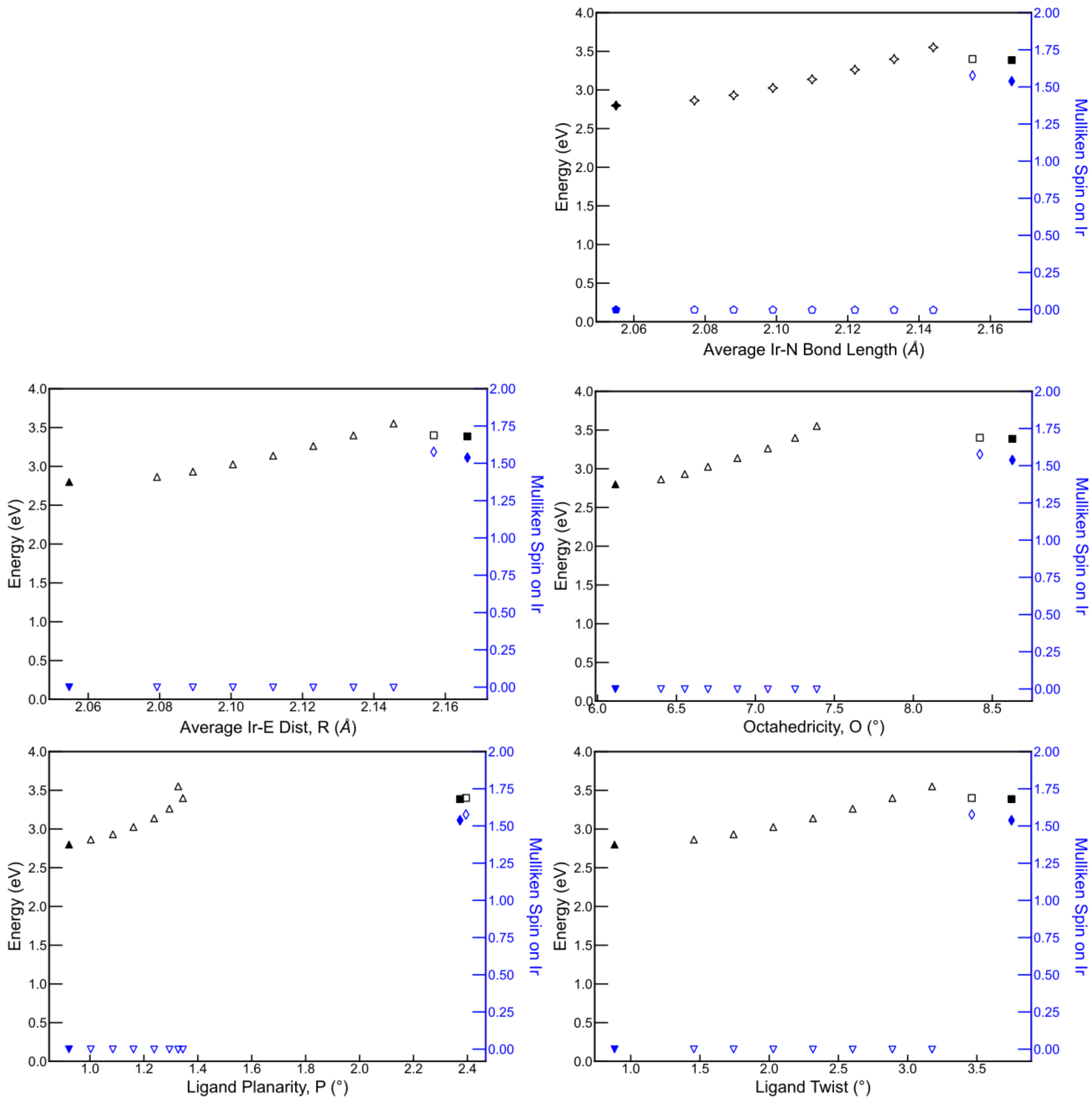

Figure S66: Constrained optimizations along the triplet manifold of **phen3** calculated at the PBE0-D3/LANL2DZ[Ir]+6-311G(d,p)[C,H,N]/PCM(MeCN) level of theory. Stars represent those triplets with LC character (spin = pentagon) while squares are those with MC character (spin = diamond).

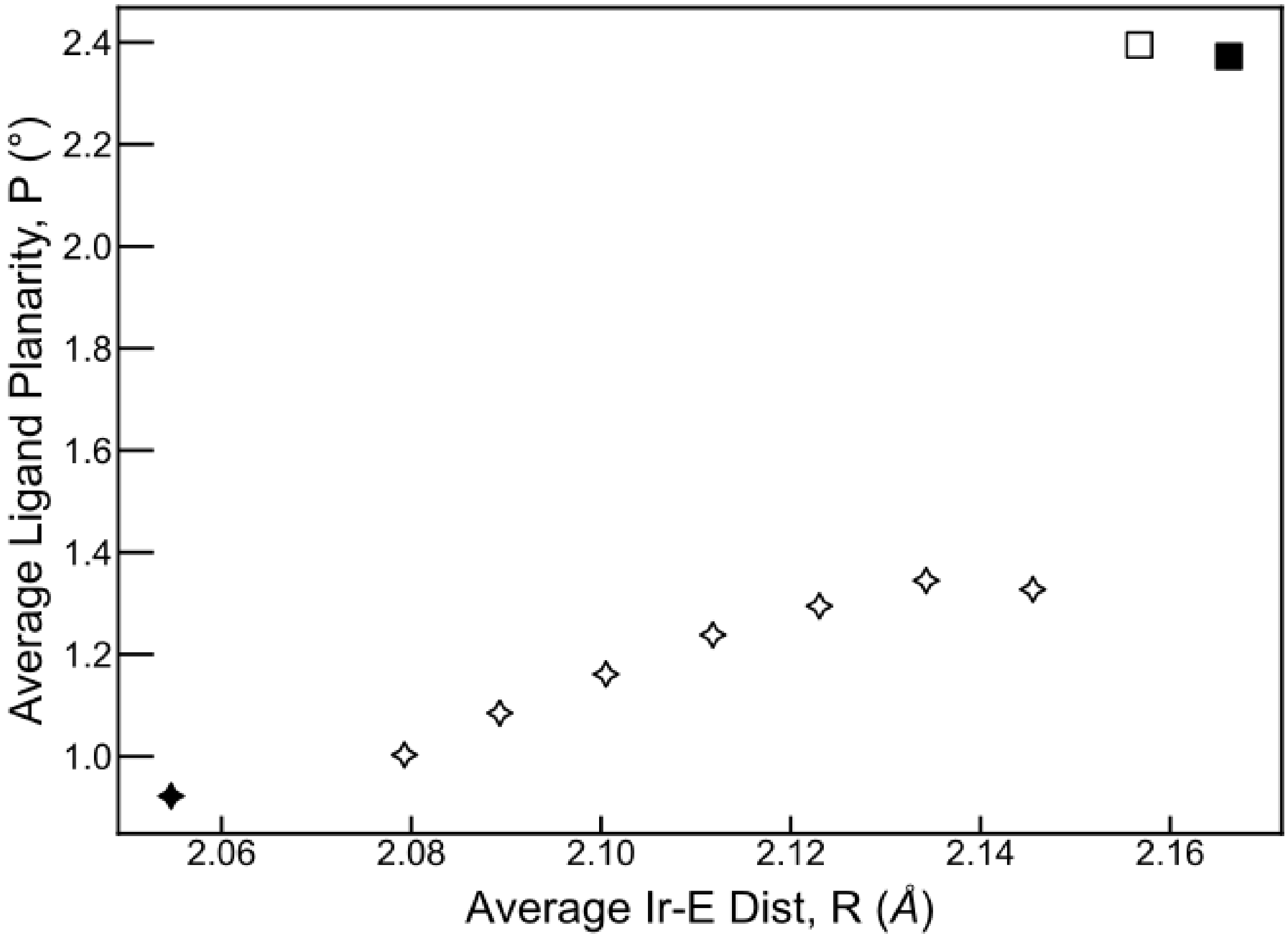

Figure S67: Constrained optimization scans projected onto a flat Structure-structure surface calculated at the PBE0-D3/LANL2DZ[Ir]+6-311G(d,p)[C,H,N]/PCM(MeCN) level of theory. Stars represent triplets with LC character, and squares represent those with MC character.

Potential Energy Curves

Table S31: Structural and energetic parameters along the triplet manifold that correlate with prolonged ^{3}MLCT lifetime, calculated at the PBE0-D3/LANL2DZ[Ir]+6-311G(d,p)[C,H,N]/PCM(MeCN) level of theory.

Complex	$\Delta\mathbb{O}_{T_1}{}^a$ (°)	$\Delta G_{T_1}{}^a$ (eV)	min $\Delta E_{a(T_1)}{}^b$ (eV)	τ (μs)
***fac*-ppy3**	8.14	0.55	((2))^c	1.90^d
***mer*-ppy3**	3.43	0.62	0.77	0.15^d
***m*-ppy2**	6.23	0.79	0.98	0.28^e
***f*-ppy2**	1.73	0.74	1.20	—
**bpy2**	2.28	0.25	0.40	—
**bpy3**	4.74	0.51	0.95	—
***fac*-bzq3***	1.12	1.48	—**	—
***mer*-bzq3**	4.77	0.36	0.73	—
**phen3**	2.52	0.59	0.75	—

^{a}Between optimized triplet minima, ^{3}MC − ^{3}MLCT. ^{b}Higher-energy switch neighbor − ^{3}MLCT. ^{c}Extrapolated fit, see methods. ^{d}Luminescent lifetime in MeCN from Tamayo et al.[13] ^{e}Luminescent lifetime in MeCN from Ladouceur et al.[11] *Lowest-energy SCF structure of ^{3}MC character, see Methods. **No crossing found; SCF-^{3}MC nested within ^{3}MLCT domain.

Minimum-energy crossing points (MECPs) were calculated with the same functional, mixed basis set, and implicit solvent as stationary points optimized in Gaussian16 but with different default dispersion and solvation method in ORCA 6.1.[14,15] Grimmes D3 dispersion corrections included Becke-Johnson damping (D3BJ)[16–18] and the polarizable continuum model was the conductor-like formalism (C-PCM).[19] To directly compare the energies of these optimized structures, single-point calculations were performed in Gaussian16 at the same level of theory as all other calculations reported in this study (Table S32).

Overall, the MECPs range from 0.01−0.18 eV relative to their respective ^{3}MCs (Table S32). Increasing the number of Ir–N contacts by exchanging ppy for bpy increases the energetic difference until homoleptic symmetry is restored at **bpy3**. Altering the number or orientation of through-metal C–Ir–N axes increases this difference on the order of 0.05 eV for the less-preferred isomers. Increasing rigidity with fused aromatic rings decreases this energetic difference by approximately 0.07 eV. As all of these MECPs are structurally more

distorted and energetically higher than the ^{3}MC states, the competition between triplet lifetimes remains governed by properties that distinguish the T_1 sub-domains. Extended PECs along the octahedricity coordinate showcase how far these crossing points are, resulting in large, broad ^{3}MC plateaus (Figure S68).

Table S32: Minimum-energy crossing points calculated at the PBE0-D3/LANL2DZ[Ir]+6-311G(d,p)[C,H,N]/PCM(MeCN) level of theory and reported relative to the ^{3}MC

Complex	^{3}MC (eV)	MECP (eV)	ΔE (eV)
***fac*-ppy3**	3.15	3.16	0.01
***mer*-ppy3**	3.01	3.09	0.08
***m*-ppy2**	3.14	3.16	0.02
***f*-ppy2**	3.04	3.15	0.12
**bpy2**	2.91	3.09	0.18
**bpy3**	3.34	3.34	0.00
***fac*-bzq3**	3.99*	3.29	-0.70**
***mer*-bzq3**	2.80	2.81	0.01
**phen3**	3.39	3.33	-0.06

*Lowest-energy SCF structure of ^{3}MC character, see Methods. **Measured between optimized MECP and un-optimzied ^{3}MC.

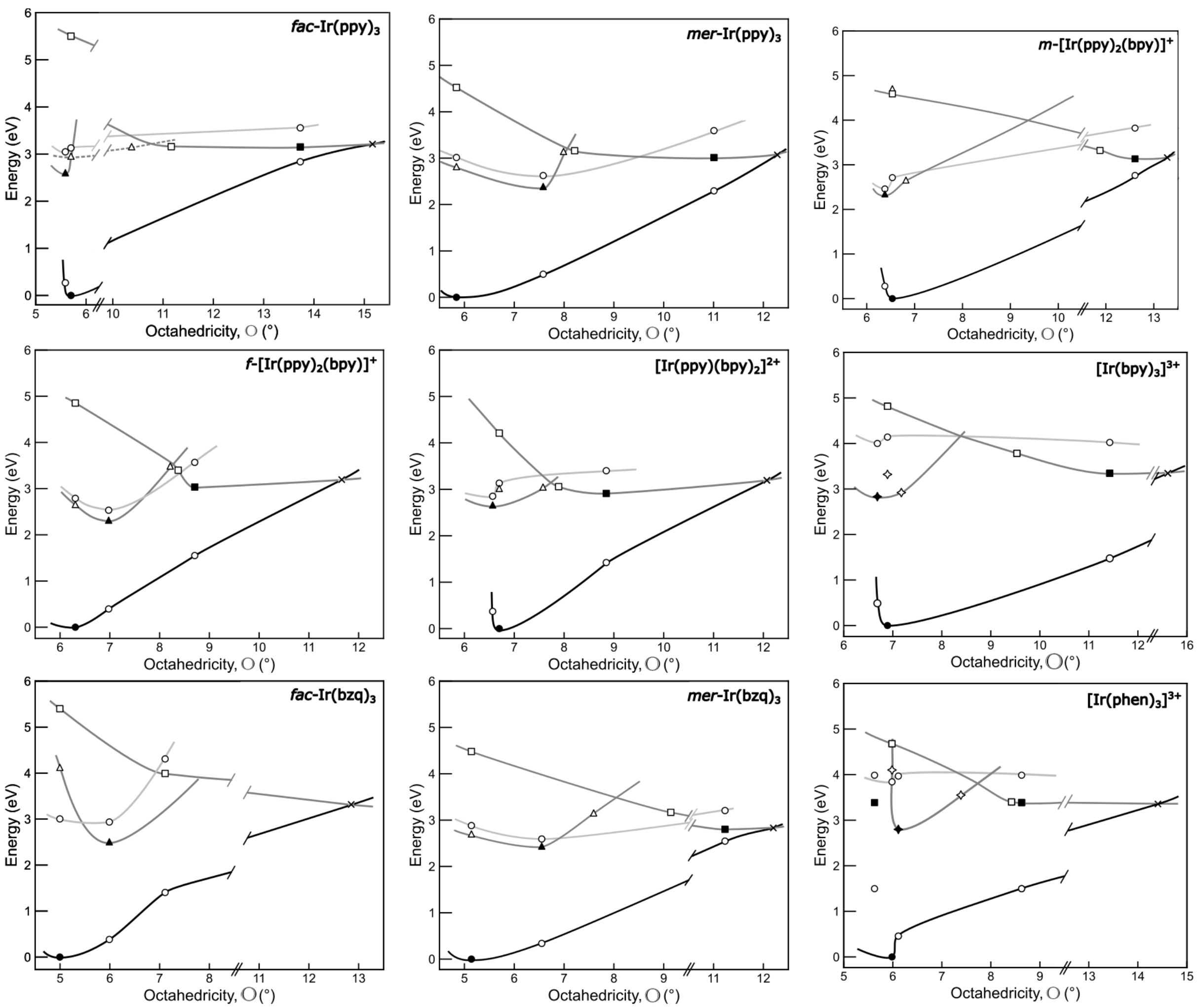

Figure S68: Expanded potential energy curves of all complexes calculated at the PBE0-D3/LANL2DZ[Ir]+6-311G(d,p)[C,H,N]/PCM(MeCN) level of theory. Circles are singlet species, triangles are triplets with charge-transfer character, stars are triplets with ligand-centered character, and squares are triplets with metal-centered character. Filled markers are freely-optimized minima and complementary open-marker points are the SCF energy or TD-DFT excitations at that geometry. Minimum-energy crossing points (MECPs) are shown with an X.

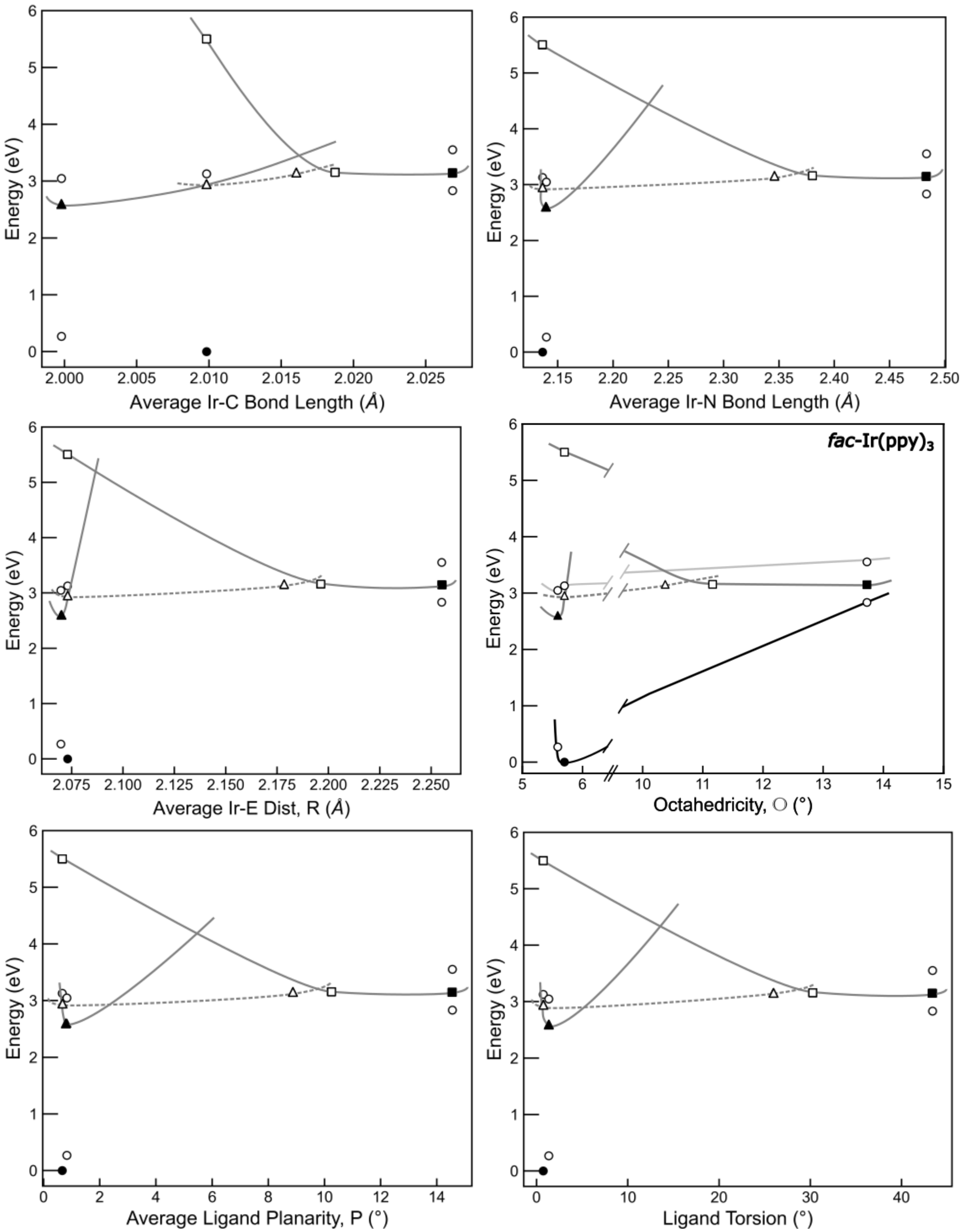

Figure S69: Potential energy curves of ***fac*-ppy3** calculated at the PBE0-D3/LANL2DZ[Ir]+6-311G(d,p)[C,H,N]/PCM(MeCN) level of theory. Circles are singlet species, triangles are triplets with CT character while squares are triplets with MC character. Filled markers are freely-optimized minima and complementary open-marker points are the SCF energy or TD-DFT excitations at that geometry.

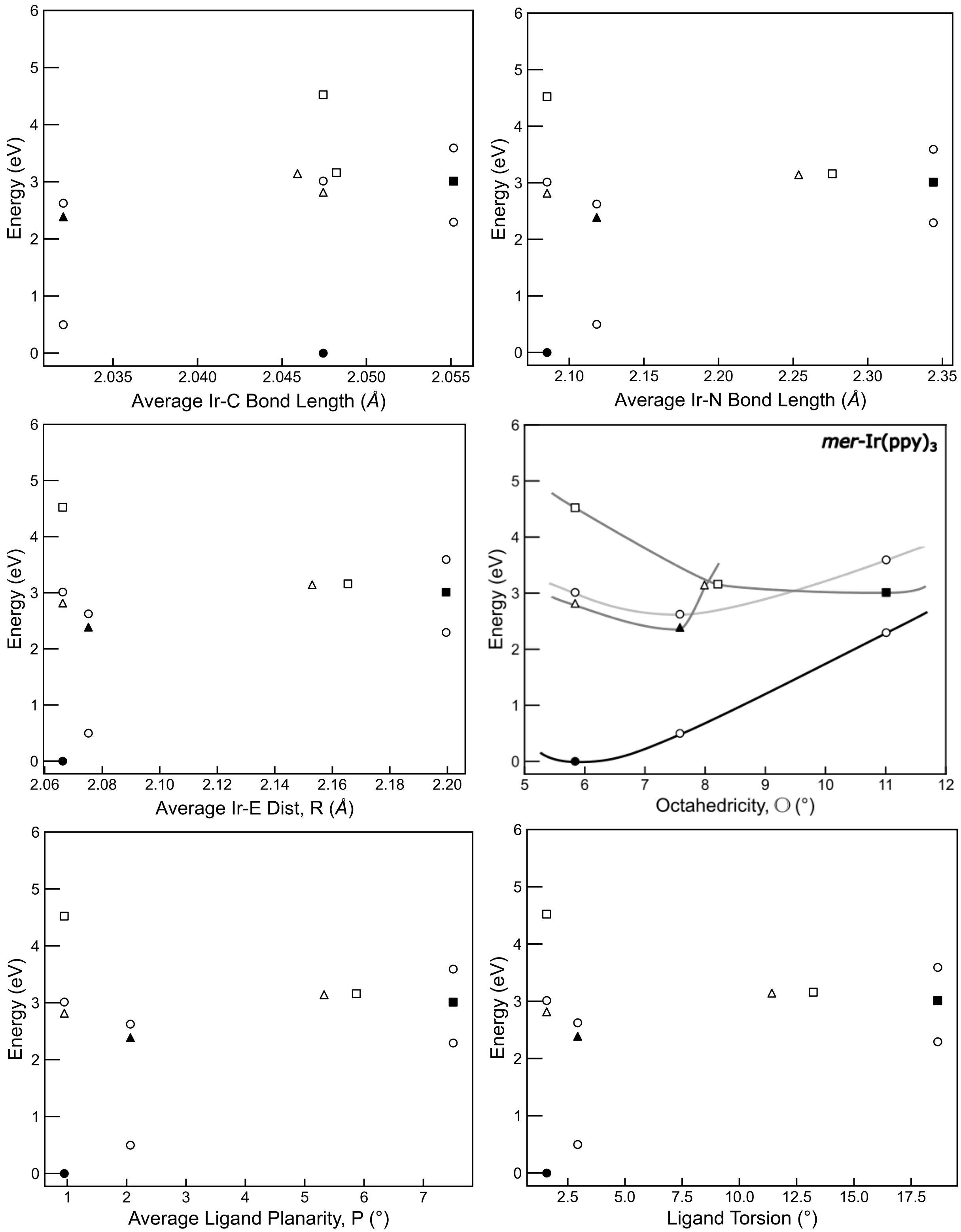

Figure S70: Potential energy curves of ***mer*-ppy3** calculated at the PBE0-D3/LANL2DZ[Ir]+6-311G(d,p)[C,H,N]/PCM(MeCN) level of theory. Circles are singlet species, triangles are triplets with CT character while squares are triplets with MC character. Filled markers are freely-optimized minima and complementary open-marker points are the SCF energy or TD-DFT excitations at that geometry.

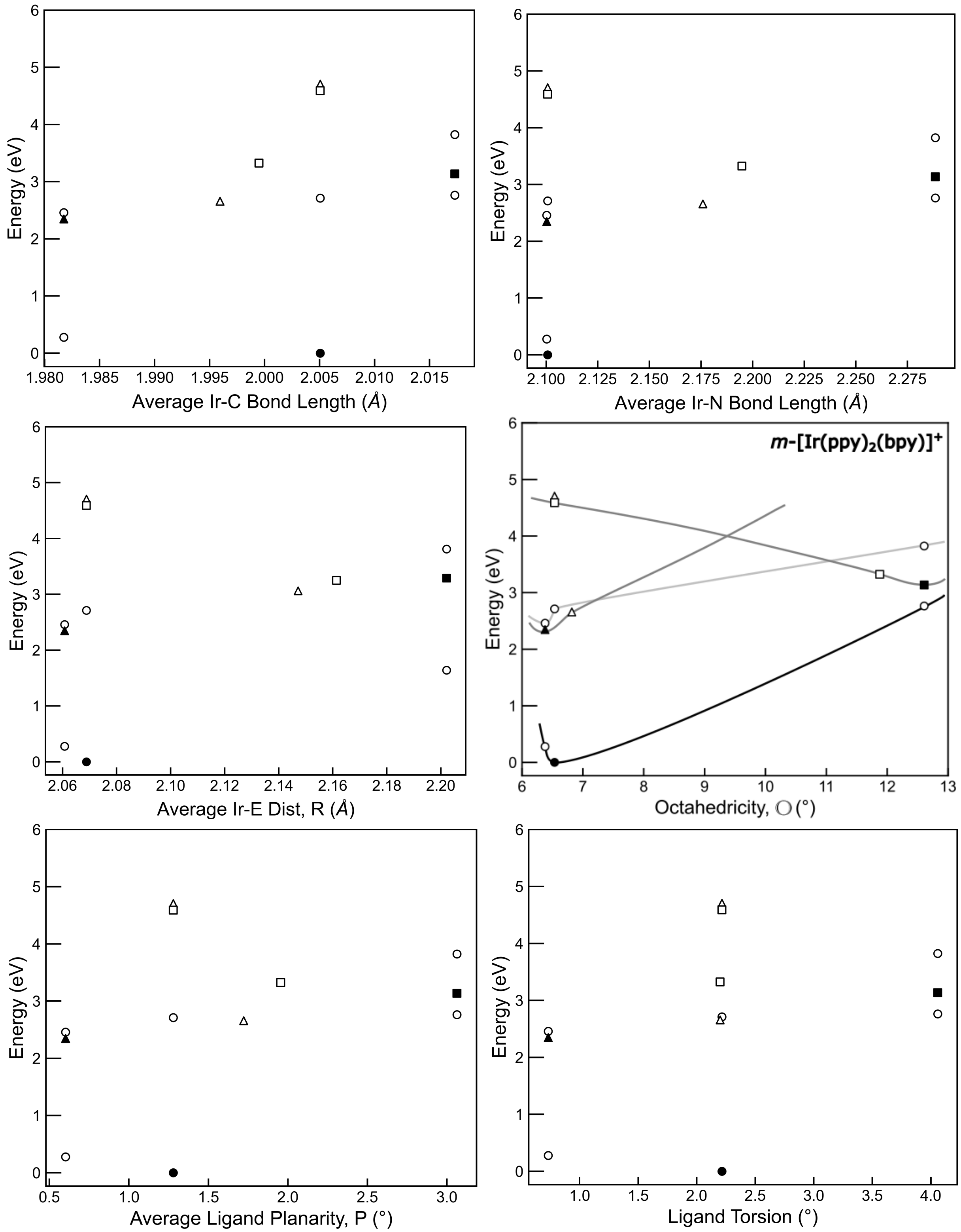

Figure S71: Potential energy curves of ***m*-ppy2** calculated at the PBE0-D3/LANL2DZ[Ir]+6-311G(d,p)[C,H,N]/PCM(MeCN) level of theory. Circles are singlet species, triangles are triplets with CT character while squares are triplets with MC character. Filled markers are freely-optimized minima and complementary open-marker points are the SCF energy or TD-DFT excitations at that geometry.

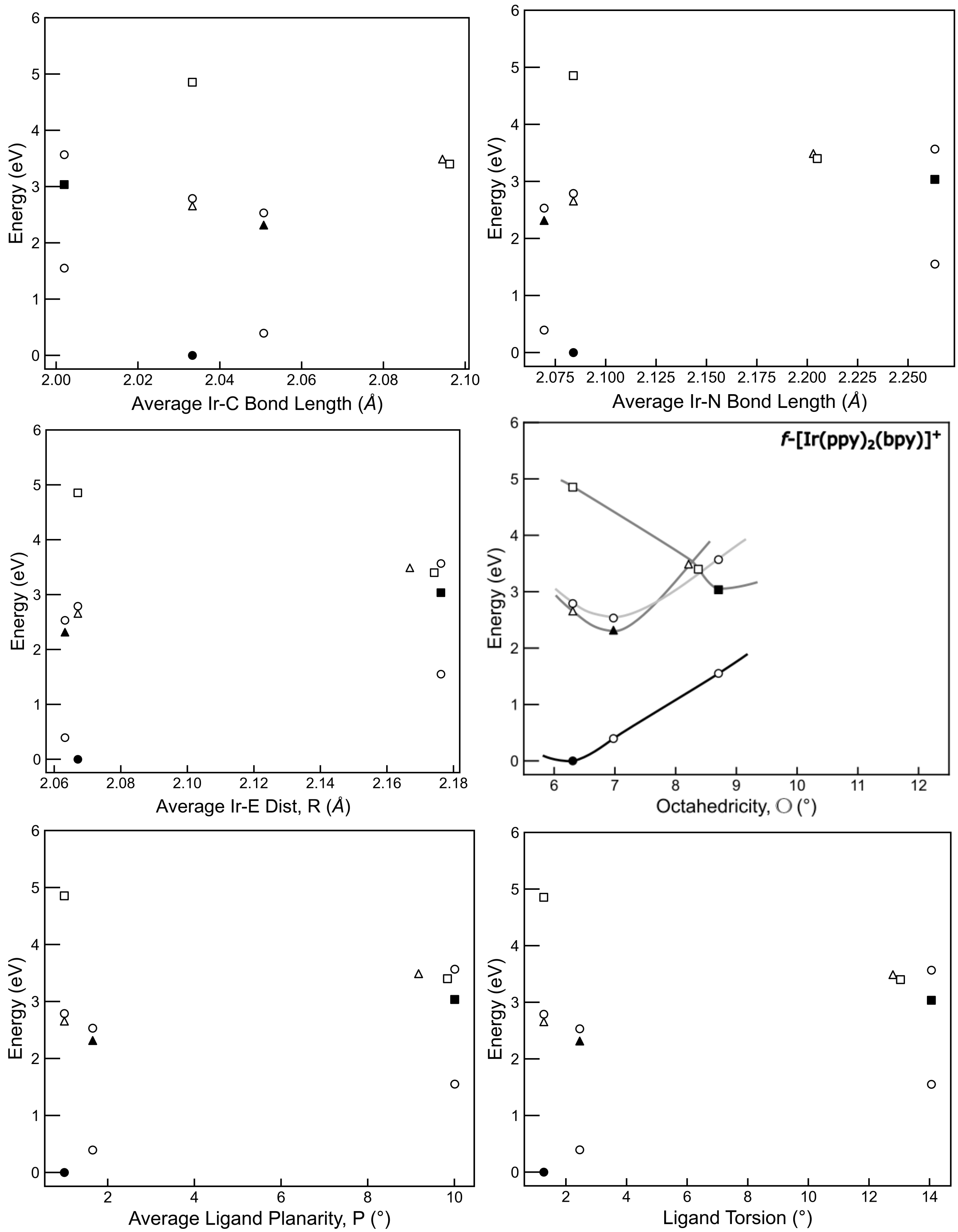

Figure S72: Potential energy curves of ***f*-ppy2** calculated at the PBE0-D3/LANL2DZ[Ir]+6-311G(d,p)[C,H,N]/PCM(MeCN) level of theory. Circles are singlet species, triangles are triplets with CT character while squares are triplets with MC character. Filled markers are freely-optimized minima and complementary open-marker points are the SCF energy or TD-DFT excitations at that geometry.

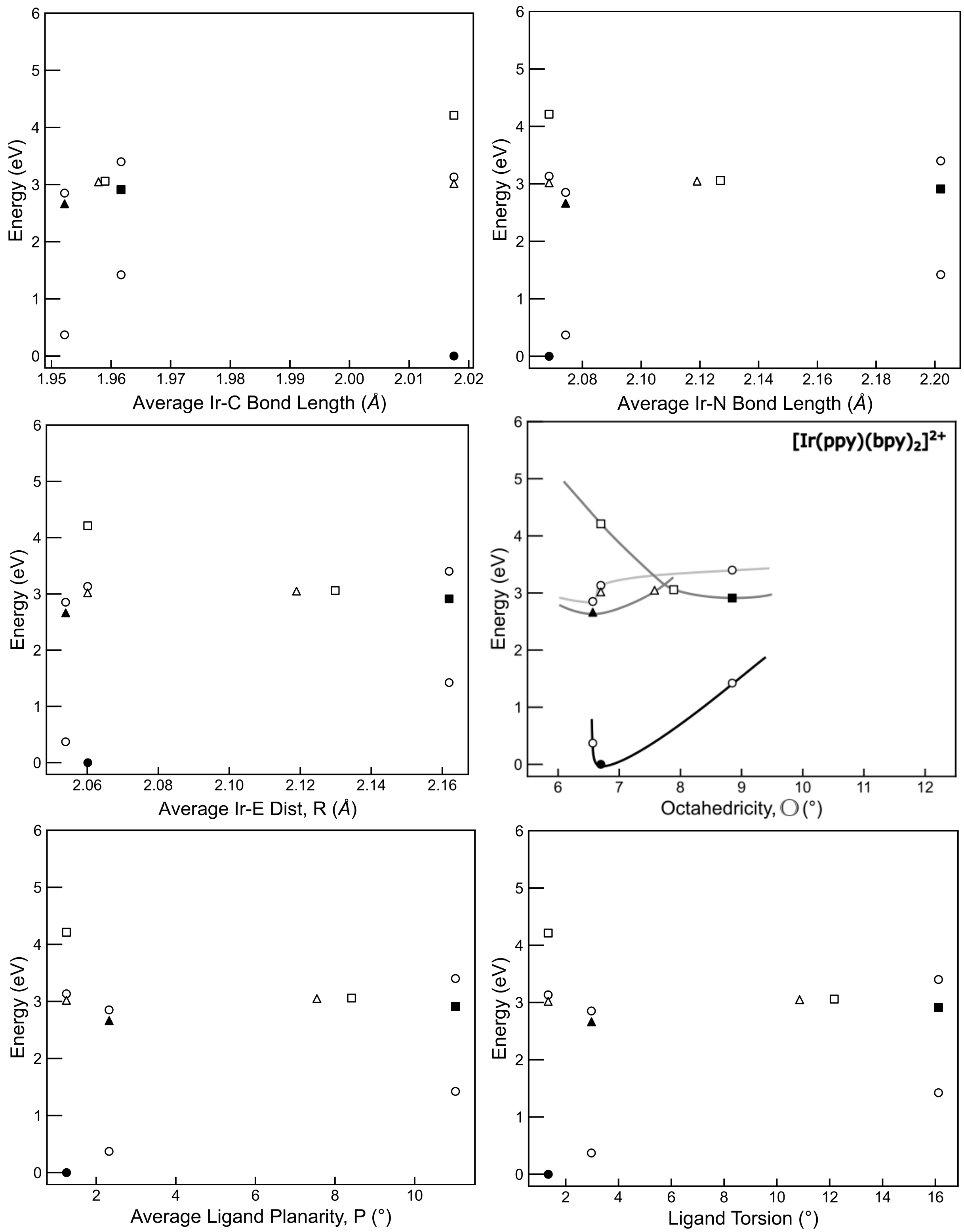

Figure S73: Potential energy curves of **bpy2** calculated at the PBE0-D3/LANL2DZ[Ir]+6-311G(d,p)[C,H,N]/PCM(MeCN) level of theory. Circles are singlet species, triangles are triplets with CT character while squares are triplets with MC character. Filled markers are freely-optimized minima and complementary open-marker points are the SCF energy or TD-DFT excitations at that geometry.

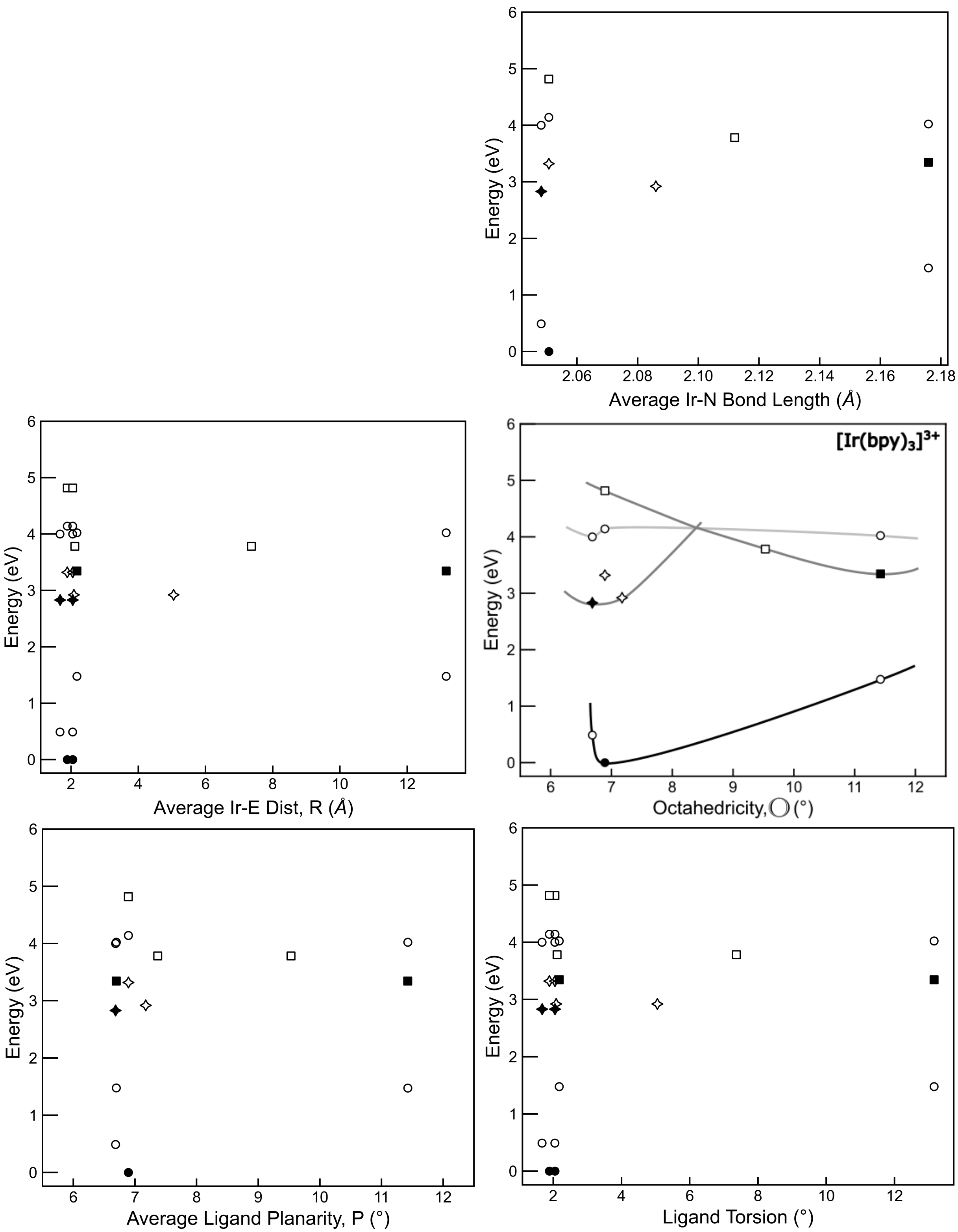

Figure S74: Potential energy curves for **bpy3** calculated at the PBE0-D3/LANL2DZ[Ir]+6-311G(d,p)[C,H,N]/PCM(MeCN) level of theory. Circles are singlet species, stars are triplets with LC character, while squares are triplets with MC character. Filled markers are freely-optimized minima and complementary open-marker points are the SCF energy or TD-DFT excitations at that geometry.

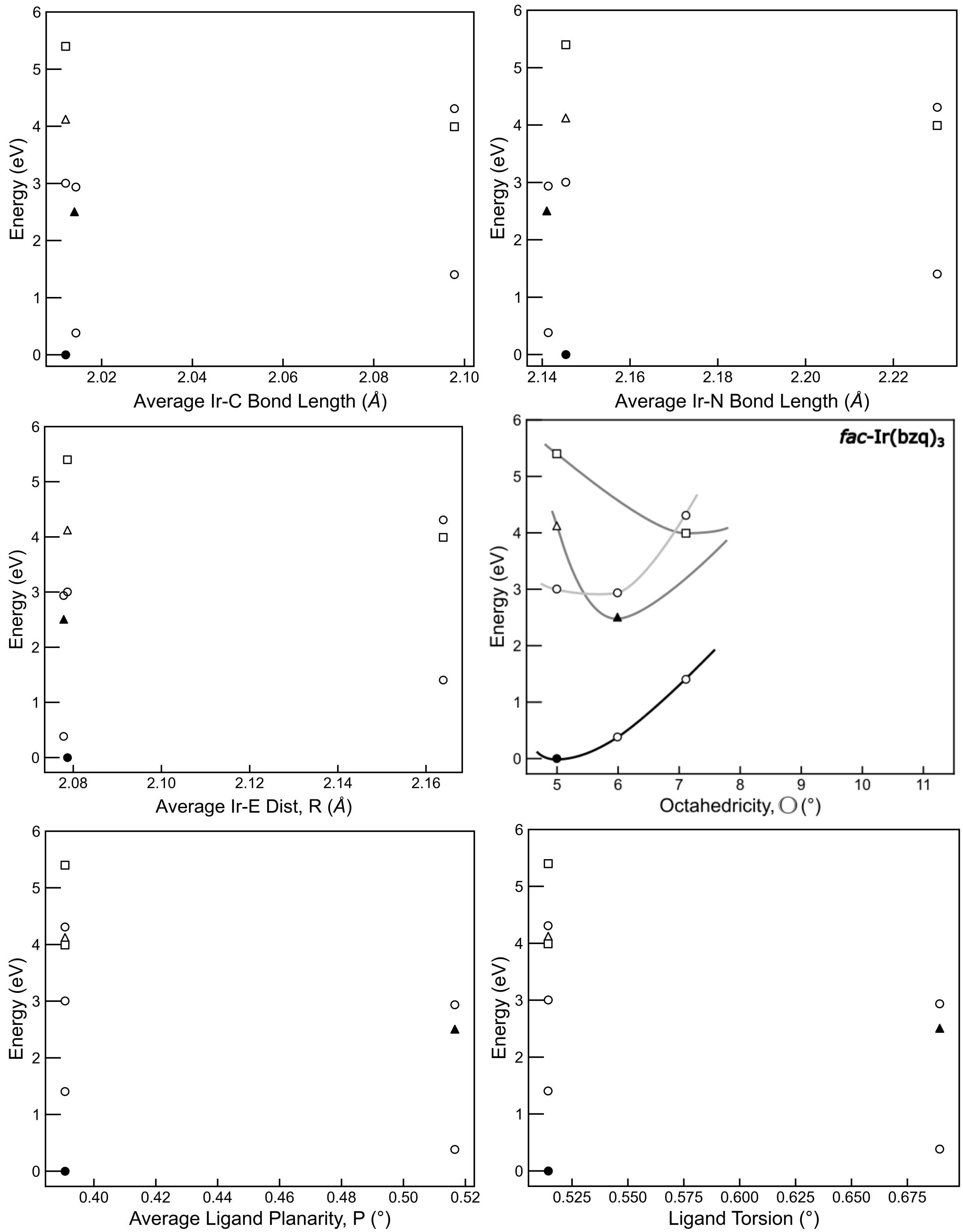

Figure S75: Potential energy curves of ***fac*-bzq3** calculated at the PBE0-D3/LANL2DZ[Ir]+6-311G(d,p)[C,H,N]/PCM(MeCN) level of theory. Circles are singlet species, triangles are triplets with CT character while squares are triplets with MC character. Filled markers are freely-optimized minima and complementary open-marker points are the SCF energy or TD-DFT excitations at that geometry.

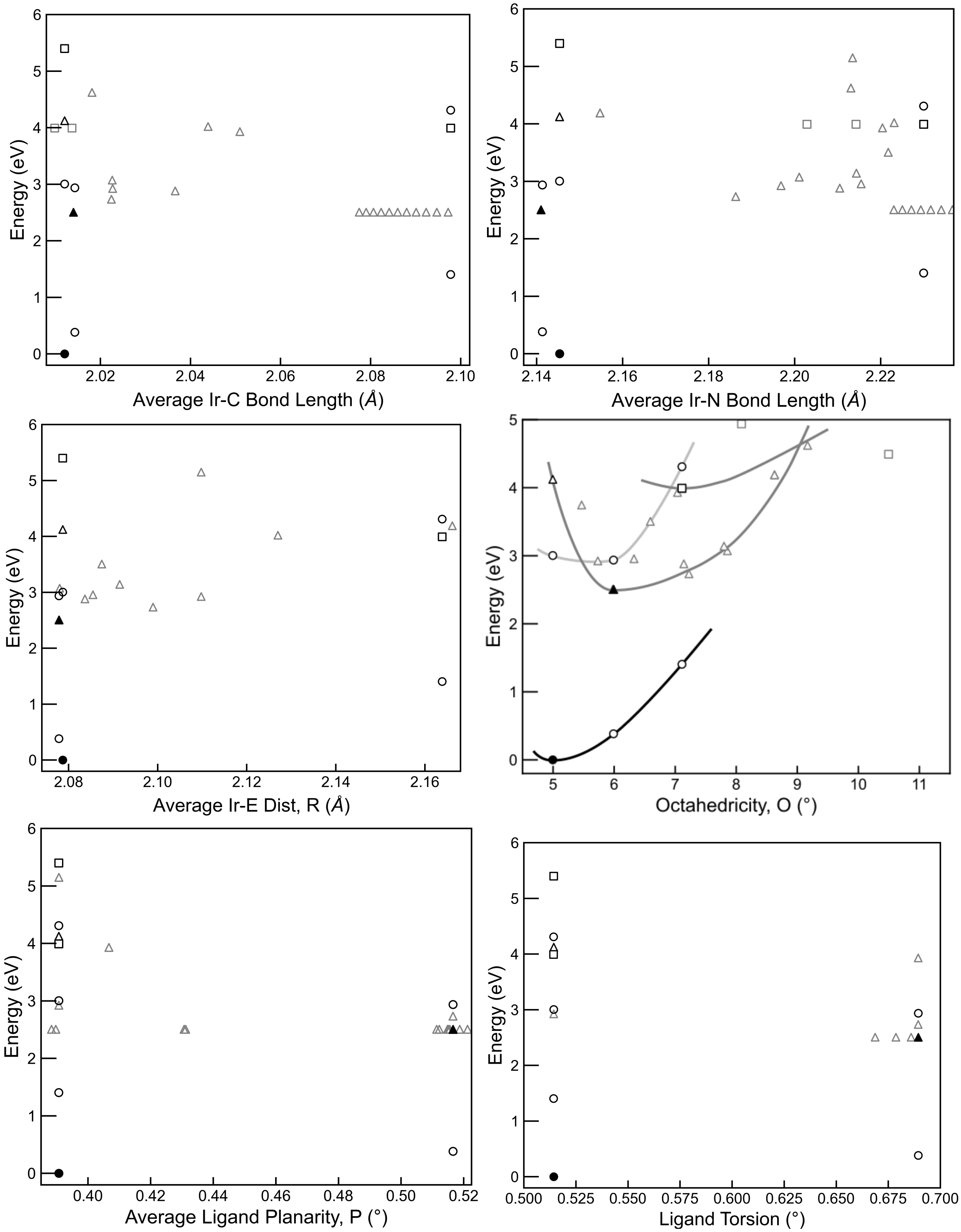

Figure S76: Potential energy curves of ***fac*-bzq3** with other ^{3}MC-search points (grey) calculated at the PBE0-D3/LANL2DZ[Ir]+6-311G(d,p)[C,H,N]/PCM(MeCN) level of theory. Circles are singlet species, triangles are triplets with CT character while squares are triplets with MC character. Filled markers are freely-optimized minima and complementary open-marker points are the SCF energy or TD-DFT excitations at that geometry. Shown on the same scale/range as main text.

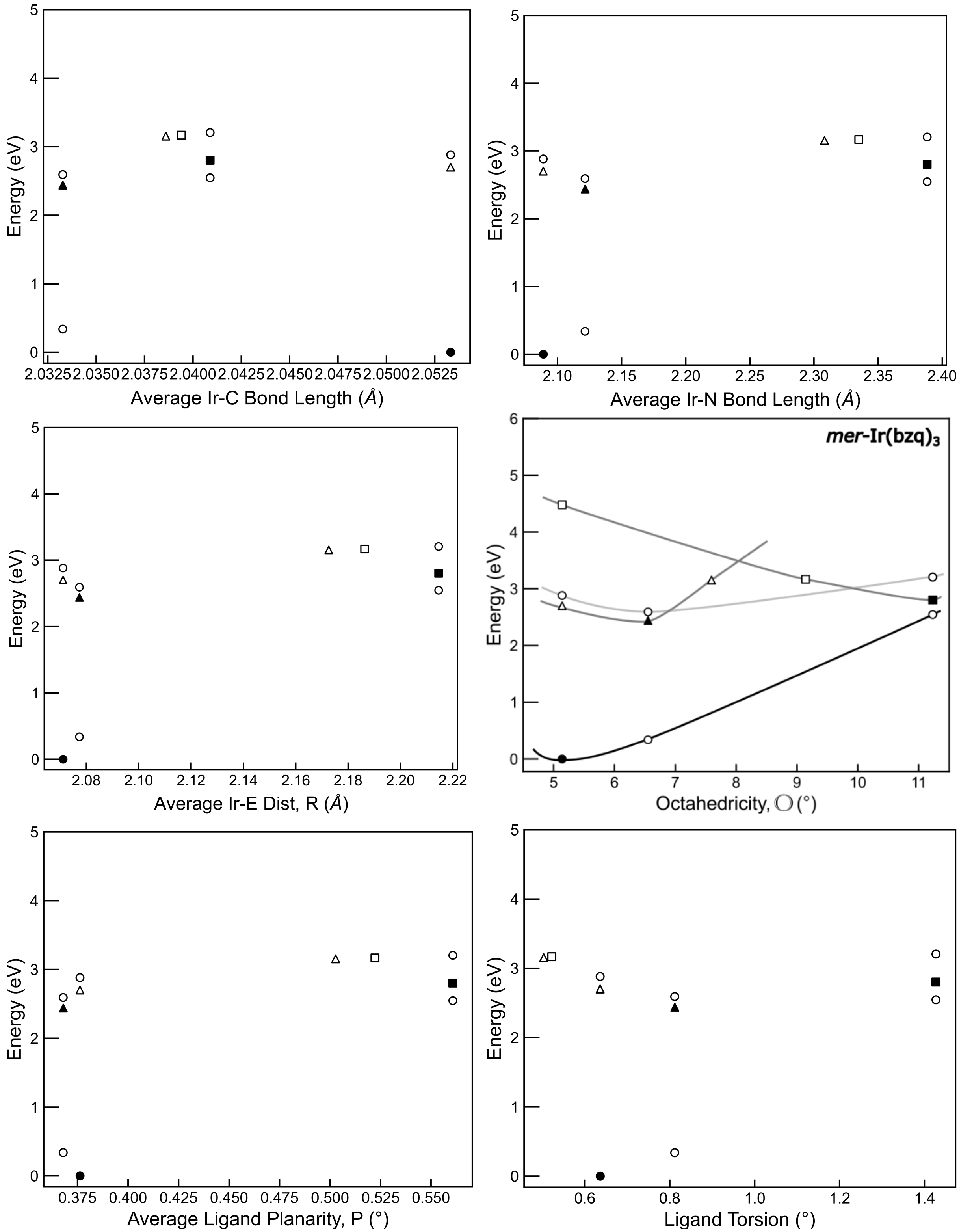

Figure S77: Potential energy curves of ***mer*-bzq3** calculated at the PBE0-D3/LANL2DZ[Ir]+6-311G(d,p)[C,H,N]/PCM(MeCN) level of theory. Circles are singlet species, triangles are triplets with CT character while squares are triplets with MC character. Filled markers are freely-optimized minima and complementary open-marker points are the SCF energy or TD-DFT excitations at that geometry.

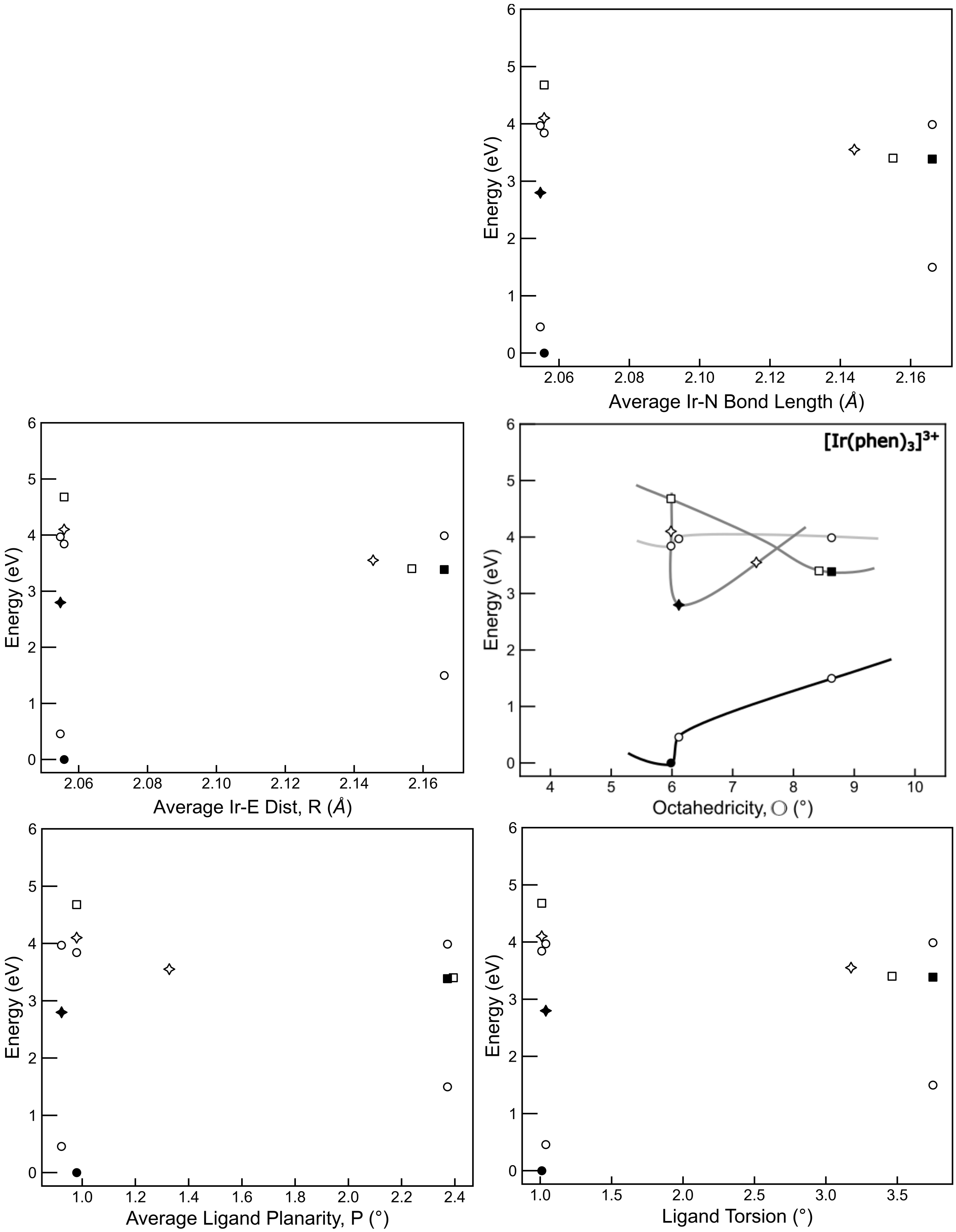

Figure S78: Potential energy curves of **phen3** calculated at the PBE0-D3/LANL2DZ[Ir]+6-311G(d,p)[C,H,N]/PCM(MeCN) level of theory. Circles are singlet species, stars are triplets with LC character, while squares are triplets with MC character. Filled markers are freely-optimized minima and complementary open-marker points are the SCF energy or TD-DFT excitations at that geometry.

Structure-Structure Projected Surfaces

Twin-axis potential energy surfaces (PES) against Ir-C bond length and Octahedricity are shown below. For complexes with no Ir-C bonds (e.g. **bpy3** and **phen3**) only the PES along $\mathbb{O}$ is shown. These twin axis plots highlight that ligand torsion p_l is driven by the nitrogen-bearing half of the ligand as it twists away from the metal, and that individual ligand torsion is the main contributor to overall average planarity, $\mathbb{P}$. For many complexes, the differences in black (twisting) and red (Ir-N or $\mathbb{P}$) connectivity between the three stationary points is imperceptible. Complexes whose plots have extreme divergence of red/black paths correspond to compounds whose ^{3}MC states may not twist much, or who push outward more than they twist.

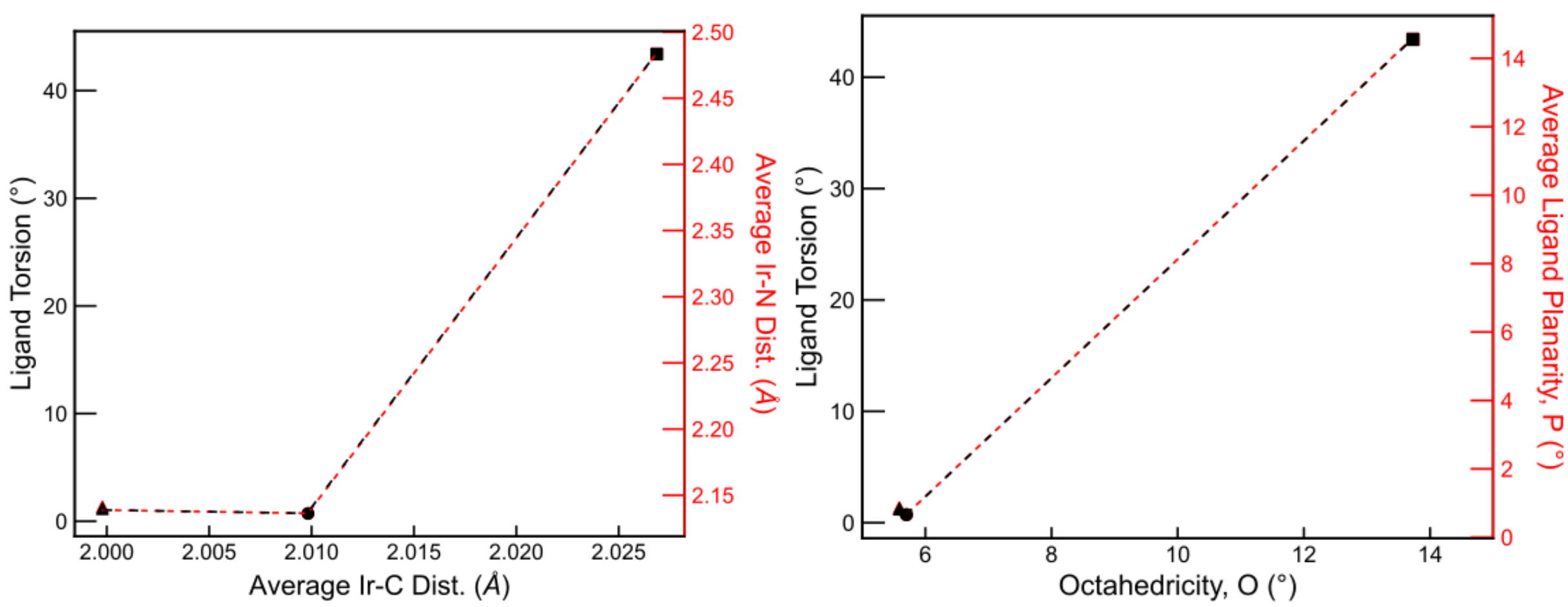

Figure S79: Twin-axis potential energy surfaces of ***fac*-ppy3** calculated at the PBE0-D3/LANL2DZ[Ir]+6-311G(d,p)[C,H,N]/PCM(MeCN) level of theory. Circles are singlet (GS) species, triangles are ^{3}MLCT, and squares are ^{3}MC.

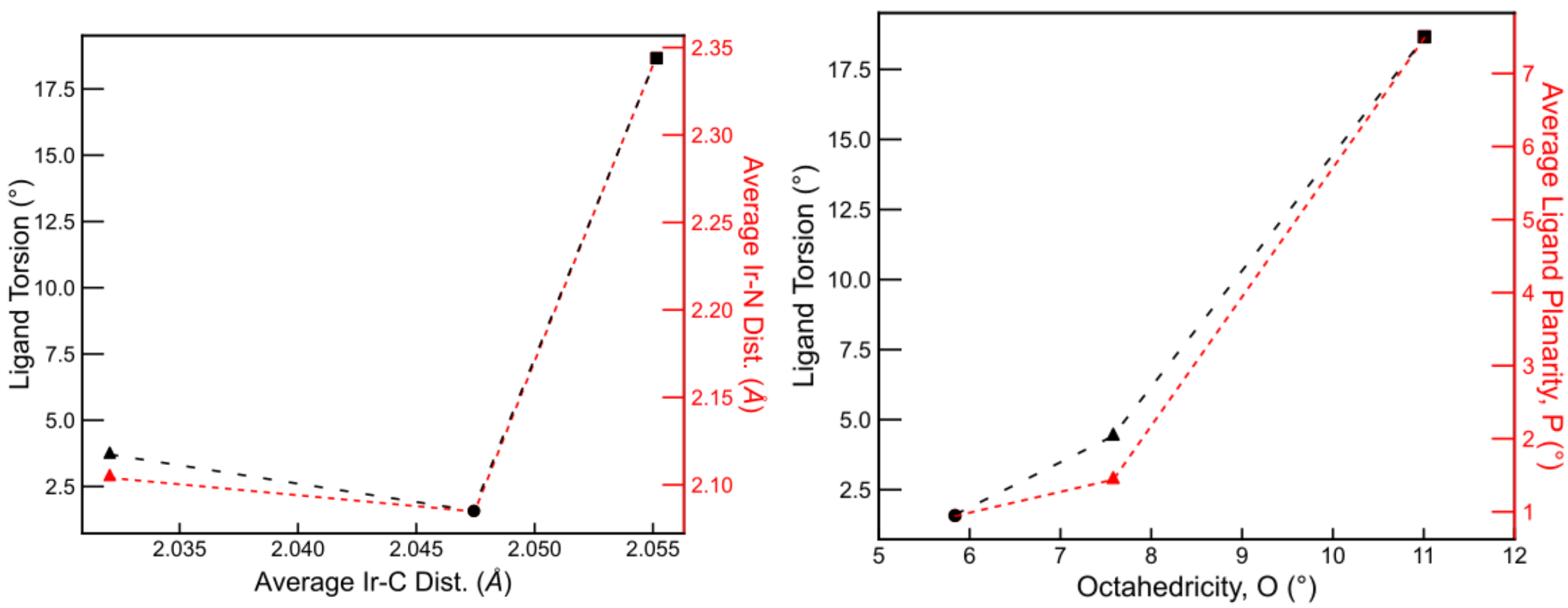

Figure S80: Twin-axis potential energy surfaces of ***mer*-ppy3** calculated at the PBE0-D3/LANL2DZ[Ir]+6-311G(d,p)[C,H,N]/PCM(MeCN) level of theory. Circles are singlet (GS) species, triangles are ^{3}MLCT, and squares are ^{3}MC.

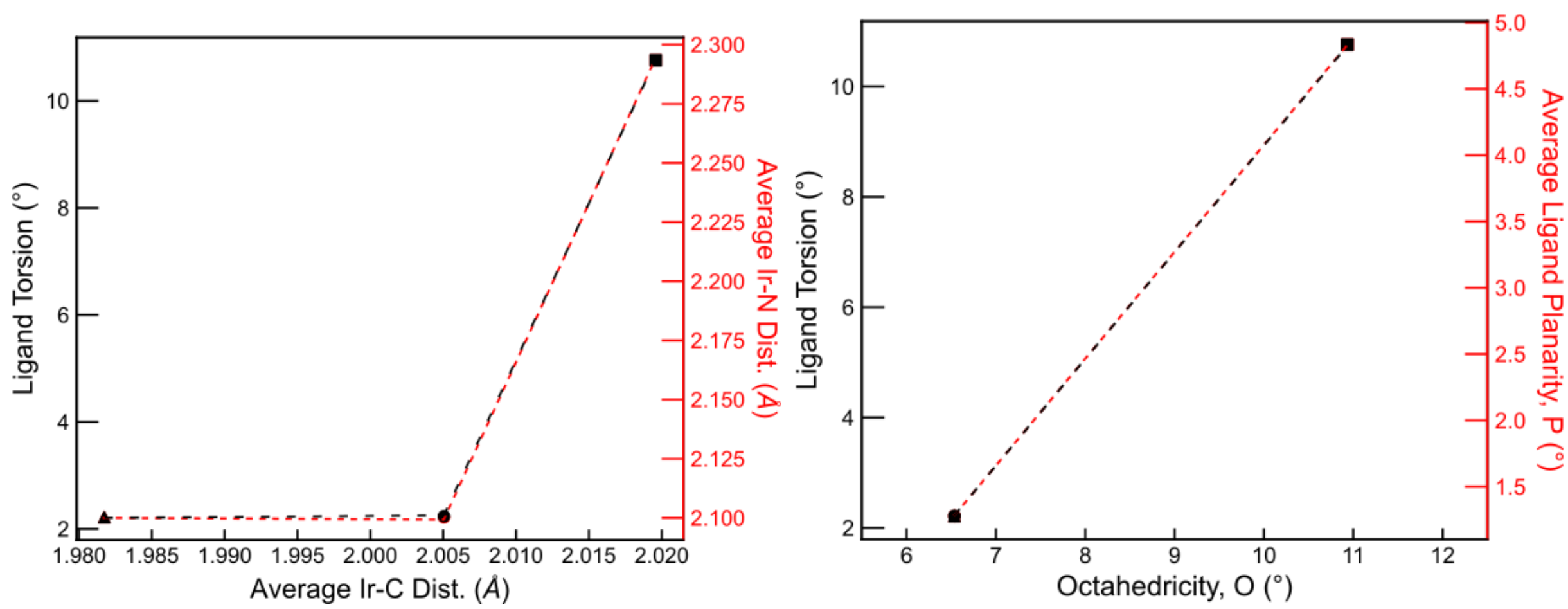

Figure S81: Twin-axis potential energy surfaces of ***m*-ppy2** calculated at the PBE0-D3/LANL2DZ[Ir]+6-311G(d,p)[C,H,N]/PCM(MeCN) level of theory. Circles are singlet (GS) species, triangles are ^{3}MLCT, and squares are ^{3}MC.

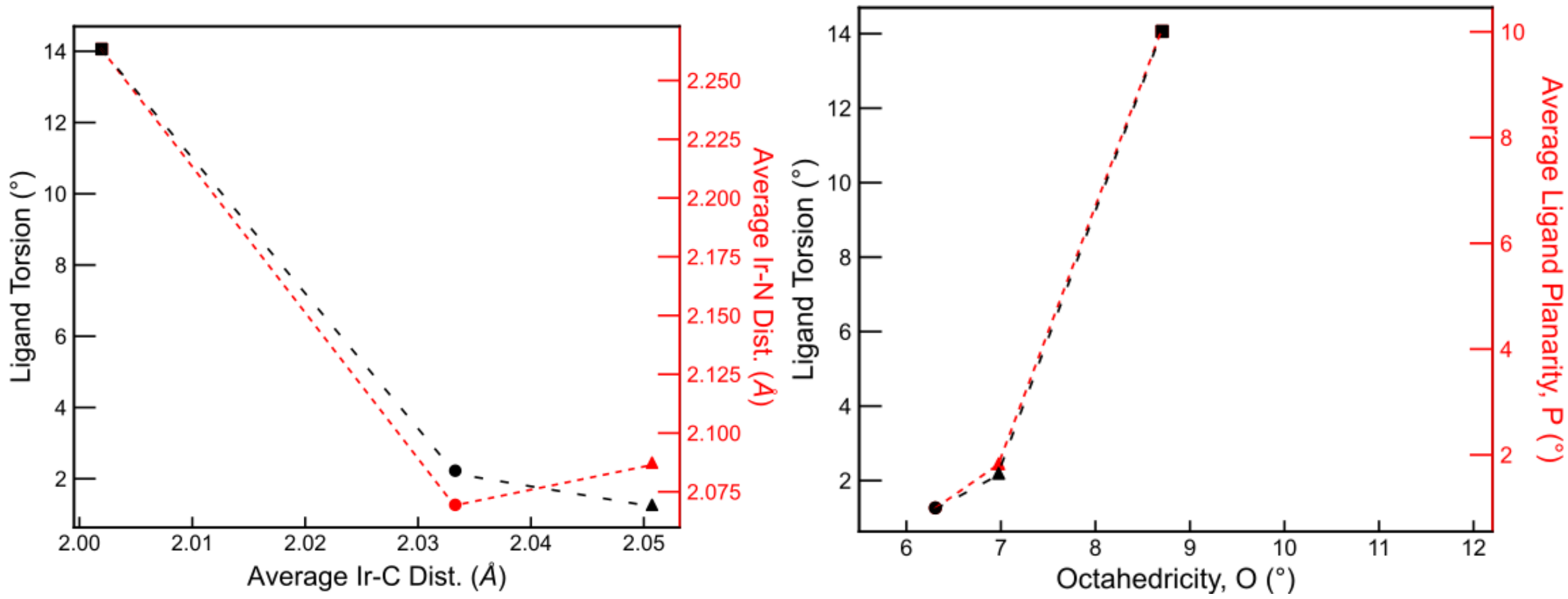

Figure S82: Twin-axis potential energy surfaces of ***f*-ppy2** calculated at the PBE0-D3/LANL2DZ[Ir]+6-311G(d,p)[C,H,N]/PCM(MeCN) level of theory. Circles are singlet (GS) species, triangles are ^{3}MLCT, and squares are ^{3}MC.

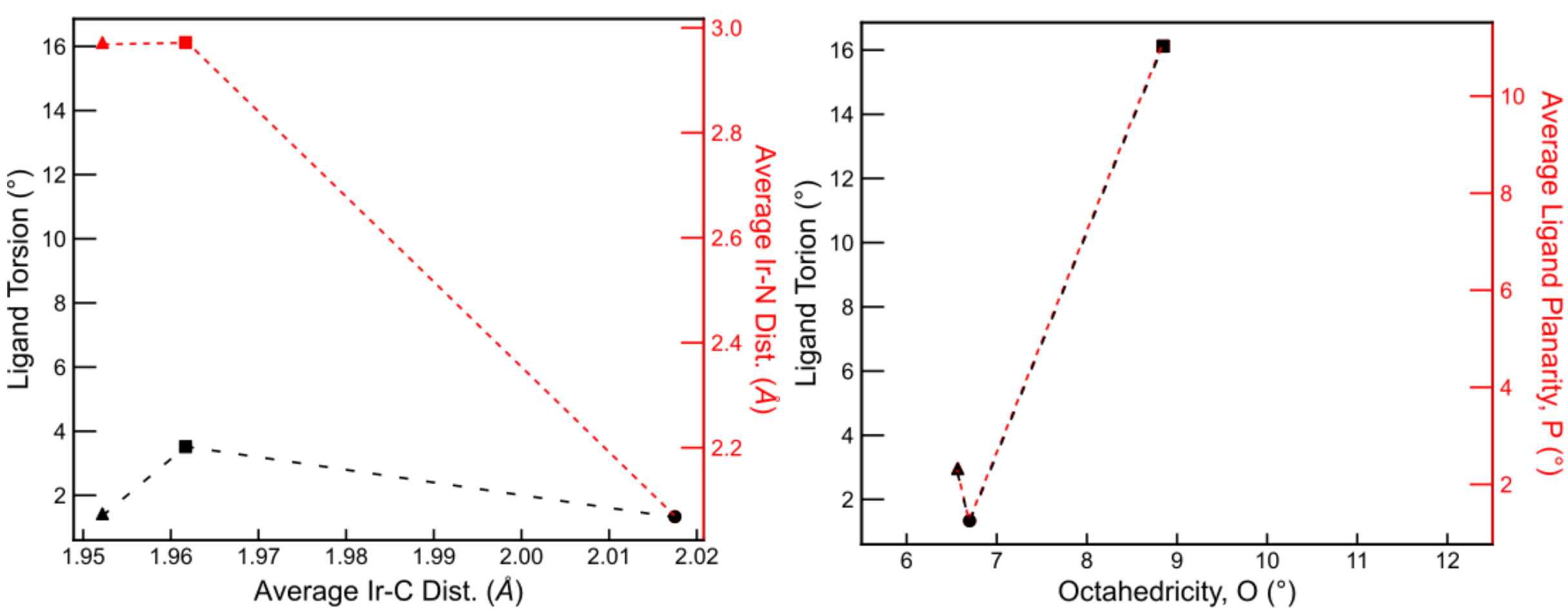

Figure S83: Twin-axis potential energy surfaces of **bpy2** calculated at the PBE0-D3/LANL2DZ[Ir]+6-311G(d,p)[C,H,N]/PCM(MeCN) level of theory. Circles are singlet (GS) species, triangles are ^{3}MLCT, and squares are ^{3}MC.

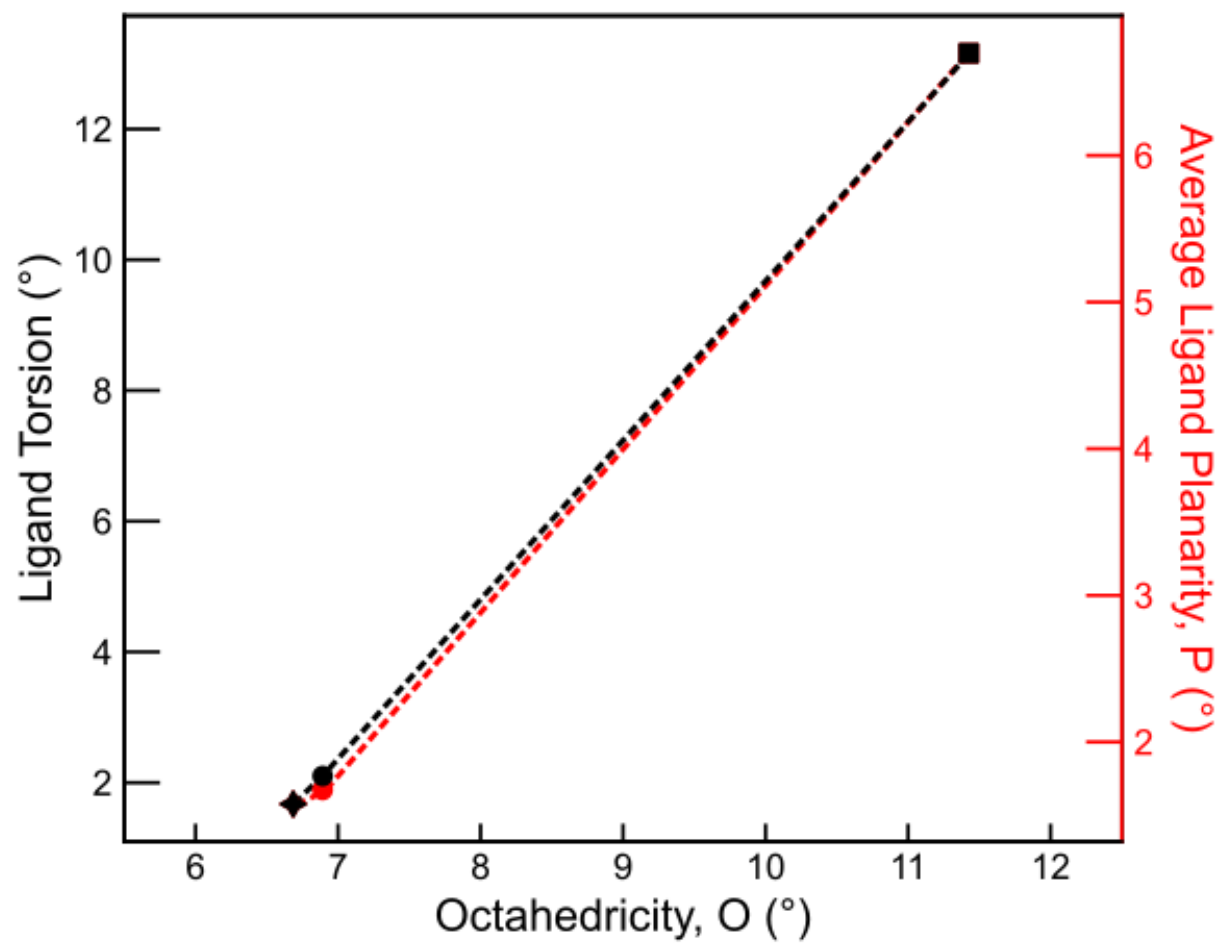

Figure S84: Twin-axis potential energy surfaces of **bpy3** calculated at the PBE0-D3/LANL2DZ[Ir]+6-311G(d,p)[C,H,N]/PCM(MeCN) level of theory. Circles are singlet (GS) species, stars are ^{3}LC, and squares are ^{3}MC.

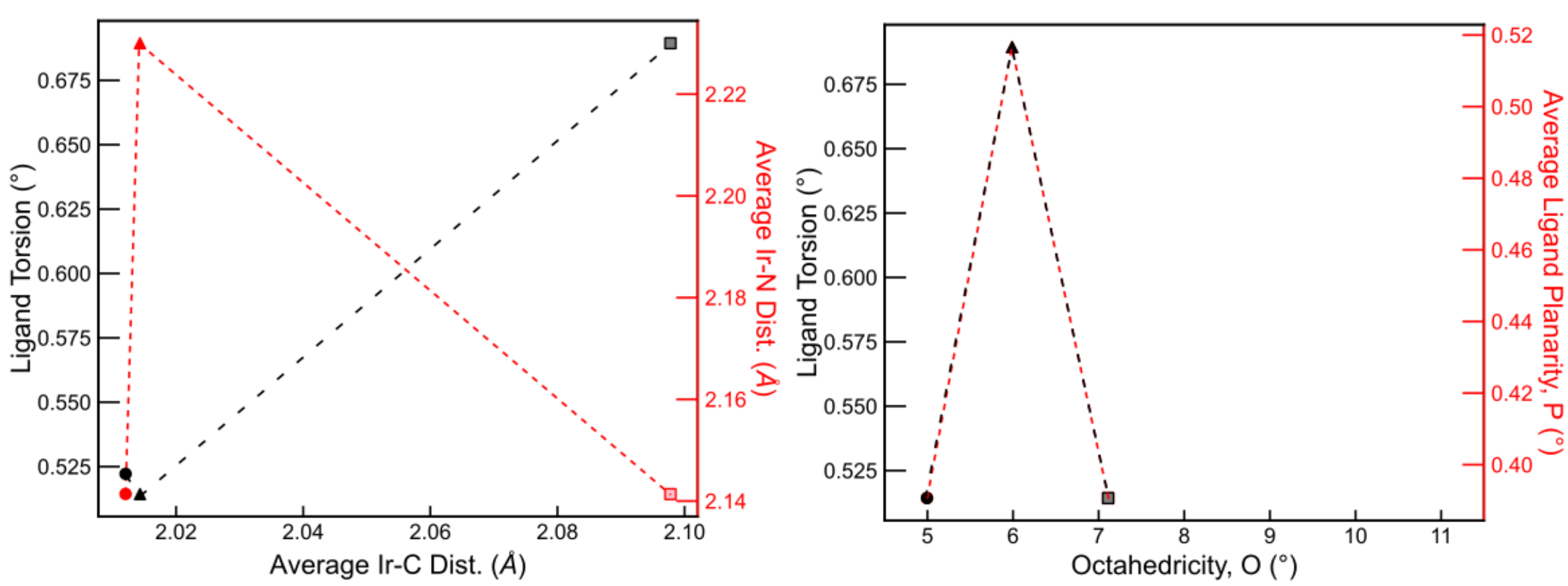

Figure S85: Twin-axis potential energy surfaces of ***fac*-bzq3** calculated at the PBE0-D3/LANL2DZ[Ir]+6-311G(d,p)[C,H,N]/PCM(MeCN) level of theory. Circles are singlet (GS) species, triangles are ^{3}MLCT, and squares are ^{3}MC.

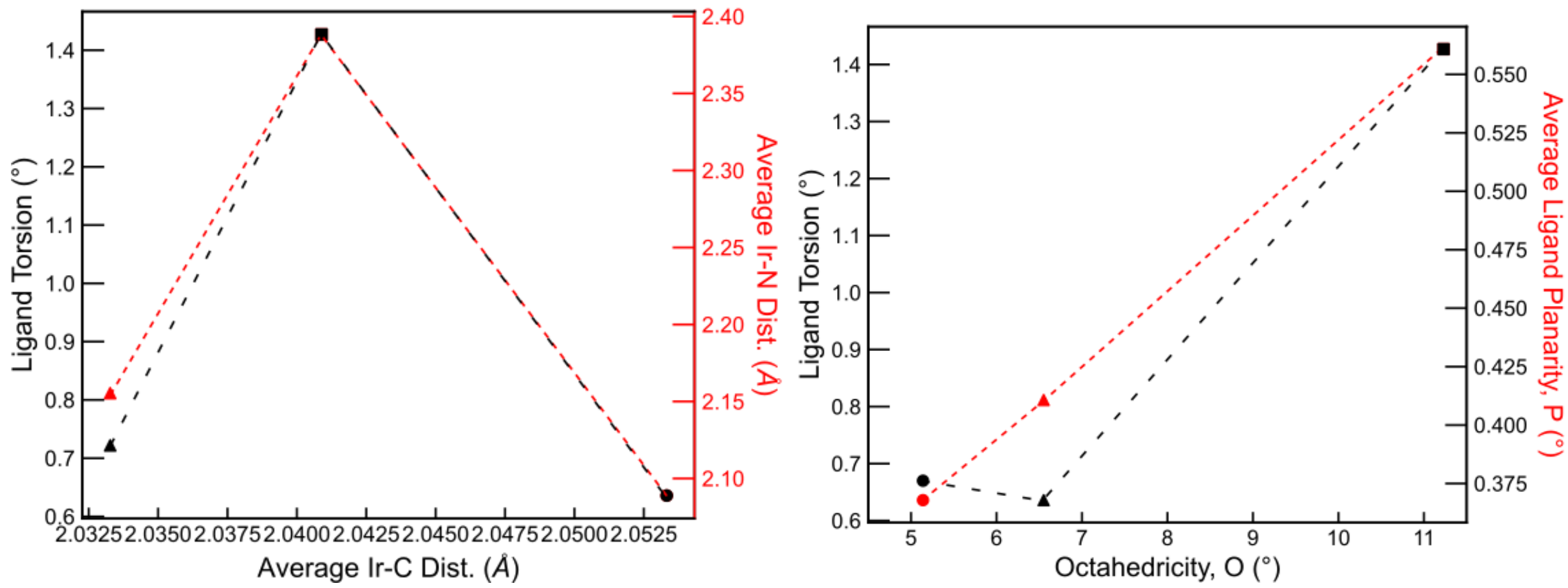

Figure S86: Twin-axis potential energy surfaces of ***mer*-bzq3** calculated at the PBE0-D3/LANL2DZ[Ir]+6-311G(d,p)[C,H,N]/PCM(MeCN) level of theory. Circles are singlet (GS) species, triangles are ^{3}MLCT, and squares are ^{3}MC.

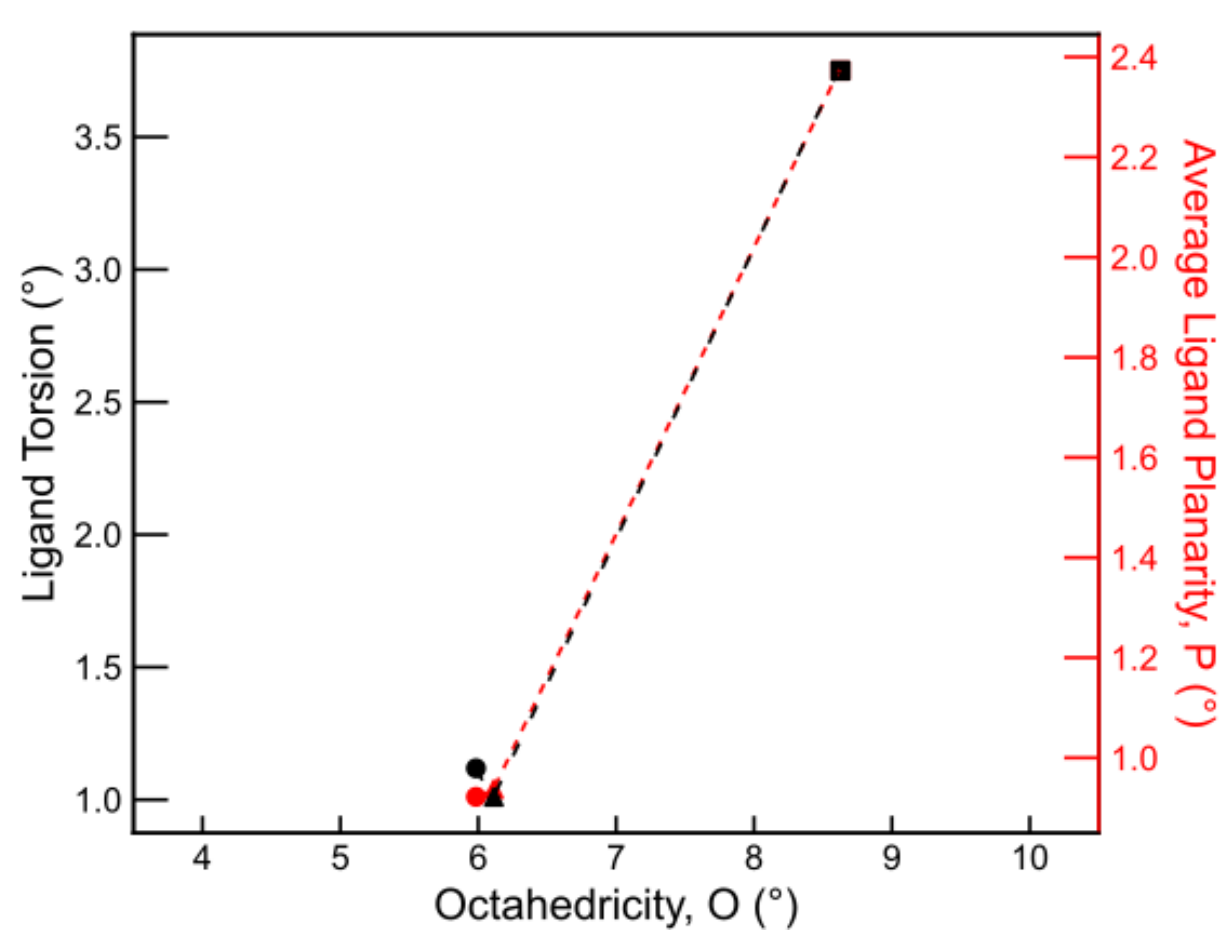

Figure S87: Twin-axis potential energy surfaces of **phen3** calculated at the PBE0-D3/LANL2DZ[Ir]+6-311G(d,p)[C,H,N]/PCM(MeCN) level of theory. Circles are singlet (GS) species, stars are ^{3}LC, and squares are ^{3}MC.

References

(1) Breu, J.; Stössel, P.; Schrader, S.; Starukhin, A.; Finkenzeller, W. J.; Yersin, H. Crystal Structure of facIr(ppy)3 and Emission Properties under Ambient Conditions and at High Pressure. *Chem. Mater.* **2005**, *17*, 1745–1752.

(2) Teng, M.-Y.; Wang, C.-C.; Jing, Y.-M.; Zheng, Y.-X.; Li, C. Efficient Green Organic Light-Emitting Diodes with fac-Tris(2-(4-trifluoromethylphenyl)pyridine)iridium Complex as Emitter. *Chin. J. Inorg. Chem.* **2013**, *29*, 1490–1496.

(3) Yao, H.; Zhang, L.; Peng, Y.; Carroll, P. J.; Gong, L.; Meggers, E. Novel metal-coordinated 1,10-phenanthroline ligands functionalized with a lactam or imide. *Inorg. Chim. Acta* **2014**, *421*, 489–495.

(4) Ma, D.; Qiu, Y.; Duan, L. New Insights into Tunable Volatility of Ionic Materials through Counter-Ion Control. *Adv. Funct. Mater.* **2016**, *26*, 3438–3445.

(5) Soriano-Díaz, I.; Ortí, E.; Giussani, A. On the importance of equatorial metal-centered excited states in the photophysics of cyclometallated Ir(iii) complexes. *Dalton Trans.* **2023**, *52*, 10437–10447.

(6) Wickramasinghe, W. A.; Bird, P. H.; Serpone, N. Nature of the '[Ir(bpy)2(H2O)(bpy)]3+', (bipy = 2,2-bipyridine) cation. Monodentate or covalently hydrated bipyridine ligand? X-Ray crystal structure of the perchlorate salt. *J. Chem. Soc., Chem. Commun.* **1981**, 1284–1286.

(7) Qin, L.-Q.; Zou, B.-Q.; Qin, Q.-P.; Wang, Z.-F.; Yang, L.; Tan, M.-X.; Liang, C.-J.; Liang, H. Highly cytotoxic, cyclometalated iridium(iii)-5-fluoro-8-quinolinol complexes as cancer cell mitochondriotropic agents. *New J. Chem.* **2020**, *44*, 7832–7837.

(8) Lee, P.-K.; Liu, H.-W.; Yiu, S.-M.; Louie, M.-W.; Lo, K. K.-W. Luminescent cyclometallated iridium(iii) bis(quinolylbenzaldehyde) diimine complexes—synthesis, photo-

physics, electrochemistry, protein cross-linking properties, cytotoxicity and cellular uptake. *Dalton Trans.* **2011**, *40*, 2180–2189.

(9) Demissie, T. B.; Ruud, K.; Hansen, J. H. DFT as a Powerful Predictive Tool in Photoredox Catalysis: Redox Potentials and Mechanistic Analysis. *Organometallics* **2015**, *34*, 4218–4228.

(10) Flamigni, L.; Barbieri, A.; Sabatini, C.; Ventura, B.; Barigelletti, F. In *Photochemistry and Photophysics of Coordination Compounds II*; Balzani, V., Campagna, S., Eds.; Springer, 2007; pp 143–203.

(11) Ladouceur, S.; Fortin, D.; Zysman-Colman, E. Enhanced Luminescent Iridium(III) Complexes Bearing Aryltriazole Cyclometallated Ligands. *Inorg. Chem.* **2011**, *50*, 11514–11526.

(12) Gao, X.; Bai, S.; Fazzi, D.; Niehaus, T.; Barbatti, M.; Thiel, W. Evaluation of Spin-Orbit Couplings with Linear-Response Time-Dependent Density Functional Methods. *J. Chem. Theory Comput.* **2017**, *13*, 515–524.

(13) Tamayo, A. B.; Alleyne, B. D.; Djurovich, P. I.; Lamansky, S.; Tsyba, I.; Ho, N. N.; Bau, R.; Thompson, M. E. Synthesis and Characterization of Facial and Meridional Tris-cyclometalated Iridium(III) Complexes. *J. Am. Chem. Soc.* **2003**, *125*, 7377–7387.

(14) Neese, F. The ORCA program system. *WIREs Comput. Mol. Sci.* **2012**, *2*, 73–78.

(15) Neese, F. Software Update: The ORCA Program System—Version 6.0. *WIREs Comput. Mol. Sci.* **2025**, *15*, e70019.

(16) Johnson, E. R.; Becke, A. D. A post-Hartree–Fock model of intermolecular interactions. *J. Chem. Phys.* **2005**, *123*, 024101.

(17) Johnson, E. R.; Becke, A. D. A post-Hartree-Fock model of intermolecular interactions: Inclusion of higher-order corrections. *J. Chem. Phys.* **2006**, *124*, 174104.

(18) Becke, A. D.; Johnson, E. R. A density-functional model of the dispersion interaction. *J. Chem. Phys.* **2005**, *123*, 154101.

(19) Barone, V.; Cossi, M. Quantum Calculation of Molecular Energies and Energy Gradients in Solution by a Conductor Solvent Model. *J. Phys. Chem. A* **1998**, *102*, 1995–2001.
